# JNK Inhibition Overcomes Resistance of Metastatic Tetraploid Cancer Cells to Irradiation-Induced Apoptosis

**DOI:** 10.3390/ijms26031209

**Published:** 2025-01-30

**Authors:** Mohamed Jemaà, Nouha Setti Boubaker, Nesrine Kerkeni, Stephan M. Huber

**Affiliations:** 1 Human Genetics Laboratory LR99ES10, Faculty of Medicine of Tunis, Tunis El Manar University, Tunis 2092, Tunisia; 2 Neurophysiology, Cellular Physiopathology and Valorisation of Biomolecules Laboratory LR18ES03, Faculty of Sciences of Tunis, Tunis El Manar University, Tunis 2092, Tunisia; 3 Department of Biology, Faculty of Sciences of Tunis, Tunis El Manar University, Tunis 2092, Tunisia; 4 Urology Department, Charles Nicolle Hospital, Faculty of Medicine, Tunis El Manar University, Tunis 2092, Tunisia; nouha.setti@hotmail.fr; 5 Theranostic Biomarkers Laboratory, Faculty of Medicine of Tunis, University Tunis El Manar, Tunis 2092, Tunisia; 6 Department of Radiation Oncology, University of Tübingen, 72076 Tübingen, Germany

**Keywords:** polyploidy, colon carcinoma, irradiation, SP600125, combination, gene ontology, DNA damage, JNK pathway

## Abstract

Tetraploidy is a condition in which the entire set of chromosomes doubles, most often due to errors during cell division. Tetraploidy can lead to genomic instability and significant consequences, in particular metastasis and treatment failure in tumours, including radiotherapy. The development of new strategies to sensitise these cells to treatment is of great importance. In our study, we investigated the in vitro combination of chemical treatment with the kinase inhibitor SP600125 and irradiation on diploid versus metastatic tetraploid RKO colon cancer clones. We assessed mitochondrial transmembrane potential, cell cycle and subG1 population by flow cytometry and performed clonogenic assays to evaluate cell sensitivity. We found that the combination overcomes irradiation resistance in metastatic tetraploid clones. To identify the main pathway involved in cell sensitivity, we screened the Harvard Medical School KINOMEscan library and performed a gene ontology biological process analysis. We found that the major kinases inhibited by SP600125 were ANKK1, BIKE, IKKA, JNK1, MP2K3, MP2K4, MKNK2, MYLK, PLK4, RPS6KA4(Kin,Dom,1), MYLK4 and TTK, and the pathways involved in clone sensitivity were DNA damage repair, radiation resistance and apoptosis, through JNK pathway inhibition. Finally, our main finding was that combined treatment with SP600125 and radiotherapy reduced the resistance of metastatic tetraploid cells to treatment, essentially by inhibiting the JNK pathway. This result supports a promising anti-cancer strategy to overcome the resistance of tetraploid cancer cells to irradiation.

## 1. Introduction

Tetraploidy is a condition with a double set of chromosomes, twice as many as their normal diploid counterparts. Except in certain physiological states, cells do not tolerate tetraploidy and activate programmed death pathways leading to their elimination [1,2].

Indeed, tetraploidy is an unhealthy and transitional phase that leads to polyploidy, and then asymmetric cell divisions and chromosome loss, causing aneuploidy and chromosomal instability [3]. Tetraploidy can be formed by endoreplication, i.e., DNA replication without mitosis, or by endomitosis, i.e., karyokinesis without cytokinesis. Tetraploidy also occurs during mitotic failure [3,4]. Tetraploid cells can also be generated by cell fusion [5]. A documented example is fusion between tumour cells and infiltrating cells of myeloid origin, which may contribute to cancer plasticity [6,7]. This unhealthy situation is facilitated by a genetic defect, in particular by p53-deficient protein or downstream pathways [8,9,10]. It is interesting to note that several studies have shown that metastatic tumours contain a significant proportion of tetraploid cells compared to primary tumours [11]. Our most recent study has confirmed, both in vitro and in vivo, that tetraploid cancer cells are a driving force in cancer metastasis [12].

On the other side, tetraploidy is correlated with tumourigenesis, cancer growth and aggressiveness, as well as therapeutic failure and poor patient prognosis [5,12]. In addition, the tetraploid status of cancer cells confers resistance to genotoxic stress, either induced by ionising radiation or by genotoxic agents used in chemotherapy, such as platinum compounds like cisplatin or oxaliplatin and topoisomerase inhibitors like camptothecin [13]. One of the most direct consequences of this is resistance to apoptosis-inducing regimes [14,15], which might contribute to chemotherapeutic failure and could explain metastasis.

Previous studies showed that radiotherapy induces epithelial–mesenchymal transition and stemness, leading to increased invasion and metastasis of cancer cells surviving after primary radiotherapy [16]. This could explain the link between radioresistance and metastasis in tetraploid cancer cells.

Interestingly, tetraploid cancer cells showed sensitivity to some mitotic inhibitors, essentially inhibitors of Checkpoint kinase 1 (CHK1), Aurora B, Kinesin-5 (Eg5), Polo Like Kinase 1 (Plk1) and Monopolar spindle 1 (Mps1 also named threonine tyrosine kinase TTK) [17]. One of these mitotic inhibitors is SP600125, (anthra[1,9-cd]pyrazol-6(2H)-one), which has been used to inhibit Mps1 in several studies [18,19,20,21]. We have previously shown that SP600125 at 30 uM induces apoptosis in tetraploid colon cancer cells more selectively than in diploid cells [22].

Within this frame, our work proposes to investigate SP600125 with ionising radiation on the survival of a diploid and metastatic tetraploid cancer cells.

## 2. Results

### 2.1. Metastatic Tetraploid Cells Are More Resistant to DNA Damage Induced by Radiation

**Figure 1:**
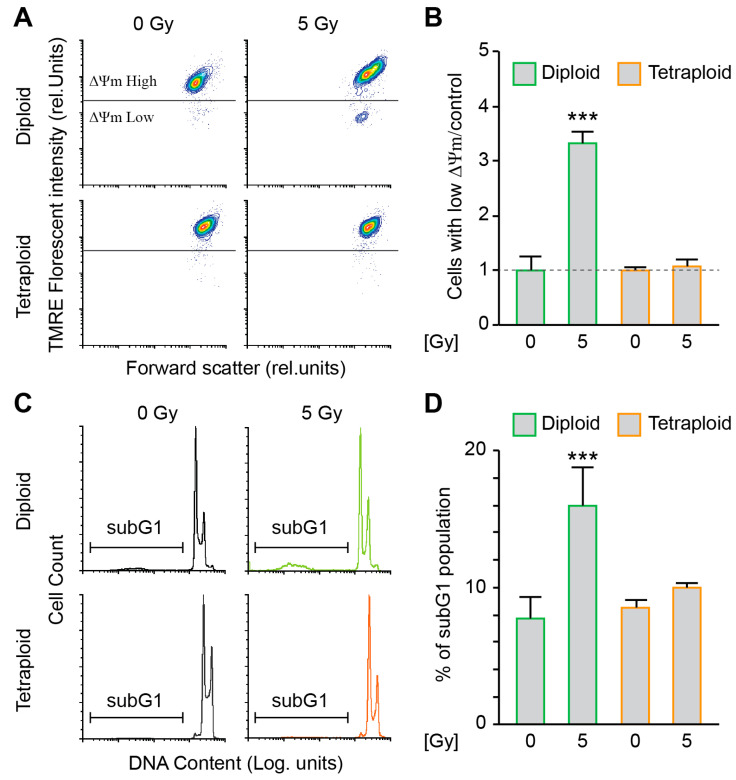
Metastatic tetraploid colon cancer cells are resistant to apoptosis induced by radiation. (**A**,**B**) Diploid and metastatic tetraploid human colon carcinoma RKO cells (framed in green and orange, respectively) were left untreated or irradiated with 5 Gy. After 48 h, the cells were stained with TMRE for the assessment of the dissipation of the mitochondrial inner transmembrane potential (Δψm) by flow cytometry. Representative plots are showed in panel (**A**), while quantitative data are represented in panel (**B**). Data ware normalised to the diploid control condition. (**C**,**D**) Diploid and metastatic tetraploid RKO cells (framed in green and orange, respectively) were left untreated or irradiated with 5 Gy. After 48 h, the cells were labelled with the DNA dye propidium iodide, for the quantification of the hypodiploid, subG1 apoptotic population by flow cytometry. Representative histograms are showed in panel (**C**), while quantitative data are represented in panel (**D**). The data are reported as means ± SEM (n ≥ 3). *** (*p* < 0.001) indicates significant differences compared to all conditions (ANOVA).

**Figure 2:**
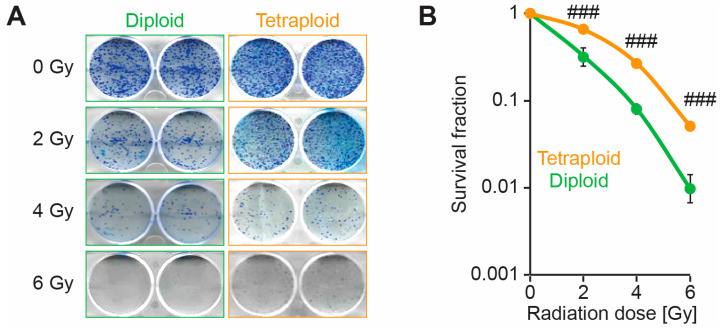
Metastatic tetraploid colon cancer cells are resistant to radiation. Pre-plating colony formation of diploid (left and labelled in green) and metastatic tetraploid (right and labelled in orange) human colon carcinoma RKO cells after irradiation with 0, 2, 4 and 6 Gy, respectively, and cultured for 2 weeks. Representative cut-outs of 6-well plates with Coomassie-stained cells are showed in panel (**A**), while quantitative data are represented in panel (**B**). The data are reported as means ± SEM (n ≥ 3). ### (*p* < 0.001) indicates significant differences compared to diploid cells (ANOVA).

In order to evaluate the sensitivity of the diploid and metastatic tetraploid cells to DNA damage, we decided to study their stress response to ionising radiation. First, we compared the dissipation of the mitochondrial inner transmembrane potential (Δψm) and DNA fragmentation by flow cytometry experiments as a measure of intrinsic apoptosis, between control (0 Gy) and irradiated (5 Gy) diploid and tetraploid RKO cells at 48h post-irradiation. Diploid cells were particularly more sensitive to radiation than tetraploid cells, as demonstrated by the elevated mitochondrial potential dissipation (Figure 1A,B) and larger cell fraction with hypodiploid (subG1) DNA content (Figure 1C,D). To further study the response of diploid and metastatic tetraploid cells to irradiation, we performed a clonogenic survival assay using increasing doses of MV photon (0 to 6 Gy). As a result, metastatic tetraploid cells were more resistant to radiation than diploid cells (Figure 2A,B). Radiation significantly decreased the survival fraction of diploid cells already starting at 2 Gy, which is the clinically relevant dose applied during fractionated radiotherapy [23]. These finding stipulate that metastatic tetraploid cells are more resistant to DNA damage induced by radiation compared to diploid cells.

### 2.2. SP600125 Administration Sensitises Metastatic Tetraploid Cells to Radiation

**Figure 3:**
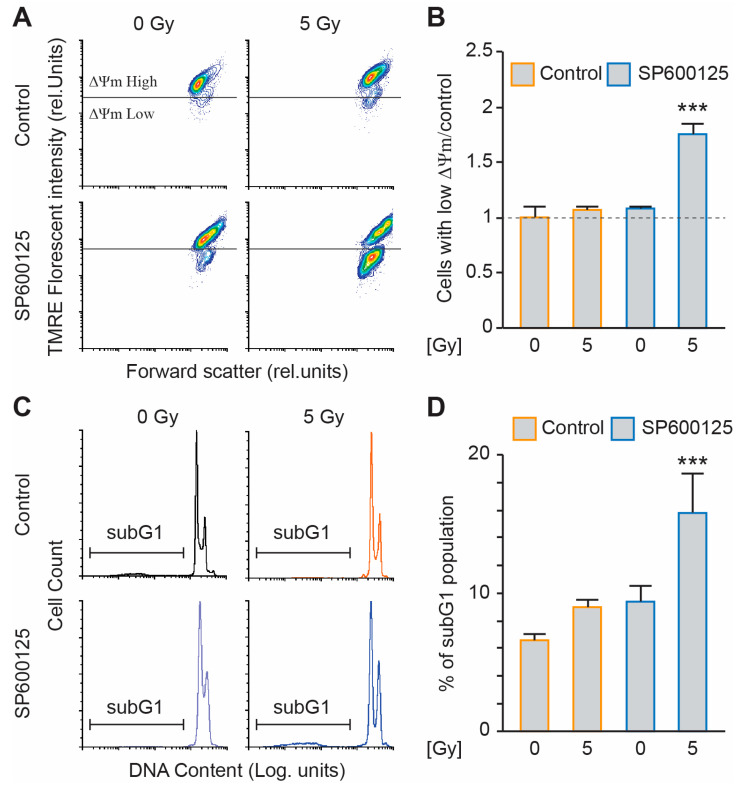
SP600125 overcomes radioresistance of metastatic tetraploid cells. (**A**,**B**) Metastatic tetraploid human colon carcinoma RKO cells were left untreated or irradiated with 5 Gy. Then, the cells were treated with 10μM SP600125 (labelled in bleu) or left untreated (labelled in orange). After 48 h, the cells were stained with TMRE for the assessment of the dissipation of the mitochondrial inner transmembrane potential (Δψm) by flow cytometry. Representative plots are showed in panel (**A**), while quantitative data are represented in panel (**B**). The data ware normalised to the tetraploid control condition. (**C**,**D**) The tetraploid RKO cells were left untreated or irradiated with 5 Gy. Then, the cells were treated with 10 μM SP600125 (labelled in bleu) or left untreated (labelled in orange). After 48h, the cells were fixed with ethanol and labelled with the DNA dye propidium iodide, for the quantification of the hypodiploid, subG1 apoptotic population by flow cytometry. Representative histograms are showed in panel (**C**), while quantitative data are represented in panel (**D**). The data are reported as means ± SEM (n ≥ 3). *** (*p* < 0.001) indicates significant differences compared to all conditions (ANOVA).

We previously demonstrated that SP600125 affects the survival of colon tetraploid cells [22]. We wondered then if combining radiation and SP600125 treatment could sensitise our metastatic tetraploid clones. First, we performed a clonogenic survival assay with the tetraploid clones using increasing doses of MV photon (0 to 8 Gy) in combination or not with 10 µM SP600125 treatment. The combination showed a better toxicity compared to SP600125 treatment alone (Figure S1). We decided to continue with the 5 Gy radiation dose. We treated the metastatic tetraploid cells with 0 or 10 µM SP600125 and radiated them with 0 or 5 Gy MV photon. We compared, using flow cytometry experiments, the dissipation of the mitochondrial inner transmembrane potential (Δψm) and DNA fragmentation as a measure of intrinsic apoptosis, between control (0 Gy) and irradiated (5 Gy) metastatic tetraploid RKO cells in the presence or absence of SP600125 at 48 h post-irradiation. Compared to the control, cells treated with 10 µM SP600125 did not show any apoptotic death. However, when combined to radiation, the cells displayed mitochondrial potential loss (Figure 3A,B) and hypodiploid (subG1) DNA content accumulation (Figure 3C,D).

### 2.3. SP600125 Target the JNK Pathway to Sensitise Metastatic Tetraploid Cells

**Figure 4:**
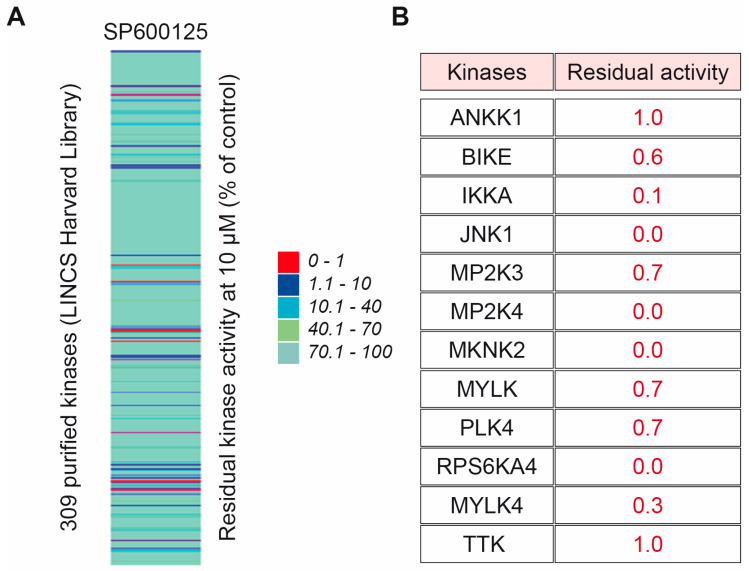
KINOMEscan to identify the kinase targeted when treated with 10 μM SP600125 (A–B). We took advantage of the Harvard Library of Integrated Network-based Cellular Signatures LINCS (http://lincs.hms.harvard.edu/ accessed on 22 September 2024) and its kinase profiler screen. It is based on a competition-binding assay performed for a compound of interest against each of a panel of 309 kinases. We set our threshold at 99% inhibition, meaning that compared to DMSO-treated kinases, the residual activity of the kinase should be equal to or less than 1%. The heat map in panel (**A**) shows the residual kinase activity of all 309 kinases in the assay (% of control DMSO). A full list of the genes can be found in Supplementary Table S1. The scale ranges from 0% (red) to 100% (blue). The table in panel (**B**) shows the proteins that were more than 99% inhibited by SP600125.

**Figure 5:**
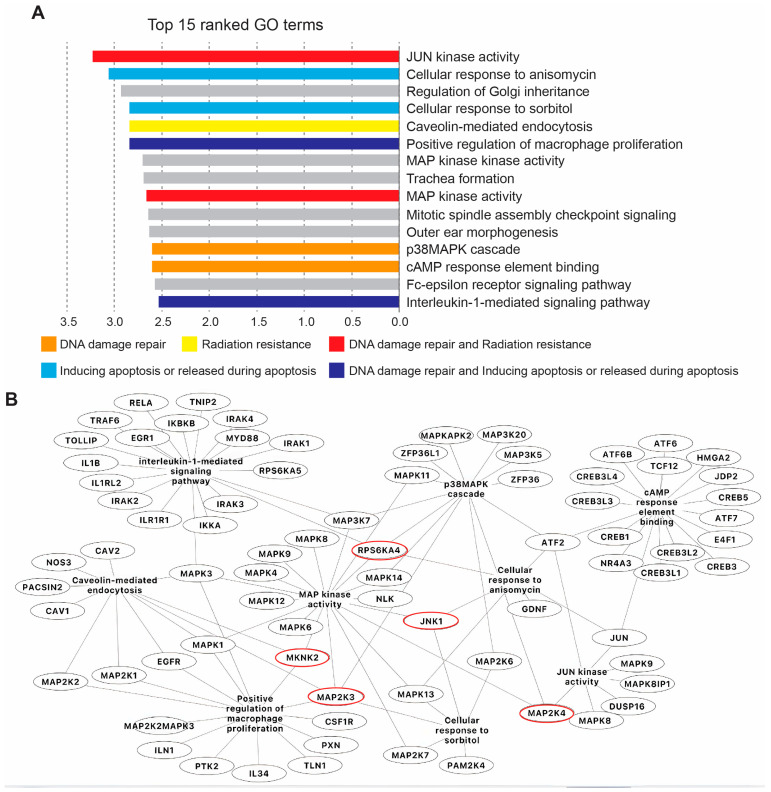
Gene ontology (GO) analysis associated with SP600125 target proteins. (**A**) Top 15 ranked GO terms commonly enriched between selected kinases according to their strength score, performed by the Cytoscape associated plugin ‘ClueGO’. (**B**) Merged network of gene interactions involved in common GO pathways associated with DNA damage repair, radioresistance and apoptosis. Red circles highlight genes listed in the table Figure 4.

SP600125 is a well-documented inhibitor of serine/threonine kinases [20]. To identify SP600125 potential targets, we took advantage of the KINOMEscan library, an online biochemical kinase-profiling assay that measures drug binding using a panel of 309 purified kinases (https://lincs.hms.harvard.edu/kinomescan/ accessed on 22 September 2024). Based on the values obtained from the KINOMEscan library using a 10 μM dose of SP600125 as used in our present study (Supplementary Table S1), a heatmap of specificity was generated. The results are expressed as a “percent of control”, where the control is dimethyl sulfoxide (DMSO). A value of 100% indicates no inhibition of kinase binding to the ligand in the presence of SP600125, while a low percentage indicates strong inhibition (Figure 4A). Then, 99% of inhibition was set as a cut-off. Accordingly, we found that several kinases were potentially highly inhibited, namely Ankyrin repeat and kinase domain containing I (ANKK1); BMP2 inducible kinase (BIKE); inhibitory-κB kinase (IKK) α (IKKA); c-Jun N-terminal kinase 1 (JNK1); Mitogen-Activated Protein Kinase Kinase 3 (MP2K3); Mitogen-Activated Protein Kinase Kinase 4 (MP2K4); MAP kinase-interacting serine/threonine-protein kinase 2 (MKNK2); Myosin light-chain kinase (MYLK), Polo Like Kinase 4 (PLK4); Ribosomal protein S6 kinase (RPS6KA4(Kin,Dom,1)); Myosin light chain kinase family member 4 (MYLK4); and threonine tyrosine kinase (TTK), also named as Monopolar Spindle 1 (MPS1) (Figure 4B). These 12 kinases were our main focus for gene ontology (GO), KEGG and Reactome enrichment analyses as an attempt to identify the biological pathways that are potentially involved in the sensitisation of tetraploid cancer cells after SP600125 co-treatment with ionising radiation. Accordingly, numerous pathways were highlighted, including Positive regulation of dopamine uptake involved in synaptic transmission, Tyrosine metabolism, Neuroblast division in subventricular zone, Necroptotic signalling pathway, cAMP response element binding, Apoptosis—multiple species, JNK (c-Jun kinases) phosphorylation and activation mediated by activated human TAK1, Cellular response to sorbitol and Activation of the AP-1 family of transcription factors (Supplementary Table S2). Further, all GO terms were loaded into Cytoscape for network construction (Figure S2A). As shown by the molecular network, different signalling pathways are involved. The most commonly enriched pathways between ANKK1, BIKE, IKKA, JNK1, MP2K3, MP2K4, MKNK2, MYLK, PLK4, RPS6KA4, MYLK4 and TTK were singled out (Figure S2B), namely MAP kinase activity (GO:0004707), Positive regulation of JUN kinase activity (GO:0043507), p38MAPK cascade (GO:0038066), Positive regulation of MAP Kinase activity (GO:0043406), Cellular response to anisomycin (GO:0072740), Cellular senescence (GO:0090398), Lipopolysaccharide-mediated signalling pathway (GO:0031663), Cellular response to tumour necrosis factor (GO:0071356), Positive regulation of interleukin-12 production (GO:0032735) and Cellular response to reactive oxygen species (GO:0034614). Then, the top 15 GO terms and pathways were sorted according to their enrichment strength score values (Figure 5A). The most involved pathway was the JUN Kinase activity pathway, which is known to be associated with DNA damage repair and radiation resistance (Figure 5B) [24]. The majority of the other main pathways were also described to be involved in DNA damage repair, radiation resistance or apoptosis [25,26,27,28].

## 3. Discussion

In our study, we provided additional evidence that metastatic tetraploidy confers resistance to radiation-induced DNA damage in contrast to the diploid state. Indeed, the hallmarks of cell death and apoptosis, namely loss of mitochondrial potential, accumulation of hypodiploid DNA content and disrupted clonogenicity, were very pronounced in diploid cells compared to metastatic tetraploid cells when irradiated with up to 6 Gy. Our data confirm what has been reported in several previous studies [13,15,29]. Several lines of evidence have suggested that this abnormal event promotes the acquisition of a transformed phenotype; generates high genetic diversity reflected in the genesis of resistant clones; and may thus contribute to oncogenesis, therapeutic failure and more importantly metastasis [11,12,15,29,30]. Therefore, limiting the generation/survival of tetraploid cells may restore chromosomal stability, could be considered as an effective anti-cancer target and could prevent metastasis.

SP600125 (anthra[1,9-cd]pyrazol-6(2H)-one) was first documented as a specific and reversible ATP-competitive inhibitor of the MAPK (mitogen-activated protein kinase) c-Jun amino-terminal kinase (JNK) family [31]. However, it has been shown to target a wider range of proteins and kinases, including some key mitotic kinases, such as Mps1 or Aurora A for example [20], and is a de facto widely used agent that is suitable for disrupting various biological signalling processes, mainly in inflammation, neurodegeneration and cancer [32].

In this context, we have previously shown that SP600125 at high concentrations (30 µM) affects the survival of colon tetraploid cells [22]. It should also be noted that tetraploidy is promoted by p53 loss [8,10], and we have previously shown that SP600125 preferentially kills p53-deficient cancer cells, including RKO colon cancer [20]. More importantly, at 10 µM, we also previously showed that SP600125 blocks RKO colon cancer migration in vitro and metastasis in vivo [32].

Here, we tested whether a combination of radiation and treatment with a lower concentration of SP600125 could sensitise the metastatic tetraploid cells. At 10 µM, SP600125 did not kill metastatic tetraploid cancer cells, a contrario to diploid ones, but the combination of the drug and irradiation overcame resistance and the metastatic tetraploid cells underwent apoptosis. This result supports the potential use of combined ionising radiation-SP600125 mediated therapy to sensitise tumours with metastatic tetraploid cancer cell niches to radiotherapy, which is promising for the development of novel anti-cancer strategies and for overcoming tumour metastasis. Indeed, it has been documented that tetraploid cancer cells, once irradiated, adopt a reduction in apoptotic potential [13,15,33]. On the other hand, SP600125 is a well-known apoptosis inducer, in addition to G2/M phase arrest and endoreduplication, and then G1 phase arrest [34,35]. This could explain how the combination of ionising radiation and SP600125 leads to a disruption of the survival capacity of metastatic tetraploid cells and makes them sensitive to radiation. As reported in the literature, SP600125 is able to selectively induce cancer cell death by specifically targeting the pathogenic pathways of cancer development, primarily resistance to apoptosis [36]. In addition, tumour cells are known to respond to radiation-induced DNA damage by activating a complex network of DNA damage signalling and repair pathways that control cell fate, including survival, death and genome stability [37]. We therefore sought to identify potential signalling/metabolic cascades that may be required for the survival of tetraploid cells and therefore targeted by SP600125 to sensitise them to treatment.

**Figure 6:**
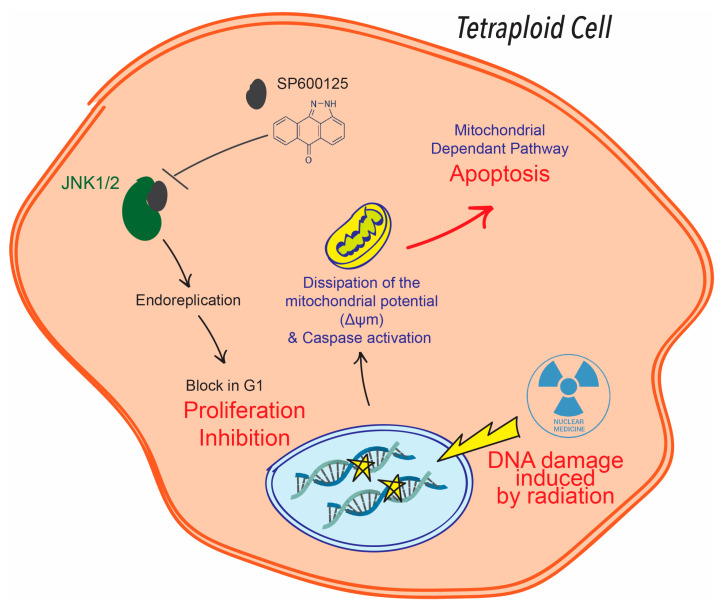
Presumed mechanism of the combination of irradiation and SP600125 treatment leading to apoptosis in tetraploid cells.

We identified SP600125 principal targets with the kinase-profiling assay KINOMEscan Harvard library, and then, we performed a gene ontology (GO) in silico enrichment analysis to identify the main pathways potentially involved in the sensitisation of metastatic tetraploid cancer cells after SP600125 co-treatment with ionising radiation. Interestingly, the most involved pathway was the JUN kinase pathway, which is known to be associated with DNA damage repair, apoptosis and radiation resistance [24]. Most importantly, SP600125 has been used to characterise the role of JNK in apoptotic pathways and survival potential [38,39]. Taken together, it seems reasonable to hypothesise that potential inhibition of the JNK pathway by SP600125 overcomes the resistance of tetraploid cancer cells to radiation-induced apoptosis (Figure 6). Most of the other keys signalling pathways have also been described to correlate with DNA damage repair, radiation resistance or apoptosis [25,26,27,28,40], which may further explain the reduction in survival and resistance of tetraploid cells in the combined SP600125-radiation context.

In line with our findings, we should highlight here that previous reports have demonstrated the synergistic effect of the combination of irradiation and JNK inhibition by SP600125 in vivo using Lewis lung carcinoma and mouse models, with subcutaneous xenograft or intracranial [41,42], and using patient-derived vestibular schwannoma cells [43] and in vitro using liver cancer HepG2 cells [44]. In all of these reports, SP600125 was used exclusively as a JNK inhibitor.

Our data and the literature thus provide complementary evidence that tetraploidy is associated with radiotherapy resistance; could explain the high ratio of polyploid/aneuploid cells in metastatic sites; and further advance the novel combined SP600125-ionising radiation strategy which is significantly efficient at reducing tetraploid cell survival and resistance and remarkably reliable at inducing sensitivity to cancer treatments through JNK inhibition, which may pave the way for the development of novel protocol for anti-cancer treatment.

## 4. Materials and Methods

### 4.1. Cell Lines, Culture Conditions and Reagents

Diploid and metastatic tetraploid clones derived from human colon carcinoma RKO cells [12] were routinely maintained in McCoy’s 5A medium supplemented with 10% foetal calf serum (FCS), 10 mM HEPES buffer, 100 units/mL penicillin G sodium and 100 μg/mL streptomycin sulphate (all provided from Thermo Fisher Scientific-Gibco, Waltham, MA, USA). The cells were routinely maintained at 37 °C under 5% CO_2_. SP600125 (Sigma-Aldrich, St Louis, MO, USA) was stocked as 10 mM solution in DMSO. The cells were seeded onto the appropriate supports (6- or 12-well plates) 24 h before the beginning of the experiments.

### 4.2. Ionising Radiation (IR)

Diploid and metastatic tetraploid RKO cells were irradiated (single doses of 0, 2, 4, 5, 6 and 8 Gy) with 6 MV photons by using a linear accelerator (LINAC SL25 Philips) at a dose rate of 4 Gy/min at room temperature. Open-field irradiation was applied. For a sufficient dose build-up in the plane of the cells, a RW3 slab (1 cm thick) was placed above the cells. Energy deposition at the cell plane was calibrated by film dosimetry. Following IR, the cells were post-incubated in the respective cell culture media for different analyses.

### 4.3. Clonogenic Survival Assay

The cells were seeded at low concentrations (1000 cells/well of 6-well plate) and were left untreated, treated with SP600125, irradiated (0, 2, 4, 6 or 8 Gy), or combined with SP600125 (0 and 10 µM in 0.1% DMSO, respectively) and irradiation. A washout was performed at 24 h and followed by culture in standard conditions for up to 2–3 weeks. The colonies were then fixed/stained with aqueous Coomassie blue and counted manually. The plating efficiency was defined by dividing the number of colonies by the number of plated cells. Survival fractions were calculated by dividing the plating efficiency of the irradiated/treated cells by those of the un-irradiated controls.

### 4.4. Cytofluorometric Studies

(i) The assessment of the mitochondrial transmembrane potential (Δψm) was performed as follows: cells of interest (SP600125, irradiation or combination) were cultured for 48 h, then trypsinised, washed and incubated for 30 min at room temperature in serum-free medium solution containing the ΔΨm-specific dye tetramethylrhodamine ethyl ester perchlorate (TMRE, 25 nM, Invitrogen, Karlsruhe, Germany). Fluorescence was measured by flow cytometry (FACS Calibur, Becton Dickinson, Heidelberg, Germany, 488 nm excitation wavelength) at the fluorescence channel FL-2 (585/42 nm) emission wavelength.

(ii) The assessment of the cell cycle and subG1 fraction was performed as follows: cells were trypsinised, washed, then permeabilised and stained (30 min at room temperature) with propidium iodide (PI) according to the Nicoletti protocol (containing 0.1% Na-citrate, 0.1% triton X-100, 10 μg/mL PI in RNase-containing phosphate-buffered saline, PBS). The DNA amount was analysed by flow cytometry in fluorescence channel FL-2 (585/42 nm, logarithmic scale). The data were analysed with the FCS Express 6 software (De Novo Software, Los Angeles, CA, USA).

### 4.5. Pathway Enrichment Analysis and Network Construction

Gene ontology (GO) biological process, reactome (REAC) and the Kyoto Encyclopedia of Genes Genomes (KEGG) pathway enrichment analyses were performed using The Search Tool for the Retrieval of Interacting Genes/Proteins (STRING) database (V.12.0) (https://string-db.org/ accessed on 01 October 2024) (Supplementary Table S2). These above-cited databases cluster genes depending on their relationships (GO) or their involvement in biological pathways (Reactome and KEGG). Furthermore, Cytoscape software (V.3.10.1) (https://cytoscape.org/ accessed on 01 October 2024) and a clueGO-related plugin were used to construct networks visualising all GO enriched terms and to explore the top 15 commonly enriched pathways.

### 4.6. Statistical Procedures

The data are expressed as arithmetic means ± SEM. The statistical analysis was made with GraphPad Prism using ANOVA.

## 5. Conclusions

In summary, we developed a novel strategy to target metastatic tetraploid cancer cells based on the combination of irradiation and treatment with the broad-spectrum kinase inhibitor SP600125. Using the kinase-profiling assay KINOMEscan Harvard library and gene ontology in silico analysis, we suggest that the positive regulation of the JUN kinase activity pathway, and more specifically, the inhibition of JNK kinase, is responsible for the sensitivity of tetraploid cells to irradiation. This may be relevant in the context of cancer therapy, as metastatic tetraploid cells are thought to be highly resistant to conventional anti-cancer therapies.

## Data Availability

The original contributions presented in this study are included in the article/Supplementary Material. Further inquiries can be directed to the corresponding authors.

## Supplementary Materials

The following supporting information can be downloaded at https://www.mdpi.com/article/10.3390/ijms26031209/s1.
