# Supplementary material for: JNK Inhibition Overcomes Resistance of Metastatic Tetraploid Cancer Cells to Irradiation-Induced Apoptosis
Source: Int J Mol Sci. 2025 Jan 30;26(3):1209. doi: 10.3390/ijms26031209 (PMC11818936; doi:10.3390/ijms26031209)
Supplement: Supplementary file 1 [file ijms-26-01209-s001.zip › ijms-3408280-supplementary/Supplementary files/Supplementary Figure S1-2.pdf]

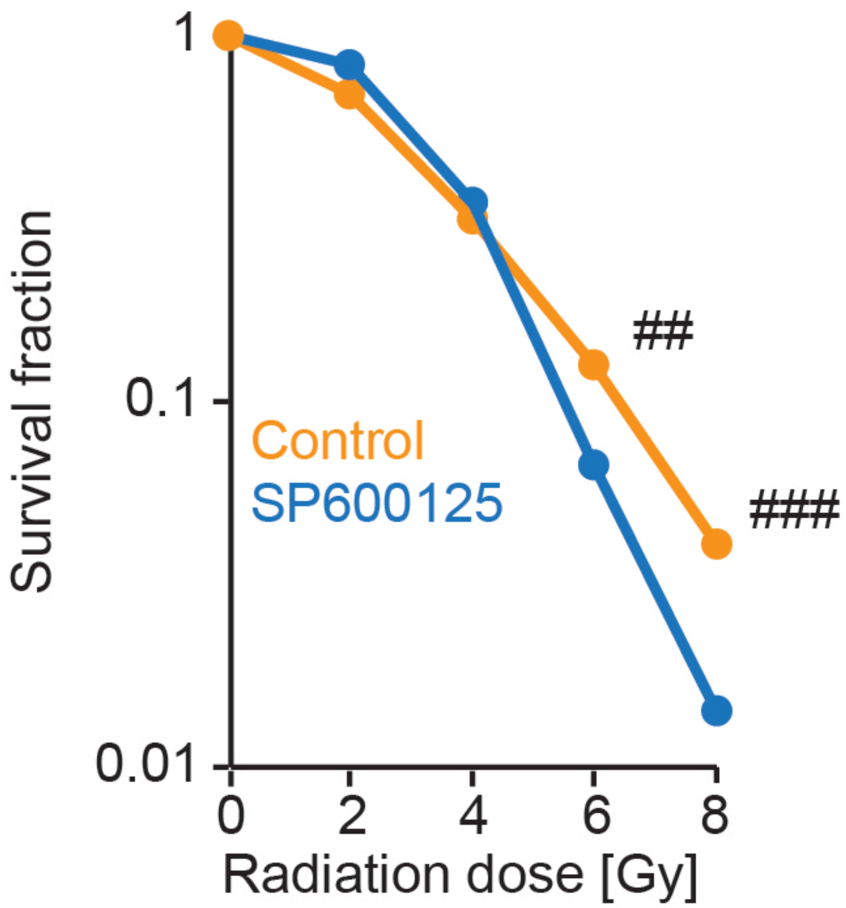

Figure S1. Co administration of SP600125 and radiation kills Tetraploid colon cancer cells.

Pre-plating colony formation of tetraploid human colon carcinoma RKO cells after treatment with 10 µM SP600125 (labelled in blue) or left untreated (labelled in orange) and then irradiation with 0, 2, 4, 6 or 8 Gy, respectively and cultured for 2 weeks.

Quantitative data are represented. Data are reported as means ± SEM (n = 3). ##($p < 0.01$) and ###($p < 0.001$) indicate significant difference compared to non treated with SP600125 cells (ANOVA).

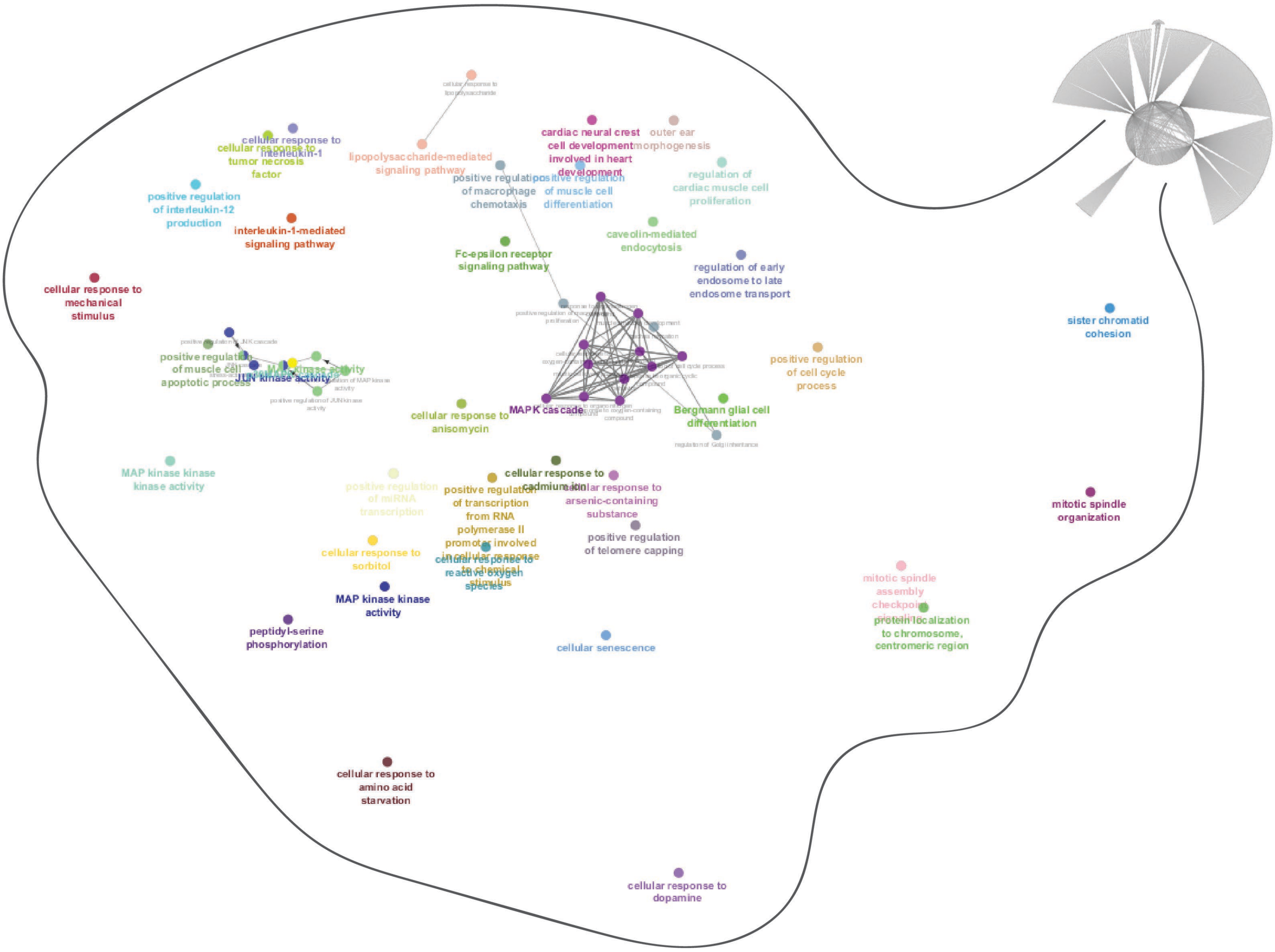

Figure S2. Principal pathways inhibited by SP600125

Interaction network of the Gene Ontology (GO) pathway enrichment analysis performed with the Cytoscape associated plugin 'ClueGO' plus top enriched GO terms of ANKK1, BIKE, IKKA, JNK1, MP2K3, MP2K4, MKNK2, MYLK, PLK4, RPS6KA4, MYLK4 and TTK
