# Supplementary material for: JNK Inhibition Overcomes Resistance of Metastatic Tetraploid Cancer Cells to Irradiation-Induced Apoptosis
Source: Int J Mol Sci. 2025 Jan 30;26(3):1209. doi: 10.3390/ijms26031209 (PMC11818936; doi:10.3390/ijms26031209)
Supplement: Supplementary file 1 [file ijms-26-01209-s001.zip › ijms-3408280-supplementary/Supplementary files/Supplementary Table 2.pdf]

Supplementary Table 2. Pathway Enrichment Analysis for GO, KEGG, STRING clusters and Reactome Terms within ANKK1 gene

#category	term ID	term description	observed gene count	background gene count	strength	false discovery rate	matching proteins in your network (IDs)	matching proteins in your network (labels)
GO Process	GO:0001505	Regulation of neurotransmitter levels	6	222	1.68	1.55e-05	9606.ENSP00000176183,9606.ENSP00000261707,9606.ENSP00000270349,9606.ENSP00000354511,9606.ENSP00000354859,9606.ENSP00000399745	DRD4,SLC6A4,SLC6A3,COMT,DRD2,CHRM2
GO Process	GO:0042417	Dopamine metabolic process	4	30	2.38	1.89e-05	9606.ENSP00000176183,9606.ENSP00000270349,9606.ENSP00000354511,9606.ENSP00000354859	DRD4,SLC6A3,COMT,DRD2
GO Process	GO:0042220	Response to cocaine	4	47	2.18	5.27e-05	9606.ENSP00000176183,9606.ENSP00000270349,9606.ENSP00000354859,9606.ENSP00000394624	DRD4,SLC6A3,DRD2,OPRM1
GO Process	GO:0048149	Behavioral response to ethanol	3	9	2.78	7.42e-05	9606.ENSP00000176183,9606.ENSP00000354859,9606.ENSP00000394624	DRD4,DRD2,OPRM1
GO Process	GO:0007193	Adenylate cyclase-inhibiting G protein-coupled receptor signaling pathway	4	74	1.99	0.00016	9606.ENSP00000176183,9606.ENSP00000354859,9606.ENSP00000394624,9606.ENSP00000399745	DRD4,DRD2,OPRM1,CHRM2
GO Process	GO:1901615	Organic hydroxy compound metabolic process	6	478	1.35	0.00017	9606.ENSP00000176183,9606.ENSP00000261733,9606.ENSP00000270349,9606.ENSP00000306606,9606.ENSP00000354511,9606.ENSP00000354859	DRD4,ALDH2,SLC6A3,ADH1B,COMT,DRD2
GO Process	GO:0001963	Synaptic transmission, dopaminergic	3	16	2.53	0.00021	9606.ENSP00000176183,9606.ENSP00000270349,9606.ENSP00000354859	DRD4,SLC6A3,DRD2
GO Process	GO:0042053	Regulation of dopamine metabolic process	3	20	2.43	0.00025	9606.ENSP00000176183,9606.ENSP00000261733,9606.ENSP00000270349	DRD4,ALDH2,SLC6A3
GO Process	GO:0015844	Monoamine transport	3	22	2.39	0.00027	9606.ENSP00000261707,9606.ENSP00000270349,9606.ENSP00000354859	SLC6A4,SLC6A3,DRD2
GO Process	GO:0098810	Neurotransmitter reuptake	3	23	2.37	0.00029	9606.ENSP00000261707,9606.ENSP00000270349,9606.ENSP00000354859	SLC6A4,SLC6A3,DRD2

GO Process	GO:0099565	Chemical synaptic transmission, postsynaptic	4	114	1.8	0.00032	9606.ENSP00000176183,9606.ENSP00000354859,9606.ENSP00000394624,9606.ENSP00000399745	DRD4,DRD2,OPRM1,CHRM2
GO Process	GO:0045471	Response to ethanol	4	126	1.75	0.00045	9606.ENSP00000176183,9606.ENSP00000270349,9606.ENSP00000354859,9606.ENSP00000394624	DRD4,SLC6A3,DRD2,OPRM1
GO Process	GO:0007268	Chemical synaptic transmission	5	409	1.34	0.0011	9606.ENSP00000176183,9606.ENSP00000270349,9606.ENSP00000354859,9606.ENSP00000394624,9606.ENSP00000399745	DRD4,SLC6A3,DRD2,OPRM1,CHRM2
GO Process	GO:0014070	Response to organic cyclic compound	6	861	1.1	0.0016	9606.ENSP00000176183,9606.ENSP00000261707,9606.ENSP00000270349,9606.ENSP00000354859,9606.ENSP00000394624,9606.ENSP00000399745	DRD4,SLC6A4,SLC6A3,DRD2,OPRM1,CHRM2
GO Process	GO:0051586	Positive regulation of dopamine uptake involved in synaptic transmission	2	3	3.08	0.0016	9606.ENSP00000176183,9606.ENSP00000354859	DRD4,DRD2
GO Process	GO:0071407	Cellular response to organic cyclic compound	5	508	1.25	0.0025	9606.ENSP00000176183,9606.ENSP00000261707,9606.ENSP00000354859,9606.ENSP00000394624,9606.ENSP00000399745	DRD4,SLC6A4,DRD2,OPRM1,CHRM2
GO Process	GO:0010243	Response to organonitrogen compound	6	963	1.05	0.0026	9606.ENSP00000176183,9606.ENSP00000261707,9606.ENSP00000270349,9606.ENSP00000354859,9606.ENSP00000394624,9606.ENSP00000399745	DRD4,SLC6A4,SLC6A3,DRD2,OPRM1,CHRM2
GO Process	GO:0050709	Negative regulation of protein secretion	3	62	1.94	0.0027	9606.ENSP00000176183,9606.ENSP00000354859,9606.ENSP00000394624	DRD4,DRD2,OPRM1
GO Process	GO:0007195	Adenylate cyclase-inhibiting dopamine receptor signaling pathway	2	5	2.86	0.0028	9606.ENSP00000176183,9606.ENSP00000354859	DRD4,DRD2
GO Process	GO:0007187	G protein-coupled receptor signaling pathway, coupled to cyclic nucleotide second messenger	3	65	1.92	0.0029	9606.ENSP00000176183,9606.ENSP00000394624,9606.ENSP00000399745	DRD4,OPRM1,CHRM2
GO Proces	GO:0051583	Dopamine uptake involved in	2	6	2.78	0.0034	9606.ENSP00000270349,9606.ENSP00000354859	SLC6A3,DRD2

s		synaptic transmission						
GO Process	GO:0071417	Cellular response to organonitrogen compound	5	574	1.19	0.0034	9606.ENSP00000176183,9606.ENSP00000261707,9606.ENSP00000354859,9606.ENSP00000394624,9606.ENSP00000399745	DRD4,SLC6A4,DRD2,OPRM1,CHRM2
GO Process	GO:1903530	Regulation of secretion by cell	5	564	1.2	0.0034	9606.ENSP00000176183,9606.ENSP00000261707,9606.ENSP00000354859,9606.ENSP00000394624,9606.ENSP00000399745	DRD4,SLC6A4,DRD2,OPRM1,CHRM2
GO Process	GO:0007610	Behavior	5	598	1.18	0.0037	9606.ENSP00000176183,9606.ENSP00000261707,9606.ENSP00000270349,9606.ENSP00000354859,9606.ENSP00000394624	DRD4,SLC6A4,SLC6A3,DRD2,OPRM1
GO Process	GO:1990384	Hyaloid vascular plexus regression	2	7	2.71	0.0037	9606.ENSP00000270349,9606.ENSP00000354859	SLC6A3,DRD2
GO Process	GO:0048511	Rhythmic process	4	270	1.42	0.0039	9606.ENSP00000176183,9606.ENSP00000261707,9606.ENSP00000354859,9606.ENSP00000394624	DRD4,SLC6A4,DRD2,OPRM1
GO Process	GO:1901616	Organic hydroxy compound catabolic process	3	80	1.83	0.0040	9606.ENSP00000261733,9606.ENSP00000270349,9606.ENSP00000354511	ALDH2,SLC6A3,COMT
GO Process	GO:1903351	Cellular response to dopamine	3	81	1.82	0.0040	9606.ENSP00000176183,9606.ENSP00000354859,9606.ENSP00000399745	DRD4,DRD2,CHRM2
GO Process	GO:0007197	Adenylate cyclase-inhibiting G protein-coupled acetylcholine receptor signaling pathway	2	8	2.65	0.0041	9606.ENSP00000394624,9606.ENSP00000399745	OPRM1,CHRM2
GO Process	GO:0042420	Dopamine catabolic process	2	9	2.6	0.0045	9606.ENSP00000270349,9606.ENSP00000354511	SLC6A3,COMT
GO Process	GO:0007200	Phospholipase C-activating G protein-coupled receptor signaling pathway	3	97	1.74	0.0054	9606.ENSP00000354859,9606.ENSP00000394624,9606.ENSP00000399745	DRD2,OPRM1,CHRM2
GO Proces	GO:0034776	Response to histamine	2	12	2.47	0.0063	9606.ENSP00000176183,9606.ENSP00000354859	DRD4,DRD2

s								
GO Process	GO:0060078	Regulation of postsynaptic membrane potential	3	107	1.7	0.0065	9606.ENSP00000176183,9606.ENSP00000354859,9606.ENSP00000394624	DRD4,DRD2,OPRM1
GO Process	GO:0051588	Regulation of neurotransmitter transport	3	108	1.7	0.0066	9606.ENSP00000176183,9606.ENSP00000354859,9606.ENSP00000399745	DRD4,DRD2,CHRM2
GO Process	GO:0048148	Behavioral response to cocaine	2	13	2.44	0.0069	9606.ENSP00000176183,9606.ENSP00000354859	DRD4,DRD2
GO Process	GO:0060134	Prepulse inhibition	2	13	2.44	0.0069	9606.ENSP00000270349,9606.ENSP00000354859	SLC6A3,DRD2
GO Process	GO:0051481	Negative regulation of cytosolic calcium ion concentration	2	15	2.38	0.0085	9606.ENSP00000354859,9606.ENSP00000394624	DRD2,OPRM1
GO Process	GO:0021984	Adenohypophysis development	2	17	2.32	0.0101	9606.ENSP00000270349,9606.ENSP00000354859	SLC6A3,DRD2
GO Process	GO:0050877	Nervous system process	6	1423	0.88	0.0101	9606.ENSP00000176183,9606.ENSP00000261707,9606.ENSP00000270349,9606.ENSP00000354859,9606.ENSP00000394624,9606.ENSP00000399745	DRD4,SLC6A4,SLC6A3,DRD2,OPRM1,CHRM2
GO Process	GO:1901386	Negative regulation of voltage-gated calcium channel activity	2	19	2.28	0.0114	9606.ENSP00000176183,9606.ENSP00000354859	DRD4,DRD2
GO Process	GO:0006067	Ethanol metabolic process	2	21	2.23	0.0131	9606.ENSP00000261733,9606.ENSP00000306606	ALDH2,ADH1B
GO Process	GO:1901700	Response to oxygen-containing compound	6	1547	0.84	0.0141	9606.ENSP00000176183,9606.ENSP00000261707,9606.ENSP00000270349,9606.ENSP00000354859,9606.ENSP00000394624,9606.ENSP00000399745	DRD4,SLC6A4,SLC6A3,DRD2,OPRM1,CHRM2
GO Proces	GO:0006820	Anion transport	4	469	1.18	0.0165	9606.ENSP00000176183,9606.ENSP00000261707,9606.ENSP00000270349,9606.ENS	DRD4,SLC6A4,SLC6A3,DRD2

s							P00000354859	
GO Process	GO:0015850	Organic hydroxy compound transport	3	163	1.52	0.0165	9606.ENSP00000261707,9606.ENSP00000270349,9606.ENSP00000354859	SLC6A4,SLC6A3,DRD2
GO Process	GO:0050482	Arachidonic acid secretion	2	26	2.14	0.0183	9606.ENSP00000176183,9606.ENSP00000354859	DRD4,DRD2
GO Process	GO:0043278	Response to morphine	2	27	2.12	0.0192	9606.ENSP00000354859,9606.ENSP00000394624	DRD2,OPRM1
GO Process	GO:2001257	Regulation of cation channel activity	3	185	1.46	0.0213	9606.ENSP00000176183,9606.ENSP00000354859,9606.ENSP00000394624	DRD4,DRD2,OPRM1
GO Process	GO:0065008	Regulation of biological quality	8	3654	0.59	0.0215	9606.ENSP00000176183,9606.ENSP00000261707,9606.ENSP00000270349,9606.ENSP00000306606,9606.ENSP00000354511,9606.ENSP00000354859,9606.ENSP00000394624,9606.ENSP00000399745	DRD4,SLC6A4,SLC6A3,ADH1B,COMT,DRD2,OPRM1,CHRM2
GO Process	GO:0010039	Response to iron ion	2	31	2.06	0.0230	9606.ENSP00000270349,9606.ENSP00000354859	SLC6A3,DRD2
GO Process	GO:0001975	Response to amphetamine	2	32	2.05	0.0237	9606.ENSP00000176183,9606.ENSP00000354859	DRD4,DRD2
GO Process	GO:1901701	Cellular response to oxygen-containing compound	5	1057	0.93	0.0237	9606.ENSP00000176183,9606.ENSP00000261707,9606.ENSP00000354859,9606.ENSP00000394624,9606.ENSP00000399745	DRD4,SLC6A4,DRD2,OPRM1,CHRM2
GO Process	GO:0007626	Locomotory behavior	3	198	1.43	0.0238	9606.ENSP00000176183,9606.ENSP00000270349,9606.ENSP00000354859	DRD4,SLC6A3,DRD2
GO Process	GO:0040018	Positive regulation of multicellular organism growth	2	33	2.04	0.0242	9606.ENSP00000270349,9606.ENSP00000354859	SLC6A3,DRD2
GO Process	GO:0098664	G protein-coupled serotonin receptor signaling pathway	2	33	2.04	0.0242	9606.ENSP00000176183,9606.ENSP00000399745	DRD4,CHRM2
GO Proces	GO:0006811	Ion transport	5	1214	0.87	0.0391	9606.ENSP00000176183,9606.ENSP00000261707,9606.ENSP00000270349,9606.ENS	DRD4,SLC6A4,SLC6A3,DRD2,OPRM1

s							P00000354859,9606.ENSP00000394624	
GO Process	GO:0030001	Metal ion transport	4	649	1.04	0.0410	9606.ENSP00000261707,9606.ENSP00000270349,9606.ENSP00000354859,9606.ENSP00000394624	SLC6A4,SLC6A3,DRD2,OPRM1
GO Process	GO:0098662	Inorganic cation transmembrane transport	4	649	1.04	0.0410	9606.ENSP00000261707,9606.ENSP00000270349,9606.ENSP00000354859,9606.ENSP00000394624	SLC6A4,SLC6A3,DRD2,OPRM1
GO Process	GO:0035094	Response to nicotine	2	48	1.87	0.0422	9606.ENSP00000270349,9606.ENSP00000354859	SLC6A3,DRD2
GO Process	GO:0045744	Negative regulation of G protein-coupled receptor signaling pathway	2	49	1.86	0.0429	9606.ENSP00000354859,9606.ENSP00000394624	DRD2,OPRM1
GO Process	GO:0035176	Social behavior	2	52	1.84	0.0470	9606.ENSP00000176183,9606.ENSP00000261707	DRD4,SLC6A4
GO Function	GO:0035240	Dopamine binding	3	7	2.89	7.64e-05	9606.ENSP00000176183,9606.ENSP00000270349,9606.ENSP00000354859	DRD4,SLC6A3,DRD2
GO Function	GO:0001591	Dopamine neurotransmitter receptor activity, coupled via Gi/Go	2	3	3.08	0.0047	9606.ENSP00000176183,9606.ENSP00000354859	DRD4,DRD2
GO Function	GO:0098960	Postsynaptic neurotransmitter receptor activity	3	75	1.86	0.0095	9606.ENSP00000176183,9606.ENSP00000354859,9606.ENSP00000399745	DRD4,DRD2,CHRM2
GO Function	GO:0008504	Monoamine transmembrane transporter activity	2	9	2.6	0.0128	9606.ENSP00000261707,9606.ENSP00000270349	SLC6A4,SLC6A3
GO Function	GO:0015378	Sodium:chloride symporter activity	2	10	2.55	0.0131	9606.ENSP00000261707,9606.ENSP00000270349	SLC6A4,SLC6A3
GO Function	GO:0043176	Amine binding	2	13	2.44	0.0181	9606.ENSP00000261707,9606.ENSP00000270349	SLC6A4,SLC6A3
GO Functi	GO:0005326	Neurotransmitter transmembrane	2	19	2.28	0.0243	9606.ENSP00000261707,9606.ENSP00000270349	SLC6A4,SLC6A3

on		transporter activity						
GO Component	GO:0099056	Integral component of presynaptic membrane	5	75	2.08	9.00e-07	9606.ENSP00000261707,9606.ENSP00000270349,9606.ENSP00000354859,9606.ENSP00000394624,9606.ENSP00000399745	SLC6A4,SLC6A3,DRD2,OPRM1,CHRM2
GO Component	GO:0099055	Integral component of postsynaptic membrane	5	118	1.88	2.67e-06	9606.ENSP00000261707,9606.ENSP00000270349,9606.ENSP00000354859,9606.ENSP00000394624,9606.ENSP00000399745	SLC6A4,SLC6A3,DRD2,OPRM1,CHRM2
GO Component	GO:0098794	Postsynapse	6	621	1.24	9.28e-05	9606.ENSP00000176183,9606.ENSP00000261707,9606.ENSP00000270349,9606.ENSP00000354859,9606.ENSP00000394624,9606.ENSP00000399745	DRD4,SLC6A4,SLC6A3,DRD2,OPRM1,CHRM2
GO Component	GO:0043005	Neuron projection	7	1391	0.95	0.00042	9606.ENSP00000176183,9606.ENSP00000261707,9606.ENSP00000270349,9606.ENSP00000354511,9606.ENSP00000354859,9606.ENSP00000394624,9606.ENSP00000399745	DRD4,SLC6A4,SLC6A3,COMT,DRD2,OPRM1,CHRM2
GO Component	GO:0036477	Somatodendritic compartment	6	855	1.1	0.00045	9606.ENSP00000176183,9606.ENSP00000270349,9606.ENSP00000354511,9606.ENSP00000354859,9606.ENSP00000394624,9606.ENSP00000399745	DRD4,SLC6A3,COMT,DRD2,OPRM1,CHRM2
GO Component	GO:0042995	Cell projection	8	2379	0.78	0.00085	9606.ENSP00000176183,9606.ENSP00000261707,9606.ENSP00000270349,9606.ENSP00000306678,9606.ENSP00000354511,9606.ENSP00000354859,9606.ENSP00000394624,9606.ENSP00000399745	DRD4,SLC6A4,SLC6A3,ANKK1,COMT,DRD2,OPRM1,CHRM2
GO Component	GO:0030425	Dendrite	5	624	1.16	0.0018	9606.ENSP00000176183,9606.ENSP00000354511,9606.ENSP00000354859,9606.ENSP00000394624,9606.ENSP00000399745	DRD4,COMT,DRD2,OPRM1,CHRM2
GO Component	GO:0030424	Axon	5	651	1.14	0.0019	9606.ENSP00000270349,9606.ENSP00000354511,9606.ENSP00000354859,9606.ENSP00000394624,9606.ENSP00000399745	SLC6A3,COMT,DRD2,OPRM1,CHRM2
GO Component	GO:0098691	Dopaminergic synapse	2	11	2.51	0.0025	9606.ENSP00000270349,9606.ENSP00000354859	SLC6A3,DRD2
GO Component	GO:0043679	Axon terminus	3	114	1.67	0.0035	9606.ENSP00000270349,9606.ENSP00000354859,9606.ENSP00000399745	SLC6A3,DRD2,CHRM2
GO Comp	GO:0043025	Neuronal cell body	4	502	1.15	0.0109	9606.ENSP00000270349,9606.ENSP00000354859,9606.ENSP00000394624,9606.ENS	SLC6A3,DRD2,OPRM1,CHRM2

onent							P00000399745	
GO Component	GO:0005887	Integral component of plasma membrane	6	1706	0.8	0.0114	9606.ENSP00000176183,9606.ENSP00000261707,9606.ENSP00000270349,9606.ENSP00000354859,9606.ENSP00000394624,9606.ENSP00000399745	DRD4,SLC6A4,SLC6A3,DRD2,OPRM1,CHRM2
GO Component	GO:0045121	Membrane raft	3	320	1.23	0.0445	9606.ENSP00000261707,9606.ENSP00000270349,9606.ENSP00000394624	SLC6A4,SLC6A3,OPRM1
GO Component	GO:0098978	Glutamatergic synapse	3	334	1.21	0.0472	9606.ENSP00000176183,9606.ENSP00000354859,9606.ENSP00000399745	DRD4,DRD2,CHRM2
STRING clusters	CL:23582	Serotonin receptors, and Neurotransmitter disorders	7	29	2.64	5.47e-14	9606.ENSP00000176183,9606.ENSP00000261707,9606.ENSP00000270349,9606.ENSP00000306678,9606.ENSP00000354511,9606.ENSP00000354859,9606.ENSP00000419652	DRD4,SLC6A4,SLC6A3,ANKK1,COMT,DRD2,TTC12
STRING clusters	CL:23585	Amine binding, and Neurotransmitter disorders	5	23	2.59	1.67e-09	9606.ENSP00000176183,9606.ENSP00000261707,9606.ENSP00000270349,9606.ENSP00000354511,9606.ENSP00000354859	DRD4,SLC6A4,SLC6A3,COMT,DRD2
STRING clusters	CL:23587	Neurotransmitter disorders, and Autosomal recessive nonsyndromic deafness 63	3	11	2.69	3.58e-05	9606.ENSP00000270349,9606.ENSP00000354511,9606.ENSP00000354859	SLC6A3,COMT,DRD2
STRING clusters	CL:23599	Aminergic neurotransmitter loading into synaptic vesicle, and Pathological gambling	2	5	2.86	0.0039	9606.ENSP00000270349,9606.ENSP00000354859	SLC6A3,DRD2
STRING clusters	CL:9785	Alcohol dehydrogenase (NAD+) activity	2	6	2.78	0.0045	9606.ENSP00000261733,9606.ENSP00000306606	ALDH2,ADH1B
KEGG	hsa04728	Dopaminergic synapse	4	126	1.75	0.00019	9606.ENSP00000176183,9606.ENSP00000270349,9606.ENSP00000354511,9606.ENSP00000354859	DRD4,SLC6A3,COMT,DRD2

KEGG	hsa04080	Neuroactive ligand-receptor interaction	4	329	1.34	0.0040	9606.ENSP00000176183,9606.ENSP00000354859,9606.ENSP00000394624,9606.ENSP00000399745	DRD4,DRD2,OPRM1,CHRM2
KEGG	hsa00350	Tyrosine metabolism	2	35	2.01	0.0209	9606.ENSP00000306606,9606.ENSP00000354511	ADH1B,COMT
KEGG	hsa00071	Fatty acid degradation	2	42	1.93	0.0222	9606.ENSP00000261733,9606.ENSP00000306606	ALDH2,ADH1B
KEGG	hsa05030	Cocaine addiction	2	49	1.86	0.0239	9606.ENSP00000270349,9606.ENSP00000354859	SLC6A3,DRD2
KEGG	hsa00010	Glycolysis / Gluconeogenesis	2	64	1.75	0.0334	9606.ENSP00000261733,9606.ENSP00000306606	ALDH2,ADH1B
KEGG	hsa04721	Synaptic vesicle cycle	2	72	1.7	0.0359	9606.ENSP00000261707,9606.ENSP00000270349	SLC6A4,SLC6A3
Reactome	HSA-112311	Neurotransmitter clearance	4	10	2.86	1.20e-07	9606.ENSP00000261707,9606.ENSP00000261733,9606.ENSP00000270349,9606.ENSP00000354511	SLC6A4,ALDH2,SLC6A3,COMT
Reactome	HSA-375280	Amine ligand-binding receptors	3	42	2.11	0.0021	9606.ENSP00000176183,9606.ENSP00000354859,9606.ENSP00000399745	DRD4,DRD2,CHRM2
Reactome	HSA-380615	Serotonin clearance from the synaptic cleft	2	3	3.08	0.0022	9606.ENSP00000261707,9606.ENSP00000261733	SLC6A4,ALDH2
Reactome	HSA-379401	Dopamine clearance from the synaptic cleft	2	4	2.95	0.0024	9606.ENSP00000270349,9606.ENSP00000354511	SLC6A3,COMT
Reactome	HSA-390651	Dopamine receptors	2	5	2.86	0.0027	9606.ENSP00000176183,9606.ENSP00000354859	DRD4,DRD2
Reactome	HSA-373076	Class A/1 (Rhodopsin-like receptors)	4	326	1.34	0.0076	9606.ENSP00000176183,9606.ENSP00000354859,9606.ENSP00000394624,9606.ENSP00000399745	DRD4,DRD2,OPRM1,CHRM2
Reactome	HSA-71384	Ethanol oxidation	2	12	2.47	0.0076	9606.ENSP00000261733,9606.ENSP00000306606	ALDH2,ADH1B
Reactome	HSA-211859	Biological oxidations	3	215	1.4	0.0428	9606.ENSP00000261733,9606.ENSP00000306606,9606.ENSP00000354511	ALDH2,ADH1B,COMT

Table 2. Pathway Enrichment Analysis for GO, KEGG, STRING clusters and Reactome Terms within BIKE gene

#category	term ID	term description	observed gene	background gene count	strength	false discovery rate	matching proteins in your network (IDs)	matching proteins in your network (labels)

			count					
GO Process	GO:0006897	Endocytosis	7	447	1.45	1.57e-05	9606.ENSP00000372924,9606.ENSP00000377015,9606.ENSP00000392065,9606.ENSP00000403362,9606.ENSP00000429824,9606.ENSP00000470176,9606.ENSP00000483185	RALBP1,PICALM,REPS1,AP2M1,CLINT1,AP2S1,AP2B1
GO Process	GO:0072583	Clathrin-dependent endocytosis	4	31	2.36	2.14e-05	9606.ENSP00000377015,9606.ENSP00000403362,9606.ENSP00000470176,9606.ENSP00000483185	PICALM,AP2M1,AP2S1,AP2B1
GO Process	GO:0006898	Receptor-mediated endocytosis	5	166	1.73	8.09e-05	9606.ENSP00000377015,9606.ENSP00000392065,9606.ENSP00000403362,9606.ENSP00000470176,9606.ENSP00000483185	PICALM,REPS1,AP2M1,AP2S1,AP2B1
GO Process	GO:0098884	Postsynaptic neurotransmitter receptor internalization	3	7	2.89	8.09e-05	9606.ENSP00000403362,9606.ENSP00000470176,9606.ENSP00000483185	AP2M1,AP2S1,AP2B1
GO Process	GO:0030100	Regulation of endocytosis	5	209	1.63	0.00015	9606.ENSP00000334836,9606.ENSP00000377015,9606.ENSP00000403362,9606.ENSP00000451300,9606.ENSP00000470176	BMP2K,PICALM,AP2M1,NUMB,AP2S1
GO Process	GO:0031623	Receptor internalization	4	70	2.01	0.00015	9606.ENSP00000377015,9606.ENSP00000403362,9606.ENSP00000470176,9606.ENSP00000483185	PICALM,AP2M1,AP2S1,AP2B1
GO Process	GO:0048259	Regulation of receptor-mediated endocytosis	4	109	1.82	0.00057	9606.ENSP00000334836,9606.ENSP00000377015,9606.ENSP00000403362,9606.ENSP00000451300	BMP2K,PICALM,AP2M1,NUMB
GO Process	GO:1903077	Negative regulation of protein localization to plasma membrane	3	27	2.3	0.00082	9606.ENSP00000377015,9606.ENSP00000403362,9606.ENSP00000451300	PICALM,AP2M1,NUMB
GO Process	GO:0099003	Vesicle-mediated transport in synapse	4	134	1.73	0.00096	9606.ENSP00000377015,9606.ENSP00000403362,9606.ENSP00000470176,9606.ENSP00000483185	PICALM,AP2M1,AP2S1,AP2B1

GO Process	GO:0021849	Neuroblast division in subventricular zone	2	3	3.08	0.0028	9606.ENSP00000252891,9606.ENSP00000451300	NUMBL,NUMB
GO Process	GO:0048488	Synaptic vesicle endocytosis	3	56	1.98	0.0038	9606.ENSP00000377015,9606.ENSP00000403362,9606.ENSP00000483185	PICALM,AP2M1,AP2B1
GO Process	GO:0021670	Lateral ventricle development	2	12	2.47	0.0192	9606.ENSP00000252891,9606.ENSP00000451300	NUMBL,NUMB
GO Process	GO:0048268	Clathrin coat assembly	2	16	2.35	0.0278	9606.ENSP00000377015,9606.ENSP00000470176	PICALM,AP2S1
GO Process	GO:0097494	Regulation of vesicle size	2	19	2.28	0.0343	9606.ENSP00000377015,9606.ENSP00000403362	PICALM,AP2M1
GO Process	GO:2000369	Regulation of clathrin-dependent endocytosis	2	19	2.28	0.0343	9606.ENSP00000334836,9606.ENSP00000377015	BMP2K,PICALM
GO Function	GO:0030276	Clathrin binding	5	69	2.11	1.46e-06	9606.ENSP00000377015,9606.ENSP00000403362,9606.ENSP00000429824,9606.ENSP00000470176,9606.ENSP00000483185	PICALM,AP2M1,CLINT1,AP2S1,AP2B1
GO Component	GO:0005905	Clathrin-coated pit	6	71	2.18	2.71e-09	9606.ENSP00000377015,9606.ENSP00000392065,9606.ENSP00000403362,9606.ENSP00000451300,9606.ENSP00000470176,9606.ENSP00000483185	PICALM,REPS1,AP2M1,NUMB,AP2S1,AP2B1
GO Component	GO:0030118	Clathrin coat	5	44	2.31	3.60e-08	9606.ENSP00000377015,9606.ENSP00000403362,9606.ENSP00000429824,9606.ENSP00000470176,9606.ENSP00000483185	PICALM,AP2M1,CLINT1,AP2S1,AP2B1
GO	GO:00	Clathrin coat of	4	15	2.68	1.38e-07	9606.ENSP00000377015,9606.ENSP00000403362	PICALM,AP2M1,AP2S1,AP2B1

Component	30132	coated pit					,9606.ENSP00000470176,9606.ENSP00000483185	
GO Component	GO:0030136	Clathrin-coated vesicle	6	211	1.71	3.75e-07	9606.ENSP00000377015,9606.ENSP00000403362,9606.ENSP00000429824,9606.ENSP00000451300,9606.ENSP00000470176,9606.ENSP00000483185	PICALM,AP2M1,CLINT1,NUMB,AP2S1,AP2B1
GO Component	GO:0030125	Clathrin vesicle coat	4	30	2.38	7.06e-07	9606.ENSP00000403362,9606.ENSP00000429824,9606.ENSP00000470176,9606.ENSP00000483185	AP2M1,CLINT1,AP2S1,AP2B1
GO Component	GO:0030122	AP-2 adaptor complex	3	7	2.89	3.52e-06	9606.ENSP00000403362,9606.ENSP00000470176,9606.ENSP00000483185	AP2M1,AP2S1,AP2B1
GO Component	GO:0045334	Clathrin-coated endocytic vesicle	4	86	1.92	2.23e-05	9606.ENSP00000377015,9606.ENSP00000403362,9606.ENSP00000470176,9606.ENSP00000483185	PICALM,AP2M1,AP2S1,AP2B1
GO Component	GO:0036020	Endolysosome membrane	3	20	2.43	3.58e-05	9606.ENSP00000403362,9606.ENSP00000470176,9606.ENSP00000483185	AP2M1,AP2S1,AP2B1
GO Component	GO:0005768	Endosome	6	1030	1.02	0.00082	9606.ENSP00000377015,9606.ENSP00000403362,9606.ENSP00000429824,9606.ENSP00000451300,9606.ENSP00000470176,9606.ENSP00000483185	PICALM,AP2M1,CLINT1,NUMB,AP2S1,AP2B1
GO Component	GO:0009898	Cytoplasmic side of plasma membrane	3	174	1.49	0.0099	9606.ENSP00000403362,9606.ENSP00000470176,9606.ENSP00000483185	AP2M1,AP2S1,AP2B1

GO Component	GO:0010008	Endosome membrane	4	540	1.12	0.0133	9606.ENSP00000403362,9606.ENSP00000451300,9606.ENSP00000470176,9606.ENSP00000483185	AP2M1,NUMB,AP2S1,AP2B1
GO Component	GO:0030659	Cytoplasmic vesicle membrane	5	1190	0.88	0.0202	9606.ENSP00000403362,9606.ENSP00000429824,9606.ENSP00000451300,9606.ENSP00000470176,9606.ENSP00000483185	AP2M1,CLINT1,NUMB,AP2S1,AP2B1
GO Component	GO:0098794	Postsynapse	4	621	1.06	0.0202	9606.ENSP00000377015,9606.ENSP00000403362,9606.ENSP00000470176,9606.ENSP00000483185	PICALM,AP2M1,AP2S1,AP2B1
GO Component	GO:0005886	Plasma membrane	9	5544	0.46	0.0219	9606.ENSP00000372924,9606.ENSP00000377015,9606.ENSP00000392065,9606.ENSP00000403362,9606.ENSP00000410862,9606.ENSP00000429824,9606.ENSP00000451300,9606.ENSP00000470176,9606.ENSP00000483185	RALBP1,PICALM,REPS1,AP2M1,RUNDC3A,CLINT1,NUMB,AP2S1,AP2B1
GO Component	GO:0045202	Synapse	5	1350	0.82	0.0308	9606.ENSP00000377015,9606.ENSP00000403362,9606.ENSP00000451300,9606.ENSP00000470176,9606.ENSP00000483185	PICALM,AP2M1,NUMB,AP2S1,AP2B1
GO Component	GO:0098978	Glutamatergic synapse	3	334	1.21	0.0432	9606.ENSP00000403362,9606.ENSP00000451300,9606.ENSP00000483185	AP2M1,NUMB,AP2B1
STRING clusters	CL:13771	Clathrin coat, and Eps15 homology domain	7	37	2.53	2.51e-13	9606.ENSP00000334836,9606.ENSP00000377015,9606.ENSP00000392065,9606.ENSP00000403362,9606.ENSP00000429824,9606.ENSP00000470176,9606.ENSP00000483185	BMP2K,PICALM,REPS1,AP2M1,CLINT1,AP2S1,AP2B1
STRING clus	CL:13773	Clathrin vesicle coat, and Eps15 homology domain	4	30	2.38	1.84e-06	9606.ENSP00000392065,9606.ENSP00000403362,9606.ENSP00000470176,9606.ENSP00000483185	REPS1,AP2M1,AP2S1,AP2B1

ters								
STRING clusters	CL:13780	Nef Mediated CD8 Down-regulation	3	5	3.03	4.73e-06	9606.ENSP00000403362,9606.ENSP00000470176,9606.ENSP00000483185	AP2M1,AP2S1,AP2B1
STRING clusters	CL:21490	NUMB domain, and RNA-binding protein Musashi homologue, RNA recognition motif 2	2	5	2.86	0.0027	9606.ENSP00000252891,9606.ENSP00000451300	NUMBL,NUMB
Reactome	HSA-8856825	Cargo recognition for clathrin-mediated endocytosis	5	105	1.93	5.07e-06	9606.ENSP00000377015,9606.ENSP00000392065,9606.ENSP00000403362,9606.ENSP00000470176,9606.ENSP00000483185	PICALM,REPS1,AP2M1,AP2S1,AP2B1
Reactome	HSA-182218	Nef Mediated CD8 Down-regulation	3	6	2.95	1.21e-05	9606.ENSP00000403362,9606.ENSP00000470176,9606.ENSP00000483185	AP2M1,AP2S1,AP2B1
Reactome	HSA-437239	Recycling pathway of L1	4	48	2.17	1.21e-05	9606.ENSP00000403362,9606.ENSP00000451300,9606.ENSP00000470176,9606.ENSP00000483185	AP2M1,NUMB,AP2S1,AP2B1
Reactome	HSA-167590	Nef Mediated CD4 Down-regulation	3	9	2.78	1.30e-05	9606.ENSP00000403362,9606.ENSP00000470176,9606.ENSP00000483185	AP2M1,AP2S1,AP2B1
Reactome	HSA-5140745	WNT5A-dependent internalization of FZD2, FZD5 and ROR2	3	13	2.62	2.74e-05	9606.ENSP00000403362,9606.ENSP00000470176,9606.ENSP00000483185	AP2M1,AP2S1,AP2B1
Reactome	HSA-177504	Retrograde neurotrophin signalling	3	14	2.58	2.86e-05	9606.ENSP00000403362,9606.ENSP00000470176,9606.ENSP00000483185	AP2M1,AP2S1,AP2B1
Reactome	HSA-5099900	WNT5A-dependent internalization of FZD4	3	15	2.55	3.00e-05	9606.ENSP00000403362,9606.ENSP00000470176,9606.ENSP00000483185	AP2M1,AP2S1,AP2B1
Reactome	HSA-8866427	VLDLR internalisation and degradation	3	16	2.53	3.16e-05	9606.ENSP00000403362,9606.ENSP00000470176,9606.ENSP00000483185	AP2M1,AP2S1,AP2B1

Reactome	HSA-416993	Trafficking of GluR2-containing AMPA receptors	3	17	2.5	3.35e-05	9606.ENSP00000403362,9606.ENSP00000470176,9606.ENSP00000483185	AP2M1,AP2S1,AP2B1
Reactome	HSA-8964038	LDL clearance	3	19	2.45	4.11e-05	9606.ENSP00000403362,9606.ENSP00000470176,9606.ENSP00000483185	AP2M1,AP2S1,AP2B1
Reactome	HSA-199991	Membrane Trafficking	6	626	1.23	7.51e-05	9606.ENSP00000377015,9606.ENSP00000392065,9606.ENSP00000403362,9606.ENSP00000429824,9606.ENSP00000470176,9606.ENSP00000483185	PICALM,REPS1,AP2M1,CLINT1,AP2S1,AP2B1
Reactome	HSA-3928665	EPH-ephrin mediated repulsion of cells	3	51	2.02	0.00036	9606.ENSP00000403362,9606.ENSP00000470176,9606.ENSP00000483185	AP2M1,AP2S1,AP2B1
Reactome	HSA-9679191	Potential therapeutics for SARS	3	101	1.73	0.0022	9606.ENSP00000403362,9606.ENSP00000470176,9606.ENSP00000483185	AP2M1,AP2S1,AP2B1
Reactome	HSA-2132295	MHC class II antigen presentation	3	122	1.64	0.0035	9606.ENSP00000403362,9606.ENSP00000470176,9606.ENSP00000483185	AP2M1,AP2S1,AP2B1
Reactome	HSA-432722	Golgi Associated Vesicle Biogenesis	2	56	1.81	0.0300	9606.ENSP00000377015,9606.ENSP00000429824	PICALM,CLINT1

Table 3. Pathway Enrichment Analysis for GO, KEGG, STRING clusters and Reactome Terms within IKKA gene

#category	term ID	term description	observed gene count	background gene count	strength	false discovery rate	matching proteins in your network (IDs)	matching proteins in your network (labels)
GO Process	GO:0033209	Tumor necrosis factor-mediated signaling pathway	8	56	2.41	2.02e-14	9606.ENSP00000162749,9606.ENSP00000216797,9606.ENSP00000259808,9606.ENSP00000359424,9606.ENSP00000384273,9606.ENSP00000398698,9606.ENSP00000430684,9606.ENSP00000433623	TNFRSF1A,NFKBIA,RIPK1,CHUK,RELA,TNF,IKBKB,TRAF6
GO Process	GO:0071356	Cellular response to tumor necrosis factor	9	175	1.96	1.88e-13	9606.ENSP00000162749,9606.ENSP00000216797,9606.ENSP00000226574,9606.ENSP00000259808,9606.ENSP00000359424,9606.ENSP00000384273,9606.ENSP00000398698,9606.ENSP00000430684,9606.ENSP00000433623	TNFRSF1A,NFKBIA,NFKB1,RIPK1,CHUK,RELA,TNF,IKBKB,TRAF6
GO	GO:0043	Positive regulation of I-	9	191	1.93	2.69e-13	9606.ENSP00000162749,9606.ENSP00000259808,9606.E	TNFRSF1A,RIPK1,IRA

Process	123	kappaB kinase/NF-kappaB signaling					NSP00000358997,9606.ENSP00000359424,9606.ENSP00000384273,9606.ENSP00000398698,9606.ENSP00000430684,9606.ENSP00000433623,9606.ENSP00000483825	K1,CHUK,RELA,TNF,IKBKB,TRAF6,IKBKG
GO Process	GO:0007249	I-kappaB kinase/NF-kappaB signaling	7	68	2.27	7.38e-12	9606.ENSP00000216797,9606.ENSP00000359424,9606.ENSP00000384273,9606.ENSP00000398698,9606.ENSP00000430684,9606.ENSP00000433623,9606.ENSP00000483825	NFKBIA,CHUK,RELA,TNF,IKBKB,TRAF6,IKBKG
GO Process	GO:0051092	Positive regulation of NF-kappaB transcription factor activity	8	157	1.96	7.38e-12	9606.ENSP00000259808,9606.ENSP00000358997,9606.ENSP00000359424,9606.ENSP00000384273,9606.ENSP00000398698,9606.ENSP00000430684,9606.ENSP00000433623,9606.ENSP00000483825	RIPK1,IRAK1,CHUK,RELA,TNF,IKBKB,TRAF6,IKBKG
GO Process	GO:0019221	Cytokine-mediated signaling pathway	9	369	1.64	3.33e-11	9606.ENSP00000162749,9606.ENSP00000216797,9606.ENSP00000259808,9606.ENSP00000358997,9606.ENSP00000359424,9606.ENSP00000384273,9606.ENSP00000398698,9606.ENSP00000430684,9606.ENSP00000433623	TNFRSF1A,NFKBIA,RIPK1,IRAK1,CHUK,RELA,TNF,IKBKB,TRAF6
GO Process	GO:0071345	Cellular response to cytokine stimulus	10	711	1.4	7.49e-11	9606.ENSP00000162749,9606.ENSP00000216797,9606.ENSP00000226574,9606.ENSP00000259808,9606.ENSP00000358997,9606.ENSP00000359424,9606.ENSP00000384273,9606.ENSP00000398698,9606.ENSP00000430684,9606.ENSP00000433623	TNFRSF1A,NFKBIA,NFKB1,RIPK1,IRAK1,CHUK,RELA,TNF,IKBKB,TRAF6
GO Process	GO:0051090	Regulation of DNA-binding transcription factor activity	9	436	1.57	1.17e-10	9606.ENSP00000216797,9606.ENSP00000259808,9606.ENSP00000358997,9606.ENSP00000359424,9606.ENSP00000384273,9606.ENSP00000398698,9606.ENSP00000430684,9606.ENSP00000433623,9606.ENSP00000483825	NFKBIA,RIPK1,IRAK1,CHUK,RELA,TNF,IKBKB,TRAF6,IKBKG
GO Process	GO:0035556	Intracellular signal transduction	11	1518	1.11	7.14e-10	9606.ENSP00000162749,9606.ENSP00000216797,9606.ENSP00000226574,9606.ENSP00000259808,9606.ENSP00000358997,9606.ENSP00000359424,9606.ENSP00000384273,9606.ENSP00000398698,9606.ENSP00000430684,9606.ENSP00000433623,9606.ENSP00000483825	TNFRSF1A,NFKBIA,NFKB1,RIPK1,IRAK1,CHUK,RELA,TNF,IKBKB,TRAF6,IKBKG
GO Process	GO:0033554	Cellular response to stress	11	1572	1.1	9.73e-10	9606.ENSP00000162749,9606.ENSP00000216797,9606.ENSP00000226574,9606.ENSP00000259808,9606.ENSP00000358997,9606.ENSP00000359424,9606.ENSP00000384273,9606.ENSP00000398698,9606.ENSP00000430684,9606.ENSP00000433623,9606.ENSP00000483825	TNFRSF1A,NFKBIA,NFKB1,RIPK1,IRAK1,CHUK,RELA,TNF,IKBKB,TRAF6,IKBKG
GO Process	GO:0045944	Positive regulation of transcription by RNA polymerase II	10	1250	1.16	1.19e-08	9606.ENSP00000162749,9606.ENSP00000216797,9606.ENSP00000226574,9606.ENSP00000259808,9606.ENSP00000359424,9606.ENSP00000384273,9606.ENSP00000398698,9606.ENSP00000430684,9606.ENSP00000433623,9606.ENSP00000483825	TNFRSF1A,NFKBIA,NFKB1,RIPK1,CHUK,RELA,TNF,IKBKB,TRAF6,IKBKG

GO Process	GO:0051707	Response to other organism	10	1328	1.13	2.04e-08	9606.ENSP00000162749,9606.ENSP00000216797,9606.ENSP00000226574,9606.ENSP00000358997,9606.ENSP00000359424,9606.ENSP00000384273,9606.ENSP00000398698,9606.ENSP00000430684,9606.ENSP00000433623,9606.ENSP00000483825	TNFRSF1A,NFKBIA,NFKB1,IRAK1,CHUK,RELA,TNF,IKBKB,TRAF6,IKBKG
GO Process	GO:0048584	Positive regulation of response to stimulus	11	2131	0.97	2.13e-08	9606.ENSP00000162749,9606.ENSP00000216797,9606.ENSP00000226574,9606.ENSP00000259808,9606.ENSP00000358997,9606.ENSP00000359424,9606.ENSP00000384273,9606.ENSP00000398698,9606.ENSP00000430684,9606.ENSP00000433623,9606.ENSP00000483825	TNFRSF1A,NFKBIA,NFKB1,RIPK1,IRAK1,CHUK,RELA,TNF,IKBKB,TRAF6,IKBKG
GO Process	GO:0006954	Inflammatory response	8	538	1.43	3.96e-08	9606.ENSP00000162749,9606.ENSP00000226574,9606.ENSP00000259808,9606.ENSP00000359424,9606.ENSP00000384273,9606.ENSP00000398698,9606.ENSP00000430684,9606.ENSP00000483825	TNFRSF1A,NFKB1,RIPK1,CHUK,RELA,TNF,IKBKB,IKBKG
GO Process	GO:0002764	Immune response-regulating signaling pathway	7	312	1.6	5.82e-08	9606.ENSP00000216797,9606.ENSP00000358997,9606.ENSP00000384273,9606.ENSP00000398698,9606.ENSP00000430684,9606.ENSP00000433623,9606.ENSP00000483825	NFKBIA,IRAK1,RELA,TNF,IKBKB,TRAF6,IKBKG
GO Process	GO:0009967	Positive regulation of signal transduction	10	1525	1.07	5.82e-08	9606.ENSP00000162749,9606.ENSP00000226574,9606.ENSP00000259808,9606.ENSP00000358997,9606.ENSP00000359424,9606.ENSP00000384273,9606.ENSP00000398698,9606.ENSP00000430684,9606.ENSP00000433623,9606.ENSP00000483825	TNFRSF1A,NFKB1,RIPK1,IRAK1,CHUK,RELA,TNF,IKBKB,TRAF6,IKBKG
GO Process	GO:0031347	Regulation of defense response	8	638	1.35	1.21e-07	9606.ENSP00000162749,9606.ENSP00000216797,9606.ENSP00000226574,9606.ENSP00000259808,9606.ENSP00000384273,9606.ENSP00000398698,9606.ENSP00000430684,9606.ENSP00000433623	TNFRSF1A,NFKBIA,NFKB1,RIPK1,RELA,TNF,IKBKB,TRAF6
GO Process	GO:1902531	Regulation of intracellular signal transduction	10	1726	1.02	1.42e-07	9606.ENSP00000162749,9606.ENSP00000216797,9606.ENSP00000259808,9606.ENSP00000358997,9606.ENSP00000359424,9606.ENSP00000384273,9606.ENSP00000398698,9606.ENSP00000430684,9606.ENSP00000433623,9606.ENSP00000483825	TNFRSF1A,NFKBIA,RIPK1,IRAK1,CHUK,RELA,TNF,IKBKB,TRAF6,IKBKG
GO Process	GO:0071222	Cellular response to lipopolysaccharide	6	195	1.74	2.27e-07	9606.ENSP00000216797,9606.ENSP00000226574,9606.ENSP00000358997,9606.ENSP00000384273,9606.ENSP00000398698,9606.ENSP00000433623	NFKBIA,NFKB1,IRAK1,RELA,TNF,TRAF6
GO Process	GO:0070498	interleukin-1-mediated signaling pathway	4	20	2.55	2.56e-07	9606.ENSP00000358997,9606.ENSP00000384273,9606.ENSP00000430684,9606.ENSP00000433623	IRAK1,RELA,IKBKB,TRAF6
GO	GO:1904	Positive regulation of	4	23	2.49	3.88e-07	9606.ENSP00000358997,9606.ENSP00000384273,9606.E	IRAK1,RELA,TNF,TR

Process	996	leukocyte adhesion to vascular endothelial cell					NSP00000398698,9606.ENSP00000433623	AF6
GO Process	GO:0009966	Regulation of signal transduction	11	2978	0.82	3.96e-07	9606.ENSP00000162749,9606.ENSP00000216797,9606.ENSP00000226574,9606.ENSP00000259808,9606.ENSP00000358997,9606.ENSP00000359424,9606.ENSP00000384273,9606.ENSP00000398698,9606.ENSP00000430684,9606.ENSP00000433623,9606.ENSP00000483825	TNFRSF1A,NFKBIA,NFKB1,RIPK1,IRAK1,CHUK,RELA,TNF,IKBKB,TRAF6,IKBKG
GO Process	GO:0007166	Cell surface receptor signaling pathway	10	2040	0.94	5.54e-07	9606.ENSP00000162749,9606.ENSP00000216797,9606.ENSP00000259808,9606.ENSP00000358997,9606.ENSP00000359424,9606.ENSP00000384273,9606.ENSP00000398698,9606.ENSP00000430684,9606.ENSP00000433623,9606.ENSP00000483825	TNFRSF1A,NFKBIA,RIPK1,IRAK1,CHUK,RELA,TNF,IKBKB,TRAF6,IKBKG
GO Process	GO:0031325	Positive regulation of cellular metabolic process	11	3114	0.8	5.70e-07	9606.ENSP00000162749,9606.ENSP00000216797,9606.ENSP00000226574,9606.ENSP00000259808,9606.ENSP00000358997,9606.ENSP00000359424,9606.ENSP00000384273,9606.ENSP00000398698,9606.ENSP00000430684,9606.ENSP00000433623,9606.ENSP00000483825	TNFRSF1A,NFKBIA,NFKB1,RIPK1,IRAK1,CHUK,RELA,TNF,IKBKB,TRAF6,IKBKG
GO Process	GO:0071347	Cellular response to interleukin-1	5	98	1.96	5.70e-07	9606.ENSP00000226574,9606.ENSP00000358997,9606.ENSP00000384273,9606.ENSP00000430684,9606.ENSP00000433623	NFKB1,IRAK1,RELA,IKBKB,TRAF6
GO Process	GO:0002221	Pattern recognition receptor signaling pathway	5	101	1.95	6.15e-07	9606.ENSP00000216797,9606.ENSP00000358997,9606.ENSP00000384273,9606.ENSP00000398698,9606.ENSP00000433623	NFKBIA,IRAK1,RELA,TNF,TRAF6
GO Process	GO:0051173	Positive regulation of nitrogen compound metabolic process	11	3166	0.79	6.15e-07	9606.ENSP00000162749,9606.ENSP00000216797,9606.ENSP00000226574,9606.ENSP00000259808,9606.ENSP00000358997,9606.ENSP00000359424,9606.ENSP00000384273,9606.ENSP00000398698,9606.ENSP00000430684,9606.ENSP00000433623,9606.ENSP00000483825	TNFRSF1A,NFKBIA,NFKB1,RIPK1,IRAK1,CHUK,RELA,TNF,IKBKB,TRAF6,IKBKG
GO Process	GO:1901222	Regulation of NIK/NF-kappaB signaling	5	102	1.94	6.18e-07	9606.ENSP00000216797,9606.ENSP00000358997,9606.ENSP00000384273,9606.ENSP00000398698,9606.ENSP00000433623	NFKBIA,IRAK1,RELA,TNF,TRAF6
GO Process	GO:0006952	Defense response	9	1394	1.06	6.93e-07	9606.ENSP00000162749,9606.ENSP00000226574,9606.ENSP00000259808,9606.ENSP00000358997,9606.ENSP00000359424,9606.ENSP00000384273,9606.ENSP00000398698,9606.ENSP00000430684,9606.ENSP00000483825	TNFRSF1A,NFKB1,RIPK1,IRAK1,CHUK,RELA,TNF,IKBKB,IKBKG
GO Process	GO:0038061	NIK/NF-kappaB signaling	4	36	2.3	1.35e-06	9606.ENSP00000358997,9606.ENSP00000359424,9606.ENSP00000384273,9606.ENSP00000433623	IRAK1,CHUK,RELA,TRAF6
GO	GO:0006	Regulation of	11	3460	0.76	1.38e-06	9606.ENSP00000162749,9606.ENSP00000216797,9606.E	TNFRSF1A,NFKBIA,N

Process	355	transcription, DNA-templated					NSP00000226574,9606.ENSP00000259808,9606.ENSP00000358997,9606.ENSP00000359424,9606.ENSP00000384273,9606.ENSP00000398698,9606.ENSP00000430684,9606.ENSP00000433623,9606.ENSP00000483825	FKB1,RIPK1,IRAK1,CHUK,RELA,TNF,IKBKB,TRAF6,IKBKG
GO Process	GO:0031349	Positive regulation of defense response	6	296	1.56	1.38e-06	9606.ENSP00000162749,9606.ENSP00000216797,9606.ENSP00000259808,9606.ENSP00000398698,9606.ENSP00000430684,9606.ENSP00000433623	TNFRSF1A,NFKBIA,RIPK1,TNF,IKBKB,TRAF6
GO Process	GO:0032101	Regulation of response to external stimulus	8	964	1.17	1.38e-06	9606.ENSP00000162749,9606.ENSP00000216797,9606.ENSP00000226574,9606.ENSP00000259808,9606.ENSP00000384273,9606.ENSP00000398698,9606.ENSP00000430684,9606.ENSP00000433623	TNFRSF1A,NFKBIA,NFKB1,RIPK1,RELA,TNF,IKBKB,TRAF6
GO Process	GO:0010604	Positive regulation of macromolecule metabolic process	11	3533	0.75	1.56e-06	9606.ENSP00000162749,9606.ENSP00000216797,9606.ENSP00000226574,9606.ENSP00000259808,9606.ENSP00000358997,9606.ENSP00000359424,9606.ENSP00000384273,9606.ENSP00000398698,9606.ENSP00000430684,9606.ENSP00000433623,9606.ENSP00000483825	TNFRSF1A,NFKBIA,NFKB1,RIPK1,IRAK1,CHUK,RELA,TNF,IKBKB,TRAF6,IKBKG
GO Process	GO:1901701	Cellular response to oxygen-containing compound	8	1057	1.13	2.44e-06	9606.ENSP00000216797,9606.ENSP00000226574,9606.ENSP00000259808,9606.ENSP00000358997,9606.ENSP00000359424,9606.ENSP00000384273,9606.ENSP00000398698,9606.ENSP00000433623	NFKBIA,NFKB1,RIPK1,IRAK1,CHUK,RELA,TNF,TRAF6
GO Process	GO:0009617	Response to bacterium	7	663	1.28	3.44e-06	9606.ENSP00000162749,9606.ENSP00000216797,9606.ENSP00000226574,9606.ENSP00000358997,9606.ENSP00000384273,9606.ENSP00000398698,9606.ENSP00000433623	TNFRSF1A,NFKBIA,NFKB1,IRAK1,RELA,TNF,TRAF6
GO Process	GO:0071316	Cellular response to nicotine	3	7	2.89	3.47e-06	9606.ENSP00000226574,9606.ENSP00000384273,9606.ENSP00000398698	NFKB1,RELA,TNF
GO Process	GO:0009615	Response to virus	6	356	1.48	3.48e-06	9606.ENSP00000226574,9606.ENSP00000359424,9606.ENSP00000384273,9606.ENSP00000398698,9606.ENSP00000430684,9606.ENSP00000483825	NFKB1,CHUK,RELA,TNF,IKBKB,IKBKG
GO Process	GO:0050727	Regulation of inflammatory response	6	371	1.46	4.25e-06	9606.ENSP00000162749,9606.ENSP00000216797,9606.ENSP00000226574,9606.ENSP00000259808,9606.ENSP00000384273,9606.ENSP00000398698	TNFRSF1A,NFKBIA,NFKB1,RIPK1,RELA,TNF
GO Process	GO:0051239	Regulation of multicellular organismal process	10	2749	0.81	5.50e-06	9606.ENSP00000162749,9606.ENSP00000216797,9606.ENSP00000226574,9606.ENSP00000259808,9606.ENSP00000358997,9606.ENSP00000359424,9606.ENSP00000384273,9606.ENSP00000398698,9606.ENSP00000430684,9606.ENSP00000433623	TNFRSF1A,NFKBIA,NFKB1,RIPK1,IRAK1,CHUK,RELA,TNF,IKBKB,TRAF6
GO	GO:0001	Regulation of cytokine	7	739	1.23	6.13e-06	9606.ENSP00000226574,9606.ENSP00000259808,9606.E	NFKB1,RIPK1,IRAK1,

Process	817	production					NSP00000358997,9606.ENSP00000359424,9606.ENSP00000384273,9606.ENSP00000398698,9606.ENSP00000433623	CHUK,RELA,TNF,TRAF6
GO Process	GO:0002224	Toll-like receptor signaling pathway	4	61	2.07	6.76e-06	9606.ENSP00000216797,9606.ENSP00000358997,9606.ENSP00000398698,9606.ENSP00000433623	NFKBIA,IRAK1,TNF,TRAF6
GO Process	GO:1901224	Positive regulation of NIK/NF-kappaB signaling	4	67	2.03	9.34e-06	9606.ENSP00000358997,9606.ENSP00000384273,9606.ENSP00000398698,9606.ENSP00000433623	IRAK1,RELA,TNF,TRAF6
GO Process	GO:0032103	Positive regulation of response to external stimulus	6	453	1.38	1.19e-05	9606.ENSP00000162749,9606.ENSP00000216797,9606.ENSP00000259808,9606.ENSP00000398698,9606.ENSP00000430684,9606.ENSP00000433623	TNFRSF1A,NFKBIA,RIPK1,TNF,IKBKB,TRAF6
GO Process	GO:0009612	Response to mechanical stimulus	5	214	1.62	1.29e-05	9606.ENSP00000162749,9606.ENSP00000216797,9606.ENSP00000226574,9606.ENSP00000384273,9606.ENSP00000398698	TNFRSF1A,NFKBIA,NFKB1,RELA,TNF
GO Process	GO:0000165	MAPK cascade	5	219	1.61	1.39e-05	9606.ENSP00000226574,9606.ENSP00000259808,9606.ENSP00000358997,9606.ENSP00000398698,9606.ENSP00000430684	NFKB1,RIPK1,IRAK1,TNF,IKBKB
GO Process	GO:0051403	Stress-activated MAPK cascade	4	75	1.98	1.39e-05	9606.ENSP00000226574,9606.ENSP00000358997,9606.ENSP00000398698,9606.ENSP00000430684	NFKB1,IRAK1,TNF,IKBKB
GO Process	GO:0065009	Regulation of molecular function	10	3085	0.76	1.46e-05	9606.ENSP00000216797,9606.ENSP00000226574,9606.ENSP00000259808,9606.ENSP00000358997,9606.ENSP00000359424,9606.ENSP00000384273,9606.ENSP00000398698,9606.ENSP00000430684,9606.ENSP00000433623,9606.ENSP00000483825	NFKBIA,NFKB1,RIPK1,IRAK1,CHUK,RELA,TNF,IKBKB,TRAF6,IKBKG
GO Process	GO:0001819	Positive regulation of cytokine production	6	482	1.35	1.58e-05	9606.ENSP00000259808,9606.ENSP00000358997,9606.ENSP00000359424,9606.ENSP00000384273,9606.ENSP00000398698,9606.ENSP00000433623	RIPK1,IRAK1,CHUK,RELA,TNF,TRAF6
GO Process	GO:0002682	Regulation of immune system process	8	1438	1.0	1.86e-05	9606.ENSP00000216797,9606.ENSP00000259808,9606.ENSP00000358997,9606.ENSP00000384273,9606.ENSP00000398698,9606.ENSP00000430684,9606.ENSP00000433623,9606.ENSP00000483825	NFKBIA,RIPK1,IRAK1,RELA,TNF,IKBKB,TRAF6,IKBKG
GO Process	GO:1903140	Regulation of establishment of endothelial barrier	3	16	2.53	2.04e-05	9606.ENSP00000162749,9606.ENSP00000398698,9606.ENSP00000430684	TNFRSF1A,TNF,IKBKB
GO Process	GO:0098542	Defense response to other organism	7	989	1.1	3.57e-05	9606.ENSP00000162749,9606.ENSP00000358997,9606.ENSP00000359424,9606.ENSP00000384273,9606.ENSP00000398698,9606.ENSP00000430684,9606.ENSP000004838	TNFRSF1A,IRAK1,CHUK,RELA,TNF,IKBKB,IKBKG

							25	
GO Process	GO:0035994	Response to muscle stretch	3	26	2.32	7.00e-05	9606.ENSP00000216797,9606.ENSP00000226574,9606.ENSP00000384273	NFKBIA,NFKB1,RELA
GO Process	GO:0051246	Regulation of protein metabolic process	9	2622	0.79	8.38e-05	9606.ENSP00000162749,9606.ENSP00000216797,9606.ENSP00000226574,9606.ENSP00000259808,9606.ENSP00000358997,9606.ENSP00000384273,9606.ENSP00000398698,9606.ENSP00000430684,9606.ENSP00000433623	TNFRSF1A,NFKBIA,NFKB1,RIPK1,IRAK1,RELA,TNF,IKBKB,TRAF6
GO Process	GO:0002753	Cytoplasmic pattern recognition receptor signaling pathway	3	28	2.28	8.45e-05	9606.ENSP00000216797,9606.ENSP00000384273,9606.ENSP00000433623	NFKBIA,RELA,TRAF6
GO Process	GO:0050729	Positive regulation of inflammatory response	4	145	1.69	0.00014	9606.ENSP00000162749,9606.ENSP00000216797,9606.ENSP00000259808,9606.ENSP00000398698	TNFRSF1A,NFKBIA,RIPK1,TNF
GO Process	GO:0001959	Regulation of cytokine-mediated signaling pathway	4	152	1.67	0.00017	9606.ENSP00000162749,9606.ENSP00000259808,9606.ENSP00000358997,9606.ENSP00000430684	TNFRSF1A,RIPK1,IRAK1,IKBKB
GO Process	GO:0045087	Innate immune response	6	754	1.15	0.00017	9606.ENSP00000358997,9606.ENSP00000359424,9606.ENSP00000384273,9606.ENSP00000398698,9606.ENSP00000430684,9606.ENSP00000483825	IRAK1,CHUK,RELA,TNF,IKBKB,IKBKG
GO Process	GO:0031663	Lipopolysaccharide-mediated signaling pathway	3	39	2.14	0.00020	9606.ENSP00000216797,9606.ENSP00000358997,9606.ENSP00000398698	NFKBIA,IRAK1,TNF
GO Process	GO:0006955	Immune response	7	1321	0.98	0.00021	9606.ENSP00000358997,9606.ENSP00000359424,9606.ENSP00000384273,9606.ENSP00000398698,9606.ENSP00000430684,9606.ENSP00000433623,9606.ENSP00000483825	IRAK1,CHUK,RELA,TNF,IKBKB,TRAF6,IKBKG
GO Process	GO:1905953	Negative regulation of lipid localization	3	47	2.06	0.00032	9606.ENSP00000216797,9606.ENSP00000226574,9606.ENSP00000398698	NFKBIA,NFKB1,TNF
GO Process	GO:0002684	Positive regulation of immune system process	6	874	1.09	0.00038	9606.ENSP00000259808,9606.ENSP00000384273,9606.ENSP00000398698,9606.ENSP00000430684,9606.ENSP00000433623,9606.ENSP00000483825	RIPK1,RELA,TNF,IKBKB,TRAF6,IKBKG
GO Process	GO:0010883	Regulation of lipid storage	3	50	2.03	0.00038	9606.ENSP00000216797,9606.ENSP00000226574,9606.ENSP00000398698	NFKBIA,NFKB1,TNF
GO Process	GO:0010803	Regulation of tumor necrosis factor-mediated signaling pathway	3	54	2.0	0.00046	9606.ENSP00000162749,9606.ENSP00000259808,9606.ENSP00000430684	TNFRSF1A,RIPK1,IKBKB

GO Process	GO:2000630	Positive regulation of miRNA metabolic process	3	54	2.0	0.00046	9606.ENSP00000226574,9606.ENSP00000384273,9606.ENSP00000398698	NFKB1,RELA,TNF
GO Process	GO:0051247	Positive regulation of protein metabolic process	7	1512	0.92	0.00048	9606.ENSP00000162749,9606.ENSP00000216797,9606.ENSP00000259808,9606.ENSP00000358997,9606.ENSP00000384273,9606.ENSP00000398698,9606.ENSP00000433623	TNFRSF1A,NFKBIA,RIPK1,IRAK1,RELA,TNF,TRAF6
GO Process	GO:1903347	Negative regulation of bicellular tight junction assembly	2	4	2.95	0.00051	9606.ENSP00000398698,9606.ENSP00000430684	TNF,IKBKB
GO Process	GO:0007254	JNK cascade	3	57	1.97	0.00052	9606.ENSP00000226574,9606.ENSP00000358997,9606.ENSP00000398698	NFKB1,IRAK1,TNF
GO Process	GO:0002763	Positive regulation of myeloid leukocyte differentiation	3	58	1.97	0.00053	9606.ENSP00000259808,9606.ENSP00000398698,9606.ENSP00000433623	RIPK1,TNF,TRAF6
GO Process	GO:0045637	Regulation of myeloid cell differentiation	4	214	1.52	0.00053	9606.ENSP00000216797,9606.ENSP00000259808,9606.ENSP00000398698,9606.ENSP00000433623	NFKBIA,RIPK1,TNF,TRAF6
GO Process	GO:0050778	Positive regulation of immune response	5	502	1.25	0.00053	9606.ENSP00000384273,9606.ENSP00000398698,9606.ENSP00000430684,9606.ENSP00000433623,9606.ENSP00000483825	RELA,TNF,IKBKB,TRAF6,IKBKG
GO Process	GO:0031399	Regulation of protein modification process	7	1560	0.91	0.00057	9606.ENSP00000162749,9606.ENSP00000259808,9606.ENSP00000358997,9606.ENSP00000384273,9606.ENSP00000398698,9606.ENSP00000430684,9606.ENSP00000433623	TNFRSF1A,RIPK1,IRAK1,RELA,TNF,IKBKB,TRAF6
GO Process	GO:0045595	Regulation of cell differentiation	7	1582	0.9	0.00062	9606.ENSP00000162749,9606.ENSP00000216797,9606.ENSP00000226574,9606.ENSP00000259808,9606.ENSP00000398698,9606.ENSP00000430684,9606.ENSP00000433623	TNFRSF1A,NFKBIA,NFKB1,RIPK1,TNF,IKBKB,TRAF6
GO Process	GO:0035509	Negative regulation of myosin-light-chain-phosphatase activity	2	5	2.86	0.00067	9606.ENSP00000398698,9606.ENSP00000430684	TNF,IKBKB
GO Process	GO:0032655	Regulation of interleukin-12 production	3	64	1.92	0.00069	9606.ENSP00000226574,9606.ENSP00000384273,9606.ENSP00000433623	NFKB1,RELA,TRAF6
GO Process	GO:0032757	Positive regulation of interleukin-8 production	3	65	1.92	0.00071	9606.ENSP00000259808,9606.ENSP00000384273,9606.ENSP00000398698	RIPK1,RELA,TNF

GO Process	GO:0010941	Regulation of cell death	7	1651	0.88	0.00080	9606.ENSP00000162749,9606.ENSP00000226574,9606.ENSP00000259808,9606.ENSP00000384273,9606.ENSP00000398698,9606.ENSP00000433623,9606.ENSP00000483825	TNFRSF1A,NFKB1,RIPK1,RELA,TNF,TRAF6,IKBKG
GO Process	GO:0060558	Regulation of calcidiol 1-monooxygenase activity	2	6	2.78	0.00087	9606.ENSP00000226574,9606.ENSP00000398698	NFKB1,TNF
GO Process	GO:0006915	Apoptotic process	6	1041	1.01	0.00089	9606.ENSP00000162749,9606.ENSP00000216797,9606.ENSP00000226574,9606.ENSP00000259808,9606.ENSP00000398698,9606.ENSP00000483825	TNFRSF1A,NFKBIA,NFKB1,RIPK1,TNF,IKBKG
GO Process	GO:0060556	Regulation of vitamin D biosynthetic process	2	7	2.71	0.0011	9606.ENSP00000226574,9606.ENSP00000398698	NFKB1,TNF
GO Process	GO:0009628	Response to abiotic stimulus	6	1107	0.99	0.0012	9606.ENSP00000162749,9606.ENSP00000216797,9606.ENSP00000226574,9606.ENSP00000358997,9606.ENSP00000384273,9606.ENSP00000398698	TNFRSF1A,NFKBIA,NFKB1,IRAK1,RELA,TNF
GO Process	GO:0097527	Necroptotic signaling pathway	2	8	2.65	0.0013	9606.ENSP00000259808,9606.ENSP00000398698	RIPK1,TNF
GO Process	GO:0044092	Negative regulation of molecular function	6	1143	0.97	0.0014	9606.ENSP00000216797,9606.ENSP00000226574,9606.ENSP00000358997,9606.ENSP00000359424,9606.ENSP00000398698,9606.ENSP00000430684	NFKBIA,NFKB1,IRAK1,CHUK,TNF,IKBKB
GO Process	GO:0032088	Negative regulation of NF-kappaB transcription factor activity	3	90	1.78	0.0016	9606.ENSP00000216797,9606.ENSP00000358997,9606.ENSP00000359424	NFKBIA,IRAK1,CHUK
GO Process	GO:0070431	Nucleotide-binding oligomerization domain containing 2 signaling pathway	2	9	2.6	0.0016	9606.ENSP00000216797,9606.ENSP00000384273	NFKBIA,RELA
GO Process	GO:0046330	Positive regulation of JNK cascade	3	96	1.75	0.0020	9606.ENSP00000259808,9606.ENSP00000398698,9606.ENSP00000433623	RIPK1,TNF,TRAF6
GO Process	GO:2001237	Negative regulation of extrinsic apoptotic signaling pathway	3	97	1.74	0.0020	9606.ENSP00000259808,9606.ENSP00000384273,9606.ENSP00000398698	RIPK1,RELA,TNF
GO Process	GO:0042325	Regulation of phosphorylation	6	1251	0.93	0.0023	9606.ENSP00000162749,9606.ENSP00000259808,9606.ENSP00000358997,9606.ENSP00000398698,9606.ENSP00000430684,9606.ENSP00000433623	TNFRSF1A,RIPK1,IRAK1,TNF,IKBKB,TRAF6
GO	GO:0050	T cell receptor signaling	3	103	1.72	0.0023	9606.ENSP00000430684,9606.ENSP00000433623,9606.E	IKBKB,TRAF6,IKBKG

Process	852	pathway					NSP00000483825	
GO Process	GO:0001934	Positive regulation of protein phosphorylation	5	747	1.08	0.0029	9606.ENSP00000162749,9606.ENSP00000259808,9606.ENSP00000358997,9606.ENSP00000398698,9606.ENSP00000433623	TNFRSF1A,RIPK1,IRAK1,TNF,TRAF6
GO Process	GO:0097191	Extrinsic apoptotic signaling pathway	3	113	1.68	0.0030	9606.ENSP00000162749,9606.ENSP00000259808,9606.ENSP00000398698	TNFRSF1A,RIPK1,TNF
GO Process	GO:0043406	Positive regulation of MAP kinase activity	3	116	1.67	0.0032	9606.ENSP00000358997,9606.ENSP00000398698,9606.ENSP00000433623	IRAK1,TNF,TRAF6
GO Process	GO:0034614	Cellular response to reactive oxygen species	3	118	1.66	0.0033	9606.ENSP00000259808,9606.ENSP00000359424,9606.ENSP00000384273	RIPK1,CHUK,RELA
GO Process	GO:0050862	Positive regulation of T cell receptor signaling pathway	2	16	2.35	0.0038	9606.ENSP00000384273,9606.ENSP00000483825	RELA,IKBKG
GO Process	GO:2000026	Regulation of multicellular organismal development	6	1389	0.89	0.0038	9606.ENSP00000162749,9606.ENSP00000216797,9606.ENSP00000259808,9606.ENSP00000398698,9606.ENSP00000430684,9606.ENSP00000433623	TNFRSF1A,NFKBIA,RIPK1,TNF,IKBKB,TRAF6
GO Process	GO:0007250	Activation of NF-kappaB-inducing kinase activity	2	18	2.3	0.0046	9606.ENSP00000358997,9606.ENSP00000433623	IRAK1,TRAF6
GO Process	GO:0042981	Regulation of apoptotic process	6	1462	0.87	0.0048	9606.ENSP00000162749,9606.ENSP00000226574,9606.ENSP00000259808,9606.ENSP00000384273,9606.ENSP00000398698,9606.ENSP00000433623	TNFRSF1A,NFKB1,RIPK1,RELA,TNF,TRAF6
GO Process	GO:0034142	Toll-like receptor 4 signaling pathway	2	19	2.28	0.0050	9606.ENSP00000216797,9606.ENSP00000358997	NFKBIA,IRAK1
GO Process	GO:0032495	Response to muramyl dipeptide	2	20	2.25	0.0054	9606.ENSP00000216797,9606.ENSP00000384273	NFKBIA,RELA
GO Process	GO:0002755	MyD88-dependent toll-like receptor signaling pathway	2	21	2.23	0.0057	9606.ENSP00000358997,9606.ENSP00000433623	IRAK1,TRAF6
GO Process	GO:0010888	Negative regulation of lipid storage	2	21	2.23	0.0057	9606.ENSP00000216797,9606.ENSP00000398698	NFKBIA,TNF
GO	GO:1901	Response to peptide	4	437	1.21	0.0057	9606.ENSP00000216797,9606.ENSP00000226574,9606.E	NFKBIA,NFKB1,REL

Process	652						NSP00000384273,9606.ENSP00000398698	A,TNF
GO Process	GO:1902004	Positive regulation of amyloid-beta formation	2	21	2.23	0.0057	9606.ENSP00000384273,9606.ENSP00000398698	RELA,TNF
GO Process	GO:0002223	Stimulatory C-type lectin receptor signaling pathway	2	22	2.21	0.0061	9606.ENSP00000430684,9606.ENSP00000433623	IKBKB,TRAF6
GO Process	GO:0038095	Fc-epsilon receptor signaling pathway	2	23	2.19	0.0064	9606.ENSP00000430684,9606.ENSP00000433623	IKBKB,TRAF6
GO Process	GO:1903209	Positive regulation of oxidative stress-induced cell death	2	23	2.19	0.0064	9606.ENSP00000259808,9606.ENSP00000398698	RIPK1,TNF
GO Process	GO:0048523	Negative regulation of cellular process	9	4736	0.53	0.0069	9606.ENSP00000162749,9606.ENSP00000216797,9606.ENSP00000226574,9606.ENSP00000259808,9606.ENSP00000384273,9606.ENSP00000398698,9606.ENSP00000430684,9606.ENSP00000433623,9606.ENSP00000483825	TNFRSF1A,NFKBIA,NFKB1,RIPK1,RELA,TNF,IKBKB,TRAF6,IKBKG
GO Process	GO:1904385	Cellular response to angiotensin	2	24	2.17	0.0069	9606.ENSP00000226574,9606.ENSP00000384273	NFKB1,RELA
GO Process	GO:0048585	Negative regulation of response to stimulus	6	1612	0.82	0.0073	9606.ENSP00000162749,9606.ENSP00000216797,9606.ENSP00000226574,9606.ENSP00000259808,9606.ENSP00000384273,9606.ENSP00000398698	TNFRSF1A,NFKBIA,NFKB1,RIPK1,RELA,TNF
GO Process	GO:0016310	Phosphorylation	5	966	0.97	0.0075	9606.ENSP00000259808,9606.ENSP00000358997,9606.ENSP00000359424,9606.ENSP00000430684,9606.ENSP00000483825	RIPK1,IRAK1,CHUK,IKBKB,IKBKG
GO Process	GO:0043410	Positive regulation of MAPK cascade	4	481	1.17	0.0075	9606.ENSP00000259808,9606.ENSP00000358997,9606.ENSP00000398698,9606.ENSP00000433623	RIPK1,IRAK1,TNF,TRAF6
GO Process	GO:0045672	Positive regulation of osteoclast differentiation	2	26	2.14	0.0076	9606.ENSP00000398698,9606.ENSP00000433623	TNF,TRAF6
GO Process	GO:1903798	Regulation of miRNA maturation	2	26	2.14	0.0076	9606.ENSP00000259808,9606.ENSP00000398698	RIPK1,TNF
GO Process	GO:1903959	Regulation of anion transmembrane transport	2	28	2.11	0.0085	9606.ENSP00000259808,9606.ENSP00000398698	RIPK1,TNF

GO Process	GO:0060548	Negative regulation of cell death	5	1016	0.95	0.0089	9606.ENSP00000226574,9606.ENSP00000259808,9606.ENSP00000384273,9606.ENSP00000398698,9606.ENSP00000483825	NFKB1,RIPK1,RELA,TNF,IKBKG
GO Process	GO:0071354	Cellular response to interleukin-6	2	29	2.09	0.0089	9606.ENSP00000226574,9606.ENSP00000384273	NFKB1,RELA
GO Process	GO:0010743	Regulation of macrophage derived foam cell differentiation	2	31	2.06	0.0098	9606.ENSP00000216797,9606.ENSP00000226574	NFKBIA,NFKB1
GO Process	GO:0018105	Peptidyl-serine phosphorylation	3	193	1.44	0.0099	9606.ENSP00000259808,9606.ENSP00000359424,9606.ENSP00000430684	RIPK1,CHUK,IKBKB
GO Process	GO:0072659	Protein localization to plasma membrane	3	200	1.43	0.0107	9606.ENSP00000162749,9606.ENSP00000398698,9606.ENSP00000430684	TNFRSF1A,TNF,IKBKB
GO Process	GO:2001240	Negative regulation of extrinsic apoptotic signaling pathway in absence of ligand	2	36	2.0	0.0125	9606.ENSP00000259808,9606.ENSP00000398698	RIPK1,TNF
GO Process	GO:0002478	Antigen processing and presentation of exogenous peptide antigen	2	39	1.96	0.0142	9606.ENSP00000430684,9606.ENSP00000433623	IKBKB,TRAF6
GO Process	GO:0030866	Cortical actin cytoskeleton organization	2	40	1.95	0.0147	9606.ENSP00000398698,9606.ENSP00000430684	TNF,IKBKB
GO Process	GO:0043507	Positive regulation of JUN kinase activity	2	42	1.93	0.0160	9606.ENSP00000398698,9606.ENSP00000433623	TNF,TRAF6
GO Process	GO:0060964	Regulation of miRNA-mediated gene silencing	2	42	1.93	0.0160	9606.ENSP00000259808,9606.ENSP00000398698	RIPK1,TNF
GO Process	GO:0008625	Extrinsic apoptotic signaling pathway via death domain receptors	2	43	1.92	0.0165	9606.ENSP00000162749,9606.ENSP00000398698	TNFRSF1A,TNF
GO Process	GO:0032735	Positive regulation of interleukin-12 production	2	43	1.92	0.0165	9606.ENSP00000384273,9606.ENSP00000433623	RELA,TRAF6
GO	GO:1902	Positive regulation of	2	48	1.87	0.0198	9606.ENSP00000384273,9606.ENSP00000398698	RELA,TNF

Process	895	miRNA transcription						
GO Process	GO:2001238	Positive regulation of extrinsic apoptotic signaling pathway	2	50	1.86	0.0211	9606.ENSP00000259808,9606.ENSP00000398698	RIPK1,TNF
GO Process	GO:0030316	Osteoclast differentiation	2	54	1.82	0.0240	9606.ENSP00000398698,9606.ENSP00000433623	TNF,TRAF6
GO Process	GO:0051094	Positive regulation of developmental process	5	1332	0.83	0.0260	9606.ENSP00000162749,9606.ENSP00000226574,9606.ENSP00000259808,9606.ENSP00000398698,9606.ENSP00000433623	TNFRSF1A,NFKB1,RIPK1,TNF,TRAF6
GO Process	GO:0043331	Response to dsRNA	2	57	1.8	0.0265	9606.ENSP00000216797,9606.ENSP00000226574	NFKBIA,NFKB1
GO Process	GO:0043170	Macromolecule metabolic process	9	5781	0.45	0.0268	9606.ENSP00000162749,9606.ENSP00000226574,9606.ENSP00000259808,9606.ENSP00000358997,9606.ENSP00000359424,9606.ENSP00000384273,9606.ENSP00000398698,9606.ENSP00000430684,9606.ENSP00000433623	TNFRSF1A,NFKB1,RIPK1,IRAK1,CHUK,RELA,TNF,IKBKB,TRAF6
GO Process	GO:0006468	Protein phosphorylation	4	736	0.99	0.0289	9606.ENSP00000259808,9606.ENSP00000358997,9606.ENSP00000359424,9606.ENSP00000430684	RIPK1,IRAK1,CHUK,IKBKB
GO Process	GO:0006974	Cellular response to DNA damage stimulus	4	744	0.98	0.0299	9606.ENSP00000162749,9606.ENSP00000398698,9606.ENSP00000433623,9606.ENSP00000483825	TNFRSF1A,TNF,TRAF6,IKBKG
GO Process	GO:1901653	Cellular response to peptide	3	302	1.25	0.0300	9606.ENSP00000226574,9606.ENSP00000384273,9606.ENSP00000398698	NFKB1,RELA,TNF
GO Process	GO:0032481	Positive regulation of type I interferon production	2	63	1.75	0.0310	9606.ENSP00000358997,9606.ENSP00000359424	IRAK1,CHUK
GO Process	GO:0032374	Regulation of cholesterol transport	2	64	1.75	0.0319	9606.ENSP00000216797,9606.ENSP00000226574	NFKBIA,NFKB1
GO Process	GO:0042531	Positive regulation of tyrosine phosphorylation of STAT protein	2	65	1.74	0.0326	9606.ENSP00000162749,9606.ENSP00000398698	TNFRSF1A,TNF
GO Process	GO:0070301	Cellular response to hydrogen peroxide	2	66	1.73	0.0332	9606.ENSP00000259808,9606.ENSP00000384273	RIPK1,RELA

GO Process	GO:0008630	Intrinsic apoptotic signaling pathway in response to DNA damage	2	75	1.68	0.0416	9606.ENSP00000162749,9606.ENSP00000398698	TNFRSF1A,TNF
GO Process	GO:0071260	Cellular response to mechanical stimulus	2	75	1.68	0.0416	9606.ENSP00000162749,9606.ENSP00000226574	TNFRSF1A,NFKB1
GO Process	GO:0002637	Regulation of immunoglobulin production	2	76	1.67	0.0424	9606.ENSP00000398698,9606.ENSP00000433623	TNF,TRAF6
GO Process	GO:0008152	Metabolic process	10	7988	0.35	0.0427	9606.ENSP00000162749,9606.ENSP00000226574,9606.ENSP00000259808,9606.ENSP00000358997,9606.ENSP00000359424,9606.ENSP00000384273,9606.ENSP00000398698,9606.ENSP00000430684,9606.ENSP00000433623,9606.ENSP00000483825	TNFRSF1A,NFKB1,RIPK1,IRAK1,CHUK,RELA,TNF,IKBKB,TRAF6,IKBKG
GO Function	GO:0042802	Identical protein binding	10	2144	0.92	1.17e-05	9606.ENSP00000216797,9606.ENSP00000226574,9606.ENSP00000259808,9606.ENSP00000358997,9606.ENSP00000359424,9606.ENSP00000384273,9606.ENSP00000398698,9606.ENSP00000430684,9606.ENSP00000433623,9606.ENSP00000483825	NFKBIA,NFKB1,RIPK1,IRAK1,CHUK,RELA,TNF,IKBKB,TRAF6,IKBKG
GO Function	GO:1990459	Transferrin receptor binding	3	11	2.69	0.00012	9606.ENSP00000359424,9606.ENSP00000430684,9606.ENSP00000483825	CHUK,IKBKB,IKBKG
GO Function	GO:0019899	Enzyme binding	9	2084	0.89	0.00013	9606.ENSP00000216797,9606.ENSP00000226574,9606.ENSP00000259808,9606.ENSP00000358997,9606.ENSP00000384273,9606.ENSP00000398698,9606.ENSP00000430684,9606.ENSP00000433623,9606.ENSP00000483825	NFKBIA,NFKB1,RIPK1,IRAK1,RELA,TNF,IKBKB,TRAF6,IKBKG
GO Function	GO:0031625	Ubiquitin protein ligase binding	5	299	1.48	0.00045	9606.ENSP00000216797,9606.ENSP00000259808,9606.ENSP00000384273,9606.ENSP00000433623,9606.ENSP00000483825	NFKBIA,RIPK1,RELA,TRAF6,IKBKG
GO Function	GO:0042803	Protein homodimerization activity	6	709	1.18	0.00073	9606.ENSP00000259808,9606.ENSP00000358997,9606.ENSP00000359424,9606.ENSP00000384273,9606.ENSP00000430684,9606.ENSP00000483825	RIPK1,IRAK1,CHUK,RELA,IKBKB,IKBKG
GO Functio	GO:0008384	IkappaB kinase activity	2	3	3.08	0.0020	9606.ENSP00000359424,9606.ENSP00000430684	CHUK,IKBKB

n								
GO Function	GO:0032813	Tumor necrosis factor receptor superfamily binding	3	50	2.03	0.0020	9606.ENSP00000259808,9606.ENSP00000398698,9606.ENSP00000433623	RIPK1,TNF,TRAF6
GO Function	GO:0005515	Protein binding	11	7242	0.43	0.0082	9606.ENSP00000162749,9606.ENSP00000216797,9606.ENSP00000226574,9606.ENSP00000259808,9606.ENSP00000358997,9606.ENSP00000359424,9606.ENSP00000384273,9606.ENSP00000398698,9606.ENSP00000430684,9606.ENSP00000433623,9606.ENSP00000483825	TNFRSF1A,NFKBIA,NFKB1,RIPK1,IRAK1,CHUK,RELA,TNF,IKBKB,TRAF6,IKBKG
GO Function	GO:0016301	Kinase activity	5	788	1.06	0.0166	9606.ENSP00000259808,9606.ENSP00000358997,9606.ENSP00000359424,9606.ENSP00000430684,9606.ENSP00000483825	RIPK1,IRAK1,CHUK,IKBKB,IKBKG
GO Function	GO:0046982	Protein heterodimerization activity	4	368	1.29	0.0166	9606.ENSP00000358997,9606.ENSP00000359424,9606.ENSP00000430684,9606.ENSP00000483825	IRAK1,CHUK,IKBKB,IKBKG
GO Function	GO:0005102	Signaling receptor binding	6	1499	0.86	0.0246	9606.ENSP00000259808,9606.ENSP00000359424,9606.ENSP00000398698,9606.ENSP00000430684,9606.ENSP00000433623,9606.ENSP00000483825	RIPK1,CHUK,TNF,IKBKB,TRAF6,IKBKG
GO Function	GO:0004674	Protein serine/threonine kinase activity	4	434	1.22	0.0247	9606.ENSP00000259808,9606.ENSP00000358997,9606.ENSP00000359424,9606.ENSP00000430684	RIPK1,IRAK1,CHUK,IKBKB
GO Function	GO:0005164	Tumor necrosis factor receptor binding	2	32	2.05	0.0485	9606.ENSP00000398698,9606.ENSP00000433623	TNF,TRAF6
GO Function	GO:0051059	NF-kappaB binding	2	32	2.05	0.0485	9606.ENSP00000216797,9606.ENSP00000384273	NFKBIA,RELA
GO Component	GO:0008385	IkappaB kinase complex	3	7	2.89	3.17e-05	9606.ENSP00000359424,9606.ENSP00000430684,9606.ENSP00000483825	CHUK,IKBKB,IKBKG

GO Component	GO:0035631	CD40 receptor complex	3	12	2.65	6.00e-05	9606.ENSP00000359424,9606.ENSP00000430684,9606.ENSP00000433623	CHUK,IKBKB,TRAF6
GO Component	GO:0032991	Protein-containing complex	11	5506	0.55	0.00056	9606.ENSP00000162749,9606.ENSP00000216797,9606.ENSP00000226574,9606.ENSP00000259808,9606.ENSP00000358997,9606.ENSP00000359424,9606.ENSP00000384273,9606.ENSP00000398698,9606.ENSP00000430684,9606.ENSP00000433623,9606.ENSP00000483825	TNFRSF1A,NFKBIA,NFKB1,RIPK1,IRAK1,CHUK,RELA,TNF,IKBKB,TRAF6,IKBKG
GO Component	GO:0035525	NF-kappaB p50/p65 complex	2	2	3.25	0.00087	9606.ENSP00000226574,9606.ENSP00000384273	NFKB1,RELA
GO Component	GO:0043235	Receptor complex	5	418	1.33	0.00087	9606.ENSP00000162749,9606.ENSP00000259808,9606.ENSP00000359424,9606.ENSP00000430684,9606.ENSP00000433623	TNFRSF1A,RIPK1,CHUK,IKBKB,TRAF6
GO Component	GO:0033256	I-kappaB/NF-kappaB complex	2	4	2.95	0.0014	9606.ENSP00000216797,9606.ENSP00000226574	NFKBIA,NFKB1
GO Component	GO:0045121	Membrane raft	4	320	1.35	0.0055	9606.ENSP00000162749,9606.ENSP00000259808,9606.ENSP00000398698,9606.ENSP00000430684	TNFRSF1A,RIPK1,TNF,IKBKB
GO Component	GO:0009898	Cytoplasmic side of plasma membrane	3	174	1.49	0.0190	9606.ENSP00000359424,9606.ENSP00000430684,9606.ENSP00000433623	CHUK,IKBKB,TRAF6
GO Component	GO:0098797	Plasma membrane protein complex	4	589	1.08	0.0309	9606.ENSP00000259808,9606.ENSP00000359424,9606.ENSP00000430684,9606.ENSP00000433623	RIPK1,CHUK,IKBKB,TRAF6
GO Component	GO:0098552	Side of membrane	4	611	1.07	0.0333	9606.ENSP00000359424,9606.ENSP00000398698,9606.ENSP00000430684,9606.ENSP00000433623	CHUK,TNF,IKBKB,TRAF6
GO Co	GO:0005829	Cytosol	9	5438	0.47	0.0350	9606.ENSP00000216797,9606.ENSP00000226574,9606.ENSP00000259808,9606.ENSP00000358997,9606.ENSP000	NFKBIA,NFKB1,RIPK1,IRAK1,CHUK,RELA,

mponent							00359424,9606.ENSP00000384273,9606.ENSP00000430684,9606.ENSP00000433623,9606.ENSP00000483825	IKBKB,TRAF6,IKBKG
STRING clusters	CL:16587	Mixed, incl. TNFR1-induced NFkappaB signaling pathway, and NF-kappa-B/Dorsal	10	43	2.62	4.03e-22	9606.ENSP00000162749,9606.ENSP00000216797,9606.ENSP00000226574,9606.ENSP00000259808,9606.ENSP00000359424,9606.ENSP00000384273,9606.ENSP00000398698,9606.ENSP00000430684,9606.ENSP00000433623,9606.ENSP00000483825	TNFRSF1A,NFKBIA,NFKB1,RIPK1,CHUK,RELA,TNF,IKBKB,TRAF6,IKBKG
STRING clusters	CL:16584	Toll-like Receptor Cascades, and Novel intracellular components of RIG-I-like receptor pathway	11	105	2.27	4.13e-22	9606.ENSP00000162749,9606.ENSP00000216797,9606.ENSP00000226574,9606.ENSP00000259808,9606.ENSP00000358997,9606.ENSP00000359424,9606.ENSP00000384273,9606.ENSP00000398698,9606.ENSP00000430684,9606.ENSP00000433623,9606.ENSP00000483825	TNFRSF1A,NFKBIA,NFKB1,RIPK1,IRAK1,CHUK,RELA,TNF,IKBKB,TRAF6,IKBKG
STRING clusters	CL:16589	TNFR1-induced NFkappaB signaling pathway	6	25	2.63	2.72e-12	9606.ENSP00000162749,9606.ENSP00000259808,9606.ENSP00000359424,9606.ENSP00000398698,9606.ENSP00000430684,9606.ENSP00000483825	TNFRSF1A,RIPK1,CHUK,TNF,IKBKB,IKBKG
STRING clusters	CL:16629	IkappaB kinase complex	3	5	3.03	4.13e-06	9606.ENSP00000359424,9606.ENSP00000430684,9606.ENSP00000483825	CHUK,IKBKB,IKBKG
STRING clusters	CL:16644	Mixed, incl. NF-kappa-B/Dorsal, and D domain of beta-TrCP	3	13	2.62	3.67e-05	9606.ENSP00000216797,9606.ENSP00000226574,9606.ENSP00000384273	NFKBIA,NFKB1,RELA
STRING clusters	CL:16590	TNFR1-induced NFkappaB signaling pathway	3	20	2.43	0.00010	9606.ENSP00000162749,9606.ENSP00000259808,9606.ENSP00000398698	TNFRSF1A,RIPK1,TNF
STRING clusters	CL:16591	TNFR1-induced NFkappaB signaling pathway, and Death-inducing signaling complex assembly	2	14	2.41	0.0141	9606.ENSP00000259808,9606.ENSP00000398698	RIPK1,TNF
KEGG	hsa04064	NF-kappa B signaling pathway	11	101	2.29	4.05e-23	9606.ENSP00000162749,9606.ENSP00000216797,9606.ENSP00000226574,9606.ENSP00000259808,9606.ENSP00000358997,9606.ENSP00000359424,9606.ENSP00000384273,9606.ENSP00000398698,9606.ENSP00000430684,9606.ENSP00000433623,9606.ENSP00000483825	TNFRSF1A,NFKBIA,NFKB1,RIPK1,IRAK1,CHUK,RELA,TNF,IKBKB,TRAF6,IKBKG
KE	hsa05130	Pathogenic Escherichia	11	187	2.02	1.33e-20	9606.ENSP00000162749,9606.ENSP00000216797,9606.E	TNFRSF1A,NFKBIA,N

GG		coli infection					NSP00000226574,9606.ENSP00000259808,9606.ENSP00000358997,9606.ENSP00000359424,9606.ENSP00000384273,9606.ENSP00000398698,9606.ENSP00000430684,9606.ENSP00000433623,9606.ENSP00000483825	FKB1,RIPK1,IRAK1,CHUK,RELA,TNF,IKBKB,TRAF6,IKBKG
KEGG	hsa04620	Toll-like receptor signaling pathway	10	100	2.25	1.77e-20	9606.ENSP00000216797,9606.ENSP00000226574,9606.ENSP00000259808,9606.ENSP00000358997,9606.ENSP00000359424,9606.ENSP00000384273,9606.ENSP00000398698,9606.ENSP00000430684,9606.ENSP00000433623,9606.ENSP00000483825	NFKBIA,NFKB1,RIPK1,IRAK1,CHUK,RELA,TNF,IKBKB,TRAF6,IKBKG
KEGG	hsa05132	Salmonella infection	11	209	1.97	1.77e-20	9606.ENSP00000162749,9606.ENSP00000216797,9606.ENSP00000226574,9606.ENSP00000259808,9606.ENSP00000358997,9606.ENSP00000359424,9606.ENSP00000384273,9606.ENSP00000398698,9606.ENSP00000430684,9606.ENSP00000433623,9606.ENSP00000483825	TNFRSF1A,NFKBIA,NFKB1,RIPK1,IRAK1,CHUK,RELA,TNF,IKBKB,TRAF6,IKBKG
KEGG	hsa05142	Chagas disease	10	97	2.27	1.77e-20	9606.ENSP00000162749,9606.ENSP00000216797,9606.ENSP00000226574,9606.ENSP00000358997,9606.ENSP00000359424,9606.ENSP00000384273,9606.ENSP00000398698,9606.ENSP00000430684,9606.ENSP00000433623,9606.ENSP00000483825	TNFRSF1A,NFKBIA,NFKB1,IRAK1,CHUK,RELA,TNF,IKBKB,TRAF6,IKBKG
KEGG	hsa05145	Toxoplasmosis	10	103	2.24	1.77e-20	9606.ENSP00000162749,9606.ENSP00000216797,9606.ENSP00000226574,9606.ENSP00000358997,9606.ENSP00000359424,9606.ENSP00000384273,9606.ENSP00000398698,9606.ENSP00000430684,9606.ENSP00000433623,9606.ENSP00000483825	TNFRSF1A,NFKBIA,NFKB1,IRAK1,CHUK,RELA,TNF,IKBKB,TRAF6,IKBKG
KEGG	hsa05170	Human immunodeficiency virus 1 infection	11	203	1.99	1.77e-20	9606.ENSP00000162749,9606.ENSP00000216797,9606.ENSP00000226574,9606.ENSP00000259808,9606.ENSP00000358997,9606.ENSP00000359424,9606.ENSP00000384273,9606.ENSP00000398698,9606.ENSP00000430684,9606.ENSP00000433623,9606.ENSP00000483825	TNFRSF1A,NFKBIA,NFKB1,RIPK1,IRAK1,CHUK,RELA,TNF,IKBKB,TRAF6,IKBKG
KEGG	hsa04622	RIG-I-like receptor signaling pathway	9	69	2.37	3.39e-19	9606.ENSP00000216797,9606.ENSP00000226574,9606.ENSP00000259808,9606.ENSP00000359424,9606.ENSP00000384273,9606.ENSP00000398698,9606.ENSP00000430684,9606.ENSP00000433623,9606.ENSP00000483825	NFKBIA,NFKB1,RIPK1,CHUK,RELA,TNF,IKBKB,TRAF6,IKBKG
KEGG	hsa05160	Hepatitis C	10	157	2.06	5.92e-19	9606.ENSP00000162749,9606.ENSP00000216797,9606.ENSP00000226574,9606.ENSP00000259808,9606.ENSP00000359424,9606.ENSP00000384273,9606.ENSP00000398698,9606.ENSP00000430684,9606.ENSP00000433623,9606.ENSP00000483825	TNFRSF1A,NFKBIA,NFKB1,RIPK1,CHUK,RELA,TNF,IKBKB,TRAF6,IKBKG
KE	hsa05169	Epstein-Barr virus	10	192	1.97	3.74e-18	9606.ENSP00000216797,9606.ENSP00000226574,9606.E	NFKBIA,NFKB1,RIPK

GG		infection					NSP00000259808,9606.ENSP00000358997,9606.ENSP00000359424,9606.ENSP00000384273,9606.ENSP00000398698,9606.ENSP00000430684,9606.ENSP00000433623,9606.ENSP00000483825	1,IRAK1,CHUK,RELA,TNF,IKBKB,TRAF6,IKBKG
KEGG	hsa05131	Shigellosis	10	218	1.91	1.17e-17	9606.ENSP00000162749,9606.ENSP00000216797,9606.ENSP00000226574,9606.ENSP00000259808,9606.ENSP00000359424,9606.ENSP00000384273,9606.ENSP00000398698,9606.ENSP00000430684,9606.ENSP00000433623,9606.ENSP00000483825	TNFRSF1A,NFKBIA,NFKB1,RIPK1,CHUK,RELA,TNF,IKBKB,TRAF6,IKBKG
KEGG	hsa04668	TNF signaling pathway	9	111	2.16	1.29e-17	9606.ENSP00000162749,9606.ENSP00000216797,9606.ENSP00000226574,9606.ENSP00000259808,9606.ENSP00000359424,9606.ENSP00000384273,9606.ENSP00000398698,9606.ENSP00000430684,9606.ENSP00000483825	TNFRSF1A,NFKBIA,NFKB1,RIPK1,CHUK,RELA,TNF,IKBKB,IKBKG
KEGG	hsa04380	Osteoclast differentiation	9	120	2.13	2.33e-17	9606.ENSP00000162749,9606.ENSP00000216797,9606.ENSP00000226574,9606.ENSP00000359424,9606.ENSP00000384273,9606.ENSP00000398698,9606.ENSP00000430684,9606.ENSP00000433623,9606.ENSP00000483825	TNFRSF1A,NFKBIA,NFKB1,CHUK,RELA,TNF,IKBKB,TRAF6,IKBKG
KEGG	hsa05135	Yersinia infection	9	124	2.11	2.88e-17	9606.ENSP00000216797,9606.ENSP00000226574,9606.ENSP00000358997,9606.ENSP00000359424,9606.ENSP00000384273,9606.ENSP00000398698,9606.ENSP00000430684,9606.ENSP00000433623,9606.ENSP00000483825	NFKBIA,NFKB1,IRAK1,CHUK,RELA,TNF,IKBKB,TRAF6,IKBKG
KEGG	hsa04210	Apoptosis	9	131	2.09	4.32e-17	9606.ENSP00000162749,9606.ENSP00000216797,9606.ENSP00000226574,9606.ENSP00000259808,9606.ENSP00000359424,9606.ENSP00000384273,9606.ENSP00000398698,9606.ENSP00000430684,9606.ENSP00000483825	TNFRSF1A,NFKBIA,NFKB1,RIPK1,CHUK,RELA,TNF,IKBKB,IKBKG
KEGG	hsa04920	Adipocytokine signaling pathway	8	68	2.32	1.15e-16	9606.ENSP00000162749,9606.ENSP00000216797,9606.ENSP00000226574,9606.ENSP00000359424,9606.ENSP00000384273,9606.ENSP00000398698,9606.ENSP00000430684,9606.ENSP00000483825	TNFRSF1A,NFKBIA,NFKB1,CHUK,RELA,TNF,IKBKB,IKBKG
KEGG	hsa05161	Hepatitis B	9	158	2.01	1.94e-16	9606.ENSP00000216797,9606.ENSP00000226574,9606.ENSP00000358997,9606.ENSP00000359424,9606.ENSP00000384273,9606.ENSP00000398698,9606.ENSP00000430684,9606.ENSP00000433623,9606.ENSP00000483825	NFKBIA,NFKB1,IRAK1,CHUK,RELA,TNF,IKBKB,TRAF6,IKBKG
KEGG	hsa04621	NOD-like receptor signaling pathway	9	173	1.97	4.04e-16	9606.ENSP00000216797,9606.ENSP00000226574,9606.ENSP00000259808,9606.ENSP00000359424,9606.ENSP00000384273,9606.ENSP00000398698,9606.ENSP00000430684,9606.ENSP00000433623,9606.ENSP00000483825	NFKBIA,NFKB1,RIPK1,CHUK,RELA,TNF,IKBKB,TRAF6,IKBKG
KEGG	hsa04657	IL-17 signaling pathway	8	91	2.2	8.75e-16	9606.ENSP00000216797,9606.ENSP00000226574,9606.ENSP00000359424,9606.ENSP00000384273,9606.ENSP000	NFKBIA,NFKB1,CHUK,RELA,TNF,IKBKB,T

							00398698,9606.ENSP00000430684,9606.ENSP00000433623,9606.ENSP00000483825	RAF6,IKBKG
KEGG	hsa05163	Human cytomegalovirus infection	9	217	1.87	2.65e-15	9606.ENSP00000162749,9606.ENSP00000216797,9606.ENSP00000226574,9606.ENSP00000259808,9606.ENSP00000359424,9606.ENSP00000384273,9606.ENSP00000398698,9606.ENSP00000430684,9606.ENSP00000483825	TNFRSF1A,NFKBIA,NFKB1,RIPK1,CHUK,RELA,TNF,IKBKB,IKBKG
KEGG	hsa05168	Herpes simplex virus 1 infection	10	478	1.57	1.36e-14	9606.ENSP00000162749,9606.ENSP00000216797,9606.ENSP00000226574,9606.ENSP00000358997,9606.ENSP00000359424,9606.ENSP00000384273,9606.ENSP00000398698,9606.ENSP00000430684,9606.ENSP00000433623,9606.ENSP00000483825	TNFRSF1A,NFKBIA,NFKB1,IRAK1,CHUK,RELA,TNF,IKBKB,TRAF6,IKBKG
KEGG	hsa05162	Measles	8	137	2.02	1.75e-14	9606.ENSP00000216797,9606.ENSP00000226574,9606.ENSP00000358997,9606.ENSP00000359424,9606.ENSP00000384273,9606.ENSP00000430684,9606.ENSP00000433623,9606.ENSP00000483825	NFKBIA,NFKB1,IRAK1,CHUK,RELA,IKBKB,TRAF6,IKBKG
KEGG	hsa04623	Cytosolic DNA-sensing pathway	7	62	2.31	2.25e-14	9606.ENSP00000216797,9606.ENSP00000226574,9606.ENSP00000259808,9606.ENSP00000359424,9606.ENSP00000384273,9606.ENSP00000430684,9606.ENSP00000483825	NFKBIA,NFKB1,RIPK1,CHUK,RELA,IKBKB,IKBKG
KEGG	hsa04010	MAPK signaling pathway	9	286	1.75	2.50e-14	9606.ENSP00000162749,9606.ENSP00000226574,9606.ENSP00000358997,9606.ENSP00000359424,9606.ENSP00000384273,9606.ENSP00000398698,9606.ENSP00000430684,9606.ENSP00000433623,9606.ENSP00000483825	TNFRSF1A,NFKB1,IRAK1,CHUK,RELA,TNF,IKBKB,TRAF6,IKBKG
KEGG	hsa05164	Influenza A	8	163	1.94	5.90e-14	9606.ENSP00000162749,9606.ENSP00000216797,9606.ENSP00000226574,9606.ENSP00000359424,9606.ENSP00000384273,9606.ENSP00000398698,9606.ENSP00000430684,9606.ENSP00000483825	TNFRSF1A,NFKBIA,NFKB1,CHUK,RELA,TNF,IKBKB,IKBKG
KEGG	hsa01523	Antifolate resistance	6	31	2.54	1.69e-13	9606.ENSP00000226574,9606.ENSP00000359424,9606.ENSP00000384273,9606.ENSP00000398698,9606.ENSP00000430684,9606.ENSP00000483825	NFKB1,CHUK,RELA,TNF,IKBKB,IKBKG
KEGG	hsa05235	PD-L1 expression and PD-1 checkpoint pathway in cancer	7	87	2.16	1.81e-13	9606.ENSP00000216797,9606.ENSP00000226574,9606.ENSP00000359424,9606.ENSP00000384273,9606.ENSP00000430684,9606.ENSP00000433623,9606.ENSP00000483825	NFKBIA,NFKB1,CHUK,RELA,IKBKB,TRAF6,IKBKG
KEGG	hsa05222	Small cell lung cancer	7	92	2.13	2.53e-13	9606.ENSP00000216797,9606.ENSP00000226574,9606.ENSP00000359424,9606.ENSP00000384273,9606.ENSP00000430684,9606.ENSP00000433623,9606.ENSP00000483825	NFKBIA,NFKB1,CHUK,RELA,IKBKB,TRAF6,IKBKG
KE	hsa05166	Human T-cell leukemia	8	210	1.83	3.66e-13	9606.ENSP00000162749,9606.ENSP00000216797,9606.E	TNFRSF1A,NFKBIA,N

GG		virus 1 infection					NSP00000226574,9606.ENSP00000359424,9606.ENSP00000384273,9606.ENSP00000398698,9606.ENSP00000430684,9606.ENSP00000483825	FKB1,CHUK,RELA,TNF,IKBKB,IKBKG
KEGG	hsa04660	T cell receptor signaling pathway	7	100	2.1	4.13e-13	9606.ENSP00000216797,9606.ENSP00000226574,9606.ENSP00000359424,9606.ENSP00000384273,9606.ENSP00000398698,9606.ENSP00000430684,9606.ENSP00000483825	NFKBIA,NFKB1,CHUK,RELA,TNF,IKBKB,IKBKG
KEGG	hsa04625	C-type lectin receptor signaling pathway	7	101	2.09	4.28e-13	9606.ENSP00000216797,9606.ENSP00000226574,9606.ENSP00000359424,9606.ENSP00000384273,9606.ENSP00000398698,9606.ENSP00000430684,9606.ENSP00000483825	NFKBIA,NFKB1,CHUK,RELA,TNF,IKBKB,IKBKG
KEGG	hsa05418	Fluid shear stress and atherosclerosis	7	129	1.99	2.16e-12	9606.ENSP00000162749,9606.ENSP00000226574,9606.ENSP00000359424,9606.ENSP00000384273,9606.ENSP00000398698,9606.ENSP00000430684,9606.ENSP00000483825	TNFRSF1A,NFKB1,CHUK,RELA,TNF,IKBKB,IKBKG
KEGG	hsa05120	Epithelial cell signaling in Helicobacter pylori infection	6	65	2.22	8.16e-12	9606.ENSP00000216797,9606.ENSP00000226574,9606.ENSP00000359424,9606.ENSP00000384273,9606.ENSP00000430684,9606.ENSP00000483825	NFKBIA,NFKB1,CHUK,RELA,IKBKB,IKBKG
KEGG	hsa05140	Leishmaniasis	6	69	2.19	1.11e-11	9606.ENSP00000216797,9606.ENSP00000226574,9606.ENSP00000358997,9606.ENSP00000384273,9606.ENSP00000398698,9606.ENSP00000433623	NFKBIA,NFKB1,IRAK1,RELA,TNF,TRAF6
KEGG	hsa05220	Chronic myeloid leukemia	6	75	2.16	1.74e-11	9606.ENSP00000216797,9606.ENSP00000226574,9606.ENSP00000359424,9606.ENSP00000384273,9606.ENSP00000430684,9606.ENSP00000483825	NFKBIA,NFKB1,CHUK,RELA,IKBKB,IKBKG
KEGG	hsa04662	B cell receptor signaling pathway	6	78	2.14	2.12e-11	9606.ENSP00000216797,9606.ENSP00000226574,9606.ENSP00000359424,9606.ENSP00000384273,9606.ENSP00000430684,9606.ENSP00000483825	NFKBIA,NFKB1,CHUK,RELA,IKBKB,IKBKG
KEGG	hsa05167	Kaposi sarcoma-associated herpesvirus infection	7	187	1.83	2.33e-11	9606.ENSP00000162749,9606.ENSP00000216797,9606.ENSP00000226574,9606.ENSP00000359424,9606.ENSP00000384273,9606.ENSP00000430684,9606.ENSP00000483825	TNFRSF1A,NFKBIA,NFKB1,CHUK,RELA,IKBKB,IKBKG
KEGG	hsa04658	Th1 and Th2 cell differentiation	6	85	2.1	3.29e-11	9606.ENSP00000216797,9606.ENSP00000226574,9606.ENSP00000359424,9606.ENSP00000384273,9606.ENSP00000430684,9606.ENSP00000483825	NFKBIA,NFKB1,CHUK,RELA,IKBKB,IKBKG
KEGG	hsa05215	Prostate cancer	6	97	2.04	6.85e-11	9606.ENSP00000216797,9606.ENSP00000226574,9606.ENSP00000359424,9606.ENSP00000384273,9606.ENSP00000430684,9606.ENSP00000483825	NFKBIA,NFKB1,CHUK,RELA,IKBKB,IKBKG
KEGG	hsa04659	Th17 cell differentiation	6	99	2.04	7.51e-11	9606.ENSP00000216797,9606.ENSP00000226574,9606.ENSP00000359424,9606.ENSP00000384273,9606.ENSP000	NFKBIA,NFKB1,CHUK,RELA,IKBKB,IKBK

							00430684,9606.ENSP00000483825	G
KEGG	hsa04931	Insulin resistance	6	106	2.01	1.09e-10	9606.ENSP00000162749,9606.ENSP00000216797,9606.ENSP00000226574,9606.ENSP00000384273,9606.ENSP00000398698,9606.ENSP00000430684	TNFRSF1A,NFKBIA,NFKB1,RELA,TNF,IKBKB
KEGG	hsa04722	Neurotrophin signaling pathway	6	112	1.98	1.46e-10	9606.ENSP00000216797,9606.ENSP00000226574,9606.ENSP00000358997,9606.ENSP00000384273,9606.ENSP00000430684,9606.ENSP00000433623	NFKBIA,NFKB1,IRAK1,RELA,IKBKB,TRAF6
KEGG	hsa05165	Human papillomavirus infection	7	324	1.59	8.61e-10	9606.ENSP00000162749,9606.ENSP00000226574,9606.ENSP00000359424,9606.ENSP00000384273,9606.ENSP00000398698,9606.ENSP00000430684,9606.ENSP00000483825	TNFRSF1A,NFKB1,CHUK,RELA,TNF,IKBKB,IKBKG
KEGG	hsa05152	Tuberculosis	6	165	1.81	1.33e-09	9606.ENSP00000162749,9606.ENSP00000226574,9606.ENSP00000358997,9606.ENSP00000384273,9606.ENSP00000398698,9606.ENSP00000433623	TNFRSF1A,NFKB1,IRAK1,RELA,TNF,TRAF6
KEGG	hsa05221	Acute myeloid leukemia	5	67	2.13	1.92e-09	9606.ENSP00000226574,9606.ENSP00000359424,9606.ENSP00000384273,9606.ENSP00000430684,9606.ENSP00000483825	NFKB1,CHUK,RELA,IKBKB,IKBKG
KEGG	hsa05212	Pancreatic cancer	5	71	2.1	2.47e-09	9606.ENSP00000226574,9606.ENSP00000359424,9606.ENSP00000384273,9606.ENSP00000430684,9606.ENSP00000483825	NFKB1,CHUK,RELA,IKBKB,IKBKG
KEGG	hsa04062	Chemokine signaling pathway	6	186	1.76	2.51e-09	9606.ENSP00000216797,9606.ENSP00000226574,9606.ENSP00000359424,9606.ENSP00000384273,9606.ENSP00000430684,9606.ENSP00000483825	NFKBIA,NFKB1,CHUK,RELA,IKBKB,IKBKG
KEGG	hsa05133	Pertussis	5	73	2.09	2.70e-09	9606.ENSP00000226574,9606.ENSP00000358997,9606.ENSP00000384273,9606.ENSP00000398698,9606.ENSP00000433623	NFKB1,IRAK1,RELA,TNF,TRAF6
KEGG	hsa05200	Pathways in cancer	7	515	1.39	1.81e-08	9606.ENSP00000216797,9606.ENSP00000226574,9606.ENSP00000359424,9606.ENSP00000384273,9606.ENSP00000430684,9606.ENSP00000433623,9606.ENSP00000483825	NFKBIA,NFKB1,CHUK,RELA,IKBKB,TRAF6,IKBKG
KEGG	hsa04932	Non-alcoholic fatty liver disease	5	146	1.79	7.39e-08	9606.ENSP00000162749,9606.ENSP00000226574,9606.ENSP00000384273,9606.ENSP00000398698,9606.ENSP00000430684	TNFRSF1A,NFKB1,RELA,TNF,IKBKB
KEGG	hsa05010	Alzheimer disease	6	354	1.48	1.00e-07	9606.ENSP00000162749,9606.ENSP00000226574,9606.ENSP00000359424,9606.ENSP00000384273,9606.ENSP00000398698,9606.ENSP00000430684	TNFRSF1A,NFKB1,CHUK,RELA,TNF,IKBKB
KEGG	hsa05134	Legionellosis	4	55	2.11	1.52e-07	9606.ENSP00000216797,9606.ENSP00000226574,9606.ENSP00000384273,9606.ENSP00000398698	NFKBIA,NFKB1,RELA,TNF

KEGG	hsa04014	Ras signaling pathway	5	225	1.6	5.73e-07	9606.ENSP00000226574,9606.ENSP00000359424,9606.ENSP00000384273,9606.ENSP00000430684,9606.ENSP00000483825	NFKB1,CHUK,RELA,IKBKB,IKBKG
KEGG	hsa04071	Sphingolipid signaling pathway	4	116	1.79	2.60e-06	9606.ENSP00000162749,9606.ENSP00000226574,9606.ENSP00000384273,9606.ENSP00000398698	TNFRSF1A,NFKB1,RELA,TNF
KEGG	hsa04151	PI3K-Akt signaling pathway	5	349	1.41	4.69e-06	9606.ENSP00000226574,9606.ENSP00000359424,9606.ENSP00000384273,9606.ENSP00000430684,9606.ENSP00000483825	NFKB1,CHUK,RELA,IKBKB,IKBKG
KEGG	hsa04150	mTOR signaling pathway	4	150	1.68	6.80e-06	9606.ENSP00000162749,9606.ENSP00000359424,9606.ENSP00000398698,9606.ENSP00000430684	TNFRSF1A,CHUK,TNF,IKBKB
KEGG	hsa05203	Viral carcinogenesis	4	183	1.59	1.45e-05	9606.ENSP00000216797,9606.ENSP00000226574,9606.ENSP00000384273,9606.ENSP00000483825	NFKBIA,NFKB1,RELA,IKBKG
KEGG	hsa05321	Inflammatory bowel disease	3	59	1.96	2.78e-05	9606.ENSP00000226574,9606.ENSP00000384273,9606.ENSP00000398698	NFKB1,RELA,TNF
KEGG	hsa04933	AGE-RAGE signaling pathway in diabetic complications	3	96	1.75	0.00011	9606.ENSP00000226574,9606.ENSP00000384273,9606.ENSP00000398698	NFKB1,RELA,TNF
KEGG	hsa05146	Amoebiasis	3	101	1.73	0.00013	9606.ENSP00000226574,9606.ENSP00000384273,9606.ENSP00000398698	NFKB1,RELA,TNF
KEGG	hsa04926	Relaxin signaling pathway	3	126	1.63	0.00024	9606.ENSP00000216797,9606.ENSP00000226574,9606.ENSP00000384273	NFKBIA,NFKB1,RELA
KEGG	hsa04217	Necroptosis	3	147	1.56	0.00037	9606.ENSP00000162749,9606.ENSP00000259808,9606.ENSP00000398698	TNFRSF1A,RIPK1,TNF
KEGG	hsa04024	cAMP signaling pathway	3	207	1.41	0.00099	9606.ENSP00000216797,9606.ENSP00000226574,9606.ENSP00000384273	NFKBIA,NFKB1,RELA
KEGG	hsa04930	Type II diabetes mellitus	2	45	1.9	0.0016	9606.ENSP00000398698,9606.ENSP00000430684	TNF,IKBKB
KEGG	hsa05030	Cocaine addiction	2	49	1.86	0.0018	9606.ENSP00000226574,9606.ENSP00000384273	NFKB1,RELA
KEGG	hsa04917	Prolactin signaling pathway	2	68	1.72	0.0034	9606.ENSP00000226574,9606.ENSP00000384273	NFKB1,RELA
KEGG	hsa04211	Longevity regulating pathway	2	87	1.61	0.0054	9606.ENSP00000226574,9606.ENSP00000384273	NFKB1,RELA
KEGG	hsa04061	Viral protein interaction with cytokine and cytokine receptor	2	96	1.57	0.0065	9606.ENSP00000162749,9606.ENSP00000398698	TNFRSF1A,TNF
KEGG	hsa04066	HIF-1 signaling pathway	2	102	1.55	0.0072	9606.ENSP00000226574,9606.ENSP00000384273	NFKB1,RELA
KE	hsa04068	FoxO signaling pathway	2	126	1.45	0.0106	9606.ENSP00000359424,9606.ENSP00000430684	CHUK,IKBKB

GG								
KEGG	hsa04218	Cellular senescence	2	150	1.38	0.0147	9606.ENSP00000226574,9606.ENSP00000384273	NFKB1,RELA
KEGG	hsa05206	MicroRNAs in cancer	2	159	1.35	0.0162	9606.ENSP00000226574,9606.ENSP00000430684	NFKB1,IKBKB
KEGG	hsa05202	Transcriptional misregulation in cancer	2	171	1.32	0.0184	9606.ENSP00000226574,9606.ENSP00000384273	NFKB1,RELA
KEGG	hsa04060	Cytokine-cytokine receptor interaction	2	282	1.1	0.0474	9606.ENSP00000162749,9606.ENSP00000398698	TNFRSF1A,TNF
Reactome	HSA-73887	Death Receptor Signaling	11	152	2.11	2.01e-20	9606.ENSP00000162749,9606.ENSP00000216797,9606.ENSP00000226574,9606.ENSP00000259808,9606.ENSP00000358997,9606.ENSP00000359424,9606.ENSP00000384273,9606.ENSP00000398698,9606.ENSP00000430684,9606.ENSP00000433623,9606.ENSP00000483825	TNFRSF1A,NFKBIA,NFKB1,RIPK1,IRAK1,CHUK,RELA,TNF,IKBKB,TRAF6,IKBKG
Reactome	HSA-168164	Toll Like Receptor 3 (TLR3) Cascade	9	100	2.21	1.78e-16	9606.ENSP00000216797,9606.ENSP00000226574,9606.ENSP00000259808,9606.ENSP00000358997,9606.ENSP00000359424,9606.ENSP00000384273,9606.ENSP00000430684,9606.ENSP00000433623,9606.ENSP00000483825	NFKBIA,NFKB1,RIPK1,IRAK1,CHUK,RELA,IKBKB,TRAF6,IKBKG
Reactome	HSA-445989	TAK1-dependent IKK and NF-kappa-B activation	8	42	2.53	1.78e-16	9606.ENSP00000216797,9606.ENSP00000226574,9606.ENSP00000358997,9606.ENSP00000359424,9606.ENSP00000384273,9606.ENSP00000430684,9606.ENSP00000433623,9606.ENSP00000483825	NFKBIA,NFKB1,IRAK1,CHUK,RELA,IKBKB,TRAF6,IKBKG
Reactome	HSA-937061	TRIF(TICAM1)-mediated TLR4 signaling	9	104	2.19	1.78e-16	9606.ENSP00000216797,9606.ENSP00000226574,9606.ENSP00000259808,9606.ENSP00000358997,9606.ENSP00000359424,9606.ENSP00000384273,9606.ENSP00000430684,9606.ENSP00000433623,9606.ENSP00000483825	NFKBIA,NFKB1,RIPK1,IRAK1,CHUK,RELA,IKBKB,TRAF6,IKBKG
Reactome	HSA-1810476	RIP-mediated NFkB activation via ZBP1	7	17	2.87	1.89e-16	9606.ENSP00000216797,9606.ENSP00000226574,9606.ENSP00000259808,9606.ENSP00000359424,9606.ENSP00000384273,9606.ENSP00000430684,9606.ENSP00000483825	NFKBIA,NFKB1,RIPK1,CHUK,RELA,IKBKB,IKBKG
Reactome	HSA-933542	TRAF6 mediated NF-kB activation	7	25	2.7	1.22e-15	9606.ENSP00000216797,9606.ENSP00000226574,9606.ENSP00000359424,9606.ENSP00000384273,9606.ENSP00000430684,9606.ENSP00000433623,9606.ENSP00000483825	NFKBIA,NFKB1,CHUK,RELA,IKBKB,TRAF6,IKBKG
Reactome	HSA-5603029	IkBA variant leads to EDA-ID	6	7	3.19	2.22e-15	9606.ENSP00000216797,9606.ENSP00000226574,9606.ENSP00000359424,9606.ENSP00000384273,9606.ENSP00000430684,9606.ENSP00000483825	NFKBIA,NFKB1,CHUK,RELA,IKBKB,IKBKG
Reacto	HSA-168928	DDX58/IFIH1-mediated induction of interferon-	8	80	2.25	3.54e-15	9606.ENSP00000216797,9606.ENSP00000226574,9606.ENSP00000259808,9606.ENSP00000359424,9606.ENSP000	NFKBIA,NFKB1,RIPK1,CHUK,RELA,IKBKB

me		alpha/beta					00384273,9606.ENSP00000430684,9606.ENSP00000433623,9606.ENSP00000483825	,TRAF6,IKBKG
Reactome	HSA-209560	NF-kB is activated and signals survival	6	13	2.92	1.75e-14	9606.ENSP00000216797,9606.ENSP00000226574,9606.ENSP00000358997,9606.ENSP00000384273,9606.ENSP00000430684,9606.ENSP00000433623	NFKBIA,NFKB1,IRAK1,RELA,IKBKB,TRAF6
Reactome	HSA-449147	Signaling by Interleukins	10	453	1.6	4.23e-14	9606.ENSP00000162749,9606.ENSP00000216797,9606.ENSP00000226574,9606.ENSP00000358997,9606.ENSP00000359424,9606.ENSP00000384273,9606.ENSP00000398698,9606.ENSP00000430684,9606.ENSP00000433623,9606.ENSP00000483825	TNFRSF1A,NFKBIA,NFKB1,IRAK1,CHUK,RELA,TNF,IKBKB,TRAF6,IKBKG
Reactome	HSA-168643	Nucleotide-binding domain, leucine rich repeat containing receptor (NLR) signaling pathways	7	55	2.36	5.72e-14	9606.ENSP00000226574,9606.ENSP00000358997,9606.ENSP00000359424,9606.ENSP00000384273,9606.ENSP00000430684,9606.ENSP00000433623,9606.ENSP00000483825	NFKB1,IRAK1,CHUK,RELA,IKBKB,TRAF6,IKBKG
Reactome	HSA-2871837	FCERI mediated NF-kB activation	7	82	2.18	7.21e-13	9606.ENSP00000216797,9606.ENSP00000226574,9606.ENSP00000359424,9606.ENSP00000384273,9606.ENSP00000430684,9606.ENSP00000433623,9606.ENSP00000483825	NFKBIA,NFKB1,CHUK,RELA,IKBKB,TRAF6,IKBKG
Reactome	HSA-5357956	TNFR1-induced NFkappaB signaling pathway	6	33	2.51	1.23e-12	9606.ENSP00000162749,9606.ENSP00000259808,9606.ENSP00000359424,9606.ENSP00000398698,9606.ENSP00000430684,9606.ENSP00000483825	TNFRSF1A,RIPK1,CHUK,TNF,IKBKB,IKBKG
Reactome	HSA-202424	Downstream TCR signaling	7	92	2.13	1.38e-12	9606.ENSP00000216797,9606.ENSP00000226574,9606.ENSP00000359424,9606.ENSP00000384273,9606.ENSP00000430684,9606.ENSP00000433623,9606.ENSP00000483825	NFKBIA,NFKB1,CHUK,RELA,IKBKB,TRAF6,IKBKG
Reactome	HSA-5607764	CLEC7A (Dectin-1) signaling	7	96	2.12	1.78e-12	9606.ENSP00000216797,9606.ENSP00000226574,9606.ENSP00000359424,9606.ENSP00000384273,9606.ENSP00000430684,9606.ENSP00000433623,9606.ENSP00000483825	NFKBIA,NFKB1,CHUK,RELA,IKBKB,TRAF6,IKBKG
Reactome	HSA-9679506	SARS-CoV Infections	9	411	1.59	2.65e-12	9606.ENSP00000216797,9606.ENSP00000226574,9606.ENSP00000259808,9606.ENSP00000358997,9606.ENSP00000359424,9606.ENSP00000384273,9606.ENSP00000430684,9606.ENSP00000433623,9606.ENSP00000483825	NFKBIA,NFKB1,RIPK1,IRAK1,CHUK,RELA,IKBKB,TRAF6,IKBKG
Reactome	HSA-5357905	Regulation of TNFR1 signaling	6	46	2.37	6.53e-12	9606.ENSP00000162749,9606.ENSP00000259808,9606.ENSP00000359424,9606.ENSP00000398698,9606.ENSP00000430684,9606.ENSP00000483825	TNFRSF1A,RIPK1,CHUK,TNF,IKBKB,IKBKG
Reacto	HSA-937039	IRAK1 recruits IKK complex	5	13	2.84	8.87e-12	9606.ENSP00000358997,9606.ENSP00000359424,9606.ENSP00000430684,9606.ENSP00000433623,9606.ENSP000	IRAK1,CHUK,IKBKB,TRAF6,IKBKG

me							00483825	
Reactome	HSA-975144	IRAK1 recruits IKK complex upon TLR7/8 or 9 stimulation	5	13	2.84	8.87e-12	9606.ENSP00000358997,9606.ENSP00000359424,9606.ENSP00000430684,9606.ENSP00000433623,9606.ENSP00000483825	IRAK1,CHUK,IKBKB,TRAF6,IKBKG
Reactome	HSA-168927	TICAM1, RIP1-mediated IKK complex recruitment	5	17	2.72	2.43e-11	9606.ENSP00000259808,9606.ENSP00000359424,9606.ENSP00000430684,9606.ENSP00000433623,9606.ENSP00000483825	RIPK1,CHUK,IKBKB,TRAF6,IKBKG
Reactome	HSA-450294	MAP kinase activation	6	61	2.25	2.72e-11	9606.ENSP00000226574,9606.ENSP00000358997,9606.ENSP00000359424,9606.ENSP00000430684,9606.ENSP00000433623,9606.ENSP00000483825	NFKB1,IRAK1,CHUK,IKBKB,TRAF6,IKBKG
Reactome	HSA-1169091	Activation of NF-kappaB in B cells	6	66	2.21	4.16e-11	9606.ENSP00000216797,9606.ENSP00000226574,9606.ENSP00000359424,9606.ENSP00000384273,9606.ENSP00000430684,9606.ENSP00000483825	NFKBIA,NFKB1,CHUK,RELA,IKBKB,IKBKG
Reactome	HSA-937041	IKK complex recruitment mediated by RIP1	5	21	2.63	5.57e-11	9606.ENSP00000259808,9606.ENSP00000359424,9606.ENSP00000430684,9606.ENSP00000433623,9606.ENSP00000483825	RIPK1,CHUK,IKBKB,TRAF6,IKBKG
Reactome	HSA-168256	Immune System	11	1979	1.0	4.67e-10	9606.ENSP00000162749,9606.ENSP00000216797,9606.ENSP00000226574,9606.ENSP00000259808,9606.ENSP00000358997,9606.ENSP00000359424,9606.ENSP00000384273,9606.ENSP00000398698,9606.ENSP00000430684,9606.ENSP00000433623,9606.ENSP00000483825	TNFRSF1A,NFKBIA,NFKB1,RIPK1,IRAK1,CHUK,RELA,TNF,IKBKB,TRAF6,IKBKG
Reactome	HSA-168638	NOD1/2 Signaling Pathway	5	35	2.41	5.14e-10	9606.ENSP00000358997,9606.ENSP00000359424,9606.ENSP00000430684,9606.ENSP00000433623,9606.ENSP00000483825	IRAK1,CHUK,IKBKB,TRAF6,IKBKG
Reactome	HSA-933543	NF-kB activation through FADD/RIP-1 pathway mediated by caspase-8 and -10	4	13	2.74	4.99e-09	9606.ENSP00000259808,9606.ENSP00000359424,9606.ENSP00000430684,9606.ENSP00000483825	RIPK1,CHUK,IKBKB,IKBKG
Reactome	HSA-5684264	MAP3K8 (TPL2)-dependent MAPK1/3 activation	4	16	2.65	9.63e-09	9606.ENSP00000226574,9606.ENSP00000359424,9606.ENSP00000430684,9606.ENSP00000483825	NFKB1,CHUK,IKBKB,IKBKG
Reactome	HSA-9758274	Regulation of NF-kappa B signaling	4	17	2.62	1.17e-08	9606.ENSP00000359424,9606.ENSP00000430684,9606.ENSP00000433623,9606.ENSP00000483825	CHUK,IKBKB,TRAF6,IKBKG
Reactome	HSA-9692914	SARS-CoV-1-host interactions	5	95	1.97	5.05e-08	9606.ENSP00000216797,9606.ENSP00000226574,9606.ENSP00000259808,9606.ENSP00000384273,9606.ENSP00000433623	NFKBIA,NFKB1,RIPK1,RELA,TRAF6
Reacto	HSA-5602636	IKBKB deficiency causes SCID	3	3	3.25	9.36e-08	9606.ENSP00000359424,9606.ENSP00000430684,9606.ENSP00000483825	CHUK,IKBKB,IKBKG

me								
Reactome	HSA-5603027	IKBKG deficiency causes anhidrotic ectodermal dysplasia with immunodeficiency (EDA-ID) (via TLR)	3	3	3.25	9.36e-08	9606.ENSP00000359424,9606.ENSP00000430684,9606.ENSP00000483825	CHUK,IKBKB,IKBKG
Reactome	HSA-9705671	SARS-CoV-2 activates/modulates innate and adaptive immune responses	5	123	1.86	1.68e-07	9606.ENSP00000358997,9606.ENSP00000359424,9606.ENSP00000430684,9606.ENSP00000433623,9606.ENSP00000483825	IRAK1,CHUK,IKBKB,TRAF6,IKBKG
Reactome	HSA-9692916	SARS-CoV-1 activates/modulates innate immune responses	4	40	2.25	2.44e-07	9606.ENSP00000216797,9606.ENSP00000226574,9606.ENSP00000384273,9606.ENSP00000433623	NFKBIA,NFKB1,RELA,TRAF6
Reactome	HSA-4755510	SUMOylation of immune response proteins	3	10	2.73	1.22e-06	9606.ENSP00000216797,9606.ENSP00000384273,9606.ENSP00000483825	NFKBIA,RELA,IKBKG
Reactome	HSA-193692	Regulated proteolysis of p75NTR	3	11	2.69	1.51e-06	9606.ENSP00000226574,9606.ENSP00000384273,9606.ENSP00000433623	NFKB1,RELA,TRAF6
Reactome	HSA-209543	p75NTR recruits signalling complexes	3	13	2.62	2.26e-06	9606.ENSP00000358997,9606.ENSP00000430684,9606.ENSP00000433623	IRAK1,IKBKB,TRAF6
Reactome	HSA-450321	JNK (c-Jun kinases) phosphorylation and activation mediated by activated human TAK1	3	20	2.43	7.02e-06	9606.ENSP00000358997,9606.ENSP00000433623,9606.ENSP00000483825	IRAK1,TRAF6,IKBKG
Reactome	HSA-450302	Activated TAK1 mediates p38 MAPK activation	3	22	2.39	8.99e-06	9606.ENSP00000358997,9606.ENSP00000433623,9606.ENSP00000483825	IRAK1,TRAF6,IKBKG
Reactome	HSA-5357786	TNFR1-induced proapoptotic signaling	3	24	2.35	1.11e-05	9606.ENSP00000162749,9606.ENSP00000259808,9606.ENSP00000398698	TNFRSF1A,RIPK1,TNF
Reactome	HSA-5689896	Ovarian tumor domain proteases	3	39	2.14	4.29e-05	9606.ENSP00000259808,9606.ENSP00000433623,9606.ENSP00000483825	RIPK1,TRAF6,IKBKG
Reactome	HSA-5689880	Ub-specific processing proteases	4	203	1.55	0.00011	9606.ENSP00000216797,9606.ENSP00000259808,9606.ENSP00000433623,9606.ENSP00000483825	NFKBIA,RIPK1,TRAF6,IKBKG

Reactome	HSA-5626978	TNFR1-mediated ceramide production	2	6	2.78	0.00023	9606.ENSP00000162749,9606.ENSP00000398698	TNFRSF1A,TNF
Reactome	HSA-5660668	CLEC7A/inflammasome pathway	2	6	2.78	0.00023	9606.ENSP00000226574,9606.ENSP00000384273	NFKB1,RELA
Reactome	HSA-3134963	DEx/H-box helicases activate type I IFN and inflammatory cytokines production	2	7	2.71	0.00028	9606.ENSP00000226574,9606.ENSP00000384273	NFKB1,RELA
Reactome	HSA-381340	Transcriptional regulation of white adipocyte differentiation	3	84	1.81	0.00036	9606.ENSP00000226574,9606.ENSP00000384273,9606.ENSP00000398698	NFKB1,RELA,TNF
Reactome	HSA-1236974	ER-Phagosome pathway	3	89	1.78	0.00042	9606.ENSP00000359424,9606.ENSP00000430684,9606.ENSP00000483825	CHUK,IKBKB,IKBKG
Reactome	HSA-448706	Interleukin-1 processing	2	9	2.6	0.00042	9606.ENSP00000226574,9606.ENSP00000384273	NFKB1,RELA
Reactome	HSA-5668541	TNFR2 non-canonical NF-kB pathway	3	99	1.73	0.00057	9606.ENSP00000162749,9606.ENSP00000359424,9606.ENSP00000398698	TNFRSF1A,CHUK,TNF
Reactome	HSA-975110	TRAF6 mediated IRF7 activation in TLR7/8 or 9 signaling	2	12	2.47	0.00066	9606.ENSP00000358997,9606.ENSP00000433623	IRAK1,TRAF6
Reactome	HSA-844456	The NLRP3 inflammasome	2	16	2.35	0.0011	9606.ENSP00000226574,9606.ENSP00000384273	NFKB1,RELA
Reactome	HSA-5621575	CD209 (DC-SIGN) signaling	2	21	2.23	0.0018	9606.ENSP00000226574,9606.ENSP00000384273	NFKB1,RELA
Reactome	HSA-9660826	Purinergic signaling in leishmaniasis infection	2	26	2.14	0.0026	9606.ENSP00000226574,9606.ENSP00000384273	NFKB1,RELA
Reactome	HSA-8853884	Transcriptional Regulation by VENTX	2	39	1.96	0.0054	9606.ENSP00000226574,9606.ENSP00000384273	NFKB1,RELA
Reacto	HSA-6783783	Interleukin-10 signaling	2	45	1.9	0.0070	9606.ENSP00000162749,9606.ENSP00000398698	TNFRSF1A,TNF

me								
Reactome	HSA-3214841	PKMTs methylate histone lysines	2	47	1.88	0.0076	9606.ENSP00000226574,9606.ENSP00000384273	NFKB1,RELA
Reactome	HSA-1257604	PIP3 activates AKT signaling	3	266	1.31	0.0087	9606.ENSP00000358997,9606.ENSP00000359424,9606.ENSP00000433623	IRAK1,CHUK,TRAF6
Reactome	HSA-5607761	Dectin-1 mediated noncanonical NF-kB signaling	2	59	1.78	0.0115	9606.ENSP00000359424,9606.ENSP00000384273	CHUK,RELA
Reactome	HSA-597592	Post-translational protein modification	5	1405	0.8	0.0132	9606.ENSP00000216797,9606.ENSP00000259808,9606.ENSP00000384273,9606.ENSP00000433623,9606.ENSP00000483825	NFKBIA,RIPK1,RELA,TRAF6,IKBKG
Reactome	HSA-2559582	Senescence-Associated Secretory Phenotype (SASP)	2	81	1.65	0.0206	9606.ENSP00000226574,9606.ENSP00000384273	NFKB1,RELA
Reactome	HSA-6811558	PI5P, PP2A and IER3 Regulate PI3K/AKT Signaling	2	106	1.53	0.0341	9606.ENSP00000358997,9606.ENSP00000433623	IRAK1,TRAF6

Table 4. Pathway Enrichment Analysis for GO, KEGG, STRING clusters and Reactome Terms within JNK1 gene

#category	term ID	term description	observed gene count	background gene count	strength	false discovery rate	matching proteins in your network (IDs)	matching proteins in your network (labels)
GO Process	GO:0007254	JNK cascade	6	57	2.28	5.98e-09	9606.ENSP00000241014,9606.ENSP00000264110,9606.ENSP00000378974,9606.ENSP00000381070,9606.ENSP00000394560,9606.ENSP00000410402	MAPK8IP1,ATF2,MAPK8,MAP2K7,MAPK9,MAP2K4
GO Process	GO:0090398	Cellular senescence	5	60	2.17	5.95e-07	9606.ENSP00000269305,9606.ENSP00000378974,9606.ENSP00000381070,9606.ENSP00000394560,9606.ENSP00000410402	TP53,MAPK8,MAP2K7,MAPK9,MAP2K4
GO Process	GO:0038095	Fc-epsilon receptor signaling pathway	4	23	2.49	2.86e-06	9606.ENSP00000378974,9606.ENSP00000381070,9606.ENSP00000394560,9606.ENSP00000410402	MAPK8,MAP2K7,MAPK9,MAP2K4
GO Process	GO:0043065	Positive regulation of apoptotic process	7	507	1.39	5.32e-06	9606.ENSP00000264110,9606.ENSP00000269305,9606.ENSP00000360266,9606.ENSP00000378974,9606.ENSP00000381070,9606.ENSP00000394560,9606.ENSP00000410402	ATF2,TP53,JUN,MAPK8,MAP2K7,MAPK9,MAP2K4
GO Process	GO:0035556	Intracellular signal transduction	9	1518	1.03	8.17e-06	9606.ENSP00000241014,9606.ENSP00000264110,9606.ENSP00000269305,9606.ENSP00000300659,9606.ENSP00000360266,9606.ENSP00000378974,9606.ENSP00000381070,9606.ENSP00000394560,9606.ENSP00000410402	MAPK8IP1,ATF2,TP53,NFATC3,JUN,MAPK8,MAP2K7,MAPK9,MAP2K4
GO Proc	GO:00	Regulation of protein modification process	9	1560	1.0	9.35e	9606.ENSP00000241014,9606.ENSP00000264110,9606.ENSP00000269305,9606.ENSP00000298573,9606.ENSP00000360266,9606.ENSP000003789	MAPK8IP1,ATF2,TP53,DUSP16,JUN,MAPK8,

ess	31399				1	-06	74,9606.ENSP00000381070,9606.ENSP00000394560,9606.ENSP00000410402	MAP2K7,MAPK9,MAP2K4
GO Process	GO:0033554	Cellular response to stress	9	1572	1.01	9.35e-06	9606.ENSP00000241014,9606.ENSP00000252818,9606.ENSP00000264110,9606.ENSP00000269305,9606.ENSP00000360266,9606.ENSP00000378974,9606.ENSP00000381070,9606.ENSP00000394560,9606.ENSP00000410402	MAPK8IP1,JUND,ATF2,TP53,JUN,MAPK8,MAP2K7,MAPK9,MAP2K4
GO Process	GO:0043525	Positive regulation of neuron apoptotic process	4	54	2.12	2.46e-05	9606.ENSP00000264110,9606.ENSP00000269305,9606.ENSP00000381070,9606.ENSP00000410402	ATF2,TP53,MAP2K7,MAP2K4
GO Process	GO:0043523	Regulation of neuron apoptotic process	5	216	1.62	7.75e-05	9606.ENSP00000264110,9606.ENSP00000269305,9606.ENSP00000360266,9606.ENSP00000381070,9606.ENSP00000410402	ATF2,TP53,JUN,MAP2K7,MAP2K4
GO Process	GO:0034599	Cellular response to oxidative stress	5	224	1.6	8.67e-05	9606.ENSP00000264110,9606.ENSP00000269305,9606.ENSP00000360266,9606.ENSP00000378974,9606.ENSP00000394560	ATF2,TP53,JUN,MAPK8,MAPK9
GO Process	GO:0042981	Regulation of apoptotic process	8	1462	0.99	0.00012	9606.ENSP00000241014,9606.ENSP00000264110,9606.ENSP00000269305,9606.ENSP00000360266,9606.ENSP00000378974,9606.ENSP00000381070,9606.ENSP00000394560,9606.ENSP00000410402	MAPK8IP1,ATF2,TP53,JUN,MAPK8,MAP2K7,MAPK9,MAP2K4
GO Process	GO:0006950	Response to stress	10	3358	0.73	0.00015	9606.ENSP00000241014,9606.ENSP00000252818,9606.ENSP00000264110,9606.ENSP00000269305,9606.ENSP00000300659,9606.ENSP00000360266,9606.ENSP00000378974,9606.ENSP00000381070,9606.ENSP00000394560,9606.ENSP00000410402	MAPK8IP1,JUND,ATF2,TP53,NFATC3,JUN,MAPK8,MAP2K7,MAPK9,MAP2K4
GO Process	GO:1901700	Response to oxygen-containing compound	8	1547	0.97	0.00015	9606.ENSP00000252818,9606.ENSP00000264110,9606.ENSP00000269305,9606.ENSP00000360266,9606.ENSP00000378974,9606.ENSP00000381070,9606.ENSP00000394560,9606.ENSP00000410402	JUND,ATF2,TP53,JUN,MAPK8,MAP2K7,MAPK9,MAP2K4
GO Process	GO:1901522	Positive regulation of transcription from RNA polymerase II promoter involved in cellular response to chemical stimulus	3	23	2.37	0.00022	9606.ENSP00000264110,9606.ENSP00000269305,9606.ENSP00000360266	ATF2,TP53,JUN
GO Process	GO:0001932	Regulation of protein phosphorylation	7	1108	1.05	0.00031	9606.ENSP00000241014,9606.ENSP00000264110,9606.ENSP00000269305,9606.ENSP00000298573,9606.ENSP00000360266,9606.ENSP00000381070,9606.ENSP00000410402	MAPK8IP1,ATF2,TP53,DUSP16,JUN,MAP2K7,MAP2K4
GO	GO	Response to abiotic stimulus	7	1107	1.	0.0	9606.ENSP00000252818,9606.ENSP00000264110,9606.ENSP0000026930	JUND,ATF2,TP53,JUN,

Process	:0009628				05	0031	5,9606.ENSP00000360266,9606.ENSP00000378974,9606.ENSP00000381070,9606.ENSP00000410402	MAPK8,MAP2K7,MAP2K4
GO Process	GO:0035794	Positive regulation of mitochondrial membrane permeability	3	32	2.23	0.00045	9606.ENSP00000264110,9606.ENSP00000269305,9606.ENSP00000378974	ATF2,TP53,MAPK8
GO Process	GO:0046328	Regulation of JNK cascade	4	140	1.71	0.00045	9606.ENSP00000241014,9606.ENSP00000298573,9606.ENSP00000381070,9606.ENSP00000410402	MAPK8IP1,DUSP16,MAP2K7,MAP2K4
GO Process	GO:0010556	Regulation of macromolecule biosynthetic process	10	3980	0.65	0.00050	9606.ENSP00000241014,9606.ENSP00000252818,9606.ENSP00000264110,9606.ENSP00000269305,9606.ENSP00000300659,9606.ENSP00000360266,9606.ENSP00000378974,9606.ENSP00000381070,9606.ENSP00000394560,9606.ENSP00000410402	MAPK8IP1,JUND,ATF2,TP53,NFATC3,JUN,MAPK8,MAP2K7,MAPK9,MAP2K4
GO Process	GO:0006468	Protein phosphorylation	6	736	1.16	0.00052	9606.ENSP00000241014,9606.ENSP00000264110,9606.ENSP00000378974,9606.ENSP00000381070,9606.ENSP00000394560,9606.ENSP00000410402	MAPK8IP1,ATF2,MAPK8,MAP2K7,MAPK9,MAP2K4
GO Process	GO:0031323	Regulation of cellular metabolic process	11	5681	0.54	0.00052	9606.ENSP00000241014,9606.ENSP00000252818,9606.ENSP00000264110,9606.ENSP00000269305,9606.ENSP00000298573,9606.ENSP00000300659,9606.ENSP00000360266,9606.ENSP00000378974,9606.ENSP00000381070,9606.ENSP00000394560,9606.ENSP00000410402	MAPK8IP1,JUND,ATF2,TP53,DUSP16,NFATC3,JUN,MAPK8,MAP2K7,MAPK9,MAP2K4
GO Process	GO:0071276	Cellular response to cadmium ion	3	36	2.17	0.00052	9606.ENSP00000360266,9606.ENSP00000378974,9606.ENSP00000394560	JUN,MAPK8,MAPK9
GO Process	GO:0019219	Regulation of nucleobase-containing compound metabolic process	10	4074	0.64	0.00053	9606.ENSP00000241014,9606.ENSP00000252818,9606.ENSP00000264110,9606.ENSP00000269305,9606.ENSP00000300659,9606.ENSP00000360266,9606.ENSP00000378974,9606.ENSP00000381070,9606.ENSP00000394560,9606.ENSP00000410402	MAPK8IP1,JUND,ATF2,TP53,NFATC3,JUN,MAPK8,MAP2K7,MAPK9,MAP2K4
GO Process	GO:0051171	Regulation of nitrogen compound metabolic process	11	5734	0.54	0.00053	9606.ENSP00000241014,9606.ENSP00000252818,9606.ENSP00000264110,9606.ENSP00000269305,9606.ENSP00000298573,9606.ENSP00000300659,9606.ENSP00000360266,9606.ENSP00000378974,9606.ENSP00000381070,9606.ENSP00000394560,9606.ENSP00000410402	MAPK8IP1,JUND,ATF2,TP53,DUSP16,NFATC3,JUN,MAPK8,MAP2K7,MAPK9,MAP2K4
GO Process	GO:0007258	JUN phosphorylation	2	2	3.25	0.00067	9606.ENSP00000241014,9606.ENSP00000378974	MAPK8IP1,MAPK8

GO Process	GO:0080090	Regulation of primary metabolic process	11	5899	0.52	0.00067	9606.ENSP00000241014,9606.ENSP00000252818,9606.ENSP00000264110,9606.ENSP00000269305,9606.ENSP00000298573,9606.ENSP00000300659,9606.ENSP00000360266,9606.ENSP00000378974,9606.ENSP00000381070,9606.ENSP00000394560,9606.ENSP00000410402	MAPK8IP1,JUND,ATF2,TP53,DUSP16,NFATC3,JUN,MAPK8,MAP2K7,MAPK9,MAP2K4
GO Process	GO:0051173	Positive regulation of nitrogen compound metabolic process	9	3166	0.71	0.00097	9606.ENSP00000252818,9606.ENSP00000264110,9606.ENSP00000269305,9606.ENSP00000300659,9606.ENSP00000360266,9606.ENSP00000378974,9606.ENSP00000381070,9606.ENSP00000394560,9606.ENSP00000410402	JUND,ATF2,TP53,NFATC3,JUN,MAPK8,MAP2K7,MAPK9,MAP2K4
GO Process	GO:0072740	Cellular response to anisomycin	2	3	3.08	0.00097	9606.ENSP00000264110,9606.ENSP00000360266	ATF2,JUN
GO Process	GO:1990144	Intrinsic apoptotic signaling pathway in response to hypoxia	2	3	3.08	0.00097	9606.ENSP00000264110,9606.ENSP00000269305	ATF2,TP53
GO Process	GO:0060255	Regulation of macromolecule metabolic process	11	6249	0.5	0.0010	9606.ENSP00000241014,9606.ENSP00000252818,9606.ENSP00000264110,9606.ENSP00000269305,9606.ENSP00000298573,9606.ENSP00000300659,9606.ENSP00000360266,9606.ENSP00000378974,9606.ENSP00000381070,9606.ENSP00000394560,9606.ENSP00000410402	MAPK8IP1,JUND,ATF2,TP53,DUSP16,NFATC3,JUN,MAPK8,MAP2K7,MAPK9,MAP2K4
GO Process	GO:0071248	Cellular response to metal ion	4	198	1.56	0.0010	9606.ENSP00000252818,9606.ENSP00000360266,9606.ENSP00000378974,9606.ENSP00000394560	JUND,JUN,MAPK8,MAPK9
GO Process	GO:0009612	Response to mechanical stimulus	4	214	1.52	0.0013	9606.ENSP00000252818,9606.ENSP00000360266,9606.ENSP00000378974,9606.ENSP00000410402	JUND,JUN,MAPK8,MAP2K4
GO Process	GO:0043506	Regulation of JUN kinase activity	3	60	1.95	0.0014	9606.ENSP00000241014,9606.ENSP00000298573,9606.ENSP00000381070	MAPK8IP1,DUSP16,MAP2K7
GO Process	GO:0010035	Response to inorganic substance	5	532	1.23	0.0015	9606.ENSP00000252818,9606.ENSP00000264110,9606.ENSP00000360266,9606.ENSP00000378974,9606.ENSP00000394560	JUND,ATF2,JUN,MAPK8,MAPK9
GO Process	GO:00727	Cellular response to sorbitol	2	5	2.86	0.0015	9606.ENSP00000381070,9606.ENSP00000410402	MAP2K7,MAP2K4

	09							
GO Process	GO:1902893	Regulation of miRNA transcription	3	62	1.94	0.0015	9606.ENSP00000269305,9606.ENSP00000300659,9606.ENSP00000360266	TP53,NFATC3,JUN
GO Process	GO:0006355	Regulation of transcription, DNA-templated	9	3460	0.67	0.0016	9606.ENSP00000241014,9606.ENSP00000252818,9606.ENSP00000264110,9606.ENSP00000269305,9606.ENSP00000300659,9606.ENSP00000360266,9606.ENSP00000378974,9606.ENSP00000381070,9606.ENSP00000394560	MAPK8IP1,JUND,ATF2,TP53,NFATC3,JUN,MAPK8,MAP2K7,MAPK9
GO Process	GO:0010604	Positive regulation of macromolecule metabolic process	9	3533	0.66	0.0018	9606.ENSP00000252818,9606.ENSP00000264110,9606.ENSP00000269305,9606.ENSP00000300659,9606.ENSP00000360266,9606.ENSP00000378974,9606.ENSP00000381070,9606.ENSP00000394560,9606.ENSP00000410402	JUND,ATF2,TP53,NFATC3,JUN,MAPK8,MAP2K7,MAPK9,MAP2K4
GO Process	GO:0006915	Apoptotic process	6	1041	1.01	0.0019	9606.ENSP00000264110,9606.ENSP00000269305,9606.ENSP00000360266,9606.ENSP00000381070,9606.ENSP00000394560,9606.ENSP00000410402	ATF2,TP53,JUN,MAP2K7,MAPK9,MAP2K4
GO Process	GO:1901701	Cellular response to oxygen-containing compound	6	1057	1.01	0.0020	9606.ENSP00000269305,9606.ENSP00000360266,9606.ENSP00000378974,9606.ENSP00000381070,9606.ENSP00000394560,9606.ENSP00000410402	TP53,JUN,MAPK8,MAP2K7,MAPK9,MAP2K4
GO Process	GO:0006970	Response to osmotic stress	3	78	1.84	0.0023	9606.ENSP00000264110,9606.ENSP00000269305,9606.ENSP00000381070	ATF2,TP53,MAP2K7
GO Process	GO:0048511	Rhythmic process	4	270	1.42	0.0023	9606.ENSP00000252818,9606.ENSP00000269305,9606.ENSP00000378974,9606.ENSP00000394560	JUND,TP53,MAPK8,MAPK9
GO Process	GO:0070887	Cellular response to chemical stimulus	8	2609	0.74	0.0023	9606.ENSP00000252818,9606.ENSP00000264110,9606.ENSP00000269305,9606.ENSP00000360266,9606.ENSP00000378974,9606.ENSP00000381070,9606.ENSP00000394560,9606.ENSP00000410402	JUND,ATF2,TP53,JUN,MAPK8,MAP2K7,MAPK9,MAP2K4
GO Process	GO:0006796	Phosphate-containing compound metabolic process	7	1877	0.82	0.0033	9606.ENSP00000241014,9606.ENSP00000264110,9606.ENSP00000298573,9606.ENSP00000378974,9606.ENSP00000381070,9606.ENSP00000394560,9606.ENSP00000410402	MAPK8IP1,ATF2,DUSP16,MAPK8,MAP2K7,MAPK9,MAP2K4
GO Proc	GO:00	Regulation of MAPK cascade	5	668	1.1	0.003	9606.ENSP00000241014,9606.ENSP00000298573,9606.ENSP00000360266,9606.ENSP00000381070,9606.ENSP00000410402	MAPK8IP1,DUSP16,JUN,MAP2K7,MAP2K4

ess	43408				3	4		
GO Process	GO:0009416	Response to light stimulus	4	314	1.36	0.0037	9606.ENSP00000252818,9606.ENSP00000269305,9606.ENSP00000378974,9606.ENSP00000381070	JUND,TP53,MAPK8,MAP2K7
GO Process	GO:0046330	Positive regulation of JNK cascade	3	96	1.75	0.0037	9606.ENSP00000241014,9606.ENSP00000381070,9606.ENSP00000410402	MAPK8IP1,MAP2K7,MAP2K4
GO Process	GO:0010557	Positive regulation of macromolecule biosynthetic process	7	1935	0.81	0.0038	9606.ENSP00000252818,9606.ENSP00000264110,9606.ENSP00000269305,9606.ENSP00000300659,9606.ENSP00000360266,9606.ENSP00000381070,9606.ENSP00000410402	JUND,ATF2,TP53,NFATC3,JUN,MAP2K7,MAP2K4
GO Process	GO:0071496	Cellular response to external stimulus	4	317	1.35	0.0038	9606.ENSP00000264110,9606.ENSP00000269305,9606.ENSP00000378974,9606.ENSP00000410402	ATF2,TP53,MAPK8,MAP2K4
GO Process	GO:0080135	Regulation of cellular response to stress	5	712	1.1	0.0043	9606.ENSP00000241014,9606.ENSP00000269305,9606.ENSP00000298573,9606.ENSP00000381070,9606.ENSP00000410402	MAPK8IP1,TP53,DUSP16,MAP2K7,MAP2K4
GO Process	GO:0048522	Positive regulation of cellular process	10	5584	0.51	0.0049	9606.ENSP00000241014,9606.ENSP00000252818,9606.ENSP00000264110,9606.ENSP00000269305,9606.ENSP00000300659,9606.ENSP00000360266,9606.ENSP00000378974,9606.ENSP00000381070,9606.ENSP00000394560,9606.ENSP00000410402	MAPK8IP1,JUND,ATF2,TP53,NFATC3,JUN,MAPK8,MAP2K7,MAPK9,MAP2K4
GO Process	GO:0007166	Cell surface receptor signaling pathway	7	2040	0.79	0.0051	9606.ENSP00000264110,9606.ENSP00000269305,9606.ENSP00000360266,9606.ENSP00000378974,9606.ENSP00000381070,9606.ENSP00000394560,9606.ENSP00000410402	ATF2,TP53,JUN,MAPK8,MAP2K7,MAPK9,MAP2K4
GO Process	GO:0045935	Positive regulation of nucleobase-containing compound metabolic process	7	2056	0.79	0.0052	9606.ENSP00000252818,9606.ENSP00000264110,9606.ENSP00000269305,9606.ENSP00000300659,9606.ENSP00000360266,9606.ENSP00000381070,9606.ENSP00000410402	JUND,ATF2,TP53,NFATC3,JUN,MAP2K7,MAP2K4
GO Process	GO:0042752	Regulation of circadian rhythm	3	117	1.66	0.0058	9606.ENSP00000269305,9606.ENSP00000378974,9606.ENSP00000394560	TP53,MAPK8,MAPK9
GO	GO	Cellular response to reactive oxygen	3	118	1.	0.0	9606.ENSP00000360266,9606.ENSP00000378974,9606.ENSP0000039456	JUN,MAPK8,MAPK9

Process	:0034614	species			66	059	0	
GO Process	GO:0043508	Negative regulation of JUN kinase activity	2	15	2.38	0.0061	9606.ENSP00000241014,9606.ENSP00000298573	MAPK8IP1,DUSP16
GO Process	GO:0065009	Regulation of molecular function	8	3085	0.67	0.0061	9606.ENSP00000241014,9606.ENSP00000264110,9606.ENSP00000269305,9606.ENSP00000298573,9606.ENSP00000360266,9606.ENSP00000378974,9606.ENSP00000381070,9606.ENSP00000394560	MAPK8IP1,ATF2,TP53,DUSP16,JUN,MAPK8,MAP2K7,MAPK9
GO Process	GO:2000671	Regulation of motor neuron apoptotic process	2	15	2.38	0.0061	9606.ENSP00000381070,9606.ENSP00000410402	MAP2K7,MAP2K4
GO Process	GO:0031325	Positive regulation of cellular metabolic process	8	3114	0.66	0.0064	9606.ENSP00000252818,9606.ENSP00000264110,9606.ENSP00000269305,9606.ENSP00000300659,9606.ENSP00000360266,9606.ENSP00000381070,9606.ENSP00000394560,9606.ENSP00000410402	JUND,ATF2,TP53,NFATC3,JUN,MAP2K7,MAPK9,MAP2K4
GO Process	GO:0071456	Cellular response to hypoxia	3	128	1.62	0.0069	9606.ENSP00000252818,9606.ENSP00000264110,9606.ENSP00000269305	JUND,ATF2,TP53
GO Process	GO:0002682	Regulation of immune system process	6	1438	0.87	0.0077	9606.ENSP00000241014,9606.ENSP00000252818,9606.ENSP00000378974,9606.ENSP00000381070,9606.ENSP00000394560,9606.ENSP00000410402	MAPK8IP1,JUND,MAPK8,MAP2K7,MAPK9,MAP2K4
GO Process	GO:0031324	Negative regulation of cellular metabolic process	7	2265	0.74	0.0085	9606.ENSP00000241014,9606.ENSP00000252818,9606.ENSP00000264110,9606.ENSP00000269305,9606.ENSP00000298573,9606.ENSP00000300659,9606.ENSP00000360266	MAPK8IP1,JUND,ATF2,TP53,DUSP16,NFATC3,JUN
GO Process	GO:0044419	Biological process involved in interspecies interaction between organisms	6	1490	0.86	0.0091	9606.ENSP00000252818,9606.ENSP00000264110,9606.ENSP00000269305,9606.ENSP00000360266,9606.ENSP00000378974,9606.ENSP00000381070	JUND,ATF2,TP53,JUN,MAPK8,MAP2K7
GO Process	GO:0045936	Negative regulation of phosphate metabolic process	4	424	1.23	0.0092	9606.ENSP00000241014,9606.ENSP00000269305,9606.ENSP00000298573,9606.ENSP00000360266	MAPK8IP1,TP53,DUSP16,JUN

GO Process	GO:0051247	Positive regulation of protein metabolic process	6	1512	0.85	0.0096	9606.ENSP00000264110,9606.ENSP00000269305,9606.ENSP00000378974,9606.ENSP00000381070,9606.ENSP00000394560,9606.ENSP00000410402	ATF2,TP53,MAPK8,MAP2K7,MAPK9,MAP2K4
GO Process	GO:0009411	Response to UV	3	150	1.55	0.0099	9606.ENSP00000269305,9606.ENSP00000378974,9606.ENSP00000381070	TP53,MAPK8,MAP2K7
GO Process	GO:0009967	Positive regulation of signal transduction	6	1525	0.85	0.0099	9606.ENSP00000241014,9606.ENSP00000269305,9606.ENSP00000360266,9606.ENSP00000381070,9606.ENSP00000394560,9606.ENSP00000410402	MAPK8IP1,TP53,JUN,MAP2K7,MAPK9,MAP2K4
GO Process	GO:0043066	Negative regulation of apoptotic process	5	891	1.0	0.0099	9606.ENSP00000241014,9606.ENSP00000269305,9606.ENSP00000360266,9606.ENSP00000378974,9606.ENSP00000410402	MAPK8IP1,TP53,JUN,MAPK8,MAP2K4
GO Process	GO:0009605	Response to external stimulus	7	2355	0.73	0.0101	9606.ENSP00000252818,9606.ENSP00000264110,9606.ENSP00000269305,9606.ENSP00000360266,9606.ENSP00000378974,9606.ENSP00000381070,9606.ENSP00000410402	JUND,ATF2,TP53,JUN,MAPK8,MAP2K7,MAP2K4
GO Process	GO:0018193	Peptidyl-amino acid modification	5	900	1.0	0.0101	9606.ENSP00000264110,9606.ENSP00000378974,9606.ENSP00000381070,9606.ENSP00000394560,9606.ENSP00000410402	ATF2,MAPK8,MAP2K7,MAPK9,MAP2K4
GO Process	GO:1902108	Regulation of mitochondrial membrane permeability involved in apoptotic process	2	22	2.21	0.0101	9606.ENSP00000264110,9606.ENSP00000269305	ATF2,TP53
GO Process	GO:0051172	Negative regulation of nitrogen compound metabolic process	7	2403	0.72	0.0109	9606.ENSP00000241014,9606.ENSP00000252818,9606.ENSP00000264110,9606.ENSP00000269305,9606.ENSP00000298573,9606.ENSP00000300659,9606.ENSP00000360266	MAPK8IP1,JUND,ATF2,TP53,DUSP16,NFATC3,JUN
GO Process	GO:0048523	Negative regulation of cellular process	9	4736	0.53	0.0115	9606.ENSP00000241014,9606.ENSP00000252818,9606.ENSP00000264110,9606.ENSP00000269305,9606.ENSP00000298573,9606.ENSP00000300659,9606.ENSP00000360266,9606.ENSP00000378974,9606.ENSP00000410402	MAPK8IP1,JUND,ATF2,TP53,DUSP16,NFATC3,JUN,MAPK8,MAP2K4
GO Process	GO:00517	Cellular response to stimulus	10	6357	0.45	0.0119	9606.ENSP00000241014,9606.ENSP00000252818,9606.ENSP00000264110,9606.ENSP00000269305,9606.ENSP00000300659,9606.ENSP00000360266,9606.ENSP00000378974,9606.ENSP00000381070,9606.ENSP00000394	MAPK8IP1,JUND,ATF2,TP53,NFATC3,JUN,MAPK8,MAP2K7,MAP

	16						560,9606.ENSP00000410402	K9,MAP2K4
GO Process	GO:0010661	Positive regulation of muscle cell apoptotic process	2	25	2.16	0.0122	9606.ENSP00000269305,9606.ENSP00000410402	TP53,MAP2K4
GO Process	GO:0009267	Cellular response to starvation	3	169	1.55	0.0125	9606.ENSP00000264110,9606.ENSP00000269305,9606.ENSP00000378974	ATF2,TP53,MAPK8
GO Process	GO:0043410	Positive regulation of MAPK cascade	4	481	1.17	0.0126	9606.ENSP00000241014,9606.ENSP00000360266,9606.ENSP00000381070,9606.ENSP00000410402	MAPK8IP1,JUN,MAP2K7,MAP2K4
GO Process	GO:0000122	Negative regulation of transcription by RNA polymerase II	5	971	0.96	0.0127	9606.ENSP00000252818,9606.ENSP00000264110,9606.ENSP00000269305,9606.ENSP00000300659,9606.ENSP00000360266	JUND,ATF2,TP53,NFATC3,JUN
GO Process	GO:1902533	Positive regulation of intracellular signal transduction	5	997	0.95	0.0143	9606.ENSP00000241014,9606.ENSP00000269305,9606.ENSP00000360266,9606.ENSP00000381070,9606.ENSP00000410402	MAPK8IP1,TP53,JUN,MAP2K7,MAP2K4
GO Process	GO:0010638	Positive regulation of organelle organization	4	508	1.15	0.0150	9606.ENSP00000269305,9606.ENSP00000378974,9606.ENSP00000381070,9606.ENSP00000394560	TP53,MAPK8,MAP2K7,MAPK9
GO Process	GO:0031401	Positive regulation of protein modification process	5	1018	0.94	0.0151	9606.ENSP00000264110,9606.ENSP00000269305,9606.ENSP00000381070,9606.ENSP00000394560,9606.ENSP00000410402	ATF2,TP53,MAP2K7,MAPK9,MAP2K4
GO Process	GO:0045893	Positive regulation of transcription, DNA-templated	6	1710	0.88	0.0152	9606.ENSP00000252818,9606.ENSP00000264110,9606.ENSP00000269305,9606.ENSP00000300659,9606.ENSP00000360266,9606.ENSP00000381070	JUND,ATF2,TP53,NFATC3,JUN,MAP2K7
GO Process	GO:1902531	Regulation of intracellular signal transduction	6	1726	0.79	0.0157	9606.ENSP00000241014,9606.ENSP00000269305,9606.ENSP00000298573,9606.ENSP00000360266,9606.ENSP00000381070,9606.ENSP00000410402	MAPK8IP1,TP53,DUSP16,JUN,MAP2K7,MAP2K4
GO Proc	GO:00	Heart development	4	548	1.1	0.018	9606.ENSP00000264110,9606.ENSP00000269305,9606.ENSP00000360266,9606.ENSP00000410402	ATF2,TP53,JUN,MAP2K4

ess	07507				2	5		
GO Process	GO:0036211	Protein modification process	7	2674	0.67	0.0185	9606.ENSP00000241014,9606.ENSP00000264110,9606.ENSP00000298573,9606.ENSP00000378974,9606.ENSP00000381070,9606.ENSP00000394560,9606.ENSP00000410402	MAPK8IP1,ATF2,DUSP16,MAPK8,MAP2K7,MAPK9,MAP2K4
GO Process	GO:0010033	Response to organic substance	7	2692	0.67	0.0190	9606.ENSP00000252818,9606.ENSP00000264110,9606.ENSP00000269305,9606.ENSP00000360266,9606.ENSP00000378974,9606.ENSP00000381070,9606.ENSP00000410402	JUND,ATF2,TP53,JUN,MAPK8,MAP2K7,MAP2K4
GO Process	GO:0007178	Transmembrane receptor protein serine/threonine kinase signaling pathway	3	206	1.42	0.0193	9606.ENSP00000264110,9606.ENSP00000269305,9606.ENSP00000360266	ATF2,TP53,JUN
GO Process	GO:0010605	Negative regulation of macromolecule metabolic process	7	2760	0.66	0.0216	9606.ENSP00000241014,9606.ENSP00000252818,9606.ENSP00000264110,9606.ENSP00000269305,9606.ENSP00000298573,9606.ENSP00000300659,9606.ENSP00000360266	MAPK8IP1,JUND,ATF2,TP53,DUSP16,NFATC3,JUN
GO Process	GO:0044092	Negative regulation of molecular function	5	1143	0.89	0.0231	9606.ENSP00000241014,9606.ENSP00000269305,9606.ENSP00000298573,9606.ENSP00000360266,9606.ENSP00000378974	MAPK8IP1,TP53,DUSP16,JUN,MAPK8
GO Process	GO:0048583	Regulation of response to stimulus	8	3931	0.56	0.0234	9606.ENSP00000241014,9606.ENSP00000269305,9606.ENSP00000298573,9606.ENSP00000360266,9606.ENSP00000378974,9606.ENSP00000381070,9606.ENSP00000394560,9606.ENSP00000410402	MAPK8IP1,TP53,DUSP16,JUN,MAPK8,MAP2K7,MAPK9,MAP2K4
GO Process	GO:0071216	Cellular response to biotic stimulus	3	232	1.36	0.0255	9606.ENSP00000269305,9606.ENSP00000378974,9606.ENSP00000381070	TP53,MAPK8,MAP2K7
GO Process	GO:0003007	Heart morphogenesis	3	247	1.34	0.0299	9606.ENSP00000264110,9606.ENSP00000269305,9606.ENSP00000360266	ATF2,TP53,JUN
GO Process	GO:0006913	Nucleocytoplasmic transport	3	248	1.34	0.0300	9606.ENSP00000264110,9606.ENSP00000269305,9606.ENSP00000298573	ATF2,TP53,DUSP16
GO	GO	Regulation of signal transduction	7	2978	0.	0.0	9606.ENSP00000241014,9606.ENSP00000269305,9606.ENSP0000029857	MAPK8IP1,TP53,DUSP

Process	:0009966				62	322	3,9606.ENSP00000360266,9606.ENSP00000381070,9606.ENSP00000394560,9606.ENSP00000410402	16,JUN,MAP2K7,MAPK9,MAP2K4
GO Process	GO:0071310	Cellular response to organic substance	6	2019	0.73	0.0322	9606.ENSP00000264110,9606.ENSP00000269305,9606.ENSP00000360266,9606.ENSP00000378974,9606.ENSP00000381070,9606.ENSP00000410402	ATF2,TP53,JUN,MAPK8,MAP2K7,MAP2K4
GO Process	GO:1902895	Positive regulation of miRNA transcription	2	48	1.87	0.0322	9606.ENSP00000269305,9606.ENSP00000360266	TP53,JUN
GO Process	GO:0034198	Cellular response to amino acid starvation	2	49	1.86	0.0326	9606.ENSP00000264110,9606.ENSP00000378974	ATF2,MAPK8
GO Process	GO:0043618	Regulation of transcription from RNA polymerase II promoter in response to stress	2	49	1.86	0.0326	9606.ENSP00000269305,9606.ENSP00000360266	TP53,JUN
GO Process	GO:0009299	mRNA transcription	2	50	1.86	0.0330	9606.ENSP00000264110,9606.ENSP00000269305	ATF2,TP53
GO Process	GO:0051972	Regulation of telomerase activity	2	51	1.85	0.0340	9606.ENSP00000269305,9606.ENSP00000381070	TP53,MAP2K7
GO Process	GO:0051348	Negative regulation of transferase activity	3	274	1.29	0.0365	9606.ENSP00000241014,9606.ENSP00000269305,9606.ENSP00000298573	MAPK8IP1,TP53,DUSP16
GO Process	GO:0051707	Response to other organism	5	1328	0.83	0.0387	9606.ENSP00000252818,9606.ENSP00000264110,9606.ENSP00000269305,9606.ENSP00000378974,9606.ENSP00000381070	JUND,ATF2,TP53,MAPK8,MAP2K7
GO Process	GO:0043170	Macromolecule metabolic process	9	5781	0.45	0.0401	9606.ENSP00000241014,9606.ENSP00000252818,9606.ENSP00000264110,9606.ENSP00000269305,9606.ENSP00000298573,9606.ENSP00000378974,9606.ENSP00000381070,9606.ENSP00000394560,9606.ENSP00000410402	MAPK8IP1,JUND,ATF2,TP53,DUSP16,MAPK8,MAP2K7,MAPK9,MAP2K4

GO Process	GO:0001934	Positive regulation of protein phosphorylation	4	747	0.98	0.0446	9606.ENSP00000264110,9606.ENSP00000269305,9606.ENSP00000381070,9606.ENSP00000410402	ATF2,TP53,MAP2K7,MAP2K4
GO Process	GO:0032496	Response to lipopolysaccharide	3	314	1.23	0.0492	9606.ENSP00000252818,9606.ENSP00000378974,9606.ENSP00000381070	JUND,MAPK8,MAP2K7
GO Function	GO:0004707	MAP kinase activity	3	15	2.55	0.0052	9606.ENSP00000378974,9606.ENSP00000381070,9606.ENSP00000394560	MAPK8,MAP2K7,MAPK9
GO Function	GO:0019901	Protein kinase binding	6	702	1.18	0.021	9606.ENSP00000241014,9606.ENSP00000264110,9606.ENSP00000269305,9606.ENSP00000298573,9606.ENSP00000378974,9606.ENSP00000381070	MAPK8IP1,ATF2,TP53,DUSP16,MAPK8,MAP2K7
GO Function	GO:0001228	DNA-binding transcription activator activity, RNA polymerase II-specific	5	458	1.29	0.026	9606.ENSP00000252818,9606.ENSP00000264110,9606.ENSP00000269305,9606.ENSP00000300659,9606.ENSP00000360266	JUND,ATF2,TP53,NFATC3,JUN
GO Function	GO:0004705	JUN kinase activity	2	2	3.25	0.026	9606.ENSP00000378974,9606.ENSP00000394560	MAPK8,MAPK9
GO Function	GO:0004712	Protein serine/threonine/tyrosine kinase activity	3	44	2.09	0.026	9606.ENSP00000381070,9606.ENSP00000394560,9606.ENSP00000410402	MAP2K7,MAPK9,MAP2K4
GO Function	GO:0008545	JUN kinase kinase activity	2	2	3.25	0.026	9606.ENSP00000381070,9606.ENSP00000410402	MAP2K7,MAP2K4
GO Function	GO:0019899	Enzyme binding	8	2084	0.84	0.026	9606.ENSP00000241014,9606.ENSP00000252818,9606.ENSP00000264110,9606.ENSP00000269305,9606.ENSP00000298573,9606.ENSP00000360266,9606.ENSP00000378974,9606.ENSP00000381070	MAPK8IP1,JUND,ATF2,TP53,DUSP16,JUN,MAPK8,MAP2K7
GO Functio	GO:00350	Histone deacetylase regulator activity	2	5	2.86	0.029	9606.ENSP00000269305,9606.ENSP00000378974	TP53,MAPK8

n	33							
GO Function	GO:0061629	RNA polymerase II-specific DNA-binding transcription factor binding	4	353	1.31	0.0142	9606.ENSP00000252818,9606.ENSP00000264110,9606.ENSP00000269305,9606.ENSP00000360266	JUND,ATF2,TP53,JUN
GO Function	GO:0106310	Protein serine kinase activity	4	361	1.3	0.0142	9606.ENSP00000378974,9606.ENSP00000381070,9606.ENSP00000394560,9606.ENSP00000410402	MAPK8,MAP2K7,MAPK9,MAP2K4
GO Function	GO:0035497	cAMP response element binding	2	17	2.32	0.0183	9606.ENSP00000264110,9606.ENSP00000360266	ATF2,JUN
GO Function	GO:0004674	Protein serine/threonine kinase activity	4	434	1.22	0.0231	9606.ENSP00000378974,9606.ENSP00000381070,9606.ENSP00000394560,9606.ENSP00000410402	MAPK8,MAP2K7,MAPK9,MAP2K4
GO Function	GO:0019903	Protein phosphatase binding	3	152	1.55	0.0232	9606.ENSP00000269305,9606.ENSP00000378974,9606.ENSP00000381070	TP53,MAPK8,MAP2K7
GO Component	GO:0005667	Transcription regulator complex	5	517	1.24	0.0106	9606.ENSP00000252818,9606.ENSP00000264110,9606.ENSP00000269305,9606.ENSP00000300659,9606.ENSP00000360266	JUND,ATF2,TP53,NFATC3,JUN
GO Component	GO:0017053	Transcription repressor complex	3	77	1.84	0.0106	9606.ENSP00000252818,9606.ENSP00000269305,9606.ENSP00000360266	JUND,TP53,JUN
GO Component	GO:0035976	Transcription factor AP-1 complex	2	5	2.86	0.0106	9606.ENSP00000252818,9606.ENSP00000360266	JUND,JUN
GO Component	GO:0005634	Nucleus	11	7672	0.41	0.0161	9606.ENSP00000241014,9606.ENSP00000252818,9606.ENSP00000264110,9606.ENSP00000269305,9606.ENSP00000298573,9606.ENSP00000300659,9606.ENSP00000360266,9606.ENSP00000378974,9606.ENSP00000381070,9606.ENSP00000394560,9606.ENSP00000410402	MAPK8IP1,JUND,ATF2,TP53,DUSP16,NFATC3,JUN,MAPK8,MAP2K7,MAPK9,MAP2K4
STRING	CL:18	Mixed, incl. Basic-leucine zipper domain, and Host-pathogen	9	98	2.2	7.24e	9606.ENSP00000241014,9606.ENSP00000252818,9606.ENSP00000264110,9606.ENSP00000298573,9606.ENSP00000360266,9606.ENSP000003789	MAPK8IP1,JUND,ATF2,DUSP16,JUN,MAPK8

clusters	397	interaction of human coronaviruses - MAPK signaling			2	-16	74,9606.ENSP00000381070,9606.ENSP00000394560,9606.ENSP00000410402	,MAP2K7,MAPK9,MAP2K4
STRING clusters	CL:18400	Mixed, incl. Host-pathogen interaction of human coronaviruses - MAPK signaling, and Protein tyrosine/threonine phosphatase activity	6	41	2.42	6.91e-11	9606.ENSP00000241014,9606.ENSP00000298573,9606.ENSP00000378974,9606.ENSP00000381070,9606.ENSP00000394560,9606.ENSP00000410402	MAPK8IP1,DUSP16,MAPK8,MAP2K7,MAPK9,MAP2K4
STRING clusters	CL:18403	Mixed, incl. MAP kinase activity, and Protein tyrosine/threonine phosphatase activity	4	23	2.49	5.98e-07	9606.ENSP00000298573,9606.ENSP00000378974,9606.ENSP00000394560,9606.ENSP00000410402	DUSP16,MAPK8,MAPK9,MAP2K4
STRING clusters	CL:18408	JUN kinase activity, and JUN kinase kinase activity	3	5	3.03	3.68e-06	9606.ENSP00000378974,9606.ENSP00000394560,9606.ENSP00000410402	MAPK8,MAPK9,MAP2K4
STRING clusters	CL:18506	Response of EIF2AK1 (HRI) to heme deficiency, and Transcription factor AP-1 complex	3	18	2.47	7.11e-05	9606.ENSP00000252818,9606.ENSP00000264110,9606.ENSP00000360266	JUND,ATF2,JUN
STRING clusters	CL:18468	JNK-interacting protein 1, and JUN kinase kinase activity	2	5	2.86	0.0018	9606.ENSP00000241014,9606.ENSP00000381070	MAPK8IP1,MAP2K7
KEGG	hsa04010	MAPK signaling pathway	11	286	1.84	2.54e-18	9606.ENSP00000241014,9606.ENSP00000252818,9606.ENSP00000264110,9606.ENSP00000269305,9606.ENSP00000298573,9606.ENSP00000300659,9606.ENSP00000360266,9606.ENSP00000378974,9606.ENSP00000381070,9606.ENSP00000394560,9606.ENSP00000410402	MAPK8IP1,JUND,ATF2,TP53,DUSP16,NFATC3,JUN,MAPK8,MAP2K7,MAPK9,MAP2K4
KEGG	hsa05161	Hepatitis B	8	158	1.96	5.80e-13	9606.ENSP00000264110,9606.ENSP00000269305,9606.ENSP00000300659,9606.ENSP00000360266,9606.ENSP00000378974,9606.ENSP00000381070,9606.ENSP00000394560,9606.ENSP00000410402	ATF2,TP53,NFATC3,JUN,MAPK8,MAP2K7,MAPK9,MAP2K4
KEGG	hsa05167	Kaposi sarcoma-associated herpesvirus infection	7	187	1.83	2.87e-10	9606.ENSP00000269305,9606.ENSP00000300659,9606.ENSP00000360266,9606.ENSP00000378974,9606.ENSP00000381070,9606.ENSP00000394560,9606.ENSP00000410402	TP53,NFATC3,JUN,MAPK8,MAP2K7,MAPK9,MAP2K4
KEGG	hsa05166	Human T-cell leukemia virus 1 infection	7	210	1.78	4.75e-10	9606.ENSP00000264110,9606.ENSP00000269305,9606.ENSP00000300659,9606.ENSP00000360266,9606.ENSP00000378974,9606.ENSP00000394560,9606.ENSP00000410402	ATF2,TP53,NFATC3,JUN,MAPK8,MAPK9,MAP2K4

KEGG	hsa04668	TNF signaling pathway	6	111	1.99	1.16e-09	9606.ENSP00000264110,9606.ENSP00000360266,9606.ENSP00000378974,9606.ENSP00000381070,9606.ENSP00000394560,9606.ENSP00000410402	ATF2,JUN,MAPK8,MAP2K7,MAPK9,MAP2K4
KEGG	hsa04926	Relaxin signaling pathway	6	126	1.93	1.85e-09	9606.ENSP00000264110,9606.ENSP00000360266,9606.ENSP00000378974,9606.ENSP00000381070,9606.ENSP00000394560,9606.ENSP00000410402	ATF2,JUN,MAPK8,MAP2K7,MAPK9,MAP2K4
KEGG	hsa05135	Yersinia infection	6	124	1.94	1.85e-09	9606.ENSP00000300659,9606.ENSP00000360266,9606.ENSP00000378974,9606.ENSP00000381070,9606.ENSP00000394560,9606.ENSP00000410402	NFATC3,JUN,MAPK8,MAP2K7,MAPK9,MAP2K4
KEGG	hsa05418	Fluid shear stress and atherosclerosis	6	129	1.92	1.85e-09	9606.ENSP00000269305,9606.ENSP00000360266,9606.ENSP00000378974,9606.ENSP00000381070,9606.ENSP00000394560,9606.ENSP00000410402	TP53,JUN,MAPK8,MAP2K7,MAPK9,MAP2K4
KEGG	hsa05169	Epstein-Barr virus infection	6	192	1.75	1.58e-08	9606.ENSP00000269305,9606.ENSP00000360266,9606.ENSP00000378974,9606.ENSP00000381070,9606.ENSP00000394560,9606.ENSP00000410402	TP53,JUN,MAPK8,MAP2K7,MAPK9,MAP2K4
KEGG	hsa04012	ErbB signaling pathway	5	81	2.04	2.14e-08	9606.ENSP00000360266,9606.ENSP00000378974,9606.ENSP00000381070,9606.ENSP00000394560,9606.ENSP00000410402	JUN,MAPK8,MAP2K7,MAPK9,MAP2K4
KEGG	hsa04912	GnRH signaling pathway	5	87	2.01	2.74e-08	9606.ENSP00000360266,9606.ENSP00000378974,9606.ENSP00000381070,9606.ENSP00000394560,9606.ENSP00000410402	JUN,MAPK8,MAP2K7,MAPK9,MAP2K4
KEGG	hsa04620	Toll-like receptor signaling pathway	5	100	1.95	4.92e-08	9606.ENSP00000360266,9606.ENSP00000378974,9606.ENSP00000381070,9606.ENSP00000394560,9606.ENSP00000410402	JUN,MAPK8,MAP2K7,MAPK9,MAP2K4
KEGG	hsa04660	T cell receptor signaling pathway	5	100	1.95	4.92e-08	9606.ENSP00000300659,9606.ENSP00000360266,9606.ENSP00000378974,9606.ENSP00000381070,9606.ENSP00000394560	NFATC3,JUN,MAPK8,MAP2K7,MAPK9
KEGG	hsa04722	Neurotrophin signaling pathway	5	112	1.9	7.29e-	9606.ENSP00000269305,9606.ENSP00000360266,9606.ENSP00000378974,9606.ENSP00000381070,9606.ENSP00000394560	TP53,JUN,MAPK8,MAP2K7,MAPK9

						08		
KEGG	hsa04380	Osteoclast differentiation	5	120	1.87	9.50e-08	9606.ENSP00000252818,9606.ENSP00000360266,9606.ENSP00000378974,9606.ENSP00000381070,9606.ENSP00000394560	JUND,JUN,MAPK8,MAP2K7,MAPK9
KEGG	hsa04310	Wnt signaling pathway	5	154	1.76	2.99e-07	9606.ENSP00000269305,9606.ENSP00000300659,9606.ENSP00000360266,9606.ENSP00000378974,9606.ENSP00000394560	TP53,NFATC3,JUN,MAPK8,MAPK9
KEGG	hsa04137	Mitophagy - animal	4	64	2.05	8.30e-07	9606.ENSP00000269305,9606.ENSP00000360266,9606.ENSP00000378974,9606.ENSP00000394560	TP53,JUN,MAPK8,MAPK9
KEGG	hsa04664	Fc epsilon RI signaling pathway	4	65	2.04	8.32e-07	9606.ENSP00000378974,9606.ENSP00000381070,9606.ENSP00000394560,9606.ENSP00000410402	MAPK8,MAP2K7,MAPK9,MAP2K4
KEGG	hsa05120	Epithelial cell signaling in Helicobacter pylori infection	4	65	2.04	8.32e-07	9606.ENSP00000360266,9606.ENSP00000378974,9606.ENSP00000394560,9606.ENSP00000410402	JUN,MAPK8,MAPK9,MAP2K4
KEGG	hsa05170	Human immunodeficiency virus 1 infection	5	203	1.64	9.20e-07	9606.ENSP00000300659,9606.ENSP00000360266,9606.ENSP00000378974,9606.ENSP00000381070,9606.ENSP00000394560	NFATC3,JUN,MAPK8,MAP2K7,MAPK9
KEGG	hsa05132	Salmonella infection	5	209	1.63	1.01e-06	9606.ENSP00000360266,9606.ENSP00000378974,9606.ENSP00000381070,9606.ENSP00000394560,9606.ENSP00000410402	JUN,MAPK8,MAP2K7,MAPK9,MAP2K4
KEGG	hsa05210	Colorectal cancer	4	82	1.94	1.66e-06	9606.ENSP00000269305,9606.ENSP00000360266,9606.ENSP00000378974,9606.ENSP00000394560	TP53,JUN,MAPK8,MAPK9
KEGG	hsa04658	Th1 and Th2 cell differentiation	4	85	1.93	1.83e-06	9606.ENSP00000300659,9606.ENSP00000360266,9606.ENSP00000378974,9606.ENSP00000394560	NFATC3,JUN,MAPK8,MAPK9
KEGG	hsa046	IL-17 signaling pathway	4	91	1.9	2.28e	9606.ENSP00000252818,9606.ENSP00000360266,9606.ENSP00000378974,9606.ENSP00000394560	JUND,JUN,MAPK8,MAPK9

	57					-06		
KEGG	hsa01522	Endocrine resistance	4	94	1.88	2.48e-06	9606.ENSP00000269305,9606.ENSP00000360266,9606.ENSP00000378974,9606.ENSP00000394560	TP53,JUN,MAPK8,MAPK9
KEGG	hsa05142	Chagas disease	4	97	1.87	2.69e-06	9606.ENSP00000360266,9606.ENSP00000378974,9606.ENSP00000394560,9606.ENSP00000410402	JUN,MAPK8,MAPK9,MAP2K4
KEGG	hsa04659	Th17 cell differentiation	4	99	1.86	2.81e-06	9606.ENSP00000300659,9606.ENSP00000360266,9606.ENSP00000378974,9606.ENSP00000394560	NFATC3,JUN,MAPK8,MAPK9
KEGG	hsa04625	C-type lectin receptor signaling pathway	4	101	1.85	2.93e-06	9606.ENSP00000300659,9606.ENSP00000360266,9606.ENSP00000378974,9606.ENSP00000394560	NFATC3,JUN,MAPK8,MAPK9
KEGG	hsa04935	Growth hormone synthesis, secretion and action	4	117	1.79	5.00e-06	9606.ENSP00000264110,9606.ENSP00000378974,9606.ENSP00000394560,9606.ENSP00000410402	ATF2,MAPK8,MAPK9,MAP2K4
KEGG	hsa04210	Apoptosis	4	131	1.74	7.50e-06	9606.ENSP00000269305,9606.ENSP00000360266,9606.ENSP00000378974,9606.ENSP00000394560	TP53,JUN,MAPK8,MAPK9
KEGG	hsa05162	Measles	4	137	1.72	8.63e-06	9606.ENSP00000269305,9606.ENSP00000360266,9606.ENSP00000378974,9606.ENSP00000394560	TP53,JUN,MAPK8,MAPK9
KEGG	hsa04530	Tight junction	4	157	1.66	1.42e-05	9606.ENSP00000360266,9606.ENSP00000378974,9606.ENSP00000381070,9606.ENSP00000394560	JUN,MAPK8,MAP2K7,MAPK9
KEGG	hsa05131	Shigellosis	4	218	1.52	4.95e-05	9606.ENSP00000269305,9606.ENSP00000360266,9606.ENSP00000378974,9606.ENSP00000394560	TP53,JUN,MAPK8,MAPK9
KE	hsa	Pancreatic cancer	3	71	1.	8.1	9606.ENSP00000269305,9606.ENSP00000378974,9606.ENSP0000039456	TP53,MAPK8,MAPK9

GG	05212				88	1e-05	0	
KEGG	hsa05133	Pertussis	3	73	1.87	8.54e-05	9606.ENSP00000360266,9606.ENSP00000378974,9606.ENSP00000394560	JUN,MAPK8,MAPK9
KEGG	hsa05016	Huntington disease	4	295	1.39	0.00015	9606.ENSP00000269305,9606.ENSP00000378974,9606.ENSP00000381070,9606.ENSP00000394560	TP53,MAPK8,MAP2K7,MAPK9
KEGG	hsa04933	AGE-RAGE signaling pathway in diabetic complications	3	96	1.75	0.00017	9606.ENSP00000360266,9606.ENSP00000378974,9606.ENSP00000394560	JUN,MAPK8,MAPK9
KEGG	hsa05231	Choline metabolism in cancer	3	95	1.75	0.00017	9606.ENSP00000360266,9606.ENSP00000378974,9606.ENSP00000394560	JUN,MAPK8,MAPK9
KEGG	hsa04071	Sphingolipid signaling pathway	3	116	1.67	0.00029	9606.ENSP00000269305,9606.ENSP00000378974,9606.ENSP00000394560	TP53,MAPK8,MAPK9
KEGG	hsa04728	Dopaminergic synapse	3	126	1.63	0.00037	9606.ENSP00000264110,9606.ENSP00000378974,9606.ENSP00000394560	ATF2,MAPK8,MAPK9
KEGG	hsa04932	Non-alcoholic fatty liver disease	3	146	1.57	0.00055	9606.ENSP00000360266,9606.ENSP00000378974,9606.ENSP00000394560	JUN,MAPK8,MAPK9
KEGG	hsa04141	Protein processing in endoplasmic reticulum	3	163	1.52	0.00074	9606.ENSP00000378974,9606.ENSP00000381070,9606.ENSP00000394560	MAPK8,MAP2K7,MAPK9
KEGG	hsa04621	NOD-like receptor signaling pathway	3	173	1.49	0.00086	9606.ENSP00000360266,9606.ENSP00000378974,9606.ENSP00000394560	JUN,MAPK8,MAPK9
KEGG	hsa05203	Viral carcinogenesis	3	183	1.47	0.00099	9606.ENSP00000264110,9606.ENSP00000269305,9606.ENSP00000360266	ATF2,TP53,JUN
KEGG	hsa04215	Apoptosis - multiple species	2	30	2.08	0.0010	9606.ENSP00000378974,9606.ENSP00000394560	MAPK8,MAPK9
KEGG	hsa051	Pathogenic Escherichia coli infection	3	187	1.4	0.001	9606.ENSP00000360266,9606.ENSP00000378974,9606.ENSP00000394560	JUN,MAPK8,MAPK9

	30				6	0		
KEGG	hsa05200	Pathways in cancer	4	515	1.14	0.0010	9606.ENSP00000269305,9606.ENSP00000360266,9606.ENSP00000378974,9606.ENSP00000394560	TP53,JUN,MAPK8,MAPK9
KEGG	hsa04510	Focal adhesion	3	195	1.44	0.0011	9606.ENSP00000360266,9606.ENSP00000378974,9606.ENSP00000394560	JUN,MAPK8,MAPK9
KEGG	hsa04024	cAMP signaling pathway	3	207	1.41	0.0013	9606.ENSP00000360266,9606.ENSP00000378974,9606.ENSP00000394560	JUN,MAPK8,MAPK9
KEGG	hsa05163	Human cytomegalovirus infection	3	217	1.39	0.0014	9606.ENSP00000264110,9606.ENSP00000269305,9606.ENSP00000300659	ATF2,TP53,NFATC3
KEGG	hsa05012	Parkinson disease	3	236	1.36	0.0018	9606.ENSP00000269305,9606.ENSP00000378974,9606.ENSP00000394560	TP53,MAPK8,MAPK9
KEGG	hsa04930	Type II diabetes mellitus	2	45	1.9	0.0020	9606.ENSP00000378974,9606.ENSP00000394560	MAPK8,MAPK9
KEGG	hsa05020	Prion disease	3	263	1.31	0.0023	9606.ENSP00000264110,9606.ENSP00000378974,9606.ENSP00000394560	ATF2,MAPK8,MAPK9
KEGG	hsa05030	Cocaine addiction	2	49	1.86	0.0023	9606.ENSP00000264110,9606.ENSP00000360266	ATF2,JUN
KEGG	hsa05031	Amphetamine addiction	2	65	1.74	0.0037	9606.ENSP00000264110,9606.ENSP00000360266	ATF2,JUN
KEGG	hsa04622	RIG-I-like receptor signaling pathway	2	69	1.72	0.0040	9606.ENSP00000378974,9606.ENSP00000394560	MAPK8,MAPK9
KEGG	hsa04917	Prolactin signaling pathway	2	68	1.72	0.0040	9606.ENSP00000378974,9606.ENSP00000394560	MAPK8,MAPK9
KEGG	hsa04920	Adipocytokine signaling pathway	2	68	1.72	0.0040	9606.ENSP00000378974,9606.ENSP00000394560	MAPK8,MAPK9
KEGG	hsa046	B cell receptor signaling pathway	2	78	1.6	0.005	9606.ENSP00000300659,9606.ENSP00000360266	NFATC3,JUN

	62				6	0		
KEGG	hsa05010	Alzheimer disease	3	354	1.18	0.0050	9606.ENSP00000378974,9606.ENSP00000381070,9606.ENSP00000394560	MAPK8,MAP2K7,MAPK9
KEGG	hsa04211	Longevity regulating pathway	2	87	1.61	0.059	9606.ENSP00000264110,9606.ENSP00000269305	ATF2,TP53
KEGG	hsa05235	PD-L1 expression and PD-1 checkpoint pathway in cancer	2	87	1.61	0.059	9606.ENSP00000300659,9606.ENSP00000360266	NFATC3,JUN
KEGG	hsa04750	Inflammatory mediator regulation of TRP channels	2	92	1.59	0.064	9606.ENSP00000378974,9606.ENSP00000394560	MAPK8,MAPK9
KEGG	hsa04914	Progesterone-mediated oocyte maturation	2	95	1.58	0.067	9606.ENSP00000378974,9606.ENSP00000394560	MAPK8,MAPK9
KEGG	hsa05145	Toxoplasmosis	2	103	1.54	0.077	9606.ENSP00000378974,9606.ENSP00000394560	MAPK8,MAPK9
KEGG	hsa04928	Parathyroid hormone synthesis, secretion and action	2	104	1.54	0.078	9606.ENSP00000252818,9606.ENSP00000264110	JUND,ATF2
KEGG	hsa04931	Insulin resistance	2	106	1.53	0.079	9606.ENSP00000378974,9606.ENSP00000394560	MAPK8,MAPK9
KEGG	hsa04068	FoxO signaling pathway	2	126	1.45	0.109	9606.ENSP00000378974,9606.ENSP00000394560	MAPK8,MAPK9
KEGG	hsa04140	Autophagy - animal	2	131	1.44	0.116	9606.ENSP00000378974,9606.ENSP00000394560	MAPK8,MAPK9
KEGG	hsa04910	Insulin signaling pathway	2	132	1.43	0.116	9606.ENSP00000378974,9606.ENSP00000394560	MAPK8,MAPK9
KEGG	hsa04915	Estrogen signaling pathway	2	133	1.43	0.116	9606.ENSP00000264110,9606.ENSP00000360266	ATF2,JUN
KEGG	hsa050	Spinocerebellar ataxia	2	135	1.4	0.011	9606.ENSP00000378974,9606.ENSP00000394560	MAPK8,MAPK9

	17				2	8		
KEGG	hsa04723	Retrograde endocannabinoid signaling	2	142	1.4	0.0129	9606.ENSP00000378974,9606.ENSP00000394560	MAPK8,MAPK9
KEGG	hsa04217	Necroptosis	2	147	1.39	0.0134	9606.ENSP00000378974,9606.ENSP00000394560	MAPK8,MAPK9
KEGG	hsa04921	Oxytocin signaling pathway	2	147	1.39	0.0134	9606.ENSP00000300659,9606.ENSP00000360266	NFATC3,JUN
KEGG	hsa05224	Breast cancer	2	146	1.39	0.0134	9606.ENSP00000269305,9606.ENSP00000360266	TP53,JUN
KEGG	hsa04218	Cellular senescence	2	150	1.38	0.0135	9606.ENSP00000269305,9606.ENSP00000300659	TP53,NFATC3
KEGG	hsa04022	cGMP-PKG signaling pathway	2	163	1.34	0.0157	9606.ENSP00000264110,9606.ENSP00000300659	ATF2,NFATC3
KEGG	hsa05152	Tuberculosis	2	165	1.34	0.0159	9606.ENSP00000378974,9606.ENSP00000394560	MAPK8,MAPK9
KEGG	hsa04014	Ras signaling pathway	2	225	1.2	0.0285	9606.ENSP00000378974,9606.ENSP00000394560	MAPK8,MAPK9
Reactome	HSA-166058	MyD88:MAL(TIRAP) cascade initiated on plasma membrane	7	109	2.06	5.18e-11	9606.ENSP00000264110,9606.ENSP00000269305,9606.ENSP00000360266,9606.ENSP00000378974,9606.ENSP00000381070,9606.ENSP00000394560,9606.ENSP00000410402	ATF2,TP53,JUN,MAPK8,MAP2K7,MAPK9,MAP2K4
Reactome	HSA-168164	Toll Like Receptor 3 (TLR3) Cascade	7	100	2.1	5.18e-11	9606.ENSP00000264110,9606.ENSP00000269305,9606.ENSP00000360266,9606.ENSP00000378974,9606.ENSP00000381070,9606.ENSP00000394560,9606.ENSP00000410402	ATF2,TP53,JUN,MAPK8,MAP2K7,MAPK9,MAP2K4
Reactome	HSA-937061	TRIF(TICAM1)-mediated TLR4 signaling	7	104	2.08	5.18e-11	9606.ENSP00000264110,9606.ENSP00000269305,9606.ENSP00000360266,9606.ENSP00000378974,9606.ENSP00000381070,9606.ENSP00000394560,9606.ENSP00000410402	ATF2,TP53,JUN,MAPK8,MAP2K7,MAPK9,MAP2K4
Reacto	HSA-	TRAF6 mediated induction of NFkB and MAP kinases upon TLR7/8 or 9	7	98	2.1	5.18e	9606.ENSP00000264110,9606.ENSP00000269305,9606.ENSP00000360266,9606.ENSP00000378974,9606.ENSP000003945	ATF2,TP53,JUN,MAPK8,MAP2K7,MAPK9,M

me	975138	activation			1	-11	60,9606.ENSP00000410402	AP2K4
Reactome	HSA-975871	MyD88 cascade initiated on plasma membrane	7	93	2.13	5.18e-11	9606.ENSP00000264110,9606.ENSP00000269305,9606.ENSP00000360266,9606.ENSP00000378974,9606.ENSP00000381070,9606.ENSP00000394560,9606.ENSP00000410402	ATF2,TP53,JUN,MAPK8,MAP2K7,MAPK9,MAP2K4
Reactome	HSA-450294	MAP kinase activation	6	61	2.25	7.98e-11	9606.ENSP00000264110,9606.ENSP00000360266,9606.ENSP00000378974,9606.ENSP00000381070,9606.ENSP00000394560,9606.ENSP00000410402	ATF2,JUN,MAPK8,MAP2K7,MAPK9,MAP2K4
Reactome	HSA-2559580	Oxidative Stress Induced Senescence	6	92	2.07	7.04e-10	9606.ENSP00000269305,9606.ENSP00000360266,9606.ENSP00000378974,9606.ENSP00000381070,9606.ENSP00000394560,9606.ENSP00000410402	TP53,JUN,MAPK8,MAP2K7,MAPK9,MAP2K4
Reactome	HSA-2871796	FCERI mediated MAPK activation	5	34	2.42	1.21e-09	9606.ENSP00000360266,9606.ENSP00000378974,9606.ENSP00000381070,9606.ENSP00000394560,9606.ENSP00000410402	JUN,MAPK8,MAP2K7,MAPK9,MAP2K4
Reactome	HSA-450341	Activation of the AP-1 family of transcription factors	4	9	2.99	4.07e-09	9606.ENSP00000264110,9606.ENSP00000360266,9606.ENSP00000378974,9606.ENSP00000394560	ATF2,JUN,MAPK8,MAPK9
Reactome	HSA-2454202	Fc epsilon receptor (FCERI) signaling	6	131	1.91	4.71e-09	9606.ENSP00000300659,9606.ENSP00000360266,9606.ENSP00000378974,9606.ENSP00000381070,9606.ENSP00000394560,9606.ENSP00000410402	NFATC3,JUN,MAPK8,MAP2K7,MAPK9,MAP2K4
Reactome	HSA-450321	JNK (c-Jun kinases) phosphorylation and activation mediated by activated human TAK1	4	20	2.55	5.28e-08	9606.ENSP00000378974,9606.ENSP00000381070,9606.ENSP00000394560,9606.ENSP00000410402	MAPK8,MAP2K7,MAPK9,MAP2K4
Reactome	HSA-449147	Signaling by Interleukins	7	453	1.44	1.00e-07	9606.ENSP00000264110,9606.ENSP00000269305,9606.ENSP00000360266,9606.ENSP00000378974,9606.ENSP00000381070,9606.ENSP00000394560,9606.ENSP00000410402	ATF2,TP53,JUN,MAPK8,MAP2K7,MAPK9,MAP2K4
Reacto	HSA-	Innate Immune System	8	1041	1.1	7.58e	9606.ENSP00000264110,9606.ENSP00000269305,9606.ENSP00000300659,9606.ENSP00000360266,9606.ENSP00000378974,9606.ENSP000003810	ATF2,TP53,NFATC3,JUN,MAPK8,MAP2K7,

me	168249				4	-07	70,9606.ENSP00000394560,9606.ENSP00000410402	MAPK9,MAP2K4
Reactome	HSA-2262752	Cellular responses to stress	7	747	1.22	2.65e-06	9606.ENSP00000264110,9606.ENSP00000269305,9606.ENSP00000360266,9606.ENSP00000378974,9606.ENSP00000381070,9606.ENSP00000394560,9606.ENSP00000410402	ATF2,TP53,JUN,MAPK8,MAP2K7,MAPK9,MAP2K4
Reactome	HSA-8943724	Regulation of PTEN gene transcription	3	60	1.95	0.00036	9606.ENSP00000264110,9606.ENSP00000269305,9606.ENSP00000360266	ATF2,TP53,JUN
Reactome	HSA-6796648	TP53 Regulates Transcription of DNA Repair Genes	3	62	1.94	0.00038	9606.ENSP00000264110,9606.ENSP00000269305,9606.ENSP00000360266	ATF2,TP53,JUN
Reactome	HSA-5210891	Uptake and function of anthrax toxins	2	11	2.51	0.0015	9606.ENSP00000381070,9606.ENSP00000410402	MAP2K7,MAP2K4
Reactome	HSA-9673324	WNT5:FZD7-mediated leishmania damping	2	12	2.47	0.0017	9606.ENSP00000360266,9606.ENSP00000378974	JUN,MAPK8
Reactome	HSA-9018519	Estrogen-dependent gene expression	3	119	1.65	0.0023	9606.ENSP00000252818,9606.ENSP00000264110,9606.ENSP00000360266	JUND,ATF2,JUN
Reactome	HSA-446652	Interleukin-1 family signaling	3	149	1.56	0.0042	9606.ENSP00000269305,9606.ENSP00000378974,9606.ENSP00000410402	TP53,MAPK8,MAP2K4
Reactome	HSA-566	Infectious disease	5	917	0.99	0.0046	9606.ENSP00000298573,9606.ENSP00000360266,9606.ENSP00000378974,9606.ENSP00000381070,9606.ENSP00000410402	DUSP16,JUN,MAPK8,MAP2K7,MAP2K4

	3205							
Reactome	HSA-9734009	Defective Intrinsic Pathway for Apoptosis	2	24	2.17	0.0051	9606.ENSP00000269305,9606.ENSP00000360266	TP53,JUN
Reactome	HSA-114452	Activation of BH3-only proteins	2	29	2.09	0.0071	9606.ENSP00000269305,9606.ENSP00000378974	TP53,MAPK8
Reactome	HSA-1643685	Disease	6	1702	0.8	0.0071	9606.ENSP00000269305,9606.ENSP00000298573,9606.ENSP00000360266,9606.ENSP00000378974,9606.ENSP00000381070,9606.ENSP00000410402	TP53,DUSP16,JUN,MAPK8,MAP2K7,MAP2K4
Reactome	HSA-9031628	NGF-stimulated transcription	2	39	1.96	0.0114	9606.ENSP00000252818,9606.ENSP00000264110	JUND,ATF2
Reactome	HSA-5693565	Recruitment and ATM-mediated phosphorylation of repair and signaling proteins at DNA double strand breaks	2	60	1.78	0.0240	9606.ENSP00000269305,9606.ENSP00000378974	TP53,MAPK8
Reactome	HSA-1912408	Pre-NOTCH Transcription and Translation	2	62	1.76	0.0243	9606.ENSP00000269305,9606.ENSP00000360266	TP53,JUN
Reactome	HSA-162582	Signal Transduction	6	2540	0.63	0.0470	9606.ENSP00000252818,9606.ENSP00000264110,9606.ENSP00000269305,9606.ENSP00000298573,9606.ENSP00000360266,9606.ENSP00000378974	JUND,ATF2,TP53,DUSP16,JUN,MAPK8

Table 5. Pathway Enrichment Analysis for GO, KEGG, STRING clusters and Reactome Terms within MAP2K3 gene

#category	term ID	term description	observed gene count	background gene count	strength	false discovery rate	matching proteins in your network (IDs)	matching proteins in your network (labels)
GO Process	GO:0051403	Stress-activated MAPK cascade	9	75	2.33	2.55e-16	9606.ENSP00000215832,9606.ENSP00000229795,9606.ENSP00000263025,9606.ENSP00000310094,9606.ENSP00000333685,9606.ENSP00000345083,9606.ENSP00000351908,9606.ENSP00000370503,9606.ENSP00000468348	MAPK1,MAPK14,MAPK3,TAOK2,MAPK11,MAP2K3,MAP3K5,CCM2,MAP2K6
GO Process	GO:0000165	MAPK cascade	10	219	1.91	2.10e-15	9606.ENSP00000215832,9606.ENSP00000229795,9606.ENSP00000263025,9606.ENSP00000310094,9606.ENSP00000333685,9606.ENSP00000345083,9606.ENSP00000351908,9606.ENSP00000370503,9606.ENSP00000375986,9606.ENSP00000468348	MAPK1,MAPK14,MAPK3,TAOK2,MAPK11,MAP2K3,MAP3K5,CCM2,MAP3K4,MAP2K6
GO Process	GO:0038066	p38MAPK cascade	5	13	2.84	6.24e-10	9606.ENSP00000229795,9606.ENSP00000333685,9606.ENSP00000345083,9606.ENSP00000351908,9606.ENSP00000468348	MAPK14,MAPK11,MAP2K3,MAP3K5,MAP2K6
GO Process	GO:0016310	Phosphorylation	10	966	1.27	2.80e-09	9606.ENSP00000215832,9606.ENSP00000216160,9606.ENSP00000229795,9606.ENSP00000263025,9606.ENSP00000310094,9606.ENSP00000333685,9606.ENSP00000345083,9606.ENSP00000351908,9606.ENSP00000375986,9606.ENSP00000468348	MAPK1,TAB1,MAPK14,MAPK3,TAOK2,MAPK11,MAP2K3,MAP3K5,MAP3K4,MAP2K6
GO Process	GO:0006468	Protein phosphorylation	9	736	1.34	2.02e-08	9606.ENSP00000215832,9606.ENSP00000229795,9606.ENSP00000263025,9606.ENSP00000310094,9606.ENSP00000333685,9606.ENSP00000345083,9606.ENSP00000351908,9606.ENSP00000375986,9606.ENSP00000468348	MAPK1,MAPK14,MAPK3,TAOK2,MAPK11,MAP2K3,MAP3K5,MAP3K4,MAP2K6

GO Process	GO:0051347	Positive regulation of transferase activity	8	586	1.39	1.94e-07	9606.ENSP00000215832,9606.ENSP00000216160,9606.ENSP00000263025,9606.ENSP00000310094,9606.ENSP00000345083,9606.ENSP00000351908,9606.ENSP00000375986,9606.ENSP00000468348	MAPK1,TAB1,MAPK3,TAOK2,MAP2K3,MAP3K5,MAP3K4,MAP2K6
GO Process	GO:0090398	Cellular senescence	5	60	2.17	2.64e-07	9606.ENSP00000229795,9606.ENSP00000333685,9606.ENSP00000345083,9606.ENSP00000351908,9606.ENSP00000468348	MAPK14,MAPK11,MAP2K3,MAP3K5,MAP2K6
GO Process	GO:0043408	Regulation of MAPK cascade	8	668	1.33	4.34e-07	9606.ENSP00000215832,9606.ENSP00000216160,9606.ENSP00000263025,9606.ENSP00000310094,9606.ENSP00000345083,9606.ENSP00000351908,9606.ENSP00000375986,9606.ENSP00000468348	MAPK1,TAB1,MAPK3,TAOK2,MAP2K3,MAP3K5,MAP3K4,MAP2K6
GO Process	GO:0043085	Positive regulation of catalytic activity	9	1191	1.13	7.83e-07	9606.ENSP00000215832,9606.ENSP00000216160,9606.ENSP00000229795,9606.ENSP00000263025,9606.ENSP00000310094,9606.ENSP00000345083,9606.ENSP00000351908,9606.ENSP00000375986,9606.ENSP00000468348	MAPK1,TAB1,MAPK14,MAPK3,TAOK2,MAP2K3,MAP3K5,MAP3K4,MAP2K6
GO Process	GO:0001934	Positive regulation of protein phosphorylation	8	747	1.28	8.31e-07	9606.ENSP00000215832,9606.ENSP00000216160,9606.ENSP00000263025,9606.ENSP00000310094,9606.ENSP00000345083,9606.ENSP00000351908,9606.ENSP00000375986,9606.ENSP00000468348	MAPK1,TAB1,MAPK3,TAOK2,MAP2K3,MAP3K5,MAP3K4,MAP2K6
GO Process	GO:0043410	Positive regulation of MAPK cascade	7	481	1.42	1.62e-06	9606.ENSP00000216160,9606.ENSP00000263025,9606.ENSP00000310094,9606.ENSP00000345083,9606.ENSP00000351908,9606.ENSP00000375986,9606.ENSP00000468348	TAB1,MAPK3,TAOK2,MAP2K3,MAP3K5,MAP3K4,MAP2K6
GO Process	GO:1902531	Regulation of intracellular signal transduction	9	1726	0.97	9.91e-06	9606.ENSP00000215832,9606.ENSP00000216160,9606.ENSP00000229795,9606.ENSP00000263025,9606.ENSP00000310094,9606.ENSP00000345083,9606.ENSP00000351908,9606.ENSP00000375986,9606.ENSP00000468348	MAPK1,TAB1,MAPK14,MAPK3,TAOK2,MAP2K3,MAP3K5,MAP3K4,MAP2K6
GO Process	GO:0036211	Protein modification process	10	2674	0.83	1.31e-05	9606.ENSP00000215832,9606.ENSP00000216160,9606.ENSP00000229795,9606.ENSP00000263025,9606.ENSP00000310094,9606.ENSP00000333685,9606.ENSP00000345083,9606.ENSP00000351908,9606.ENSP00000375986,9606.ENSP00000468348	MAPK1,TAB1,MAPK14,MAPK3,TAOK2,MAPK11,MAP2K3,MAP3K5,MAP3K4,MAP2K6
GO Process	GO:0032872	Regulation of stress-activated MAPK cascade	5	189	1.68	2.27e-05	9606.ENSP00000215832,9606.ENSP00000263025,9606.ENSP00000310094,9606.ENSP00000351908,9606.ENSP00000375986	MAPK1,MAPK3,TAOK2,MAP3K5,MAP3K4
GO Process	GO:0045860	Positive regulation of protein kinase activity	6	416	1.41	2.27e-05	9606.ENSP00000216160,9606.ENSP00000310094,9606.ENSP00000345083,9606.ENSP00000351908,9606.ENSP00000375986,9606.ENSP00000468348	TAB1,TAOK2,MAP2K3,MAP3K5,MAP3K4,MAP2K6
GO	GO:0071902	Positive	5	226	1.6	4.67e-	9606.ENSP00000216160,9606.ENSP00000310094,9606.EN	TAB1,TAOK2,MAP3K5,

Process		regulation of protein serine/threonine kinase activity				05	SP00000351908,9606.ENSP00000375986,9606.ENSP00000468348	MAP3K4,MAP2K6
GO Process	GO:0072359	Circulatory system development	7	901	1.14	5.41e-05	9606.ENSP00000215832,9606.ENSP00000216160,9606.ENSP00000229795,9606.ENSP00000263025,9606.ENSP00000333685,9606.ENSP00000345083,9606.ENSP00000370503	MAPK1,TAB1,MAPK14,MAPK3,MAPK11,MAP2K3,CCM2
GO Process	GO:0007165	Signal transduction	11	4714	0.62	6.39e-05	9606.ENSP00000215832,9606.ENSP00000216160,9606.ENSP00000229795,9606.ENSP00000263025,9606.ENSP00000310094,9606.ENSP00000333685,9606.ENSP00000345083,9606.ENSP00000351908,9606.ENSP00000370503,9606.ENSP00000375986,9606.ENSP00000468348	MAPK1,TAB1,MAPK14,MAPK3,TAOK2,MAPK11,MAP2K3,MAP3K5,CCM2,MAP3K4,MAP2K6
GO Process	GO:1904355	Positive regulation of telomere capping	3	17	2.5	6.39e-05	9606.ENSP00000215832,9606.ENSP00000263025,9606.ENSP00000375986	MAPK1,MAPK3,MAP3K4
GO Process	GO:0007507	Heart development	6	548	1.29	7.91e-05	9606.ENSP00000215832,9606.ENSP00000216160,9606.ENSP00000263025,9606.ENSP00000333685,9606.ENSP00000345083,9606.ENSP00000370503	MAPK1,TAB1,MAPK3,MAPK11,MAP2K3,CCM2
GO Process	GO:0010604	Positive regulation of macromolecule metabolic process	10	3533	0.7	0.00012	9606.ENSP00000215832,9606.ENSP00000216160,9606.ENSP00000229795,9606.ENSP00000263025,9606.ENSP00000310094,9606.ENSP00000333685,9606.ENSP00000345083,9606.ENSP00000351908,9606.ENSP00000375986,9606.ENSP00000468348	MAPK1,TAB1,MAPK14,MAPK3,TAOK2,MAPK11,MAP2K3,MAP3K5,MAP3K4,MAP2K6
GO Process	GO:0010647	Positive regulation of cell communication	8	1693	0.93	0.00014	9606.ENSP00000215832,9606.ENSP00000216160,9606.ENSP00000263025,9606.ENSP00000310094,9606.ENSP00000345083,9606.ENSP00000351908,9606.ENSP00000375986,9606.ENSP00000468348	MAPK1,TAB1,MAPK3,TAOK2,MAP2K3,MAP3K5,MAP3K4,MAP2K6
GO Process	GO:0023056	Positive regulation of signaling	8	1698	0.93	0.00014	9606.ENSP00000215832,9606.ENSP00000216160,9606.ENSP00000263025,9606.ENSP00000310094,9606.ENSP00000345083,9606.ENSP00000351908,9606.ENSP00000375986,9606.ENSP00000468348	MAPK1,TAB1,MAPK3,TAOK2,MAP2K3,MAP3K5,MAP3K4,MAP2K6
GO Process	GO:0043406	Positive regulation of MAP kinase activity	4	116	1.79	0.00014	9606.ENSP00000216160,9606.ENSP00000351908,9606.ENSP00000375986,9606.ENSP00000468348	TAB1,MAP3K5,MAP3K4,MAP2K6
GO Process	GO:0048731	System development	10	3867	0.67	0.00023	9606.ENSP00000215832,9606.ENSP00000216160,9606.ENSP00000229795,9606.ENSP00000263025,9606.ENSP00000310094,9606.ENSP00000333685,9606.ENSP00000345083,	MAPK1,TAB1,MAPK14,MAPK3,TAOK2,MAPK11,MAP2K3,CCM2,MAP3

							9606.ENSP00000370503,9606.ENSP00000375986,9606.ENSP00000468348	K4,MAP2K6
GO Process	GO:0032212	Positive regulation of telomere maintenance via telomerase	3	34	2.2	0.00029	9606.ENSP00000215832,9606.ENSP00000263025,9606.ENSP00000375986	MAPK1,MAPK3,MAP3K4
GO Process	GO:0051973	Positive regulation of telomerase activity	3	34	2.2	0.00029	9606.ENSP00000215832,9606.ENSP00000263025,9606.ENSP00000375986	MAPK1,MAPK3,MAP3K4
GO Process	GO:0031663	Lipopolysaccharide-mediated signaling pathway	3	39	2.14	0.00040	9606.ENSP00000215832,9606.ENSP00000229795,9606.ENSP00000263025	MAPK1,MAPK14,MAPK3
GO Process	GO:0071310	Cellular response to organic substance	8	2019	0.85	0.00040	9606.ENSP00000215832,9606.ENSP00000216160,9606.ENSP00000229795,9606.ENSP00000263025,9606.ENSP00000333685,9606.ENSP00000345083,9606.ENSP00000351908,9606.ENSP00000468348	MAPK1,TAB1,MAPK14,MAPK3,MAPK11,MAP2K3,MAP3K5,MAP2K6
GO Process	GO:0071356	Cellular response to tumor necrosis factor	4	175	1.61	0.00051	9606.ENSP00000215832,9606.ENSP00000229795,9606.ENSP00000263025,9606.ENSP00000351908	MAPK1,MAPK14,MAPK3,MAP3K5
GO Process	GO:0048584	Positive regulation of response to stimulus	8	2131	0.83	0.00057	9606.ENSP00000215832,9606.ENSP00000216160,9606.ENSP00000263025,9606.ENSP00000310094,9606.ENSP00000345083,9606.ENSP00000351908,9606.ENSP00000375986,9606.ENSP00000468348	MAPK1,TAB1,MAPK3,TAOK2,MAP2K3,MAP3K5,MAP3K4,MAP2K6
GO Process	GO:0031325	Positive regulation of cellular metabolic process	9	3114	0.71	0.00060	9606.ENSP00000215832,9606.ENSP00000216160,9606.ENSP00000229795,9606.ENSP00000263025,9606.ENSP00000310094,9606.ENSP00000345083,9606.ENSP00000351908,9606.ENSP00000375986,9606.ENSP00000468348	MAPK1,TAB1,MAPK14,MAPK3,TAOK2,MAP2K3,MAP3K5,MAP3K4,MAP2K6
GO Process	GO:0034198	Cellular response to amino acid starvation	3	49	2.04	0.00065	9606.ENSP00000215832,9606.ENSP00000263025,9606.ENSP00000351908	MAPK1,MAPK3,MAP3K5
GO Process	GO:0051173	Positive regulation of nitrogen	9	3166	0.71	0.00066	9606.ENSP00000215832,9606.ENSP00000216160,9606.ENSP00000229795,9606.ENSP00000263025,9606.ENSP00000310094,9606.ENSP00000345083,9606.ENSP00000351908,	MAPK1,TAB1,MAPK14,MAPK3,TAOK2,MAP2K3,MAP3K5,MAP3K4,MA

		compound metabolic process					9606.ENSP00000375986,9606.ENSP00000468348	P2K6
GO Process	GO:0071222	Cellular response to lipopolysaccharide	4	195	1.57	0.00071	9606.ENSP00000215832,9606.ENSP00000229795,9606.ENSP00000263025,9606.ENSP00000345083	MAPK1,MAPK14,MAPK3,MAP2K3
GO Process	GO:0048513	Animal organ development	9	3246	0.7	0.00077	9606.ENSP00000215832,9606.ENSP00000216160,9606.ENSP00000229795,9606.ENSP00000263025,9606.ENSP00000333685,9606.ENSP00000345083,9606.ENSP00000370503,9606.ENSP00000375986,9606.ENSP00000468348	MAPK1,TAB1,MAPK14,MAPK3,MAPK11,MAP2K3,CCM2,MAP3K4,MAP2K6
GO Process	GO:0002931	Response to ischemia	3	55	1.99	0.00081	9606.ENSP00000345083,9606.ENSP00000351908,9606.ENSP00000468348	MAP2K3,MAP3K5,MAP2K6
GO Process	GO:0090170	Regulation of Golgi inheritance	2	4	2.95	0.00084	9606.ENSP00000215832,9606.ENSP00000263025	MAPK1,MAPK3
GO Process	GO:0070848	Response to growth factor	5	503	1.25	0.00088	9606.ENSP00000215832,9606.ENSP00000216160,9606.ENSP00000229795,9606.ENSP00000263025,9606.ENSP00000345083	MAPK1,TAB1,MAPK14,MAPK3,MAP2K3
GO Process	GO:0072584	Caveolin-mediated endocytosis	2	5	2.86	0.0011	9606.ENSP00000215832,9606.ENSP00000263025	MAPK1,MAPK3
GO Process	GO:0072709	Cellular response to sorbitol	2	5	2.86	0.0011	9606.ENSP00000345083,9606.ENSP00000468348	MAP2K3,MAP2K6
GO Process	GO:0120041	Positive regulation of macrophage proliferation	2	5	2.86	0.0011	9606.ENSP00000215832,9606.ENSP00000263025	MAPK1,MAPK3
GO Process	GO:0006915	Apoptotic process	6	1041	1.01	0.0014	9606.ENSP00000215832,9606.ENSP00000229795,9606.ENSP00000263025,9606.ENSP00000310094,9606.ENSP00000351908,9606.ENSP00000468348	MAPK1,MAPK14,MAPK3,TAOK2,MAP3K5,MAP2K6
GO Process	GO:1901701	Cellular response to oxygen-containing compound	6	1057	1.01	0.0015	9606.ENSP00000215832,9606.ENSP00000229795,9606.ENSP00000263025,9606.ENSP00000345083,9606.ENSP00000351908,9606.ENSP00000468348	MAPK1,MAPK14,MAPK3,MAP2K3,MAP3K5,MAP2K6
GO	GO:0060440	Trachea	2	7	2.71	0.0017	9606.ENSP00000215832,9606.ENSP00000263025	MAPK1,MAPK3

Process		formation						
GO Process	GO:0006975	DNA damage induced protein phosphorylation	2	8	2.65	0.0019	9606.ENSP00000263025,9606.ENSP00000468348	MAPK3,MAP2K6
GO Process	GO:0042473	Outer ear morphogenesis	2	8	2.65	0.0019	9606.ENSP00000215832,9606.ENSP00000263025	MAPK1,MAPK3
GO Process	GO:0010557	Positive regulation of macromolecule biosynthetic process	7	1935	0.81	0.0030	9606.ENSP00000215832,9606.ENSP00000229795,9606.ENSP00000263025,9606.ENSP00000345083,9606.ENSP00000351908,9606.ENSP00000375986,9606.ENSP00000468348	MAPK1,MAPK14,MAPK3,MAP2K3,MAP3K5,MAP3K4,MAP2K6
GO Process	GO:0060020	Bergmann glial cell differentiation	2	11	2.51	0.0032	9606.ENSP00000215832,9606.ENSP00000263025	MAPK1,MAPK3
GO Process	GO:0071345	Cellular response to cytokine stimulus	5	711	1.1	0.0034	9606.ENSP00000215832,9606.ENSP00000229795,9606.ENSP00000263025,9606.ENSP00000333685,9606.ENSP00000351908	MAPK1,MAPK14,MAPK3,MAPK11,MAP3K5
GO Process	GO:0061308	Cardiac neural crest cell development involved in heart development	2	12	2.47	0.0035	9606.ENSP00000215832,9606.ENSP00000263025	MAPK1,MAPK3
GO Process	GO:0048522	Positive regulation of cellular process	10	5584	0.51	0.0037	9606.ENSP00000215832,9606.ENSP00000216160,9606.ENSP00000229795,9606.ENSP00000263025,9606.ENSP00000310094,9606.ENSP00000333685,9606.ENSP00000345083,9606.ENSP00000351908,9606.ENSP00000375986,9606.ENSP00000468348	MAPK1,TAB1,MAPK14,MAPK3,TAOK2,MAPK11,MAP2K3,MAP3K5,MAP3K4,MAP2K6
GO Process	GO:0006974	Cellular response to DNA damage stimulus	5	744	1.08	0.0039	9606.ENSP00000215832,9606.ENSP00000229795,9606.ENSP00000263025,9606.ENSP00000310094,9606.ENSP00000468348	MAPK1,MAPK14,MAPK3,TAOK2,MAP2K6
GO Process	GO:0035331	Negative regulation of hippo signaling	2	13	2.44	0.0039	9606.ENSP00000229795,9606.ENSP00000345083	MAPK14,MAP2K3
GO Proces	GO:0051707	Response to other organism	6	1328	0.91	0.0042	9606.ENSP00000215832,9606.ENSP00000229795,9606.ENSP00000263025,9606.ENSP00000333685,9606.ENSP00000	MAPK1,MAPK14,MAPK3,MAPK11,MAP2K3,M

s							345083,9606.ENSP00000351908	AP3K5
GO Process	GO:0034614	Cellular response to reactive oxygen species	3	118	1.66	0.0045	9606.ENSP00000215832,9606.ENSP00000263025,9606.ENSP00000351908	MAPK1,MAPK3,MAP3K5
GO Process	GO:0030278	Regulation of ossification	3	120	1.65	0.0047	9606.ENSP00000215832,9606.ENSP00000229795,9606.ENSP00000263025	MAPK1,MAPK14,MAPK3
GO Process	GO:0080134	Regulation of response to stress	6	1373	0.89	0.0048	9606.ENSP00000215832,9606.ENSP00000229795,9606.ENSP00000263025,9606.ENSP00000310094,9606.ENSP00000351908,9606.ENSP00000375986	MAPK1,MAPK14,MAPK3,TAOK2,MAP3K5,MAP3K4
GO Process	GO:0032874	Positive regulation of stress-activated MAPK cascade	3	126	1.63	0.0051	9606.ENSP00000310094,9606.ENSP00000351908,9606.ENSP00000375986	TAOK2,MAP3K5,MAP3K4
GO Process	GO:0051770	Positive regulation of nitric-oxide synthase biosynthetic process	2	18	2.3	0.0062	9606.ENSP00000345083,9606.ENSP00000468348	MAP2K3,MAP2K6
GO Process	GO:2000641	Regulation of early endosome to late endosome transport	2	18	2.3	0.0062	9606.ENSP00000215832,9606.ENSP00000263025	MAPK1,MAPK3
GO Process	GO:0010759	Positive regulation of macrophage chemotaxis	2	19	2.28	0.0067	9606.ENSP00000215832,9606.ENSP00000263025	MAPK1,MAPK3
GO Process	GO:0001649	Osteoblast differentiation	3	141	1.58	0.0068	9606.ENSP00000229795,9606.ENSP00000333685,9606.ENSP00000468348	MAPK14,MAPK11,MAP2K6
GO Process	GO:0035295	Tube development	5	880	1.01	0.0073	9606.ENSP00000215832,9606.ENSP00000216160,9606.ENSP00000229795,9606.ENSP00000263025,9606.ENSP00000370503	MAPK1,TAB1,MAPK14,MAPK3,CCM2
GO Process	GO:0001890	Placenta development	3	149	1.56	0.0078	9606.ENSP00000215832,9606.ENSP00000229795,9606.ENSP00000375986	MAPK1,MAPK14,MAP3K4

GO Process	GO:0018193	Peptidyl-amino acid modification	5	900	1.0	0.0080	9606.ENSP00000215832,9606.ENSP00000229795,9606.ENSP00000263025,9606.ENSP00000345083,9606.ENSP00000468348	MAPK1,MAPK14,MAPK3,MAP2K3,MAP2K6
GO Process	GO:0031667	Response to nutrient levels	4	461	1.19	0.0093	9606.ENSP00000215832,9606.ENSP00000229795,9606.ENSP00000263025,9606.ENSP00000351908	MAPK1,MAPK14,MAPK3,MAP3K5
GO Process	GO:0071363	Cellular response to growth factor stimulus	4	473	1.18	0.0101	9606.ENSP00000216160,9606.ENSP00000229795,9606.ENSP00000263025,9606.ENSP00000345083	TAB1,MAPK14,MAPK3,MAP2K3
GO Process	GO:0030154	Cell differentiation	8	3507	0.61	0.0103	9606.ENSP00000215832,9606.ENSP00000229795,9606.ENSP00000263025,9606.ENSP00000310094,9606.ENSP00000333685,9606.ENSP00000370503,9606.ENSP00000375986,9606.ENSP00000468348	MAPK1,MAPK14,MAPK3,TAOK2,MAPK11,CCM2,MAP3K4,MAP2K6
GO Process	GO:0018108	Peptidyl-tyrosine phosphorylation	3	176	1.48	0.0114	9606.ENSP00000263025,9606.ENSP00000345083,9606.ENSP00000468348	MAPK3,MAP2K3,MAP2K6
GO Process	GO:1900745	Positive regulation of p38MAPK cascade	2	27	2.12	0.0114	9606.ENSP00000351908,9606.ENSP00000375986	MAP3K5,MAP3K4
GO Process	GO:0030324	Lung development	3	180	1.47	0.0118	9606.ENSP00000215832,9606.ENSP00000216160,9606.ENSP00000263025	MAPK1,TAB1,MAPK3
GO Process	GO:0030878	Thyroid gland development	2	28	2.11	0.0118	9606.ENSP00000215832,9606.ENSP00000263025	MAPK1,MAPK3
GO Process	GO:0001568	Blood vessel development	4	505	1.15	0.0119	9606.ENSP00000215832,9606.ENSP00000216160,9606.ENSP00000229795,9606.ENSP00000370503	MAPK1,TAB1,MAPK14,CCM2
GO Process	GO:0048863	Stem cell differentiation	3	183	1.47	0.0121	9606.ENSP00000215832,9606.ENSP00000229795,9606.ENSP00000263025	MAPK1,MAPK14,MAPK3
GO Process	GO:0001501	Skeletal system development	4	513	1.14	0.0122	9606.ENSP00000229795,9606.ENSP00000263025,9606.ENSP00000333685,9606.ENSP00000468348	MAPK14,MAPK3,MAPK11,MAP2K6
GO Process	GO:0007178	Transmembrane receptor protein serine/threonine	3	206	1.42	0.0162	9606.ENSP00000216160,9606.ENSP00000229795,9606.ENSP00000263025	TAB1,MAPK14,MAPK3

		kinase signaling pathway						
GO Process	GO:0071276	Cellular response to cadmium ion	2	36	2.0	0.0173	9606.ENSP00000215832,9606.ENSP00000263025	MAPK1,MAPK3
GO Process	GO:0051239	Regulation of multicellular organismal process	7	2749	0.66	0.0179	9606.ENSP00000215832,9606.ENSP00000229795,9606.ENSP00000263025,9606.ENSP00000333685,9606.ENSP00000345083,9606.ENSP00000370503,9606.ENSP00000468348	MAPK1,MAPK14,MAPK3,MAPK11,MAP2K3,CCM2,MAP2K6
GO Process	GO:0060348	Bone development	3	216	1.4	0.0182	9606.ENSP00000229795,9606.ENSP00000333685,9606.ENSP00000468348	MAPK14,MAPK11,MAP2K6
GO Process	GO:0060043	Regulation of cardiac muscle cell proliferation	2	39	1.96	0.0198	9606.ENSP00000229795,9606.ENSP00000333685	MAPK14,MAPK11
GO Process	GO:0043583	Ear development	3	229	1.37	0.0209	9606.ENSP00000215832,9606.ENSP00000263025,9606.ENSP00000370503	MAPK1,MAPK3,CCM2
GO Process	GO:0035924	Cellular response to vascular endothelial growth factor stimulus	2	42	1.93	0.0222	9606.ENSP00000229795,9606.ENSP00000345083	MAPK14,MAP2K3
GO Process	GO:0043507	Positive regulation of JUN kinase activity	2	42	1.93	0.0222	9606.ENSP00000351908,9606.ENSP00000375986	MAP3K5,MAP3K4
GO Process	GO:0032735	Positive regulation of interleukin-12 production	2	43	1.92	0.0228	9606.ENSP00000229795,9606.ENSP00000333685	MAPK14,MAPK11
GO Process	GO:0045663	Positive regulation of myoblast differentiation	2	45	1.9	0.0245	9606.ENSP00000229795,9606.ENSP00000351908	MAPK14,MAP3K5
GO Process	GO:0060425	Lung morphogenesis	2	45	1.9	0.0245	9606.ENSP00000215832,9606.ENSP00000263025	MAPK1,MAPK3

GO Process	GO:0007167	Enzyme-linked receptor protein signaling pathway	4	641	1.05	0.0251	9606.ENSP00000215832,9606.ENSP00000216160,9606.ENSP00000229795,9606.ENSP00000263025	MAPK1,TAB1,MAPK14,MAPK3
GO Process	GO:0070849	Response to epidermal growth factor	2	46	1.89	0.0251	9606.ENSP00000215832,9606.ENSP00000263025	MAPK1,MAPK3
GO Process	GO:0048538	Thymus development	2	49	1.86	0.0280	9606.ENSP00000215832,9606.ENSP00000263025	MAPK1,MAPK3
GO Process	GO:0031328	Positive regulation of cellular biosynthetic process	6	2041	0.72	0.0283	9606.ENSP00000215832,9606.ENSP00000229795,9606.ENSP00000263025,9606.ENSP00000345083,9606.ENSP00000351908,9606.ENSP00000375986	MAPK1,MAPK14,MAPK3,MAP2K3,MAP3K5,MAP3K4
GO Process	GO:0043330	Response to exogenous dsRNA	2	50	1.86	0.0287	9606.ENSP00000215832,9606.ENSP00000263025	MAPK1,MAPK3
GO Process	GO:0045935	Positive regulation of nucleobase-containing compound metabolic process	6	2056	0.72	0.0292	9606.ENSP00000215832,9606.ENSP00000229795,9606.ENSP00000263025,9606.ENSP00000345083,9606.ENSP00000351908,9606.ENSP00000375986	MAPK1,MAPK14,MAPK3,MAP2K3,MAP3K5,MAP3K4
GO Process	GO:0060324	Face development	2	52	1.84	0.0307	9606.ENSP00000215832,9606.ENSP00000263025	MAPK1,MAPK3
GO Process	GO:0031281	Positive regulation of cyclase activity	2	53	1.83	0.0313	9606.ENSP00000229795,9606.ENSP00000263025	MAPK14,MAPK3
GO Process	GO:0070371	ERK1 and ERK2 cascade	2	55	1.81	0.0333	9606.ENSP00000215832,9606.ENSP00000263025	MAPK1,MAPK3
GO Process	GO:0080090	Regulation of primary metabolic process	9	5899	0.44	0.0409	9606.ENSP00000215832,9606.ENSP00000216160,9606.ENSP00000229795,9606.ENSP00000263025,9606.ENSP00000310094,9606.ENSP00000345083,9606.ENSP00000351908,9606.ENSP00000375986,9606.ENSP00000468348	MAPK1,TAB1,MAPK14,MAPK3,TAOK2,MAP2K3,MAP3K5,MAP3K4,MAP2K6
GO	GO:0009653	Anatomical	6	2229	0.68	0.0417	9606.ENSP00000215832,9606.ENSP00000216160,9606.EN	MAPK1,TAB1,MAPK14,

Process		structure morphogenesis					SP00000229795,9606.ENSP00000263025,9606.ENSP00000310094,9606.ENSP00000370503	MAPK3,TAOK2,CCM2
GO Process	GO:0051149	Positive regulation of muscle cell differentiation	2	64	1.75	0.0421	9606.ENSP00000229795,9606.ENSP00000333685	MAPK14,MAPK11
GO Function	GO:0106310	Protein serine kinase activity	9	361	1.65	6.90e-11	9606.ENSP00000215832,9606.ENSP00000229795,9606.ENSP00000263025,9606.ENSP00000310094,9606.ENSP00000333685,9606.ENSP00000345083,9606.ENSP00000351908,9606.ENSP00000375986,9606.ENSP00000468348	MAPK1,MAPK14,MAPK3,TAOK2,MAPK11,MAP2K3,MAP3K5,MAP3K4,MAP2K6
GO Function	GO:0004674	Protein serine/threonine kinase activity	9	434	1.57	1.76e-10	9606.ENSP00000215832,9606.ENSP00000229795,9606.ENSP00000263025,9606.ENSP00000310094,9606.ENSP00000333685,9606.ENSP00000345083,9606.ENSP00000351908,9606.ENSP00000375986,9606.ENSP00000468348	MAPK1,MAPK14,MAPK3,TAOK2,MAPK11,MAP2K3,MAP3K5,MAP3K4,MAP2K6
GO Function	GO:0016301	Kinase activity	10	788	1.36	1.95e-10	9606.ENSP00000215832,9606.ENSP00000216160,9606.ENSP00000229795,9606.ENSP00000263025,9606.ENSP00000310094,9606.ENSP00000333685,9606.ENSP00000345083,9606.ENSP00000351908,9606.ENSP00000375986,9606.ENSP00000468348	MAPK1,TAB1,MAPK14,MAPK3,TAOK2,MAPK11,MAP2K3,MAP3K5,MAP3K4,MAP2K6
GO Function	GO:0004708	MAP kinase kinase activity	5	17	2.72	6.03e-10	9606.ENSP00000215832,9606.ENSP00000229795,9606.ENSP00000263025,9606.ENSP00000345083,9606.ENSP00000468348	MAPK1,MAPK14,MAPK3,MAP2K3,MAP2K6
GO Function	GO:0004707	MAP kinase activity	4	15	2.68	1.11e-07	9606.ENSP00000215832,9606.ENSP00000229795,9606.ENSP00000263025,9606.ENSP00000333685	MAPK1,MAPK14,MAPK3,MAPK11
GO Function	GO:0005524	ATP binding	9	1491	1.03	1.97e-06	9606.ENSP00000215832,9606.ENSP00000229795,9606.ENSP00000263025,9606.ENSP00000310094,9606.ENSP00000333685,9606.ENSP00000345083,9606.ENSP00000351908,9606.ENSP00000375986,9606.ENSP00000468348	MAPK1,MAPK14,MAPK3,TAOK2,MAPK11,MAP2K3,MAP3K5,MAP3K4,MAP2K6
GO Function	GO:0140096	Catalytic activity, acting on a protein	10	2279	0.9	1.97e-06	9606.ENSP00000215832,9606.ENSP00000216160,9606.ENSP00000229795,9606.ENSP00000263025,9606.ENSP00000310094,9606.ENSP00000333685,9606.ENSP00000345083,9606.ENSP00000351908,9606.ENSP00000375986,9606.ENSP00000468348	MAPK1,TAB1,MAPK14,MAPK3,TAOK2,MAPK11,MAP2K3,MAP3K5,MAP3K4,MAP2K6
GO Function	GO:0004709	MAP kinase kinase kinase activity	3	27	2.3	0.00011	9606.ENSP00000310094,9606.ENSP00000351908,9606.ENSP00000375986	TAOK2,MAP3K5,MAP3K4
GO Functi	GO:0019901	Protein kinase binding	6	702	1.18	0.00016	9606.ENSP00000216160,9606.ENSP00000229795,9606.ENSP00000310094,9606.ENSP00000345083,9606.ENSP00000	TAB1,MAPK14,TAOK2,MAP2K3,MAP3K5,MAP

on							351908,9606.ENSP00000468348	2K6
GO Function	GO:0019899	Enzyme binding	8	2084	0.84	0.00036	9606.ENSP00000215832,9606.ENSP00000216160,9606.ENSP00000229795,9606.ENSP00000263025,9606.ENSP00000310094,9606.ENSP00000345083,9606.ENSP00000351908,9606.ENSP00000468348	MAPK1,TAB1,MAPK14,MAPK3,TAOK2,MAP2K3,MAP3K5,MAP2K6
GO Function	GO:0019902	Phosphatase binding	4	198	1.56	0.00059	9606.ENSP00000215832,9606.ENSP00000229795,9606.ENSP00000263025,9606.ENSP00000351908	MAPK1,MAPK14,MAPK3,MAP3K5
GO Function	GO:0048273	Mitogen-activated protein kinase p38 binding	2	6	2.78	0.0013	9606.ENSP00000216160,9606.ENSP00000229795	TAB1,MAPK14
GO Function	GO:0019209	Kinase activator activity	3	121	1.65	0.0061	9606.ENSP00000216160,9606.ENSP00000310094,9606.ENSP00000468348	TAB1,TAOK2,MAP2K6
GO Function	GO:0001784	Phosphotyrosine residue binding	2	46	1.89	0.0461	9606.ENSP00000215832,9606.ENSP00000263025	MAPK1,MAPK3
STRING clusters	CL:18406	Mixed, incl. Cellular response to sorbitol, and Activation of the AP-1 family of transcription factors	4	15	2.68	9.27e-07	9606.ENSP00000229795,9606.ENSP00000333685,9606.ENSP00000345083,9606.ENSP00000468348	MAPK14,MAPK11,MAP2K3,MAP2K6
STRING clusters	CL:18397	Mixed, incl. Basic-leucine zipper domain, and Host-pathogen interaction of human coronaviruses - MAPK signaling	5	98	1.96	2.43e-06	9606.ENSP00000229795,9606.ENSP00000333685,9606.ENSP00000345083,9606.ENSP00000375986,9606.ENSP00000468348	MAPK14,MAPK11,MAP2K3,MAP3K4,MAP2K6
STRING clusters	CL:17676	Negative feedback regulation of MAPK pathway,	2	14	2.41	0.0155	9606.ENSP00000215832,9606.ENSP00000263025	MAPK1,MAPK3

		and RAS GTPase cycle mutants						
KEGG	hsa04010	MAPK signaling pathway	10	286	1.8	1.83e-15	9606.ENSP00000215832,9606.ENSP00000216160,9606.ENSP00000229795,9606.ENSP00000263025,9606.ENSP00000310094,9606.ENSP00000333685,9606.ENSP00000345083,9606.ENSP00000351908,9606.ENSP00000375986,9606.ENSP00000468348	MAPK1,TAB1,MAPK14,MAPK3,TAOK2,MAPK11,MAP2K3,MAP3K5,MAP3K4,MAP2K6
KEGG	hsa04668	TNF signaling pathway	8	111	2.11	3.80e-14	9606.ENSP00000215832,9606.ENSP00000216160,9606.ENSP00000229795,9606.ENSP00000263025,9606.ENSP00000333685,9606.ENSP00000345083,9606.ENSP00000351908,9606.ENSP00000468348	MAPK1,TAB1,MAPK14,MAPK3,MAPK11,MAP2K3,MAP3K5,MAP2K6
KEGG	hsa04912	GnRH signaling pathway	7	87	2.16	1.63e-12	9606.ENSP00000215832,9606.ENSP00000229795,9606.ENSP00000263025,9606.ENSP00000333685,9606.ENSP00000345083,9606.ENSP00000375986,9606.ENSP00000468348	MAPK1,MAPK14,MAPK3,MAPK11,MAP2K3,MAP3K4,MAP2K6
KEGG	hsa04620	Toll-like receptor signaling pathway	7	100	2.1	3.10e-12	9606.ENSP00000215832,9606.ENSP00000216160,9606.ENSP00000229795,9606.ENSP00000263025,9606.ENSP00000333685,9606.ENSP00000345083,9606.ENSP00000468348	MAPK1,TAB1,MAPK14,MAPK3,MAPK11,MAP2K3,MAP2K6
KEGG	hsa05145	Toxoplasmosis	7	103	2.09	3.10e-12	9606.ENSP00000215832,9606.ENSP00000216160,9606.ENSP00000229795,9606.ENSP00000263025,9606.ENSP00000333685,9606.ENSP00000345083,9606.ENSP00000468348	MAPK1,TAB1,MAPK14,MAPK3,MAPK11,MAP2K3,MAP2K6
KEGG	hsa05135	Yersinia infection	7	124	2.0	8.81e-12	9606.ENSP00000215832,9606.ENSP00000216160,9606.ENSP00000229795,9606.ENSP00000263025,9606.ENSP00000333685,9606.ENSP00000345083,9606.ENSP00000468348	MAPK1,TAB1,MAPK14,MAPK3,MAPK11,MAP2K3,MAP2K6
KEGG	hsa04664	Fc epsilon RI signaling pathway	6	65	2.22	3.85e-11	9606.ENSP00000215832,9606.ENSP00000229795,9606.ENSP00000263025,9606.ENSP00000333685,9606.ENSP00000345083,9606.ENSP00000468348	MAPK1,MAPK14,MAPK3,MAPK11,MAP2K3,MAP2K6
KEGG	hsa05161	Hepatitis B	7	158	1.9	3.85e-11	9606.ENSP00000215832,9606.ENSP00000216160,9606.ENSP00000229795,9606.ENSP00000263025,9606.ENSP00000333685,9606.ENSP00000345083,9606.ENSP00000468348	MAPK1,TAB1,MAPK14,MAPK3,MAPK11,MAP2K3,MAP2K6
KEGG	hsa05170	Human immunodeficiency virus 1 infection	7	203	1.79	1.59e-10	9606.ENSP00000215832,9606.ENSP00000216160,9606.ENSP00000229795,9606.ENSP00000263025,9606.ENSP00000333685,9606.ENSP00000345083,9606.ENSP00000468348	MAPK1,TAB1,MAPK14,MAPK3,MAPK11,MAP2K3,MAP2K6
KEGG	hsa05235	PD-L1 expression and PD-1 checkpoint pathway in	6	87	2.09	1.59e-10	9606.ENSP00000215832,9606.ENSP00000229795,9606.ENSP00000263025,9606.ENSP00000333685,9606.ENSP00000345083,9606.ENSP00000468348	MAPK1,MAPK14,MAPK3,MAPK11,MAP2K3,MAP2K6

		cancer						
KEGG	hsa05132	Salmonella infection	7	209	1.78	1.67e-10	9606.ENSP00000215832,9606.ENSP00000216160,9606.ENSP00000229795,9606.ENSP00000263025,9606.ENSP00000333685,9606.ENSP00000345083,9606.ENSP00000468348	MAPK1,TAB1,MAPK14,MAPK3,MAPK11,MAP2K3,MAP2K6
KEGG	hsa04935	Growth hormone synthesis, secretion and action	6	117	1.96	6.59e-10	9606.ENSP00000215832,9606.ENSP00000229795,9606.ENSP00000263025,9606.ENSP00000333685,9606.ENSP00000345083,9606.ENSP00000468348	MAPK1,MAPK14,MAPK3,MAPK11,MAP2K3,MAP2K6
KEGG	hsa04380	Osteoclast differentiation	6	120	1.95	7.04e-10	9606.ENSP00000215832,9606.ENSP00000216160,9606.ENSP00000229795,9606.ENSP00000263025,9606.ENSP00000333685,9606.ENSP00000468348	MAPK1,TAB1,MAPK14,MAPK3,MAPK11,MAP2K6
KEGG	hsa04218	Cellular senescence	6	150	1.86	2.40e-09	9606.ENSP00000215832,9606.ENSP00000229795,9606.ENSP00000263025,9606.ENSP00000333685,9606.ENSP00000345083,9606.ENSP00000468348	MAPK1,MAPK14,MAPK3,MAPK11,MAP2K3,MAP2K6
KEGG	hsa05140	Leishmaniasis	5	69	2.11	6.61e-09	9606.ENSP00000215832,9606.ENSP00000216160,9606.ENSP00000229795,9606.ENSP00000263025,9606.ENSP00000333685	MAPK1,TAB1,MAPK14,MAPK3,MAPK11
KEGG	hsa04015	Rap1 signaling pathway	6	201	1.73	1.16e-08	9606.ENSP00000215832,9606.ENSP00000229795,9606.ENSP00000263025,9606.ENSP00000333685,9606.ENSP00000345083,9606.ENSP00000468348	MAPK1,MAPK14,MAPK3,MAPK11,MAP2K3,MAP2K6
KEGG	hsa04722	Neurotrophin signaling pathway	5	112	1.9	6.00e-08	9606.ENSP00000215832,9606.ENSP00000229795,9606.ENSP00000263025,9606.ENSP00000333685,9606.ENSP00000351908	MAPK1,MAPK14,MAPK3,MAPK11,MAP3K5
KEGG	hsa04071	Sphingolipid signaling pathway	5	116	1.89	6.72e-08	9606.ENSP00000215832,9606.ENSP00000229795,9606.ENSP00000263025,9606.ENSP00000333685,9606.ENSP00000351908	MAPK1,MAPK14,MAPK3,MAPK11,MAP3K5
KEGG	hsa04370	VEGF signaling pathway	4	56	2.11	4.44e-07	9606.ENSP00000215832,9606.ENSP00000229795,9606.ENSP00000263025,9606.ENSP00000333685	MAPK1,MAPK14,MAPK3,MAPK11
KEGG	hsa04621	NOD-like receptor signaling pathway	5	173	1.71	4.44e-07	9606.ENSP00000215832,9606.ENSP00000216160,9606.ENSP00000229795,9606.ENSP00000263025,9606.ENSP00000333685	MAPK1,TAB1,MAPK14,MAPK3,MAPK11
KEGG	hsa05130	Pathogenic Escherichia coli infection	5	187	1.68	5.87e-07	9606.ENSP00000215832,9606.ENSP00000216160,9606.ENSP00000229795,9606.ENSP00000263025,9606.ENSP00000333685	MAPK1,TAB1,MAPK14,MAPK3,MAPK11
KEGG	hsa05167	Kaposi sarcoma-associated herpesvirus infection	5	187	1.68	5.87e-07	9606.ENSP00000215832,9606.ENSP00000229795,9606.ENSP00000263025,9606.ENSP00000333685,9606.ENSP00000468348	MAPK1,MAPK14,MAPK3,MAPK11,MAP2K6

KEGG	hsa05169	Epstein-Barr virus infection	5	192	1.67	6.09e-07	9606.ENSP00000216160,9606.ENSP00000229795,9606.ENSP00000333685,9606.ENSP00000345083,9606.ENSP00000468348	TAB1,MAPK14,MAPK11,MAP2K3,MAP2K6
KEGG	hsa04917	Prolactin signaling pathway	4	68	2.02	7.42e-07	9606.ENSP00000215832,9606.ENSP00000229795,9606.ENSP00000263025,9606.ENSP00000333685	MAPK1,MAPK14,MAPK3,MAPK11
KEGG	hsa05133	Pertussis	4	73	1.99	9.35e-07	9606.ENSP00000215832,9606.ENSP00000229795,9606.ENSP00000263025,9606.ENSP00000333685	MAPK1,MAPK14,MAPK3,MAPK11
KEGG	hsa05131	Shigellosis	5	218	1.61	9.79e-07	9606.ENSP00000215832,9606.ENSP00000216160,9606.ENSP00000229795,9606.ENSP00000263025,9606.ENSP00000333685	MAPK1,TAB1,MAPK14,MAPK3,MAPK11
KEGG	hsa05163	Human cytomegalovirus infection	5	217	1.62	9.79e-07	9606.ENSP00000215832,9606.ENSP00000229795,9606.ENSP00000263025,9606.ENSP00000333685,9606.ENSP00000468348	MAPK1,MAPK14,MAPK3,MAPK11,MAP2K6
KEGG	hsa04658	Th1 and Th2 cell differentiation	4	85	1.93	1.50e-06	9606.ENSP00000215832,9606.ENSP00000229795,9606.ENSP00000263025,9606.ENSP00000333685	MAPK1,MAPK14,MAPK3,MAPK11
KEGG	hsa04657	IL-17 signaling pathway	4	91	1.9	1.89e-06	9606.ENSP00000215832,9606.ENSP00000229795,9606.ENSP00000263025,9606.ENSP00000333685	MAPK1,MAPK14,MAPK3,MAPK11
KEGG	hsa04750	Inflammatory mediator regulation of TRP channels	4	92	1.89	1.90e-06	9606.ENSP00000229795,9606.ENSP00000333685,9606.ENSP00000345083,9606.ENSP00000468348	MAPK14,MAPK11,MAP2K3,MAP2K6
KEGG	hsa01522	Endocrine resistance	4	94	1.88	2.00e-06	9606.ENSP00000215832,9606.ENSP00000229795,9606.ENSP00000263025,9606.ENSP00000333685	MAPK1,MAPK14,MAPK3,MAPK11
KEGG	hsa04914	Progesterone-mediated oocyte maturation	4	95	1.88	2.02e-06	9606.ENSP00000215832,9606.ENSP00000229795,9606.ENSP00000263025,9606.ENSP00000333685	MAPK1,MAPK14,MAPK3,MAPK11
KEGG	hsa04933	AGE-RAGE signaling pathway in diabetic complications	4	96	1.87	2.04e-06	9606.ENSP00000215832,9606.ENSP00000229795,9606.ENSP00000263025,9606.ENSP00000333685	MAPK1,MAPK14,MAPK3,MAPK11
KEGG	hsa05142	Chagas disease	4	97	1.87	2.06e-06	9606.ENSP00000215832,9606.ENSP00000229795,9606.ENSP00000263025,9606.ENSP00000333685	MAPK1,MAPK14,MAPK3,MAPK11
KEGG	hsa04659	Th17 cell differentiation	4	99	1.86	2.17e-06	9606.ENSP00000215832,9606.ENSP00000229795,9606.ENSP00000263025,9606.ENSP00000333685	MAPK1,MAPK14,MAPK3,MAPK11
KEGG	hsa04660	T cell receptor signaling pathway	4	100	1.86	2.19e-06	9606.ENSP00000215832,9606.ENSP00000229795,9606.ENSP00000263025,9606.ENSP00000333685	MAPK1,MAPK14,MAPK3,MAPK11

KEGG	hsa04625	C-type lectin receptor signaling pathway	4	101	1.85	2.21e-06	9606.ENSP00000215832,9606.ENSP00000229795,9606.ENSP00000263025,9606.ENSP00000333685	MAPK1,MAPK14,MAPK3,MAPK11
KEGG	hsa04114	Oocyte meiosis	4	121	1.77	4.35e-06	9606.ENSP00000215832,9606.ENSP00000229795,9606.ENSP00000263025,9606.ENSP00000333685	MAPK1,MAPK14,MAPK3,MAPK11
KEGG	hsa04611	Platelet activation	4	122	1.77	4.37e-06	9606.ENSP00000215832,9606.ENSP00000229795,9606.ENSP00000263025,9606.ENSP00000333685	MAPK1,MAPK14,MAPK3,MAPK11
KEGG	hsa04068	FoxO signaling pathway	4	126	1.75	4.83e-06	9606.ENSP00000215832,9606.ENSP00000229795,9606.ENSP00000263025,9606.ENSP00000333685	MAPK1,MAPK14,MAPK3,MAPK11
KEGG	hsa04926	Relaxin signaling pathway	4	126	1.75	4.83e-06	9606.ENSP00000215832,9606.ENSP00000229795,9606.ENSP00000263025,9606.ENSP00000333685	MAPK1,MAPK14,MAPK3,MAPK11
KEGG	hsa05418	Fluid shear stress and atherosclerosis	4	129	1.74	5.04e-06	9606.ENSP00000229795,9606.ENSP00000333685,9606.ENSP00000351908,9606.ENSP00000468348	MAPK14,MAPK11,MAP3K5,MAP2K6
KEGG	hsa05014	Amyotrophic lateral sclerosis	5	350	1.41	6.09e-06	9606.ENSP00000229795,9606.ENSP00000333685,9606.ENSP00000345083,9606.ENSP00000351908,9606.ENSP00000468348	MAPK14,MAPK11,MAP2K3,MAP3K5,MAP2K6
KEGG	hsa04550	Signaling pathways regulating pluripotency of stem cells	4	141	1.71	6.80e-06	9606.ENSP00000215832,9606.ENSP00000229795,9606.ENSP00000263025,9606.ENSP00000333685	MAPK1,MAPK14,MAPK3,MAPK11
KEGG	hsa04723	Retrograde endocannabinoid signaling	4	142	1.7	6.84e-06	9606.ENSP00000215832,9606.ENSP00000229795,9606.ENSP00000263025,9606.ENSP00000333685	MAPK1,MAPK14,MAPK3,MAPK11
KEGG	hsa04261	Adrenergic signaling in cardiomyocytes	4	146	1.69	7.45e-06	9606.ENSP00000215832,9606.ENSP00000229795,9606.ENSP00000263025,9606.ENSP00000333685	MAPK1,MAPK14,MAPK3,MAPK11
KEGG	hsa05152	Tuberculosis	4	165	1.64	1.17e-05	9606.ENSP00000215832,9606.ENSP00000229795,9606.ENSP00000263025,9606.ENSP00000333685	MAPK1,MAPK14,MAPK3,MAPK11
KEGG	hsa05205	Proteoglycans in cancer	4	194	1.57	2.16e-05	9606.ENSP00000215832,9606.ENSP00000229795,9606.ENSP00000263025,9606.ENSP00000333685	MAPK1,MAPK14,MAPK3,MAPK11
KEGG	hsa04714	Thermogenesis	4	226	1.5	3.83e-05	9606.ENSP00000229795,9606.ENSP00000333685,9606.ENSP00000345083,9606.ENSP00000351908	MAPK14,MAPK11,MAP2K3,MAP3K5
KEGG	hsa01524	Platinum drug resistance	3	70	1.89	5.29e-05	9606.ENSP00000215832,9606.ENSP00000263025,9606.ENSP00000351908	MAPK1,MAPK3,MAP3K5
KEGG	hsa05020	Prion disease	4	263	1.44	6.64e-	9606.ENSP00000215832,9606.ENSP00000229795,9606.EN	MAPK1,MAPK14,MAP

						05	SP00000263025,9606.ENSP00000333685	K3,MAPK11
KEGG	hsa04210	Apoptosis	3	131	1.61	0.0003 1	9606.ENSP00000215832,9606.ENSP00000263025,9606.EN SP00000351908	MAPK1,MAPK3,MAP3 K5
KEGG	hsa04960	Aldosterone-regulated sodium reabsorption	2	37	1.99	0.0013	9606.ENSP00000215832,9606.ENSP00000263025	MAPK1,MAPK3
KEGG	hsa05216	Thyroid cancer	2	37	1.99	0.0013	9606.ENSP00000215832,9606.ENSP00000263025	MAPK1,MAPK3
KEGG	hsa05219	Bladder cancer	2	40	1.95	0.0015	9606.ENSP00000215832,9606.ENSP00000263025	MAPK1,MAPK3
KEGG	hsa04930	Type II diabetes mellitus	2	45	1.9	0.0018	9606.ENSP00000215832,9606.ENSP00000263025	MAPK1,MAPK3
KEGG	hsa04730	Long-term depression	2	59	1.78	0.0029	9606.ENSP00000215832,9606.ENSP00000263025	MAPK1,MAPK3
KEGG	hsa05213	Endometrial cancer	2	58	1.79	0.0029	9606.ENSP00000215832,9606.ENSP00000263025	MAPK1,MAPK3
KEGG	hsa04720	Long-term potentiation	2	63	1.75	0.0033	9606.ENSP00000215832,9606.ENSP00000263025	MAPK1,MAPK3
KEGG	hsa04929	GnRH secretion	2	63	1.75	0.0033	9606.ENSP00000215832,9606.ENSP00000263025	MAPK1,MAPK3
KEGG	hsa05120	Epithelial cell signaling in Helicobacter pylori infection	2	65	1.74	0.0034	9606.ENSP00000229795,9606.ENSP00000333685	MAPK14,MAPK11
KEGG	hsa05211	Renal cell carcinoma	2	65	1.74	0.0034	9606.ENSP00000215832,9606.ENSP00000263025	MAPK1,MAPK3
KEGG	hsa04520	Adherens junction	2	69	1.72	0.0035	9606.ENSP00000215832,9606.ENSP00000263025	MAPK1,MAPK3
KEGG	hsa04622	RIG-I-like receptor signaling pathway	2	69	1.72	0.0035	9606.ENSP00000229795,9606.ENSP00000333685	MAPK14,MAPK11
KEGG	hsa05221	Acute myeloid leukemia	2	67	1.73	0.0035	9606.ENSP00000215832,9606.ENSP00000263025	MAPK1,MAPK3
KEGG	hsa05223	Non-small cell lung cancer	2	68	1.72	0.0035	9606.ENSP00000215832,9606.ENSP00000263025	MAPK1,MAPK3
KEGG	hsa05230	Central carbon metabolism in cancer	2	68	1.72	0.0035	9606.ENSP00000215832,9606.ENSP00000263025	MAPK1,MAPK3

KEGG	hsa05212	Pancreatic cancer	2	71	1.7	0.0036	9606.ENSP00000215832,9606.ENSP00000263025	MAPK1,MAPK3
KEGG	hsa05214	Glioma	2	71	1.7	0.0036	9606.ENSP00000215832,9606.ENSP00000263025	MAPK1,MAPK3
KEGG	hsa05218	Melanoma	2	72	1.7	0.0036	9606.ENSP00000215832,9606.ENSP00000263025	MAPK1,MAPK3
KEGG	hsa05220	Chronic myeloid leukemia	2	75	1.68	0.0038	9606.ENSP00000215832,9606.ENSP00000263025	MAPK1,MAPK3
KEGG	hsa01521	EGFR tyrosine kinase inhibitor resistance	2	77	1.67	0.0040	9606.ENSP00000215832,9606.ENSP00000263025	MAPK1,MAPK3
KEGG	hsa04662	B cell receptor signaling pathway	2	78	1.66	0.0040	9606.ENSP00000215832,9606.ENSP00000263025	MAPK1,MAPK3
KEGG	hsa05010	Alzheimer disease	3	354	1.18	0.0040	9606.ENSP00000215832,9606.ENSP00000263025,9606.ENSP00000351908	MAPK1,MAPK3,MAP3K5
KEGG	hsa04012	ErbB signaling pathway	2	81	1.65	0.0042	9606.ENSP00000215832,9606.ENSP00000263025	MAPK1,MAPK3
KEGG	hsa05210	Colorectal cancer	2	82	1.64	0.0043	9606.ENSP00000215832,9606.ENSP00000263025	MAPK1,MAPK3
KEGG	hsa04540	Gap junction	2	87	1.61	0.0047	9606.ENSP00000215832,9606.ENSP00000263025	MAPK1,MAPK3
KEGG	hsa04350	TGF-beta signaling pathway	2	91	1.6	0.0050	9606.ENSP00000215832,9606.ENSP00000263025	MAPK1,MAPK3
KEGG	hsa04666	Fc gamma R-mediated phagocytosis	2	90	1.6	0.0050	9606.ENSP00000215832,9606.ENSP00000263025	MAPK1,MAPK3
KEGG	hsa04713	Circadian entrainment	2	91	1.6	0.0050	9606.ENSP00000215832,9606.ENSP00000263025	MAPK1,MAPK3
KEGG	hsa04916	Melanogenesis	2	95	1.58	0.0053	9606.ENSP00000215832,9606.ENSP00000263025	MAPK1,MAPK3
KEGG	hsa05231	Choline metabolism in cancer	2	95	1.58	0.0053	9606.ENSP00000215832,9606.ENSP00000263025	MAPK1,MAPK3
KEGG	hsa05215	Prostate cancer	2	97	1.57	0.0054	9606.ENSP00000215832,9606.ENSP00000263025	MAPK1,MAPK3
KEGG	hsa04066	HIF-1 signaling pathway	2	102	1.55	0.0059	9606.ENSP00000215832,9606.ENSP00000263025	MAPK1,MAPK3
KEGG	hsa04928	Parathyroid hormone synthesis,	2	104	1.54	0.0060	9606.ENSP00000215832,9606.ENSP00000263025	MAPK1,MAPK3

		secretion and action						
KEGG	hsa04726	Serotonergic synapse	2	108	1.52	0.0064	9606.ENSP00000215832,9606.ENSP00000263025	MAPK1,MAPK3
KEGG	hsa04725	Cholinergic synapse	2	109	1.52	0.0065	9606.ENSP00000215832,9606.ENSP00000263025	MAPK1,MAPK3
KEGG	hsa04670	Leukocyte transendothelial migration	2	111	1.51	0.0066	9606.ENSP00000229795,9606.ENSP00000333685	MAPK14,MAPK11
KEGG	hsa04724	Glutamatergic synapse	2	112	1.5	0.0067	9606.ENSP00000215832,9606.ENSP00000263025	MAPK1,MAPK3
KEGG	hsa04650	Natural killer cell mediated cytotoxicity	2	120	1.47	0.0075	9606.ENSP00000215832,9606.ENSP00000263025	MAPK1,MAPK3
KEGG	hsa04919	Thyroid hormone signaling pathway	2	120	1.47	0.0075	9606.ENSP00000215832,9606.ENSP00000263025	MAPK1,MAPK3
KEGG	hsa04728	Dopaminergic synapse	2	126	1.45	0.0081	9606.ENSP00000229795,9606.ENSP00000333685	MAPK14,MAPK11
KEGG	hsa04140	Autophagy - animal	2	131	1.44	0.0086	9606.ENSP00000215832,9606.ENSP00000263025	MAPK1,MAPK3
KEGG	hsa04270	Vascular smooth muscle contraction	2	132	1.43	0.0087	9606.ENSP00000215832,9606.ENSP00000263025	MAPK1,MAPK3
KEGG	hsa04371	Apelin signaling pathway	2	133	1.43	0.0087	9606.ENSP00000215832,9606.ENSP00000263025	MAPK1,MAPK3
KEGG	hsa04910	Insulin signaling pathway	2	132	1.43	0.0087	9606.ENSP00000215832,9606.ENSP00000263025	MAPK1,MAPK3
KEGG	hsa04915	Estrogen signaling pathway	2	133	1.43	0.0087	9606.ENSP00000215832,9606.ENSP00000263025	MAPK1,MAPK3
KEGG	hsa04072	Phospholipase D signaling pathway	2	147	1.39	0.0101	9606.ENSP00000215832,9606.ENSP00000263025	MAPK1,MAPK3
KEGG	hsa04150	mTOR signaling pathway	2	150	1.38	0.0101	9606.ENSP00000215832,9606.ENSP00000263025	MAPK1,MAPK3
KEGG	hsa04921	Oxytocin signaling	2	147	1.39	0.0101	9606.ENSP00000215832,9606.ENSP00000263025	MAPK1,MAPK3

		pathway						
KEGG	hsa05034	Alcoholism	2	146	1.39	0.0101	9606.ENSP00000215832,9606.ENSP00000263025	MAPK1,MAPK3
KEGG	hsa05224	Breast cancer	2	146	1.39	0.0101	9606.ENSP00000215832,9606.ENSP00000263025	MAPK1,MAPK3
KEGG	hsa05226	Gastric cancer	2	146	1.39	0.0101	9606.ENSP00000215832,9606.ENSP00000263025	MAPK1,MAPK3
KEGG	hsa04934	Cushing syndrome	2	153	1.37	0.0104	9606.ENSP00000215832,9606.ENSP00000263025	MAPK1,MAPK3
KEGG	hsa05160	Hepatitis C	2	157	1.36	0.0109	9606.ENSP00000215832,9606.ENSP00000263025	MAPK1,MAPK3
KEGG	hsa05206	MicroRNAs in cancer	2	159	1.35	0.0110	9606.ENSP00000215832,9606.ENSP00000263025	MAPK1,MAPK3
KEGG	hsa05225	Hepatocellular carcinoma	2	161	1.35	0.0112	9606.ENSP00000215832,9606.ENSP00000263025	MAPK1,MAPK3
KEGG	hsa04022	cGMP-PKG signaling pathway	2	163	1.34	0.0113	9606.ENSP00000215832,9606.ENSP00000263025	MAPK1,MAPK3
KEGG	hsa05164	Influenza A	2	163	1.34	0.0113	9606.ENSP00000215832,9606.ENSP00000263025	MAPK1,MAPK3
KEGG	hsa04360	Axon guidance	2	176	1.31	0.0129	9606.ENSP00000215832,9606.ENSP00000263025	MAPK1,MAPK3
KEGG	hsa05203	Viral carcinogenesis	2	183	1.29	0.0138	9606.ENSP00000215832,9606.ENSP00000263025	MAPK1,MAPK3
KEGG	hsa04062	Chemokine signaling pathway	2	186	1.28	0.0141	9606.ENSP00000215832,9606.ENSP00000263025	MAPK1,MAPK3
KEGG	hsa04510	Focal adhesion	2	195	1.26	0.0153	9606.ENSP00000215832,9606.ENSP00000263025	MAPK1,MAPK3
KEGG	hsa04024	cAMP signaling pathway	2	207	1.24	0.0170	9606.ENSP00000215832,9606.ENSP00000263025	MAPK1,MAPK3
KEGG	hsa04810	Regulation of actin cytoskeleton	2	209	1.23	0.0172	9606.ENSP00000215832,9606.ENSP00000263025	MAPK1,MAPK3
KEGG	hsa05166	Human T-cell leukemia virus 1 infection	2	210	1.23	0.0172	9606.ENSP00000215832,9606.ENSP00000263025	MAPK1,MAPK3
KEGG	hsa04014	Ras signaling pathway	2	225	1.2	0.0195	9606.ENSP00000215832,9606.ENSP00000263025	MAPK1,MAPK3
KEGG	hsa05165	Human papillomavirus infection	2	324	1.04	0.0387	9606.ENSP00000215832,9606.ENSP00000263025	MAPK1,MAPK3
KEGG	hsa04151	PI3K-Akt	2	349	1.01	0.0442	9606.ENSP00000215832,9606.ENSP00000263025	MAPK1,MAPK3

		signaling pathway						
Reactome	HSA-450294	MAP kinase activation	7	61	2.31	3.15e-12	9606.ENSP00000215832,9606.ENSP00000216160,9606.ENSP00000229795,9606.ENSP00000263025,9606.ENSP00000333685,9606.ENSP00000345083,9606.ENSP00000468348	MAPK1,TAB1,MAPK14,MAPK3,MAPK11,MAP2K3,MAP2K6
Reactome	HSA-2559580	Oxidative Stress Induced Senescence	7	92	2.13	1.61e-11	9606.ENSP00000215832,9606.ENSP00000229795,9606.ENSP00000263025,9606.ENSP00000333685,9606.ENSP00000345083,9606.ENSP00000351908,9606.ENSP00000468348	MAPK1,MAPK14,MAPK3,MAPK11,MAP2K3,MAP3K5,MAP2K6
Reactome	HSA-450302	Activated TAK1 mediates p38 MAPK activation	5	22	2.61	1.63e-10	9606.ENSP00000216160,9606.ENSP00000229795,9606.ENSP00000333685,9606.ENSP00000345083,9606.ENSP00000468348	TAB1,MAPK14,MAPK11,MAP2K3,MAP2K6
Reactome	HSA-450341	Activation of the AP-1 family of transcription factors	4	9	2.9	3.88e-09	9606.ENSP00000215832,9606.ENSP00000229795,9606.ENSP00000263025,9606.ENSP00000333685	MAPK1,MAPK14,MAPK3,MAPK11
Reactome	HSA-198753	ERK/MAPK targets	4	22	2.51	7.74e-08	9606.ENSP00000215832,9606.ENSP00000229795,9606.ENSP00000263025,9606.ENSP00000333685	MAPK1,MAPK14,MAPK3,MAPK11
Reactome	HSA-5668599	RHO GTPases Activate NADPH Oxidases	4	24	2.47	1.02e-07	9606.ENSP00000215832,9606.ENSP00000229795,9606.ENSP00000263025,9606.ENSP00000333685	MAPK1,MAPK14,MAPK3,MAPK11
Reactome	HSA-187687	Signalling to ERKs	4	34	2.32	3.24e-07	9606.ENSP00000215832,9606.ENSP00000229795,9606.ENSP00000263025,9606.ENSP00000333685	MAPK1,MAPK14,MAPK3,MAPK11
Reactome	HSA-168638	NOD1/2 Signaling Pathway	4	35	2.31	3.49e-07	9606.ENSP00000216160,9606.ENSP00000229795,9606.ENSP00000333685,9606.ENSP00000468348	TAB1,MAPK14,MAPK11,MAP2K6
Reactome	HSA-525793	Myogenesis	3	29	2.27	4.03e-05	9606.ENSP00000229795,9606.ENSP00000333685,9606.ENSP00000468348	MAPK14,MAPK11,MAP2K6
Reactome	HSA-5663205	Infectious disease	6	917	1.07	0.00024	9606.ENSP00000215832,9606.ENSP00000216160,9606.ENSP00000229795,9606.ENSP00000263025,9606.ENSP00000345083,9606.ENSP00000468348	MAPK1,TAB1,MAPK14,MAPK3,MAP2K3,MAP2K6
Reactome	HSA-9652169	Signaling by MAP2K mutants	2	4	2.95	0.00025	9606.ENSP00000215832,9606.ENSP00000263025	MAPK1,MAPK3
Reactome	HSA-5674499	Negative feedback regulation of MAPK pathway	2	6	2.78	0.00045	9606.ENSP00000215832,9606.ENSP00000263025	MAPK1,MAPK3
Reacto	HSA-444257	RSK activation	2	7	2.71	0.0005	9606.ENSP00000215832,9606.ENSP00000263025	MAPK1,MAPK3

me						7		
Reactome	HSA-9635465	Suppression of apoptosis	2	7	2.71	0.00057	9606.ENSP00000215832,9606.ENSP00000263025	MAPK1,MAPK3
Reactome	HSA-2151209	Activation of PPARGC1A (PGC-1alpha) by phosphorylation	2	10	2.55	0.00097	9606.ENSP00000229795,9606.ENSP00000333685	MAPK14,MAPK11
Reactome	HSA-74749	Signal attenuation	2	10	2.55	0.00097	9606.ENSP00000215832,9606.ENSP00000263025	MAPK1,MAPK3
Reactome	HSA-170968	Frs2-mediated activation	2	12	2.47	0.0011	9606.ENSP00000215832,9606.ENSP00000263025	MAPK1,MAPK3
Reactome	HSA-376172	DSCAM interactions	2	11	2.51	0.0011	9606.ENSP00000229795,9606.ENSP00000333685	MAPK14,MAPK11
Reactome	HSA-5210891	Uptake and function of anthrax toxins	2	11	2.51	0.0011	9606.ENSP00000345083,9606.ENSP00000468348	MAP2K3,MAP2K6
Reactome	HSA-9627069	Regulation of the apoptosome activity	2	11	2.51	0.0011	9606.ENSP00000215832,9606.ENSP00000263025	MAPK1,MAPK3
Reactome	HSA-171007	p38MAPK events	2	13	2.44	0.0013	9606.ENSP00000229795,9606.ENSP00000333685	MAPK14,MAPK11
Reactome	HSA-202670	ERKs are inactivated	2	13	2.44	0.0013	9606.ENSP00000215832,9606.ENSP00000263025	MAPK1,MAPK3
Reactome	HSA-879415	Advanced glycosylation endproduct receptor signaling	2	13	2.44	0.0013	9606.ENSP00000215832,9606.ENSP00000263025	MAPK1,MAPK3
Reactome	HSA-162658	Golgi Cisternae Pericentriolar Stack Reorganization	2	14	2.41	0.0014	9606.ENSP00000215832,9606.ENSP00000263025	MAPK1,MAPK3
Reactome	HSA-1502540	Signaling by Activin	2	15	2.38	0.0015	9606.ENSP00000215832,9606.ENSP00000263025	MAPK1,MAPK3
Reactome	HSA-1295596	Spry regulation of FGF signaling	2	16	2.35	0.0017	9606.ENSP00000215832,9606.ENSP00000263025	MAPK1,MAPK3
Reactome	HSA-2454202	Fc epsilon receptor (FCERI)	3	131	1.61	0.0018	9606.ENSP00000215832,9606.ENSP00000216160,9606.ENSP00000263025	MAPK1,TAB1,MAPK3

		signaling						
Reactome	HSA-450604	KSRP (KHSRP) binds and destabilizes mRNA	2	17	2.32	0.0018	9606.ENSP00000229795,9606.ENSP00000333685	MAPK14,MAPK11
Reactome	HSA-881907	Gastrin-CREB signalling pathway via PKC and MAPK	2	18	2.3	0.0020	9606.ENSP00000215832,9606.ENSP00000263025	MAPK1,MAPK3
Reactome	HSA-9725371	Nuclear events stimulated by ALK signaling in cancer	2	19	2.28	0.0021	9606.ENSP00000215832,9606.ENSP00000263025	MAPK1,MAPK3
Reactome	HSA-445144	Signal transduction by L1	2	21	2.23	0.0025	9606.ENSP00000215832,9606.ENSP00000263025	MAPK1,MAPK3
Reactome	HSA-1181150	Signaling by NODAL	2	22	2.21	0.0026	9606.ENSP00000215832,9606.ENSP00000263025	MAPK1,MAPK3
Reactome	HSA-9768919	NPAS4 regulates expression of target genes	2	22	2.21	0.0026	9606.ENSP00000215832,9606.ENSP00000263025	MAPK1,MAPK3
Reactome	HSA-112409	RAF-independent MAPK1/3 activation	2	23	2.19	0.0028	9606.ENSP00000215832,9606.ENSP00000263025	MAPK1,MAPK3
Reactome	HSA-5633007	Regulation of TP53 Activity	3	159	1.53	0.0028	9606.ENSP00000229795,9606.ENSP00000333685,9606.ENSP00000468348	MAPK14,MAPK11,MAP2K6
Reactome	HSA-982772	Growth hormone receptor signaling	2	23	2.19	0.0028	9606.ENSP00000215832,9606.ENSP00000263025	MAPK1,MAPK3
Reactome	HSA-8940973	RUNX2 regulates osteoblast differentiation	2	24	2.17	0.0029	9606.ENSP00000215832,9606.ENSP00000263025	MAPK1,MAPK3
Reactome	HSA-9634638	Estrogen-dependent nuclear events downstream of	2	24	2.17	0.0029	9606.ENSP00000215832,9606.ENSP00000263025	MAPK1,MAPK3

		ESR-membrane signaling						
Reactome	HSA-9658195	Leishmania infection	3	165	1.51	0.0029	9606.ENSP00000215832,9606.ENSP00000229795,9606.ENSP00000263025	MAPK1,MAPK14,MAPK3
Reactome	HSA-2173795	Downregulation of SMAD2/3:SMAD4 transcriptional activity	2	29	2.09	0.0038	9606.ENSP00000215832,9606.ENSP00000263025	MAPK1,MAPK3
Reactome	HSA-456926	Thrombin signalling through proteinase activated receptors (PARs)	2	32	2.05	0.0042	9606.ENSP00000215832,9606.ENSP00000263025	MAPK1,MAPK3
Reactome	HSA-2559585	Oncogene Induced Senescence	2	33	2.04	0.0044	9606.ENSP00000215832,9606.ENSP00000263025	MAPK1,MAPK3
Reactome	HSA-2871796	FCERI mediated MAPK activation	2	34	2.02	0.0045	9606.ENSP00000215832,9606.ENSP00000263025	MAPK1,MAPK3
Reactome	HSA-422475	Axon guidance	4	551	1.11	0.0045	9606.ENSP00000215832,9606.ENSP00000229795,9606.ENSP00000263025,9606.ENSP00000333685	MAPK1,MAPK14,MAPK3,MAPK11
Reactome	HSA-1266738	Developmental Biology	5	1108	0.91	0.0049	9606.ENSP00000215832,9606.ENSP00000229795,9606.ENSP00000263025,9606.ENSP00000333685,9606.ENSP00000468348	MAPK1,MAPK14,MAPK3,MAPK11,MAP2K6
Reactome	HSA-2173796	SMAD2/SMAD3:SMAD4 heterotrimer regulates transcription	2	36	2.0	0.0049	9606.ENSP00000215832,9606.ENSP00000263025	MAPK1,MAPK3
Reactome	HSA-5663213	RHO GTPases Activate WASPs and WAVEs	2	36	2.0	0.0049	9606.ENSP00000215832,9606.ENSP00000263025	MAPK1,MAPK3
Reactome	HSA-6802948	Signaling by high-kinase activity BRAF	2	36	2.0	0.0049	9606.ENSP00000215832,9606.ENSP00000263025	MAPK1,MAPK3

		mutants						
Reactome	HSA-5674135	MAP2K and MAPK activation	2	40	1.95	0.0057	9606.ENSP00000215832,9606.ENSP00000263025	MAPK1,MAPK3
Reactome	HSA-9656223	Signaling by RAF1 mutants	2	41	1.94	0.0058	9606.ENSP00000215832,9606.ENSP00000263025	MAPK1,MAPK3
Reactome	HSA-212436	Generic Transcription Pathway	5	1215	0.87	0.0068	9606.ENSP00000215832,9606.ENSP00000229795,9606.ENSP00000263025,9606.ENSP00000333685,9606.ENSP00000468348	MAPK1,MAPK14,MAPK3,MAPK11,MAP2K6
Reactome	HSA-6802946	Signaling by moderate kinase activity BRAF mutants	2	45	1.9	0.0068	9606.ENSP00000215832,9606.ENSP00000263025	MAPK1,MAPK3
Reactome	HSA-6802955	Paradoxical activation of RAF signaling by kinase inactive BRAF	2	45	1.9	0.0068	9606.ENSP00000215832,9606.ENSP00000263025	MAPK1,MAPK3
Reactome	HSA-9649948	Signaling downstream of RAS mutants	2	45	1.9	0.0068	9606.ENSP00000215832,9606.ENSP00000263025	MAPK1,MAPK3
Reactome	HSA-76002	Platelet activation, signaling and aggregation	3	260	1.32	0.0076	9606.ENSP00000215832,9606.ENSP00000229795,9606.ENSP00000263025	MAPK1,MAPK14,MAPK3
Reactome	HSA-8943724	Regulation of PTEN gene transcription	2	60	1.78	0.0102	9606.ENSP00000215832,9606.ENSP00000263025	MAPK1,MAPK3
Reactome	HSA-9664422	FCGR3A-mediated phagocytosis	2	62	1.76	0.0107	9606.ENSP00000215832,9606.ENSP00000263025	MAPK1,MAPK3
Reactome	HSA-375165	NCAM signaling for neurite out-growth	2	63	1.75	0.0108	9606.ENSP00000215832,9606.ENSP00000263025	MAPK1,MAPK3
Reactome	HSA-2029482	Regulation of actin dynamics for phagocytic cup formation	2	64	1.75	0.0111	9606.ENSP00000215832,9606.ENSP00000263025	MAPK1,MAPK3

Reactome	HSA-6802952	Signaling by BRAF and RAF1 fusions	2	65	1.74	0.0113	9606.ENSP00000215832,9606.ENSP00000263025	MAPK1,MAPK3
Reactome	HSA-3371453	Regulation of HSF1-mediated heat shock response	2	69	1.72	0.0126	9606.ENSP00000215832,9606.ENSP00000263025	MAPK1,MAPK3
Reactome	HSA-2559582	Senescence-Associated Secretory Phenotype (SASP)	2	81	1.65	0.0164	9606.ENSP00000215832,9606.ENSP00000263025	MAPK1,MAPK3
Reactome	HSA-6804756	Regulation of TP53 Activity through Phosphorylation	2	92	1.59	0.0199	9606.ENSP00000229795,9606.ENSP00000333685	MAPK14,MAPK11
Reactome	HSA-4420097	VEGFA-VEGFR2 Pathway	2	96	1.57	0.0210	9606.ENSP00000229795,9606.ENSP00000333685	MAPK14,MAPK11
Reactome	HSA-6811558	PI5P, PP2A and IER3 Regulate PI3K/AKT Signaling	2	106	1.53	0.0251	9606.ENSP00000215832,9606.ENSP00000263025	MAPK1,MAPK3
Reactome	HSA-9020702	Interleukin-1 signaling	2	111	1.51	0.0273	9606.ENSP00000216160,9606.ENSP00000468348	TAB1,MAP2K6

Table 6. Pathway Enrichment Analysis for GO, KEGG, STRING clusters and Reactome Terms within MAP2K4 gene

#category	term ID	term description	observed gene count	background gene count	Strength	false discovery rate	matching proteins in your network (IDs)	matching proteins in your network (labels)
GO Process	GO:0038095	Fc-epsilon receptor signaling pathway	5	23	2.59	2.84e-08	9606.ENSP00000358335,9606.ENSP00000378974,9606.ENSP00000382423,9606.ENSP00000394560,9606.ENSP00000410402	MAP3K7,MAPK8,MAP3K1,MAPK9,MAP2K4
GO Process	GO:0051403	Stress-activated MAPK cascade	6	75	2.16	2.84e-08	9606.ENSP00000229795,9606.ENSP00000351908,9606.ENSP00000358335,9606.ENSP00000378974,9606.ENSP00000394560,9606.ENSP00000410402	MAPK14,MAP3K5,MAP3K7,MAPK8,MAPK9,MAP2K4
GO Process	GO:0000165	MAPK cascade	7	219	1.76	2.96e-08	9606.ENSP00000229795,9606.ENSP00000351908,9606.ENSP00000358335,9606.ENSP00000378974,9606.ENSP00000382423,9606.ENSP00000394560,9606.ENSP00000410402	MAPK14,MAP3K5,MAP3K7,MAPK8,MAP3K1,MAPK9,MAP2K4
GO Process	GO:0007254	JNK cascade	5	57	2.2	3.11e-07	9606.ENSP00000351908,9606.ENSP00000358335,9606.ENSP00000378974,9606.ENSP00000394560,9606.ENSP00000410402	MAP3K5,MAP3K7,MAPK8,MAPK9,MAP2K4
GO Process	GO:0090398	Cellular senescence	5	60	2.17	3.40e-07	9606.ENSP00000229795,9606.ENSP00000351908,9606.ENSP00000378974,9606.ENSP00000394560,9606.ENSP00000410402	MAPK14,MAP3K5,MAPK8,MAPK9,MAP2K4
GO Process	GO:0006468	Protein phosphorylation	7	736	1.23	5.96e-05	9606.ENSP00000229795,9606.ENSP00000351908,9606.ENSP00000358335,9606.ENSP00000378974,9606.ENSP00000382423,9606.ENSP00000394560,9606.ENSP00000410402	MAPK14,MAP3K5,MAP3K7,MAPK8,MAP3K1,MAPK9,MAP2K4
GO Process	GO:0038066	p38MAPK cascade	3	13	2.62	0.00013	9606.ENSP00000229795,9606.ENSP00000351908,9606.ENSP00000358335	MAPK14,MAP3K5,MAP3K7
GO Process	GO:0050776	Regulation of immune response	7	844	1.17	0.00013	9606.ENSP00000229795,9606.ENSP00000358335,9606.ENSP00000378974,9606.ENSP00000382423,9606.ENSP00000394560,9606.ENSP00000403701,9606.ENSP00000410402	MAPK14,MAP3K7,MAPK8,MAP3K1,MAPK9,ARRB2,MAP2K4
GO Process	GO:0043410	Positive regulation of MAPK cascade	5	481	1.27	0.0038	9606.ENSP00000351908,9606.ENSP00000358335,9606.ENSP00000403701,9606.ENSP00000410402,9606.ENSP00000481780	MAP3K5,MAP3K7,ARRB2,MAP2K4,MAPK8IP3
GO Process	GO:0009612	Response to mechanical stimulus	4	214	1.52	0.0042	9606.ENSP00000229795,9606.ENSP00000378974,9606.ENSP00000382423,9606.ENSP00000410402	MAPK14,MAPK8,MAP3K1,MAP2K4
GO Proc	GO:0043065	Positive regulation of apoptotic	5	507	1.25	0.0042	9606.ENSP00000351908,9606.ENSP00000378974,9606.ENSP00000394560,9606.ENSP00000403701,9606.ENSP000	MAP3K5,MAPK8,MAPK9,ARRB2,MAP2K4

ess		process					00410402	
GO Process	GO:0033554	Cellular response to stress	7	1572	0.9	0.0043	9606.ENSP00000229795,9606.ENSP00000351908,9606.ENSP00000358335,9606.ENSP00000378974,9606.ENSP00000394560,9606.ENSP00000410402,9606.ENSP00000481780	MAPK14,MAP3K5,MAP3K7,MAPK8,MAPK9,MAP2K4,MAPK8IP3
GO Process	GO:0010660	Regulation of muscle cell apoptotic process	3	67	1.9	0.0052	9606.ENSP00000351908,9606.ENSP00000403701,9606.ENSP00000410402	MAP3K5,ARRB2,MAP2K4
GO Process	GO:1901701	Cellular response to oxygen-containing compound	6	1057	1.01	0.0063	9606.ENSP00000229795,9606.ENSP00000351908,9606.ENSP00000378974,9606.ENSP00000394560,9606.ENSP00000403701,9606.ENSP00000410402	MAPK14,MAP3K5,MAPK8,MAPK9,ARRB2,MAP2K4
GO Process	GO:0071260	Cellular response to mechanical stimulus	3	75	1.86	0.0066	9606.ENSP00000378974,9606.ENSP00000382423,9606.ENSP00000410402	MAPK8,MAP3K1,MAP2K4
GO Process	GO:0070887	Cellular response to chemical stimulus	8	2609	0.74	0.0068	9606.ENSP00000229795,9606.ENSP00000351908,9606.ENSP00000358335,9606.ENSP00000378974,9606.ENSP00000394560,9606.ENSP00000403701,9606.ENSP00000410402,9606.ENSP00000420213	MAPK14,MAP3K5,MAP3K7,MAPK8,MAPK9,ARRB2,MAP2K4,FLNB
GO Process	GO:0036211	Protein modification process	8	2674	0.73	0.0079	9606.ENSP00000229795,9606.ENSP00000351908,9606.ENSP00000358335,9606.ENSP00000378974,9606.ENSP00000382423,9606.ENSP00000394560,9606.ENSP00000403701,9606.ENSP00000410402	MAPK14,MAP3K5,MAP3K7,MAPK8,MAP3K1,MAPK9,ARRB2,MAP2K4
GO Process	GO:0046330	Positive regulation of JNK cascade	3	96	1.75	0.0100	9606.ENSP00000351908,9606.ENSP00000410402,9606.ENSP00000481780	MAP3K5,MAP2K4,MAPK8IP3
GO Process	GO:0048583	Regulation of response to stimulus	9	3931	0.61	0.0100	9606.ENSP00000229795,9606.ENSP00000351908,9606.ENSP00000358335,9606.ENSP00000378974,9606.ENSP00000382423,9606.ENSP00000394560,9606.ENSP00000403701,9606.ENSP00000410402,9606.ENSP00000481780	MAPK14,MAP3K5,MAP3K7,MAPK8,MAP3K1,MAPK9,ARRB2,MAP2K4,MAPK8IP3
GO Process	GO:0071496	Cellular response to external stimulus	4	317	1.35	0.0102	9606.ENSP00000351908,9606.ENSP00000378974,9606.ENSP00000382423,9606.ENSP00000410402	MAP3K5,MAPK8,MAP3K1,MAP2K4
GO Process	GO:0071214	Cellular response to abiotic stimulus	4	325	1.34	0.0109	9606.ENSP00000229795,9606.ENSP00000378974,9606.ENSP00000382423,9606.ENSP00000410402	MAPK14,MAPK8,MAP3K1,MAP2K4
GO Process	GO:0071310	Cellular response to organic substance	7	2019	0.79	0.0119	9606.ENSP00000229795,9606.ENSP00000351908,9606.ENSP00000358335,9606.ENSP00000378974,9606.ENSP00000403701,9606.ENSP00000410402,9606.ENSP0000042021	MAPK14,MAP3K5,MAP3K7,MAPK8,ARRB2,MAP2K4,FLNB

							3	
GO Process	GO:0007166	Cell surface receptor signaling pathway	7	2040	0.79	0.0124	9606.ENSP00000229795,9606.ENSP00000358335,9606.ENSP00000378974,9606.ENSP00000382423,9606.ENSP00000394560,9606.ENSP00000403701,9606.ENSP00000410402	MAPK14,MAP3K7,MAPK8,MAP3K1,MAPK9,ARRB2,MAP2K4
GO Process	GO:0034614	Cellular response to reactive oxygen species	3	118	1.66	0.0148	9606.ENSP00000351908,9606.ENSP00000378974,9606.ENSP00000394560	MAP3K5,MAPK8,MAPK9
GO Process	GO:0080134	Regulation of response to stress	6	1373	0.89	0.0159	9606.ENSP00000229795,9606.ENSP00000351908,9606.ENSP00000358335,9606.ENSP00000403701,9606.ENSP00000410402,9606.ENSP00000481780	MAPK14,MAP3K5,MAP3K7,ARRB2,MAP2K4,MAPK8IP3
GO Process	GO:0042981	Regulation of apoptotic process	6	1462	0.87	0.0210	9606.ENSP00000351908,9606.ENSP00000378974,9606.ENSP00000394560,9606.ENSP00000403701,9606.ENSP00000410402,9606.ENSP00000481780	MAP3K5,MAPK8,MAPK9,ARRB2,MAP2K4,MAPK8IP3
GO Process	GO:0051247	Positive regulation of protein metabolic process	6	1512	0.85	0.0237	9606.ENSP00000351908,9606.ENSP00000358335,9606.ENSP00000378974,9606.ENSP00000394560,9606.ENSP00000403701,9606.ENSP00000410402	MAP3K5,MAP3K7,MAPK8,MAPK9,ARRB2,MAP2K4
GO Process	GO:0006950	Response to stress	8	3358	0.63	0.0243	9606.ENSP00000229795,9606.ENSP00000351908,9606.ENSP00000358335,9606.ENSP00000378974,9606.ENSP00000394560,9606.ENSP00000410402,9606.ENSP00000420213,9606.ENSP00000481780	MAPK14,MAP3K5,MAP3K7,MAPK8,MAPK9,MAP2K4,FLNB,MAPK8IP3
GO Process	GO:0009605	Response to external stimulus	7	2355	0.73	0.0243	9606.ENSP00000229795,9606.ENSP00000351908,9606.ENSP00000378974,9606.ENSP00000382423,9606.ENSP00000403701,9606.ENSP00000410402,9606.ENSP00000420213	MAPK14,MAP3K5,MAPK8,MAP3K1,ARRB2,MAP2K4,FLNB
GO Process	GO:0009611	Response to wounding	4	444	1.21	0.0243	9606.ENSP00000229795,9606.ENSP00000351908,9606.ENSP00000410402,9606.ENSP00000481780	MAPK14,MAP3K5,MAP2K4,MAPK8IP3
GO Process	GO:0009967	Positive regulation of signal transduction	6	1525	0.85	0.0243	9606.ENSP00000351908,9606.ENSP00000358335,9606.ENSP00000394560,9606.ENSP00000403701,9606.ENSP00000410402,9606.ENSP00000481780	MAP3K5,MAP3K7,MAPK9,ARRB2,MAP2K4,MAPK8IP3
GO Process	GO:0018193	Peptidyl-amino acid modification	5	900	1.0	0.0243	9606.ENSP00000229795,9606.ENSP00000358335,9606.ENSP00000378974,9606.ENSP00000394560,9606.ENSP00000410402	MAPK14,MAP3K7,MAPK8,MAPK9,MAP2K4
GO Process	GO:0031399	Regulation of protein modification process	6	1560	0.84	0.0243	9606.ENSP00000351908,9606.ENSP00000358335,9606.ENSP00000378974,9606.ENSP00000394560,9606.ENSP00000403701,9606.ENSP00000410402	MAP3K5,MAP3K7,MAPK8,MAPK9,ARRB2,MAP2K4

GO Process	GO:0051090	Regulation of DNA-binding transcription factor activity	4	436	1.22	0.0243	9606.ENSP00000358335,9606.ENSP00000378974,9606.ENSP00000394560,9606.ENSP00000403701	MAP3K7,MAPK8,MAPK9,ARRB2
GO Process	GO:0034391	Regulation of smooth muscle cell apoptotic process	2	23	2.19	0.0245	9606.ENSP00000403701,9606.ENSP00000410402	ARRB2,MAP2K4
GO Process	GO:0007165	Signal transduction	9	4714	0.53	0.0250	9606.ENSP00000229795,9606.ENSP00000351908,9606.ENSP00000358335,9606.ENSP00000378974,9606.ENSP00000382423,9606.ENSP00000394560,9606.ENSP00000403701,9606.ENSP00000410402,9606.ENSP00000420213	MAPK14,MAP3K5,MAP3K7,MAPK8,MAP3K1,MAPK9,ARRB2,MAP2K4,FLNB
GO Process	GO:0051716	Cellular response to stimulus	10	6357	0.45	0.0268	9606.ENSP00000229795,9606.ENSP00000351908,9606.ENSP00000358335,9606.ENSP00000378974,9606.ENSP00000382423,9606.ENSP00000394560,9606.ENSP00000403701,9606.ENSP00000410402,9606.ENSP00000420213,9606.ENSP00000481780	MAPK14,MAP3K5,MAP3K7,MAPK8,MAP3K1,MAPK9,ARRB2,MAP2K4,FLNB,MAPK8IP3
GO Process	GO:0010661	Positive regulation of muscle cell apoptotic process	2	25	2.16	0.0271	9606.ENSP00000351908,9606.ENSP00000410402	MAP3K5,MAP2K4
GO Process	GO:0061061	Muscle structure development	4	498	1.16	0.0311	9606.ENSP00000229795,9606.ENSP00000327145,9606.ENSP00000410402,9606.ENSP00000420213	MAPK14,FLNC,MAP2K4,FLNB
GO Process	GO:0031401	Positive regulation of protein modification process	5	1018	0.94	0.0325	9606.ENSP00000351908,9606.ENSP00000358335,9606.ENSP00000394560,9606.ENSP00000403701,9606.ENSP00000410402	MAP3K5,MAP3K7,MAPK9,ARRB2,MAP2K4
GO Process	GO:1902531	Regulation of intracellular signal transduction	6	1726	0.79	0.0342	9606.ENSP00000229795,9606.ENSP00000351908,9606.ENSP00000358335,9606.ENSP00000403701,9606.ENSP00000410402,9606.ENSP00000481780	MAPK14,MAP3K5,MAP3K7,ARRB2,MAP2K4,MAPK8IP3
GO Process	GO:0006915	Apoptotic process	5	1041	0.93	0.0345	9606.ENSP00000229795,9606.ENSP00000351908,9606.ENSP00000358335,9606.ENSP00000394560,9606.ENSP00000410402	MAPK14,MAP3K5,MAP3K7,MAPK9,MAP2K4
GO Process	GO:0018105	Peptidyl-serine phosphorylation	3	193	1.44	0.0345	9606.ENSP00000229795,9606.ENSP00000378974,9606.ENSP00000394560	MAPK14,MAPK8,MAPK9
GO Process	GO:0007178	Transmembrane receptor protein serine/threonine kinase signaling	3	206	1.42	0.0391	9606.ENSP00000229795,9606.ENSP00000358335,9606.ENSP00000403701	MAPK14,MAP3K7,ARRB2

		pathway						
GO Process	GO:0051146	Striated muscle cell differentiation	3	204	1.42	0.0391	9606.ENSP00000229795,9606.ENSP00000327145,9606.ENSP00000410402	MAPK14,FLNC,MAP2K4
GO Process	GO:0009628	Response to abiotic stimulus	5	1107	0.91	0.0406	9606.ENSP00000229795,9606.ENSP00000378974,9606.ENSP00000382423,9606.ENSP00000403701,9606.ENSP00000410402	MAPK14,MAPK8,MAP3K1,ARRB2,MAP2K4
GO Process	GO:0071276	Cellular response to cadmium ion	2	36	2.0	0.0406	9606.ENSP00000378974,9606.ENSP00000394560	MAPK8,MAPK9
GO Process	GO:0010628	Positive regulation of gene expression	5	1146	0.89	0.0448	9606.ENSP00000229795,9606.ENSP00000358335,9606.ENSP00000378974,9606.ENSP00000394560,9606.ENSP00000403701	MAPK14,MAP3K7,MAPK8,MAPK9,ARRB2
GO Process	GO:0043507	Positive regulation of JUN kinase activity	2	42	1.93	0.0500	9606.ENSP00000351908,9606.ENSP00000358335	MAP3K5,MAP3K7
GO Function	GO:0004707	MAP kinase activity	4	15	2.68	9.99e-07	9606.ENSP00000229795,9606.ENSP00000358335,9606.ENSP00000378974,9606.ENSP00000394560	MAPK14,MAP3K7,MAPK8,MAPK9
GO Function	GO:0106310	Protein serine kinase activity	7	361	1.54	9.99e-07	9606.ENSP00000229795,9606.ENSP00000351908,9606.ENSP00000358335,9606.ENSP00000378974,9606.ENSP00000382423,9606.ENSP00000394560,9606.ENSP00000410402	MAPK14,MAP3K5,MAP3K7,MAPK8,MAP3K1,MAPK9,MAP2K4
GO Function	GO:0004674	Protein serine/threonine kinase activity	7	434	1.46	1.34e-06	9606.ENSP00000229795,9606.ENSP00000351908,9606.ENSP00000358335,9606.ENSP00000378974,9606.ENSP00000382423,9606.ENSP00000394560,9606.ENSP00000410402	MAPK14,MAP3K5,MAP3K7,MAPK8,MAP3K1,MAPK9,MAP2K4
GO Function	GO:0019901	Protein kinase binding	7	702	1.25	1.97e-05	9606.ENSP00000229795,9606.ENSP00000351908,9606.ENSP00000358335,9606.ENSP00000378974,9606.ENSP00000382423,9606.ENSP00000403701,9606.ENSP00000481780	MAPK14,MAP3K5,MAP3K7,MAPK8,MAP3K1,ARRB2,MAPK8IP3
GO Function	GO:0004709	MAP kinase kinase kinase activity	3	27	2.3	0.00026	9606.ENSP00000351908,9606.ENSP00000358335,9606.ENSP00000382423	MAP3K5,MAP3K7,MAP3K1
GO Functio	GO:0004705	JUN kinase activity	2	2	3.25	0.00076	9606.ENSP00000378974,9606.ENSP00000394560	MAPK8,MAPK9

n								
GO Function	GO:0004712	Protein serine/threonine/tyrosine kinase activity	3	44	2.09	0.00085	9606.ENSP00000229795,9606.ENSP00000394560,9606.ENSP00000410402	MAPK14,MAPK9,MAP2K4
GO Function	GO:0005524	ATP binding	7	1491	0.92	0.0014	9606.ENSP00000229795,9606.ENSP00000351908,9606.ENSP00000358335,9606.ENSP00000378974,9606.ENSP00000382423,9606.ENSP00000394560,9606.ENSP00000410402	MAPK14,MAP3K5,MAP3K7,MAPK8,MAP3K1,MAPK9,MAP2K4
GO Function	GO:0004708	MAP kinase kinase activity	2	17	2.32	0.0103	9606.ENSP00000229795,9606.ENSP00000410402	MAPK14,MAP2K4
GO Function	GO:0019903	Protein phosphatase binding	3	152	1.55	0.0137	9606.ENSP00000229795,9606.ENSP00000351908,9606.ENSP00000378974	MAPK14,MAP3K5,MAPK8
GO Function	GO:0051019	Mitogen-activated protein kinase binding	2	27	2.12	0.0191	9606.ENSP00000229795,9606.ENSP00000403701	MAPK14,ARRB2
GO Component	GO:0005829	Cytosol	10	5438	0.52	0.0436	9606.ENSP00000229795,9606.ENSP00000327145,9606.ENSP00000351908,9606.ENSP00000358335,9606.ENSP00000378974,9606.ENSP00000382423,9606.ENSP00000394560,9606.ENSP00000403701,9606.ENSP00000410402,9606.ENSP00000420213	MAPK14,FLNC,MAP3K5,MAP3K7,MAPK8,MAP3K1,MAPK9,ARRB2,MAP2K4,FLNB
STRING clusters	CL:18406	Mixed, incl. Cellular response to sorbitol, and Activation of the AP-1 family of transcription factors	6	15	2.86	1.40e-12	9606.ENSP00000229795,9606.ENSP00000378974,9606.ENSP00000382423,9606.ENSP00000394560,9606.ENSP00000410402,9606.ENSP00000481780	MAPK14,MAPK8,MAP3K1,MAPK9,MAP2K4,MAPK8IP3
STRING clusters	CL:18408	JUN kinase activity, and JUN kinase kinase activity	5	5	3.25	7.16e-12	9606.ENSP00000378974,9606.ENSP00000382423,9606.ENSP00000394560,9606.ENSP00000410402,9606.ENSP00000481780	MAPK8,MAP3K1,MAPK9,MAP2K4,MAPK8IP3
STRING	CL:18125	Filamin family, and Von	2	5	2.86	0.0025	9606.ENSP00000327145,9606.ENSP00000420213	FLNC,FLNB

clusters		Willebrand disease						
KEGG	hsa04010	MAPK signaling pathway	11	286	1.84	2.54e-18	9606.ENSP00000229795,9606.ENSP00000327145,9606.ENSP00000351908,9606.ENSP00000358335,9606.ENSP00000378974,9606.ENSP00000382423,9606.ENSP00000394560,9606.ENSP00000403701,9606.ENSP00000410402,9606.ENSP00000420213,9606.ENSP00000481780	MAPK14,FLNC,MAP3K5,MAP3K7,MAPK8,MAP3K1,MAPK9,ARRB2,MAP2K4,FLNB,MAPK8IP3
KEGG	hsa05132	Salmonella infection	7	209	1.78	9.20e-10	9606.ENSP00000229795,9606.ENSP00000327145,9606.ENSP00000358335,9606.ENSP00000378974,9606.ENSP00000394560,9606.ENSP00000410402,9606.ENSP00000420213	MAPK14,FLNC,MAP3K7,MAPK8,MAPK9,MAP2K4,FLNB
KEGG	hsa04668	TNF signaling pathway	6	111	1.99	1.94e-09	9606.ENSP00000229795,9606.ENSP00000351908,9606.ENSP00000358335,9606.ENSP00000378974,9606.ENSP00000394560,9606.ENSP00000410402	MAPK14,MAP3K5,MAP3K7,MAPK8,MAPK9,MAP2K4
KEGG	hsa05418	Fluid shear stress and atherosclerosis	6	129	1.92	3.48e-09	9606.ENSP00000229795,9606.ENSP00000351908,9606.ENSP00000358335,9606.ENSP00000378974,9606.ENSP00000394560,9606.ENSP00000410402	MAPK14,MAP3K5,MAP3K7,MAPK8,MAPK9,MAP2K4
KEGG	hsa05161	Hepatitis B	6	158	1.83	9.08e-09	9606.ENSP00000229795,9606.ENSP00000358335,9606.ENSP00000378974,9606.ENSP00000382423,9606.ENSP00000394560,9606.ENSP00000410402	MAPK14,MAP3K7,MAPK8,MAP3K1,MAPK9,MAP2K4
KEGG	hsa04622	RIG-I-like receptor signaling pathway	5	69	2.11	1.65e-08	9606.ENSP00000229795,9606.ENSP00000358335,9606.ENSP00000378974,9606.ENSP00000382423,9606.ENSP00000394560	MAPK14,MAP3K7,MAPK8,MAP3K1,MAPK9
KEGG	hsa04912	GnRH signaling pathway	5	87	2.01	4.31e-08	9606.ENSP00000229795,9606.ENSP00000378974,9606.ENSP00000382423,9606.ENSP00000394560,9606.ENSP00000410402	MAPK14,MAPK8,MAP3K1,MAPK9,MAP2K4
KEGG	hsa04620	Toll-like receptor signaling pathway	5	100	1.95	7.37e-08	9606.ENSP00000229795,9606.ENSP00000358335,9606.ENSP00000378974,9606.ENSP00000394560,9606.ENSP00000410402	MAPK14,MAP3K7,MAPK8,MAPK9,MAP2K4
KEGG	hsa04722	Neurotrophin signaling pathway	5	112	1.9	1.13e-07	9606.ENSP00000229795,9606.ENSP00000351908,9606.ENSP00000378974,9606.ENSP00000382423,9606.ENSP00000394560	MAPK14,MAP3K5,MAPK8,MAP3K1,MAPK9
KEGG	hsa04935	Growth hormone synthesis, secretion and action	5	117	1.88	1.26e-07	9606.ENSP00000229795,9606.ENSP00000378974,9606.ENSP00000382423,9606.ENSP00000394560,9606.ENSP00000410402	MAPK14,MAPK8,MAP3K1,MAPK9,MAP2K4
KEGG	hsa04926	Relaxin signaling pathway	5	126	1.85	1.52e-07	9606.ENSP00000229795,9606.ENSP00000378974,9606.ENSP00000394560,9606.ENSP00000403701,9606.ENSP00000410402	MAPK14,MAPK8,MAPK9,ARRB2,MAP2K4
KE	hsa05135	Yersinia infection	5	124	1.86	1.52e-	9606.ENSP00000229795,9606.ENSP00000358335,9606.E	MAPK14,MAP3K7,MAPK

GG						07	NSP00000378974,9606.ENSP00000394560,9606.ENSP00000410402	8,MAPK9,MAP2K4
KEGG	hsa04664	Fc epsilon RI signaling pathway	4	65	2.04	1.08e-06	9606.ENSP00000229795,9606.ENSP00000378974,9606.ENSP00000394560,9606.ENSP00000410402	MAPK14,MAPK8,MAPK9,MAP2K4
KEGG	hsa05120	Epithelial cell signaling in Helicobacter pylori infection	4	65	2.04	1.08e-06	9606.ENSP00000229795,9606.ENSP00000378974,9606.ENSP00000394560,9606.ENSP00000410402	MAPK14,MAPK8,MAPK9,MAP2K4
KEGG	hsa05169	Epstein-Barr virus infection	5	192	1.67	1.08e-06	9606.ENSP00000229795,9606.ENSP00000358335,9606.ENSP00000378974,9606.ENSP00000394560,9606.ENSP00000410402	MAPK14,MAP3K7,MAPK8,MAPK9,MAP2K4
KEGG	hsa04657	IL-17 signaling pathway	4	91	1.9	3.42e-06	9606.ENSP00000229795,9606.ENSP00000358335,9606.ENSP00000378974,9606.ENSP00000394560	MAPK14,MAP3K7,MAPK8,MAPK9
KEGG	hsa05142	Chagas disease	4	97	1.87	4.12e-06	9606.ENSP00000229795,9606.ENSP00000378974,9606.ENSP00000394560,9606.ENSP00000410402	MAPK14,MAPK8,MAPK9,MAP2K4
KEGG	hsa04660	T cell receptor signaling pathway	4	100	1.86	4.38e-06	9606.ENSP00000229795,9606.ENSP00000358335,9606.ENSP00000378974,9606.ENSP00000394560	MAPK14,MAP3K7,MAPK8,MAPK9
KEGG	hsa05145	Toxoplasmosis	4	103	1.84	4.65e-06	9606.ENSP00000229795,9606.ENSP00000358335,9606.ENSP00000378974,9606.ENSP00000394560	MAPK14,MAP3K7,MAPK8,MAPK9
KEGG	hsa04071	Sphingolipid signaling pathway	4	116	1.79	7.01e-06	9606.ENSP00000229795,9606.ENSP00000351908,9606.ENSP00000378974,9606.ENSP00000394560	MAPK14,MAP3K5,MAPK8,MAPK9
KEGG	hsa04380	Osteoclast differentiation	4	120	1.78	7.61e-06	9606.ENSP00000229795,9606.ENSP00000358335,9606.ENSP00000378974,9606.ENSP00000394560	MAPK14,MAP3K7,MAPK8,MAPK9
KEGG	hsa04728	Dopaminergic synapse	4	126	1.75	8.79e-06	9606.ENSP00000229795,9606.ENSP00000378974,9606.ENSP00000394560,9606.ENSP00000403701	MAPK14,MAPK8,MAPK9,ARRB2
KEGG	hsa04530	Tight junction	4	157	1.66	1.98e-05	9606.ENSP00000351908,9606.ENSP00000378974,9606.ENSP00000382423,9606.ENSP00000394560	MAP3K5,MAPK8,MAP3K1,MAPK9
KEGG	hsa04621	NOD-like receptor signaling pathway	4	173	1.62	2.76e-05	9606.ENSP00000229795,9606.ENSP00000358335,9606.ENSP00000378974,9606.ENSP00000394560	MAPK14,MAP3K7,MAPK8,MAPK9
KEGG	hsa05130	Pathogenic Escherichia coli infection	4	187	1.58	3.59e-05	9606.ENSP00000229795,9606.ENSP00000358335,9606.ENSP00000378974,9606.ENSP00000394560	MAPK14,MAP3K7,MAPK8,MAPK9
KEGG	hsa05167	Kaposi sarcoma-associated herpesvirus infection	4	187	1.58	3.59e-05	9606.ENSP00000229795,9606.ENSP00000378974,9606.ENSP00000394560,9606.ENSP00000410402	MAPK14,MAPK8,MAPK9,MAP2K4
KEGG	hsa04510	Focal adhesion	4	195	1.57	3.92e-05	9606.ENSP00000327145,9606.ENSP00000378974,9606.ENSP00000394560,9606.ENSP00000420213	FLNC,MAPK8,MAPK9,FLNB
KE	hsa05170	Human	4	203	1.55	4.42e-	9606.ENSP00000229795,9606.ENSP00000358335,9606.E	MAPK14,MAP3K7,MAPK

GG		immunodeficiency virus 1 infection				05	NSP00000378974,9606.ENSP00000394560	8,MAPK9
KEGG	hsa05166	Human T-cell leukemia virus 1 infection	4	210	1.53	4.87e-05	9606.ENSP00000378974,9606.ENSP00000382423,9606.ENSP00000394560,9606.ENSP00000410402	MAPK8,MAP3K1,MAPK9,MAP2K4
KEGG	hsa05131	Shigellosis	4	218	1.52	5.44e-05	9606.ENSP00000229795,9606.ENSP00000358335,9606.ENSP00000378974,9606.ENSP00000394560	MAPK14,MAP3K7,MAPK8,MAPK9
KEGG	hsa04917	Prolactin signaling pathway	3	68	1.9	7.85e-05	9606.ENSP00000229795,9606.ENSP00000378974,9606.ENSP00000394560	MAPK14,MAPK8,MAPK9
KEGG	hsa05133	Pertussis	3	73	1.87	9.34e-05	9606.ENSP00000229795,9606.ENSP00000378974,9606.ENSP00000394560	MAPK14,MAPK8,MAPK9
KEGG	hsa04012	ErbB signaling pathway	3	81	1.82	0.00012	9606.ENSP00000378974,9606.ENSP00000394560,9606.ENSP00000410402	MAPK8,MAPK9,MAP2K4
KEGG	hsa04658	Th1 and Th2 cell differentiation	3	85	1.8	0.00014	9606.ENSP00000229795,9606.ENSP00000378974,9606.ENSP00000394560	MAPK14,MAPK8,MAPK9
KEGG	hsa01522	Endocrine resistance	3	94	1.76	0.00017	9606.ENSP00000229795,9606.ENSP00000378974,9606.ENSP00000394560	MAPK14,MAPK8,MAPK9
KEGG	hsa04750	Inflammatory mediator regulation of TRP channels	3	92	1.77	0.00017	9606.ENSP00000229795,9606.ENSP00000378974,9606.ENSP00000394560	MAPK14,MAPK8,MAPK9
KEGG	hsa04914	Progesterone-mediated oocyte maturation	3	95	1.75	0.00017	9606.ENSP00000229795,9606.ENSP00000378974,9606.ENSP00000394560	MAPK14,MAPK8,MAPK9
KEGG	hsa04933	AGE-RAGE signaling pathway in diabetic complications	3	96	1.75	0.00017	9606.ENSP00000229795,9606.ENSP00000378974,9606.ENSP00000394560	MAPK14,MAPK8,MAPK9
KEGG	hsa04625	C-type lectin receptor signaling pathway	3	101	1.73	0.00019	9606.ENSP00000229795,9606.ENSP00000378974,9606.ENSP00000394560	MAPK14,MAPK8,MAPK9
KEGG	hsa04659	Th17 cell differentiation	3	99	1.73	0.00019	9606.ENSP00000229795,9606.ENSP00000378974,9606.ENSP00000394560	MAPK14,MAPK8,MAPK9
KEGG	hsa04068	FoxO signaling pathway	3	126	1.63	0.00036	9606.ENSP00000229795,9606.ENSP00000378974,9606.ENSP00000394560	MAPK14,MAPK8,MAPK9
KEGG	hsa04140	Autophagy - animal	3	131	1.61	0.00039	9606.ENSP00000358335,9606.ENSP00000378974,9606.ENSP00000394560	MAP3K7,MAPK8,MAPK9
KEGG	hsa04210	Apoptosis	3	131	1.61	0.00039	9606.ENSP00000351908,9606.ENSP00000378974,9606.ENSP00000394560	MAP3K5,MAPK8,MAPK9
KE	hsa05017	Spinocerebellar	3	135	1.6	0.0004	9606.ENSP00000351908,9606.ENSP00000378974,9606.E	MAP3K5,MAPK8,MAPK9

GG		ataxia				1	NSP00000394560	
KEGG	hsa05162	Measles	3	137	1.59	0.00041	9606.ENSP00000358335,9606.ENSP00000378974,9606.ENSP00000394560	MAP3K7,MAPK8,MAPK9
KEGG	hsa04723	Retrograde endocannabinoid signaling	3	142	1.58	0.00045	9606.ENSP00000229795,9606.ENSP00000378974,9606.ENSP00000394560	MAPK14,MAPK8,MAPK9
KEGG	hsa04932	Non-alcoholic fatty liver disease	3	146	1.57	0.00048	9606.ENSP00000351908,9606.ENSP00000378974,9606.ENSP00000394560	MAP3K5,MAPK8,MAPK9
KEGG	hsa04310	Wnt signaling pathway	3	154	1.54	0.00055	9606.ENSP00000358335,9606.ENSP00000378974,9606.ENSP00000394560	MAP3K7,MAPK8,MAPK9
KEGG	hsa04141	Protein processing in endoplasmic reticulum	3	163	1.52	0.00063	9606.ENSP00000351908,9606.ENSP00000378974,9606.ENSP00000394560	MAP3K5,MAPK8,MAPK9
KEGG	hsa05152	Tuberculosis	3	165	1.51	0.00064	9606.ENSP00000229795,9606.ENSP00000378974,9606.ENSP00000394560	MAPK14,MAPK8,MAPK9
KEGG	hsa04215	Apoptosis - multiple species	2	30	2.08	0.00092	9606.ENSP00000378974,9606.ENSP00000394560	MAPK8,MAPK9
KEGG	hsa05205	Proteoglycans in cancer	3	194	1.44	0.00099	9606.ENSP00000229795,9606.ENSP00000327145,9606.ENSP00000420213	MAPK14,FLNC,FLNB
KEGG	hsa05012	Parkinson disease	3	236	1.36	0.0017	9606.ENSP00000351908,9606.ENSP00000378974,9606.ENSP00000394560	MAP3K5,MAPK8,MAPK9
KEGG	hsa04930	Type II diabetes mellitus	2	45	1.9	0.0019	9606.ENSP00000378974,9606.ENSP00000394560	MAPK8,MAPK9
KEGG	hsa05020	Prion disease	3	263	1.31	0.0023	9606.ENSP00000229795,9606.ENSP00000378974,9606.ENSP00000394560	MAPK14,MAPK8,MAPK9
KEGG	hsa05016	Huntington disease	3	295	1.26	0.0031	9606.ENSP00000351908,9606.ENSP00000378974,9606.ENSP00000394560	MAP3K5,MAPK8,MAPK9
KEGG	hsa04137	Mitophagy - animal	2	64	1.75	0.0035	9606.ENSP00000378974,9606.ENSP00000394560	MAPK8,MAPK9
KEGG	hsa04920	Adipocytokine signaling pathway	2	68	1.72	0.0039	9606.ENSP00000378974,9606.ENSP00000394560	MAPK8,MAPK9
KEGG	hsa05140	Leishmaniasis	2	69	1.72	0.0039	9606.ENSP00000229795,9606.ENSP00000358335	MAPK14,MAP3K7
KEGG	hsa05212	Pancreatic cancer	2	71	1.7	0.0041	9606.ENSP00000378974,9606.ENSP00000394560	MAPK8,MAPK9
KEGG	hsa05010	Alzheimer disease	3	354	1.18	0.0048	9606.ENSP00000351908,9606.ENSP00000378974,9606.ENSP00000394560	MAP3K5,MAPK8,MAPK9
KEGG	hsa05210	Colorectal cancer	2	82	1.64	0.0052	9606.ENSP00000378974,9606.ENSP00000394560	MAPK8,MAPK9

KEGG	hsa05231	Choline metabolism in cancer	2	95	1.58	0.0068	9606.ENSP00000378974,9606.ENSP00000394560	MAPK8,MAPK9
KEGG	hsa04931	Insulin resistance	2	106	1.53	0.0083	9606.ENSP00000378974,9606.ENSP00000394560	MAPK8,MAPK9
KEGG	hsa04910	Insulin signaling pathway	2	132	1.43	0.0125	9606.ENSP00000378974,9606.ENSP00000394560	MAPK8,MAPK9
KEGG	hsa04217	Necroptosis	2	147	1.39	0.0152	9606.ENSP00000378974,9606.ENSP00000394560	MAPK8,MAPK9
KEGG	hsa04024	cAMP signaling pathway	2	207	1.24	0.0290	9606.ENSP00000378974,9606.ENSP00000394560	MAPK8,MAPK9
KEGG	hsa04014	Ras signaling pathway	2	225	1.2	0.0335	9606.ENSP00000378974,9606.ENSP00000394560	MAPK8,MAPK9
KEGG	hsa04714	Thermogenesis	2	226	1.2	0.0335	9606.ENSP00000229795,9606.ENSP00000351908	MAPK14,MAP3K5
Reactome	HSA-166058	MyD88:MAL(TIRAP) cascade initiated on plasma membrane	6	109	1.99	1.42e-08	9606.ENSP00000229795,9606.ENSP00000358335,9606.ENSP00000378974,9606.ENSP00000382423,9606.ENSP00000394560,9606.ENSP00000410402	MAPK14,MAP3K7,MAPK8,MAP3K1,MAPK9,MAP2K4
Reactome	HSA-975138	TRAF6 mediated induction of NFkB and MAP kinases upon TLR7/8 or 9 activation	6	98	2.04	1.42e-08	9606.ENSP00000229795,9606.ENSP00000358335,9606.ENSP00000378974,9606.ENSP00000382423,9606.ENSP00000394560,9606.ENSP00000410402	MAPK14,MAP3K7,MAPK8,MAP3K1,MAPK9,MAP2K4
Reactome	HSA-975871	MyD88 cascade initiated on plasma membrane	6	93	2.06	1.42e-08	9606.ENSP00000229795,9606.ENSP00000358335,9606.ENSP00000378974,9606.ENSP00000382423,9606.ENSP00000394560,9606.ENSP00000410402	MAPK14,MAP3K7,MAPK8,MAP3K1,MAPK9,MAP2K4
Reactome	HSA-450294	MAP kinase activation	5	61	2.17	2.68e-08	9606.ENSP00000229795,9606.ENSP00000358335,9606.ENSP00000378974,9606.ENSP00000394560,9606.ENSP00000410402	MAPK14,MAP3K7,MAPK8,MAPK9,MAP2K4
Reactome	HSA-450321	JNK (c-Jun kinases) phosphorylation and activation mediated by activated human TAK1	4	20	2.55	7.91e-08	9606.ENSP00000358335,9606.ENSP00000378974,9606.ENSP00000394560,9606.ENSP00000410402	MAP3K7,MAPK8,MAPK9,MAP2K4
Reacto	HSA-2559580	Oxidative Stress Induced	5	92	1.99	1.58e-07	9606.ENSP00000229795,9606.ENSP00000351908,9606.ENSP00000378974,9606.ENSP00000394560,9606.ENSP000	MAPK14,MAP3K5,MAPK8,MAPK9,MAP2K4

me		Senescence					00410402	
Reactome	HSA-2871796	FCERI mediated MAPK activation	4	34	2.32	4.17e-07	9606.ENSP00000378974,9606.ENSP00000382423,9606.ENSP00000394560,9606.ENSP00000410402	MAPK8,MAP3K1,MAPK9,MAP2K4
Reactome	HSA-2454202	Fc epsilon receptor (FCERI) signaling	5	131	1.83	6.74e-07	9606.ENSP00000358335,9606.ENSP00000378974,9606.ENSP00000382423,9606.ENSP00000394560,9606.ENSP00000410402	MAP3K7,MAPK8,MAP3K1,MAPK9,MAP2K4
Reactome	HSA-450341	Activation of the AP-1 family of transcription factors	3	9	2.78	2.70e-06	9606.ENSP00000229795,9606.ENSP00000378974,9606.ENSP00000394560	MAPK14,MAPK8,MAPK9
Reactome	HSA-1280215	Cytokine Signaling in Immune system	6	706	1.18	7.56e-05	9606.ENSP00000229795,9606.ENSP00000358335,9606.ENSP00000378974,9606.ENSP00000394560,9606.ENSP00000410402,9606.ENSP00000420213	MAPK14,MAP3K7,MAPK8,MAPK9,MAP2K4,FLNB
Reactome	HSA-376172	DSCAM interactions	2	11	2.51	0.0017	9606.ENSP00000229795,9606.ENSP00000378974	MAPK14,MAPK8
Reactome	HSA-168256	Immune System	7	1979	0.8	0.0018	9606.ENSP00000229795,9606.ENSP00000358335,9606.ENSP00000378974,9606.ENSP00000382423,9606.ENSP00000394560,9606.ENSP00000410402,9606.ENSP00000420213	MAPK14,MAP3K7,MAPK8,MAP3K1,MAPK9,MAP2K4,FLNB
Reactome	HSA-446652	Interleukin-1 family signaling	3	149	1.56	0.0049	9606.ENSP00000358335,9606.ENSP00000378974,9606.ENSP00000410402	MAP3K7,MAPK8,MAP2K4
Reactome	HSA-450302	Activated TAK1 mediates p38 MAPK activation	2	22	2.21	0.0052	9606.ENSP00000229795,9606.ENSP00000358335	MAPK14,MAP3K7
Reactome	HSA-168638	NOD1/2 Signaling Pathway	2	35	2.01	0.0122	9606.ENSP00000229795,9606.ENSP00000358335	MAPK14,MAP3K7

Table 7. Pathway Enrichment Analysis for GO, KEGG, STRING clusters and Reactome Terms within MKNK2 gene

#category	term ID	term description	observed gene	background gene	strength	false discovery	matching proteins in your network (IDs)	matching proteins in your network (labels)

			count	count	gth	rate		
GO Process	GO:0010608	Post-transcriptional regulation of gene expression	8	546	1.42	8.90e-07	9606.ENSP00000229795,9606.ENSP00000250896,9606.ENSP00000258416,9606.ENSP00000336790,9606.ENSP00000416255,9606.ENSP00000425561,9606.ENSP00000497422,9606.ENSP00000498083	MAPK14,MKNK2,EIF4E2,ATF4,EIF4G1,EIF4E,EIF4E1B,MKNK1
GO Process	GO:0006417	Regulation of translation	7	456	1.44	9.00e-06	9606.ENSP00000250896,9606.ENSP00000258416,9606.ENSP00000336790,9606.ENSP00000416255,9606.ENSP00000425561,9606.ENSP00000497422,9606.ENSP00000498083	MKNK2,EIF4E2,ATF4,EIF4G1,EIF4E,EIF4E1B,MKNK1
GO Process	GO:0030324	Lung development	5	180	1.7	9.55e-05	9606.ENSP00000215832,9606.ENSP00000263025,9606.ENSP00000345728,9606.ENSP00000416255,9606.ENSP00000425561	MAPK1,MAPK3,ATP7A,EIF4G1,EIF4E
GO Process	GO:0031326	Regulation of cellular biosynthetic process	11	4143	0.68	9.55e-05	9606.ENSP00000215832,9606.ENSP00000229795,9606.ENSP00000250896,9606.ENSP00000258416,9606.ENSP00000263025,9606.ENSP00000336790,9606.ENSP00000345728,9606.ENSP00000416255,9606.ENSP00000425561,9606.ENSP00000497422,9606.ENSP00000498083	MAPK1,MAPK14,MKNK2,EIF4E2,MAPK3,ATF4,ATP7A,EIF4G1,EIF4E,EIF4E1B,MKNK1
GO Process	GO:0009059	Macromolecule biosynthetic process	8	1394	1.01	0.00012	9606.ENSP00000229795,9606.ENSP00000258416,9606.ENSP00000263025,9606.ENSP00000336790,9606.ENSP00000345728,9606.ENSP00000416255,9606.ENSP00000425561,9606.ENSP00000497422	MAPK14,EIF4E2,MAPK3,ATF4,ATP7A,EIF4G1,EIF4E,EIF4E1B
GO Process	GO:0010468	Regulation of gene expression	11	4899	0.6	0.00030	9606.ENSP00000215832,9606.ENSP00000229795,9606.ENSP00000250896,9606.ENSP00000258416,9606.ENSP00000263025,9606.ENSP00000336790,9606.ENSP00000345728,9606.ENSP00000416255,9606.ENSP00000425561,9606.ENSP00000497422,9606.ENSP00000498083	MAPK1,MAPK14,MKNK2,EIF4E2,MAPK3,ATF4,ATP7A,EIF4G1,EIF4E,EIF4E1B,MKNK1
GO Process	GO:1901564	Organonitrogen compound metabolic process	11	4981	0.6	0.00033	9606.ENSP00000215832,9606.ENSP00000229795,9606.ENSP00000250896,9606.ENSP00000258416,9606.ENSP00000263025,9606.ENSP00000336790,9606.ENSP00000345728,9606.ENSP00000416255,9606.ENSP00000425561,9606.ENSP00000497422,9606.ENSP00000498083	MAPK1,MAPK14,MKNK2,EIF4E2,MAPK3,ATF4,ATP7A,EIF4G1,EIF4E,EIF4E1B,MKNK1
GO Process	GO:0051246	Regulation of protein metabolic process	9	2622	0.79	0.00063	9606.ENSP00000215832,9606.ENSP00000250896,9606.ENSP00000258416,9606.ENSP00000263025,9606.ENSP00000336790,9606.ENSP00000416255,9606.ENSP00000425561,9606.ENSP00000497422,9606.ENSP00000498083	MAPK1,MKNK2,EIF4E2,MAPK3,ATF4,EIF4G1,EIF4E,EIF4E1B,MKNK1
GO Process	GO:0019538	Protein metabolic process	10	3910	0.66	0.00089	9606.ENSP00000215832,9606.ENSP00000229795,9606.ENSP00000250896,9606.ENSP00000258416,9606.ENSP00000263025,9606.ENSP00000345728,9606.ENSP00000416255,9606.ENSP00000425561,9606.ENSP00000497422,9606.ENSP00000498083	MAPK1,MAPK14,MKNK2,EIF4E2,MAPK3,ATP7A,EIF4G1,EIF4E,EIF4E1B,MKNK1
GO	GO	Hematopoietic or	6	705	1.	0.000	9606.ENSP00000215832,9606.ENSP00000229795,9606.ENSP00000250896,9	MAPK1,MAPK14,MKN

Process	:0048534	lymphoid organ development			18	89	606.ENSP00000263025,9606.ENSP00000336790,9606.ENSP00000345728	K2,MAPK3,ATF4,ATP7A
GO Process	GO:0010556	Regulation of macromolecule biosynthetic process	10	3980	0.65	0.00095	9606.ENSP00000215832,9606.ENSP00000229795,9606.ENSP00000250896,9606.ENSP00000258416,9606.ENSP00000263025,9606.ENSP00000336790,9606.ENSP00000416255,9606.ENSP00000425561,9606.ENSP00000497422,9606.ENSP00000498083	MAPK1,MAPK14,MKNK2,EIF4E2,MAPK3,ATF4,EIF4G1,EIF4E,EIF4E1B,MKNK1
GO Process	GO:0031663	Lipopolysaccharide-mediated signaling pathway	3	39	2.14	0.0010	9606.ENSP00000215832,9606.ENSP00000229795,9606.ENSP00000263025	MAPK1,MAPK14,MAPK3
GO Process	GO:0043170	Macromolecule metabolic process	11	5781	0.53	0.0010	9606.ENSP00000215832,9606.ENSP00000229795,9606.ENSP00000250896,9606.ENSP00000258416,9606.ENSP00000263025,9606.ENSP00000336790,9606.ENSP00000345728,9606.ENSP00000416255,9606.ENSP00000425561,9606.ENSP00000497422,9606.ENSP00000498083	MAPK1,MAPK14,MKNK2,EIF4E2,MAPK3,ATF4,ATP7A,EIF4G1,EIF4E,EIF4E1B,MKNK1
GO Process	GO:0071276	Cellular response to cadmium ion	3	36	2.17	0.0010	9606.ENSP00000215832,9606.ENSP00000263025,9606.ENSP00000345728	MAPK1,MAPK3,ATP7A
GO Process	GO:0017148	Negative regulation of translation	4	169	1.63	0.0012	9606.ENSP00000258416,9606.ENSP00000336790,9606.ENSP00000416255,9606.ENSP00000425561	EIF4E2,ATF4,EIF4G1,EIF4E
GO Process	GO:0018105	Peptidyl-serine phosphorylation	4	193	1.57	0.0018	9606.ENSP00000215832,9606.ENSP00000229795,9606.ENSP00000250896,9606.ENSP00000498083	MAPK1,MAPK14,MKNK2,MKNK1
GO Process	GO:0018193	Peptidyl-amino acid modification	6	900	1.08	0.0018	9606.ENSP00000215832,9606.ENSP00000229795,9606.ENSP00000250896,9606.ENSP00000263025,9606.ENSP00000345728,9606.ENSP00000498083	MAPK1,MAPK14,MKNK2,MAPK3,ATP7A,MKNK1
GO Process	GO:0031667	Response to nutrient levels	5	461	1.29	0.0018	9606.ENSP00000215832,9606.ENSP00000229795,9606.ENSP00000263025,9606.ENSP00000336790,9606.ENSP00000416255	MAPK1,MAPK14,MAPK3,ATF4,EIF4G1
GO Process	GO:0034198	Cellular response to amino acid starvation	3	49	2.04	0.0018	9606.ENSP00000215832,9606.ENSP00000263025,9606.ENSP00000336790	MAPK1,MAPK3,ATF4

GO Process	GO:0035295	Tube development	6	880	1.09	0.0018	9606.ENSP00000215832,9606.ENSP00000229795,9606.ENSP00000263025,9606.ENSP00000345728,9606.ENSP00000416255,9606.ENSP00000425561	MAPK1,MAPK14,MAPK3,ATP7A,EIF4G1,EIF4E
GO Process	GO:0044271	Cellular nitrogen compound biosynthetic process	7	1494	0.92	0.0018	9606.ENSP00000229795,9606.ENSP00000258416,9606.ENSP00000263025,9606.ENSP00000336790,9606.ENSP00000416255,9606.ENSP00000425561,9606.ENSP00000497422	MAPK14,EIF4E2,MAPK3,ATF4,EIF4G1,EIF4E,EIF4E1B
GO Process	GO:0071248	Cellular response to metal ion	4	198	1.56	0.0018	9606.ENSP00000215832,9606.ENSP00000263025,9606.ENSP00000336790,9606.ENSP00000345728	MAPK1,MAPK3,ATF4,ATP7A
GO Process	GO:0090170	Regulation of Golgi inheritance	2	4	2.95	0.0018	9606.ENSP00000215832,9606.ENSP00000263025	MAPK1,MAPK3
GO Process	GO:0031669	Cellular response to nutrient levels	4	217	1.52	0.0019	9606.ENSP00000215832,9606.ENSP00000263025,9606.ENSP00000336790,9606.ENSP00000416255	MAPK1,MAPK3,ATF4,EIF4G1
GO Process	GO:0044249	Cellular biosynthetic process	8	2362	0.78	0.0019	9606.ENSP00000229795,9606.ENSP00000258416,9606.ENSP00000263025,9606.ENSP00000336790,9606.ENSP00000345728,9606.ENSP00000416255,9606.ENSP00000425561,9606.ENSP00000497422	MAPK14,EIF4E2,MAPK3,ATF4,ATP7A,EIF4G1,EIF4E,EIF4E1B
GO Process	GO:1901700	Response to oxygen-containing compound	7	1547	0.91	0.0019	9606.ENSP00000215832,9606.ENSP00000229795,9606.ENSP00000263025,9606.ENSP00000336790,9606.ENSP00000345728,9606.ENSP00000416255,9606.ENSP00000425561	MAPK1,MAPK14,MAPK3,ATF4,ATP7A,EIF4G1,EIF4E
GO Process	GO:0034599	Cellular response to oxidative stress	4	224	1.5	0.0020	9606.ENSP00000215832,9606.ENSP00000263025,9606.ENSP00000336790,9606.ENSP00000345728	MAPK1,MAPK3,ATF4,ATP7A
GO Process	GO:0034641	Cellular nitrogen compound metabolic process	9	3463	0.67	0.0021	9606.ENSP00000215832,9606.ENSP00000229795,9606.ENSP00000258416,9606.ENSP00000263025,9606.ENSP00000336790,9606.ENSP00000345728,9606.ENSP00000416255,9606.ENSP00000425561,9606.ENSP00000497422	MAPK1,MAPK14,EIF4E2,MAPK3,ATF4,ATP7A,EIF4G1,EIF4E,EIF4E1B
GO Process	GO:00442	Cellular metabolic process	11	6568	0.48	0.0021	9606.ENSP00000215832,9606.ENSP00000229795,9606.ENSP00000250896,9606.ENSP00000258416,9606.ENSP00000263025,9606.ENSP00000336790,9606.ENSP00000345728,9606.ENSP00000416255,9606.ENSP00000425561,960	MAPK1,MAPK14,MKNK2,EIF4E2,MAPK3,ATF4,ATP7A,EIF4G1,EIF4E,

	37						6.ENSP00000497422,9606.ENSP00000498083	EIF4E1B,MKNK1
GO Process	GO:0072584	Caveolin-mediated endocytosis	2	5	2.86	0.0021	9606.ENSP00000215832,9606.ENSP00000263025	MAPK1,MAPK3
GO Process	GO:0120041	Positive regulation of macrophage proliferation	2	5	2.86	0.0021	9606.ENSP00000215832,9606.ENSP00000263025	MAPK1,MAPK3
GO Process	GO:1901701	Cellular response to oxygen-containing compound	6	1057	1.01	0.0026	9606.ENSP00000215832,9606.ENSP00000229795,9606.ENSP00000263025,9606.ENSP00000336790,9606.ENSP00000345728,9606.ENSP00000425561	MAPK1,MAPK14,MAPK3,ATF4,ATP7A,EIF4E
GO Process	GO:0051403	Stress-activated MAPK cascade	3	75	1.86	0.0027	9606.ENSP00000215832,9606.ENSP00000229795,9606.ENSP00000263025	MAPK1,MAPK14,MAPK3
GO Process	GO:0060440	Trachea formation	2	7	2.71	0.0028	9606.ENSP00000215832,9606.ENSP00000263025	MAPK1,MAPK3
GO Process	GO:1903351	Cellular response to dopamine	3	81	1.82	0.0032	9606.ENSP00000215832,9606.ENSP00000263025,9606.ENSP00000336790	MAPK1,MAPK3,ATF4
GO Process	GO:0042473	Outer ear morphogenesis	2	8	2.65	0.0033	9606.ENSP00000215832,9606.ENSP00000263025	MAPK1,MAPK3
GO Process	GO:0044238	Primary metabolic process	11	7156	0.44	0.0037	9606.ENSP00000215832,9606.ENSP00000229795,9606.ENSP00000250896,9606.ENSP00000258416,9606.ENSP00000263025,9606.ENSP00000336790,9606.ENSP00000345728,9606.ENSP00000416255,9606.ENSP00000425561,9606.ENSP00000497422,9606.ENSP00000498083	MAPK1,MAPK14,MKNK2,EIF4E2,MAPK3,ATF4,ATP7A,EIF4G1,EIF4E,EIF4E1B,MKNK1
GO Process	GO:0060020	Bergmann glial cell differentiation	2	11	2.51	0.0051	9606.ENSP00000215832,9606.ENSP00000263025	MAPK1,MAPK3
GO Proc	GO:00	Cardiac neural crest cell development	2	12	2.47	0.0057	9606.ENSP00000215832,9606.ENSP00000263025	MAPK1,MAPK3

ess	61308	involved in heart development						
GO Process	GO:0022008	Neurogenesis	6	1290	0.92	0.0059	9606.ENSP00000215832,9606.ENSP00000263025,9606.ENSP00000336790,9606.ENSP00000345728,9606.ENSP00000416255,9606.ENSP00000425561	MAPK1,MAPK3,ATF4,ATP7A,EIF4G1,EIF4E
GO Process	GO:0006468	Protein phosphorylation	5	736	1.09	0.0060	9606.ENSP00000215832,9606.ENSP00000229795,9606.ENSP00000250896,9606.ENSP00000263025,9606.ENSP00000498083	MAPK1,MAPK14,MKNK2,MAPK3,MKNK1
GO Process	GO:0009719	Response to endogenous stimulus	6	1363	0.9	0.0073	9606.ENSP00000215832,9606.ENSP00000229795,9606.ENSP00000263025,9606.ENSP00000336790,9606.ENSP00000345728,9606.ENSP00000425561	MAPK1,MAPK14,MAPK3,ATF4,ATP7A,EIF4E
GO Process	GO:0010467	Gene expression	7	2101	0.78	0.0073	9606.ENSP00000229795,9606.ENSP00000258416,9606.ENSP00000263025,9606.ENSP00000336790,9606.ENSP00000416255,9606.ENSP00000425561,9606.ENSP00000497422	MAPK14,EIF4E2,MAPK3,ATF4,EIF4G1,EIF4E,EIF4E1B
GO Process	GO:0030278	Regulation of ossification	3	120	1.65	0.0073	9606.ENSP00000215832,9606.ENSP00000229795,9606.ENSP00000263025	MAPK1,MAPK14,MAPK3
GO Process	GO:0034614	Cellular response to reactive oxygen species	3	118	1.66	0.0073	9606.ENSP00000215832,9606.ENSP00000263025,9606.ENSP00000345728	MAPK1,MAPK3,ATP7A
GO Process	GO:0034645	Cellular macromolecule biosynthetic process	5	778	1.06	0.0073	9606.ENSP00000258416,9606.ENSP00000345728,9606.ENSP00000416255,9606.ENSP00000425561,9606.ENSP00000497422	EIF4E2,ATP7A,EIF4G1,EIF4E,EIF4E1B
GO Process	GO:0035278	miRNA-mediated gene silencing by inhibition of translation	2	15	2.38	0.0073	9606.ENSP00000258416,9606.ENSP00000416255	EIF4E2,EIF4G1
GO Process	GO:0051171	Regulation of nitrogen compound metabolic process	10	5734	0.49	0.0073	9606.ENSP00000215832,9606.ENSP00000229795,9606.ENSP00000250896,9606.ENSP00000258416,9606.ENSP00000263025,9606.ENSP00000336790,9606.ENSP00000416255,9606.ENSP00000425561,9606.ENSP00000497422,9606.ENSP00000498083	MAPK1,MAPK14,MKNK2,EIF4E2,MAPK3,ATF4,EIF4G1,EIF4E,EIF4E1B,MKNK1
GO	GO	Regulation of response	6	1373	0.	0.007	9606.ENSP00000215832,9606.ENSP00000229795,9606.ENSP00000263025,9	MAPK1,MAPK14,MAPK

Process	:0080134	to stress			89	4	606.ENSP00000336790,9606.ENSP00000345728,9606.ENSP00000416255	3,ATF4,ATP7A,EIF4G1
GO Process	GO:0043603	Cellular amide metabolic process	5	810	1.04	0.0083	9606.ENSP00000258416,9606.ENSP00000336790,9606.ENSP00000416255,9606.ENSP00000425561,9606.ENSP00000497422	EIF4E2,ATF4,EIF4G1,EIF4E,EIF4E1B
GO Process	GO:0080090	Regulation of primary metabolic process	10	5899	0.48	0.0084	9606.ENSP00000215832,9606.ENSP00000229795,9606.ENSP00000250896,9606.ENSP00000258416,9606.ENSP00000263025,9606.ENSP00000336790,9606.ENSP00000416255,9606.ENSP00000425561,9606.ENSP00000497422,9606.ENSP00000498083	MAPK1,MAPK14,MKNK2,EIF4E2,MAPK3,ATF4,EIF4G1,EIF4E,EIF4E1B,MKNK1
GO Process	GO:1904355	Positive regulation of telomere capping	2	17	2.32	0.0086	9606.ENSP00000215832,9606.ENSP00000263025	MAPK1,MAPK3
GO Process	GO:0030003	Cellular cation homeostasis	4	404	1.25	0.0092	9606.ENSP00000215832,9606.ENSP00000263025,9606.ENSP00000336790,9606.ENSP00000345728	MAPK1,MAPK3,ATF4,ATP7A
GO Process	GO:2000641	Regulation of early endosome to late endosome transport	2	18	2.3	0.0092	9606.ENSP00000215832,9606.ENSP00000263025	MAPK1,MAPK3
GO Process	GO:0048513	Animal organ development	8	3246	0.64	0.0095	9606.ENSP00000215832,9606.ENSP00000229795,9606.ENSP00000250896,9606.ENSP00000263025,9606.ENSP00000336790,9606.ENSP00000345728,9606.ENSP00000416255,9606.ENSP00000425561	MAPK1,MAPK14,MKNK2,MAPK3,ATF4,ATP7A,EIF4G1,EIF4E
GO Process	GO:0010759	Positive regulation of macrophage chemotaxis	2	19	2.28	0.0099	9606.ENSP00000215832,9606.ENSP00000263025	MAPK1,MAPK3
GO Process	GO:0071243	Cellular response to arsenic-containing substance	2	20	2.25	0.0104	9606.ENSP00000250896,9606.ENSP00000336790	MKNK2,ATF4
GO Process	GO:0035556	Intracellular signal transduction	6	1518	0.85	0.0110	9606.ENSP00000215832,9606.ENSP00000229795,9606.ENSP00000250896,9606.ENSP00000263025,9606.ENSP00000336790,9606.ENSP00000498083	MAPK1,MAPK14,MKNK2,MAPK3,ATF4,MKNK1

GO Process	GO:0010042	Response to manganese ion	2	21	2.23	0.0112	9606.ENSP00000336790,9606.ENSP00000345728	ATF4,ATP7A
GO Process	GO:0033554	Cellular response to stress	6	1572	0.83	0.0132	9606.ENSP00000215832,9606.ENSP00000229795,9606.ENSP00000263025,9606.ENSP00000336790,9606.ENSP00000345728,9606.ENSP00000416255	MAPK1,MAPK14,MAPK3,ATF4,ATP7A,EIF4G1
GO Process	GO:0051216	Cartilage development	3	167	1.51	0.0152	9606.ENSP00000229795,9606.ENSP00000263025,9606.ENSP00000345728	MAPK14,MAPK3,ATP7A
GO Process	GO:0010243	Response to organonitrogen compound	5	963	0.97	0.0155	9606.ENSP00000215832,9606.ENSP00000229795,9606.ENSP00000263025,9606.ENSP00000336790,9606.ENSP00000345728	MAPK1,MAPK14,MAPK3,ATF4,ATP7A
GO Process	GO:0071356	Cellular response to tumor necrosis factor	3	175	1.49	0.0165	9606.ENSP00000215832,9606.ENSP00000229795,9606.ENSP00000263025	MAPK1,MAPK14,MAPK3
GO Process	GO:0030878	Thyroid gland development	2	28	2.11	0.0171	9606.ENSP00000215832,9606.ENSP00000263025	MAPK1,MAPK3
GO Process	GO:0070848	Response to growth factor	4	503	1.15	0.0171	9606.ENSP00000215832,9606.ENSP00000229795,9606.ENSP00000263025,9606.ENSP00000345728	MAPK1,MAPK14,MAPK3,ATP7A
GO Process	GO:0071396	Cellular response to lipid	4	502	1.15	0.0171	9606.ENSP00000215832,9606.ENSP00000229795,9606.ENSP00000263025,9606.ENSP00000425561	MAPK1,MAPK14,MAPK3,EIF4E
GO Process	GO:0001662	Behavioral fear response	2	29	2.09	0.0174	9606.ENSP00000416255,9606.ENSP00000425561	EIF4G1,EIF4E
GO Process	GO:00488	Stem cell differentiation	3	183	1.47	0.0174	9606.ENSP00000215832,9606.ENSP00000229795,9606.ENSP00000263025	MAPK1,MAPK14,MAPK3

	63							
GO Process	GO:0071407	Cellular response to organic cyclic compound	4	508	1.15	0.0174	9606.ENSP00000215832,9606.ENSP00000263025,9606.ENSP00000336790,9606.ENSP00000425561	MAPK1,MAPK3,ATF4,EIF4E
GO Process	GO:0098771	Inorganic ion homeostasis	4	514	1.14	0.0176	9606.ENSP00000215832,9606.ENSP00000263025,9606.ENSP00000336790,9606.ENSP00000345728	MAPK1,MAPK3,ATF4,ATP7A
GO Process	GO:0046777	Protein autophosphorylation	3	189	1.45	0.0182	9606.ENSP00000250896,9606.ENSP00000263025,9606.ENSP00000498083	MKNK2,MAPK3,MKNK1
GO Process	GO:0070887	Cellular response to chemical stimulus	7	2609	0.68	0.0184	9606.ENSP00000215832,9606.ENSP00000229795,9606.ENSP00000250896,9606.ENSP00000263025,9606.ENSP00000336790,9606.ENSP00000345728,9606.ENSP00000425561	MAPK1,MAPK14,MKNK2,MAPK3,ATF4,ATP7A,EIF4E
GO Process	GO:0006915	Apoptotic process	5	1041	0.93	0.0185	9606.ENSP00000215832,9606.ENSP00000229795,9606.ENSP00000250896,9606.ENSP00000263025,9606.ENSP00000336790	MAPK1,MAPK14,MKNK2,MAPK3,ATF4
GO Process	GO:0051130	Positive regulation of cellular component organization	5	1049	0.93	0.0188	9606.ENSP00000215832,9606.ENSP00000229795,9606.ENSP00000263025,9606.ENSP00000345728,9606.ENSP00000416255	MAPK1,MAPK14,MAPK3,ATP7A,EIF4G1
GO Process	GO:0010033	Response to organic substance	7	2692	0.67	0.0205	9606.ENSP00000215832,9606.ENSP00000229795,9606.ENSP00000263025,9606.ENSP00000336790,9606.ENSP00000345728,9606.ENSP00000416255,9606.ENSP00000425561	MAPK1,MAPK14,MAPK3,ATF4,ATP7A,EIF4G1,EIF4E
GO Process	GO:0032212	Positive regulation of telomere maintenance via telomerase	2	34	2.02	0.0205	9606.ENSP00000215832,9606.ENSP00000263025	MAPK1,MAPK3
GO Process	GO:0051973	Positive regulation of telomerase activity	2	34	2.02	0.0205	9606.ENSP00000215832,9606.ENSP00000263025	MAPK1,MAPK3
GO Proc	GO:00	Cellular response to endogenous stimulus	5	1103	0.91	0.0212	9606.ENSP00000215832,9606.ENSP00000263025,9606.ENSP00000336790,9606.ENSP00000345728,9606.ENSP00000425561	MAPK1,MAPK3,ATF4,ATP7A,EIF4E

ess	71495							
GO Process	GO:0048731	System development	8	3867	0.57	0.0223	9606.ENSP00000215832,9606.ENSP00000229795,9606.ENSP00000250896,9606.ENSP00000263025,9606.ENSP00000336790,9606.ENSP00000345728,9606.ENSP00000416255,9606.ENSP00000425561	MAPK1,MAPK14,MKNK2,MAPK3,ATF4,ATP7A,EIF4G1,EIF4E
GO Process	GO:0071417	Cellular response to organonitrogen compound	4	574	1.1	0.0223	9606.ENSP00000215832,9606.ENSP00000263025,9606.ENSP00000336790,9606.ENSP00000345728	MAPK1,MAPK3,ATF4,ATP7A
GO Process	GO:0010628	Positive regulation of gene expression	5	1146	0.89	0.0240	9606.ENSP00000215832,9606.ENSP00000229795,9606.ENSP00000263025,9606.ENSP00000336790,9606.ENSP00000416255	MAPK1,MAPK14,MAPK3,ATF4,EIF4G1
GO Process	GO:0007610	Behavior	4	598	1.08	0.0247	9606.ENSP00000215832,9606.ENSP00000345728,9606.ENSP00000416255,9606.ENSP00000425561	MAPK1,ATP7A,EIF4G1,EIF4E
GO Process	GO:0042221	Response to chemical	8	4010	0.55	0.0273	9606.ENSP00000215832,9606.ENSP00000229795,9606.ENSP00000250896,9606.ENSP00000263025,9606.ENSP00000336790,9606.ENSP00000345728,9606.ENSP00000416255,9606.ENSP00000425561	MAPK1,MAPK14,MKNK2,MAPK3,ATF4,ATP7A,EIF4G1,EIF4E
GO Process	GO:0010799	Regulation of peptidyl-threonine phosphorylation	2	44	1.91	0.0290	9606.ENSP00000215832,9606.ENSP00000416255	MAPK1,EIF4G1
GO Process	GO:0060425	Lung morphogenesis	2	45	1.9	0.0298	9606.ENSP00000215832,9606.ENSP00000263025	MAPK1,MAPK3
GO Process	GO:0070849	Response to epidermal growth factor	2	46	1.89	0.0307	9606.ENSP00000215832,9606.ENSP00000263025	MAPK1,MAPK3
GO Process	GO:0030097	Hemopoiesis	4	655	1.04	0.0328	9606.ENSP00000229795,9606.ENSP00000250896,9606.ENSP00000336790,9606.ENSP00000345728	MAPK14,MKNK2,ATF4,ATP7A
GO	GO	Cellular response to	6	2019	0.	0.032	9606.ENSP00000215832,9606.ENSP00000229795,9606.ENSP00000263025,9	MAPK1,MAPK14,MAPK

Process	:0071310	organic substance			73	9	606.ENSP00000336790,9606.ENSP00000345728,9606.ENSP00000425561	3,ATF4,ATP7A,EIF4E
GO Process	GO:0048538	Thymus development	2	49	1.86	0.0340	9606.ENSP00000215832,9606.ENSP00000263025	MAPK1,MAPK3
GO Process	GO:0031328	Positive regulation of cellular biosynthetic process	6	2041	0.72	0.0345	9606.ENSP00000215832,9606.ENSP00000229795,9606.ENSP00000263025,9606.ENSP00000336790,9606.ENSP00000345728,9606.ENSP00000416255	MAPK1,MAPK14,MAPK3,ATF4,ATP7A,EIF4G1
GO Process	GO:0043330	Response to exogenous dsRNA	2	50	1.86	0.0349	9606.ENSP00000215832,9606.ENSP00000263025	MAPK1,MAPK3
GO Process	GO:0060324	Face development	2	52	1.84	0.0370	9606.ENSP00000215832,9606.ENSP00000263025	MAPK1,MAPK3
GO Process	GO:0031281	Positive regulation of cyclase activity	2	53	1.83	0.0377	9606.ENSP00000229795,9606.ENSP00000263025	MAPK14,MAPK3
GO Process	GO:0090596	Sensory organ morphogenesis	3	276	1.29	0.0386	9606.ENSP00000215832,9606.ENSP00000263025,9606.ENSP00000336790	MAPK1,MAPK3,ATF4
GO Process	GO:0070371	ERK1 and ERK2 cascade	2	55	1.81	0.0401	9606.ENSP00000215832,9606.ENSP00000263025	MAPK1,MAPK3
GO Process	GO:0080135	Regulation of cellular response to stress	4	712	1.0	0.0412	9606.ENSP00000215832,9606.ENSP00000263025,9606.ENSP00000336790,9606.ENSP00000416255	MAPK1,MAPK3,ATF4,EIF4G1
GO Process	GO:1901566	Organonitrogen compound biosynthetic process	5	1338	0.83	0.0419	9606.ENSP00000258416,9606.ENSP00000345728,9606.ENSP00000416255,9606.ENSP00000425561,9606.ENSP00000497422	EIF4E2,ATP7A,EIF4G1,EIF4E,EIF4E1B

GO Function	GO:0003743	Translation initiation factor activity	4	57	2.1	0.00013	9606.ENSP00000258416,9606.ENSP00000416255,9606.ENSP00000425561,9606.ENSP00000497422	EIF4E2,EIF4G1,EIF4E,EIF4E1B
GO Function	GO:0000340	RNA 7-methylguanosine cap binding	3	12	2.65	0.00014	9606.ENSP00000258416,9606.ENSP00000425561,9606.ENSP00000497422	EIF4E2,EIF4E,EIF4E1B
GO Function	GO:0004707	MAP kinase activity	3	15	2.55	0.00017	9606.ENSP00000215832,9606.ENSP00000229795,9606.ENSP00000263025	MAPK1,MAPK14,MAPK3
GO Function	GO:0004708	MAP kinase kinase activity	3	17	2.5	0.00018	9606.ENSP00000215832,9606.ENSP00000229795,9606.ENSP00000263025	MAPK1,MAPK14,MAPK3
GO Function	GO:0106310	Protein serine kinase activity	5	361	1.39	0.00056	9606.ENSP00000215832,9606.ENSP00000229795,9606.ENSP00000250896,9606.ENSP00000263025,9606.ENSP00000498083	MAPK1,MAPK14,MKNK2,MAPK3,MKNK1
GO Function	GO:0004674	Protein serine/threonine kinase activity	5	434	1.31	0.00100	9606.ENSP00000215832,9606.ENSP00000229795,9606.ENSP00000250896,9606.ENSP00000263025,9606.ENSP00000498083	MAPK1,MAPK14,MKNK2,MAPK3,MKNK1
GO Function	GO:0097159	Organic cyclic compound binding	11	6050	0.51	0.00100	9606.ENSP00000215832,9606.ENSP00000229795,9606.ENSP00000250896,9606.ENSP00000258416,9606.ENSP00000263025,9606.ENSP00000336790,9606.ENSP00000345728,9606.ENSP00000416255,9606.ENSP00000425561,9606.ENSP00000497422,9606.ENSP00000498083	MAPK1,MAPK14,MKNK2,EIF4E2,MAPK3,ATF4,ATP7A,EIF4G1,EIF4E,EIF4E1B,MKNK1
GO Function	GO:1901363	Heterocyclic compound binding	11	5977	0.52	0.00100	9606.ENSP00000215832,9606.ENSP00000229795,9606.ENSP00000250896,9606.ENSP00000258416,9606.ENSP00000263025,9606.ENSP00000336790,9606.ENSP00000345728,9606.ENSP00000416255,9606.ENSP00000425561,9606.ENSP00000497422,9606.ENSP00000498083	MAPK1,MAPK14,MKNK2,EIF4E2,MAPK3,ATF4,ATP7A,EIF4G1,EIF4E,EIF4E1B,MKNK1
GO Function	GO:0005524	ATP binding	7	1491	0.92	0.0013	9606.ENSP00000215832,9606.ENSP00000229795,9606.ENSP00000250896,9606.ENSP00000263025,9606.ENSP00000345728,9606.ENSP00000416255,9606.ENSP00000498083	MAPK1,MAPK14,MKNK2,MAPK3,ATP7A,EIF4G1,MKNK1
GO Functio	GO:00198	Enzyme binding	7	2084	0.78	0.0072	9606.ENSP00000215832,9606.ENSP00000229795,9606.ENSP00000258416,9606.ENSP00000263025,9606.ENSP00000336790,9606.ENSP00000345728,9606.ENSP00000425561	MAPK1,MAPK14,EIF4E2,MAPK3,ATF4,ATP7A,EIF4E

n	99							
GO Function	GO:0009931	Calcium-dependent protein serine/threonine kinase activity	2	22	2.21	0.0137	9606.ENSP00000250896,9606.ENSP00000498083	MKNK2,MKNK1
GO Function	GO:0004683	Calmodulin-dependent protein kinase activity	2	27	2.12	0.0173	9606.ENSP00000250896,9606.ENSP00000498083	MKNK2,MKNK1
GO Function	GO:0019902	Phosphatase binding	3	198	1.43	0.0235	9606.ENSP00000215832,9606.ENSP00000229795,9606.ENSP00000263025	MAPK1,MAPK14,MAPK3
GO Function	GO:0031369	Translation initiation factor binding	2	33	2.04	0.0235	9606.ENSP00000416255,9606.ENSP00000425561	EIF4G1,EIF4E
GO Function	GO:0001784	Phosphotyrosine residue binding	2	46	1.89	0.0432	9606.ENSP00000215832,9606.ENSP00000263025	MAPK1,MAPK3
GO Function	GO:0005515	Protein binding	10	7242	0.39	0.0442	9606.ENSP00000215832,9606.ENSP00000229795,9606.ENSP00000250896,9606.ENSP00000258416,9606.ENSP00000263025,9606.ENSP00000336790,9606.ENSP00000345728,9606.ENSP00000416255,9606.ENSP00000425561,9606.ENSP00000498083	MAPK1,MAPK14,MKNK2,EIF4E2,MAPK3,ATF4,ATP7A,EIF4G1,EIF4E,MKNK1
GO Component	GO:0016281	Eukaryotic translation initiation factor 4F complex	4	12	2.78	1.95e-07	9606.ENSP00000258416,9606.ENSP00000416255,9606.ENSP00000425561,9606.ENSP00000497422	EIF4E2,EIF4G1,EIF4E,EIF4E1B
GO Component	GO:0005845	mRNA cap binding complex	3	13	2.62	7.38e-05	9606.ENSP00000258416,9606.ENSP00000425561,9606.ENSP00000497422	EIF4E2,EIF4E,EIF4E1B
GO Component	GO:0031143	Pseudopodium	2	18	2.3	0.0274	9606.ENSP00000215832,9606.ENSP00000263025	MAPK1,MAPK3
STRING	CL:452	Initiation factor	3	43	2.1	0.0089	9606.ENSP00000416255,9606.ENSP00000425561,9606.ENSP00000497422	EIF4G1,EIF4E,EIF4E1B

clusters								
STRING clusters	CL:29008	Mixed, incl. D-Glutamine and D-glutamate metabolism, and HELP motif	2	5	2.86	0.0136	9606.ENSP00000250896,9606.ENSP00000498083	MKNK2,MKNK1
STRING clusters	CL:493	Initiation factor 4G, and RNA strand-exchange activity	2	5	2.86	0.0136	9606.ENSP00000416255,9606.ENSP00000425561	EIF4G1,EIF4E
STRING clusters	CL:17676	Negative feedback regulation of MAPK pathway, and RAS GTPase cycle mutants	2	14	2.41	0.0310	9606.ENSP00000215832,9606.ENSP00000263025	MAPK1,MAPK3
KEGG	hsa04066	HIF-1 signaling pathway	6	102	2.02	3.57e-09	9606.ENSP00000215832,9606.ENSP00000250896,9606.ENSP00000258416,9606.ENSP00000263025,9606.ENSP00000497422,9606.ENSP00000498083	MAPK1,MKNK2,EIF4E2,MAPK3,EIF4E1B,MKNK1
KEGG	hsa04910	Insulin signaling pathway	6	132	1.91	7.96e-09	9606.ENSP00000215832,9606.ENSP00000250896,9606.ENSP00000258416,9606.ENSP00000263025,9606.ENSP00000497422,9606.ENSP00000498083	MAPK1,MKNK2,EIF4E2,MAPK3,EIF4E1B,MKNK1
KEGG	hsa04010	MAPK signaling pathway	6	286	1.57	4.88e-07	9606.ENSP00000215832,9606.ENSP00000229795,9606.ENSP00000250896,9606.ENSP00000263025,9606.ENSP00000336790,9606.ENSP00000498083	MAPK1,MAPK14,MKNK2,MAPK3,ATF4,MKNK1
KEGG	hsa01521	EGFR tyrosine kinase inhibitor resistance	4	77	1.97	7.18e-06	9606.ENSP00000215832,9606.ENSP00000258416,9606.ENSP00000263025,9606.ENSP00000497422	MAPK1,EIF4E2,MAPK3,EIF4E1B
KEGG	hsa04912	GnRH signaling pathway	4	87	1.92	9.20e-06	9606.ENSP00000215832,9606.ENSP00000229795,9606.ENSP00000263025,9606.ENSP00000336790	MAPK1,MAPK14,MAPK3,ATF4
KEGG	hsa04668	TNF signaling pathway	4	111	1.81	1.97e-05	9606.ENSP00000215832,9606.ENSP00000229795,9606.ENSP00000263025,9606.ENSP00000336790	MAPK1,MAPK14,MAPK3,ATF4
KEGG	hsa04722	Neurotrophin signaling pathway	4	112	1.81	1.97e-05	9606.ENSP00000215832,9606.ENSP00000229795,9606.ENSP00000263025,9606.ENSP00000336790	MAPK1,MAPK14,MAPK3,ATF4
KEGG	hsa04935	Growth hormone synthesis, secretion and action	4	117	1.79	1.97e-05	9606.ENSP00000215832,9606.ENSP00000229795,9606.ENSP00000263025,9606.ENSP00000336790	MAPK1,MAPK14,MAPK3,ATF4
KE	hsa	Relaxin signaling	4	126	1.	2.15e	9606.ENSP00000215832,9606.ENSP00000229795,9606.ENSP00000263025,9	MAPK1,MAPK14,MAPK

GG	04926	pathway			75	-05	606.ENSP00000336790	3,ATF4
KEGG	hsa04151	PI3K-Akt signaling pathway	5	349	1.41	2.58e-05	9606.ENSP00000215832,9606.ENSP00000258416,9606.ENSP00000263025,9606.ENSP00000336790,9606.ENSP00000497422	MAPK1,EIF4E2,MAPK3,ATF4,EIF4E1B
KEGG	hsa04261	Adrenergic signaling in cardiomyocytes	4	146	1.69	3.12e-05	9606.ENSP00000215832,9606.ENSP00000229795,9606.ENSP00000263025,9606.ENSP00000336790	MAPK1,MAPK14,MAPK3,ATF4
KEGG	hsa04150	mTOR signaling pathway	4	150	1.68	3.17e-05	9606.ENSP00000215832,9606.ENSP00000258416,9606.ENSP00000263025,9606.ENSP00000497422	MAPK1,EIF4E2,MAPK3,EIF4E1B
KEGG	hsa05161	Hepatitis B	4	158	1.66	3.59e-05	9606.ENSP00000215832,9606.ENSP00000229795,9606.ENSP00000263025,9606.ENSP00000336790	MAPK1,MAPK14,MAPK3,ATF4
KEGG	hsa04370	VEGF signaling pathway	3	56	1.98	9.92e-05	9606.ENSP00000215832,9606.ENSP00000229795,9606.ENSP00000263025	MAPK1,MAPK14,MAPK3
KEGG	hsa05163	Human cytomegalovirus infection	4	217	1.52	0.00011	9606.ENSP00000215832,9606.ENSP00000229795,9606.ENSP00000263025,9606.ENSP00000336790	MAPK1,MAPK14,MAPK3,ATF4
KEGG	hsa04720	Long-term potentiation	3	63	1.93	0.00012	9606.ENSP00000215832,9606.ENSP00000263025,9606.ENSP00000336790	MAPK1,MAPK3,ATF4
KEGG	hsa04664	Fc epsilon RI signaling pathway	3	65	1.92	0.00013	9606.ENSP00000215832,9606.ENSP00000229795,9606.ENSP00000263025	MAPK1,MAPK14,MAPK3
KEGG	hsa01524	Platinum drug resistance	3	70	1.89	0.00014	9606.ENSP00000215832,9606.ENSP00000263025,9606.ENSP00000345728	MAPK1,MAPK3,ATP7A
KEGG	hsa04917	Prolactin signaling pathway	3	68	1.9	0.00014	9606.ENSP00000215832,9606.ENSP00000229795,9606.ENSP00000263025	MAPK1,MAPK14,MAPK3
KEGG	hsa05133	Pertussis	3	73	1.87	0.00014	9606.ENSP00000215832,9606.ENSP00000229795,9606.ENSP00000263025	MAPK1,MAPK14,MAPK3
KEGG	hsa05140	Leishmaniasis	3	69	1.89	0.00014	9606.ENSP00000215832,9606.ENSP00000229795,9606.ENSP00000263025	MAPK1,MAPK14,MAPK3
KE	hsa	Prion disease	4	263	1.	0.000	9606.ENSP00000215832,9606.ENSP00000229795,9606.ENSP00000263025,9	MAPK1,MAPK14,MAPK

GG	05020				44	15	606.ENSP00000336790	3,ATF4
KEGG	hsa04658	Th1 and Th2 cell differentiation	3	85	1.8	0.00020	9606.ENSP00000215832,9606.ENSP00000229795,9606.ENSP00000263025	MAPK1,MAPK14,MAPK3
KEGG	hsa04211	Longevity regulating pathway	3	87	1.79	0.00021	9606.ENSP00000258416,9606.ENSP00000336790,9606.ENSP00000497422	EIF4E2,ATF4,EIF4E1B
KEGG	hsa05235	PD-L1 expression and PD-1 checkpoint pathway in cancer	3	87	1.79	0.00021	9606.ENSP00000215832,9606.ENSP00000229795,9606.ENSP00000263025	MAPK1,MAPK14,MAPK3
KEGG	hsa04657	IL-17 signaling pathway	3	91	1.77	0.00022	9606.ENSP00000215832,9606.ENSP00000229795,9606.ENSP00000263025	MAPK1,MAPK14,MAPK3
KEGG	hsa01522	Endocrine resistance	3	94	1.76	0.00023	9606.ENSP00000215832,9606.ENSP00000229795,9606.ENSP00000263025	MAPK1,MAPK14,MAPK3
KEGG	hsa04620	Toll-like receptor signaling pathway	3	100	1.73	0.00023	9606.ENSP00000215832,9606.ENSP00000229795,9606.ENSP00000263025	MAPK1,MAPK14,MAPK3
KEGG	hsa04625	C-type lectin receptor signaling pathway	3	101	1.73	0.00023	9606.ENSP00000215832,9606.ENSP00000229795,9606.ENSP00000263025	MAPK1,MAPK14,MAPK3
KEGG	hsa04659	Th17 cell differentiation	3	99	1.73	0.00023	9606.ENSP00000215832,9606.ENSP00000229795,9606.ENSP00000263025	MAPK1,MAPK14,MAPK3
KEGG	hsa04660	T cell receptor signaling pathway	3	100	1.73	0.00023	9606.ENSP00000215832,9606.ENSP00000229795,9606.ENSP00000263025	MAPK1,MAPK14,MAPK3
KEGG	hsa04914	Progesterone-mediated oocyte maturation	3	95	1.75	0.00023	9606.ENSP00000215832,9606.ENSP00000229795,9606.ENSP00000263025	MAPK1,MAPK14,MAPK3
KEGG	hsa04928	Parathyroid hormone synthesis, secretion and action	3	104	1.71	0.00023	9606.ENSP00000215832,9606.ENSP00000263025,9606.ENSP00000336790	MAPK1,MAPK3,ATF4
KEGG	hsa04933	AGE-RAGE signaling pathway in diabetic complications	3	96	1.75	0.00023	9606.ENSP00000215832,9606.ENSP00000229795,9606.ENSP00000263025	MAPK1,MAPK14,MAPK3
KE	hsa	Chagas disease	3	97	1.	0.000	9606.ENSP00000215832,9606.ENSP00000229795,9606.ENSP00000263025	MAPK1,MAPK14,MAPK

GG	05142				74	23		3
KEGG	hsa05145	Toxoplasmosis	3	103	1.72	0.00023	9606.ENSP00000215832,9606.ENSP00000229795,9606.ENSP00000263025	MAPK1,MAPK14,MAPK3
KEGG	hsa05215	Prostate cancer	3	97	1.74	0.00023	9606.ENSP00000215832,9606.ENSP00000263025,9606.ENSP00000336790	MAPK1,MAPK3,ATF4
KEGG	hsa04725	Cholinergic synapse	3	109	1.69	0.00025	9606.ENSP00000215832,9606.ENSP00000263025,9606.ENSP00000336790	MAPK1,MAPK3,ATF4
KEGG	hsa04071	Sphingolipid signaling pathway	3	116	1.67	0.00029	9606.ENSP00000215832,9606.ENSP00000229795,9606.ENSP00000263025	MAPK1,MAPK14,MAPK3
KEGG	hsa04114	Oocyte meiosis	3	121	1.65	0.00032	9606.ENSP00000215832,9606.ENSP00000229795,9606.ENSP00000263025	MAPK1,MAPK14,MAPK3
KEGG	hsa04380	Osteoclast differentiation	3	120	1.65	0.00032	9606.ENSP00000215832,9606.ENSP00000229795,9606.ENSP00000263025	MAPK1,MAPK14,MAPK3
KEGG	hsa04611	Platelet activation	3	122	1.64	0.00032	9606.ENSP00000215832,9606.ENSP00000229795,9606.ENSP00000263025	MAPK1,MAPK14,MAPK3
KEGG	hsa05135	Yersinia infection	3	124	1.64	0.00032	9606.ENSP00000215832,9606.ENSP00000229795,9606.ENSP00000263025	MAPK1,MAPK14,MAPK3
KEGG	hsa04068	FoxO signaling pathway	3	126	1.63	0.00033	9606.ENSP00000215832,9606.ENSP00000229795,9606.ENSP00000263025	MAPK1,MAPK14,MAPK3
KEGG	hsa04210	Apoptosis	3	131	1.61	0.00036	9606.ENSP00000215832,9606.ENSP00000263025,9606.ENSP00000336790	MAPK1,MAPK3,ATF4
KEGG	hsa04915	Estrogen signaling pathway	3	133	1.61	0.00037	9606.ENSP00000215832,9606.ENSP00000263025,9606.ENSP00000336790	MAPK1,MAPK3,ATF4
KEGG	hsa04550	Signaling pathways regulating pluripotency of stem cells	3	141	1.58	0.00043	9606.ENSP00000215832,9606.ENSP00000229795,9606.ENSP00000263025	MAPK1,MAPK14,MAPK3

KEGG	hsa04723	Retrograde endocannabinoid signaling	3	142	1.58	0.00043	9606.ENSP00000215832,9606.ENSP00000229795,9606.ENSP00000263025	MAPK1,MAPK14,MAPK3
KEGG	hsa05034	Alcoholism	3	146	1.57	0.00046	9606.ENSP00000215832,9606.ENSP00000263025,9606.ENSP00000336790	MAPK1,MAPK3,ATF4
KEGG	hsa04218	Cellular senescence	3	150	1.55	0.00049	9606.ENSP00000215832,9606.ENSP00000229795,9606.ENSP00000263025	MAPK1,MAPK14,MAPK3
KEGG	hsa04934	Cushing syndrome	3	153	1.55	0.00050	9606.ENSP00000215832,9606.ENSP00000263025,9606.ENSP00000336790	MAPK1,MAPK3,ATF4
KEGG	hsa03013	RNA transport	3	161	1.52	0.00057	9606.ENSP00000258416,9606.ENSP00000416255,9606.ENSP00000497422	EIF4E2,EIF4G1,EIF4E1B
KEGG	hsa04022	cGMP-PKG signaling pathway	3	163	1.52	0.00058	9606.ENSP00000215832,9606.ENSP00000263025,9606.ENSP00000336790	MAPK1,MAPK3,ATF4
KEGG	hsa05152	Tuberculosis	3	165	1.51	0.00059	9606.ENSP00000215832,9606.ENSP00000229795,9606.ENSP00000263025	MAPK1,MAPK14,MAPK3
KEGG	hsa04621	NOD-like receptor signaling pathway	3	173	1.49	0.00067	9606.ENSP00000215832,9606.ENSP00000229795,9606.ENSP00000263025	MAPK1,MAPK14,MAPK3
KEGG	hsa05203	Viral carcinogenesis	3	183	1.47	0.00077	9606.ENSP00000215832,9606.ENSP00000263025,9606.ENSP00000336790	MAPK1,MAPK3,ATF4
KEGG	hsa05130	Pathogenic Escherichia coli infection	3	187	1.46	0.00081	9606.ENSP00000215832,9606.ENSP00000229795,9606.ENSP00000263025	MAPK1,MAPK14,MAPK3
KEGG	hsa05167	Kaposi sarcoma-associated herpesvirus infection	3	187	1.46	0.00081	9606.ENSP00000215832,9606.ENSP00000229795,9606.ENSP00000263025	MAPK1,MAPK14,MAPK3
KEGG	hsa05205	Proteoglycans in cancer	3	194	1.44	0.00087	9606.ENSP00000215832,9606.ENSP00000229795,9606.ENSP00000263025	MAPK1,MAPK14,MAPK3
KEGG	hsa04015	Rap1 signaling pathway	3	201	1.43	0.00095	9606.ENSP00000215832,9606.ENSP00000229795,9606.ENSP00000263025	MAPK1,MAPK14,MAPK3

KEGG	hsa05170	Human immunodeficiency virus 1 infection	3	203	1.42	0.00096	9606.ENSP00000215832,9606.ENSP00000229795,9606.ENSP00000263025	MAPK1,MAPK14,MAPK3
KEGG	hsa05132	Salmonella infection	3	209	1.41	0.0010	9606.ENSP00000215832,9606.ENSP00000229795,9606.ENSP00000263025	MAPK1,MAPK14,MAPK3
KEGG	hsa05166	Human T-cell leukemia virus 1 infection	3	210	1.41	0.0010	9606.ENSP00000215832,9606.ENSP00000263025,9606.ENSP00000336790	MAPK1,MAPK3,ATF4
KEGG	hsa04960	Aldosterone-regulated sodium reabsorption	2	37	1.99	0.0011	9606.ENSP00000215832,9606.ENSP00000263025	MAPK1,MAPK3
KEGG	hsa05131	Shigellosis	3	218	1.39	0.0011	9606.ENSP00000215832,9606.ENSP00000229795,9606.ENSP00000263025	MAPK1,MAPK14,MAPK3
KEGG	hsa05216	Thyroid cancer	2	37	1.99	0.0011	9606.ENSP00000215832,9606.ENSP00000263025	MAPK1,MAPK3
KEGG	hsa05219	Bladder cancer	2	40	1.95	0.0012	9606.ENSP00000215832,9606.ENSP00000263025	MAPK1,MAPK3
KEGG	hsa04930	Type II diabetes mellitus	2	45	1.9	0.0015	9606.ENSP00000215832,9606.ENSP00000263025	MAPK1,MAPK3
KEGG	hsa04730	Long-term depression	2	59	1.78	0.0024	9606.ENSP00000215832,9606.ENSP00000263025	MAPK1,MAPK3
KEGG	hsa05213	Endometrial cancer	2	58	1.79	0.0024	9606.ENSP00000215832,9606.ENSP00000263025	MAPK1,MAPK3
KEGG	hsa04929	GnRH secretion	2	63	1.75	0.0027	9606.ENSP00000215832,9606.ENSP00000263025	MAPK1,MAPK3
KEGG	hsa05211	Renal cell carcinoma	2	65	1.74	0.0029	9606.ENSP00000215832,9606.ENSP00000263025	MAPK1,MAPK3
KEGG	hsa04520	Adherens junction	2	69	1.72	0.0030	9606.ENSP00000215832,9606.ENSP00000263025	MAPK1,MAPK3

KEGG	hsa05221	Acute myeloid leukemia	2	67	1.73	0.0030	9606.ENSP00000215832,9606.ENSP00000263025	MAPK1,MAPK3
KEGG	hsa05223	Non-small cell lung cancer	2	68	1.72	0.0030	9606.ENSP00000215832,9606.ENSP00000263025	MAPK1,MAPK3
KEGG	hsa05230	Central carbon metabolism in cancer	2	68	1.72	0.0030	9606.ENSP00000215832,9606.ENSP00000263025	MAPK1,MAPK3
KEGG	hsa05212	Pancreatic cancer	2	71	1.7	0.0032	9606.ENSP00000215832,9606.ENSP00000263025	MAPK1,MAPK3
KEGG	hsa05214	Glioma	2	71	1.7	0.0032	9606.ENSP00000215832,9606.ENSP00000263025	MAPK1,MAPK3
KEGG	hsa05218	Melanoma	2	72	1.7	0.0032	9606.ENSP00000215832,9606.ENSP00000263025	MAPK1,MAPK3
KEGG	hsa05220	Chronic myeloid leukemia	2	75	1.68	0.0034	9606.ENSP00000215832,9606.ENSP00000263025	MAPK1,MAPK3
KEGG	hsa04662	B cell receptor signaling pathway	2	78	1.66	0.0036	9606.ENSP00000215832,9606.ENSP00000263025	MAPK1,MAPK3
KEGG	hsa05010	Alzheimer disease	3	354	1.18	0.0036	9606.ENSP00000215832,9606.ENSP00000263025,9606.ENSP00000336790	MAPK1,MAPK3,ATF4
KEGG	hsa04012	ErbB signaling pathway	2	81	1.65	0.0038	9606.ENSP00000215832,9606.ENSP00000263025	MAPK1,MAPK3
KEGG	hsa05210	Colorectal cancer	2	82	1.64	0.0039	9606.ENSP00000215832,9606.ENSP00000263025	MAPK1,MAPK3
KEGG	hsa04540	Gap junction	2	87	1.61	0.0043	9606.ENSP00000215832,9606.ENSP00000263025	MAPK1,MAPK3
KEGG	hsa04666	Fc gamma R-mediated phagocytosis	2	90	1.6	0.0045	9606.ENSP00000215832,9606.ENSP00000263025	MAPK1,MAPK3

KEGG	hsa04350	TGF-beta signaling pathway	2	91	1.6	0.0046	9606.ENSP00000215832,9606.ENSP00000263025	MAPK1,MAPK3
KEGG	hsa04713	Circadian entrainment	2	91	1.6	0.0046	9606.ENSP00000215832,9606.ENSP00000263025	MAPK1,MAPK3
KEGG	hsa04916	Melanogenesis	2	95	1.58	0.0048	9606.ENSP00000215832,9606.ENSP00000263025	MAPK1,MAPK3
KEGG	hsa05231	Choline metabolism in cancer	2	95	1.58	0.0048	9606.ENSP00000215832,9606.ENSP00000263025	MAPK1,MAPK3
KEGG	hsa04726	Serotonergic synapse	2	108	1.52	0.0061	9606.ENSP00000215832,9606.ENSP00000263025	MAPK1,MAPK3
KEGG	hsa04724	Glutamatergic synapse	2	112	1.5	0.0064	9606.ENSP00000215832,9606.ENSP00000263025	MAPK1,MAPK3
KEGG	hsa04650	Natural killer cell mediated cytotoxicity	2	120	1.47	0.0073	9606.ENSP00000215832,9606.ENSP00000263025	MAPK1,MAPK3
KEGG	hsa04919	Thyroid hormone signaling pathway	2	120	1.47	0.0073	9606.ENSP00000215832,9606.ENSP00000263025	MAPK1,MAPK3
KEGG	hsa04728	Dopaminergic synapse	2	126	1.45	0.0078	9606.ENSP00000229795,9606.ENSP00000336790	MAPK14,ATF4
KEGG	hsa04140	Autophagy - animal	2	131	1.44	0.0084	9606.ENSP00000215832,9606.ENSP00000263025	MAPK1,MAPK3
KEGG	hsa04270	Vascular smooth muscle contraction	2	132	1.43	0.0084	9606.ENSP00000215832,9606.ENSP00000263025	MAPK1,MAPK3
KEGG	hsa04371	Apelin signaling pathway	2	133	1.43	0.0084	9606.ENSP00000215832,9606.ENSP00000263025	MAPK1,MAPK3
KEGG	hsa04072	Phospholipase D signaling pathway	2	147	1.39	0.0100	9606.ENSP00000215832,9606.ENSP00000263025	MAPK1,MAPK3

KEGG	hsa04921	Oxytocin signaling pathway	2	147	1.39	0.0100	9606.ENSP00000215832,9606.ENSP00000263025	MAPK1,MAPK3
KEGG	hsa05224	Breast cancer	2	146	1.39	0.0100	9606.ENSP00000215832,9606.ENSP00000263025	MAPK1,MAPK3
KEGG	hsa05226	Gastric cancer	2	146	1.39	0.0100	9606.ENSP00000215832,9606.ENSP00000263025	MAPK1,MAPK3
KEGG	hsa05160	Hepatitis C	2	157	1.36	0.0111	9606.ENSP00000215832,9606.ENSP00000263025	MAPK1,MAPK3
KEGG	hsa05206	MicroRNAs in cancer	2	159	1.35	0.0112	9606.ENSP00000215832,9606.ENSP00000263025	MAPK1,MAPK3
KEGG	hsa05225	Hepatocellular carcinoma	2	161	1.35	0.0114	9606.ENSP00000215832,9606.ENSP00000263025	MAPK1,MAPK3
KEGG	hsa05164	Influenza A	2	163	1.34	0.0116	9606.ENSP00000215832,9606.ENSP00000263025	MAPK1,MAPK3
KEGG	hsa04360	Axon guidance	2	176	1.31	0.0133	9606.ENSP00000215832,9606.ENSP00000263025	MAPK1,MAPK3
KEGG	hsa04062	Chemokine signaling pathway	2	186	1.28	0.0146	9606.ENSP00000215832,9606.ENSP00000263025	MAPK1,MAPK3
KEGG	hsa04510	Focal adhesion	2	195	1.26	0.0159	9606.ENSP00000215832,9606.ENSP00000263025	MAPK1,MAPK3
KEGG	hsa04024	cAMP signaling pathway	2	207	1.24	0.0177	9606.ENSP00000215832,9606.ENSP00000263025	MAPK1,MAPK3
KEGG	hsa04810	Regulation of actin cytoskeleton	2	209	1.23	0.0178	9606.ENSP00000215832,9606.ENSP00000263025	MAPK1,MAPK3
KEGG	hsa04014	Ras signaling pathway	2	225	1.2	0.0203	9606.ENSP00000215832,9606.ENSP00000263025	MAPK1,MAPK3

KEGG	hsa05165	Human papillomavirus infection	2	324	1.04	0.0404	9606.ENSP00000215832,9606.ENSP00000263025	MAPK1,MAPK3
KEGG	hsa05014	Amyotrophic lateral sclerosis	2	350	1.01	0.0463	9606.ENSP00000229795,9606.ENSP00000336790	MAPK14,ATF4
Reactome	HSA-450341	Activation of the AP-1 family of transcription factors	3	9	2.78	6.48e-05	9606.ENSP00000215832,9606.ENSP00000229795,9606.ENSP00000263025	MAPK1,MAPK14,MAPK3
Reactome	HSA-1169408	ISG15 antiviral mechanism	4	74	1.99	8.37e-05	9606.ENSP00000258416,9606.ENSP00000263025,9606.ENSP00000416255,9606.ENSP00000425561	EIF4E2,MAPK3,EIF4G1,EIF4E
Reactome	HSA-1295596	Spry regulation of FGF signaling	3	16	2.53	8.37e-05	9606.ENSP00000215832,9606.ENSP00000263025,9606.ENSP00000498083	MAPK1,MAPK3,MKNK1
Reactome	HSA-198753	ERK/MAPK targets	3	22	2.39	0.00013	9606.ENSP00000215832,9606.ENSP00000229795,9606.ENSP00000263025	MAPK1,MAPK14,MAPK3
Reactome	HSA-5668599	RHO GTPases Activate NADPH Oxidases	3	24	2.35	0.00014	9606.ENSP00000215832,9606.ENSP00000229795,9606.ENSP00000263025	MAPK1,MAPK14,MAPK3
Reactome	HSA-1280215	Cytokine Signaling in Immune system	6	706	1.18	0.00021	9606.ENSP00000215832,9606.ENSP00000229795,9606.ENSP00000258416,9606.ENSP00000263025,9606.ENSP00000416255,9606.ENSP00000425561	MAPK1,MAPK14,EIF4E2,MAPK3,EIF4G1,EIF4E
Reactome	HSA-187687	Signalling to ERKs	3	34	2.2	0.00021	9606.ENSP00000215832,9606.ENSP00000229795,9606.ENSP00000263025	MAPK1,MAPK14,MAPK3
Rea	HS	Signaling by MAP2K	2	4	2.	0.000	9606.ENSP00000215832,9606.ENSP00000263025	MAPK1,MAPK3

ctome	A-9652169	mutants			95	54		
Reactome	HSA-5674499	Negative feedback regulation of MAPK pathway	2	6	2.78	0.00082	9606.ENSP00000215832,9606.ENSP00000263025	MAPK1,MAPK3
Reactome	HSA-444257	RSK activation	2	7	2.71	0.00097	9606.ENSP00000215832,9606.ENSP00000263025	MAPK1,MAPK3
Reactome	HSA-9635465	Suppression of apoptosis	2	7	2.71	0.00097	9606.ENSP00000215832,9606.ENSP00000263025	MAPK1,MAPK3
Reactome	HSA-162658	Golgi Cisternae Pericentriolar Stack Reorganization	2	14	2.41	0.0015	9606.ENSP00000215832,9606.ENSP00000263025	MAPK1,MAPK3
Reactome	HSA-168256	Immune System	7	1979	0.8	0.0015	9606.ENSP00000215832,9606.ENSP00000229795,9606.ENSP00000258416,9606.ENSP00000263025,9606.ENSP00000345728,9606.ENSP00000416255,9606.ENSP00000425561	MAPK1,MAPK14,EIF4E2,MAPK3,ATP7A,EIF4G1,EIF4E
Reactome	HSA-170968	Frs2-mediated activation	2	12	2.47	0.0015	9606.ENSP00000215832,9606.ENSP00000263025	MAPK1,MAPK3
Reactome	HSA-202670	ERKs are inactivated	2	13	2.44	0.0015	9606.ENSP00000215832,9606.ENSP00000263025	MAPK1,MAPK3
Reactome	HSA-2262752	Cellular responses to stress	5	747	1.08	0.0015	9606.ENSP00000215832,9606.ENSP00000229795,9606.ENSP00000263025,9606.ENSP00000336790,9606.ENSP00000345728	MAPK1,MAPK14,MAPK3,ATF4,ATP7A

Reactome	HSA-2559580	Oxidative Stress Induced Senescence	3	92	1.77	0.0015	9606.ENSP00000215832,9606.ENSP00000229795,9606.ENSP00000263025	MAPK1,MAPK14,MAPK3
Reactome	HSA-74749	Signal attenuation	2	10	2.55	0.0015	9606.ENSP00000215832,9606.ENSP00000263025	MAPK1,MAPK3
Reactome	HSA-879415	Advanced glycosylation endproduct receptor signaling	2	13	2.44	0.0015	9606.ENSP00000215832,9606.ENSP00000263025	MAPK1,MAPK3
Reactome	HSA-9627069	Regulation of the apoptosome activity	2	11	2.51	0.0015	9606.ENSP00000215832,9606.ENSP00000263025	MAPK1,MAPK3
Reactome	HSA-1502540	Signaling by Activin	2	15	2.38	0.0016	9606.ENSP00000215832,9606.ENSP00000263025	MAPK1,MAPK3
Reactome	HSA-881907	Gastrin-CREB signalling pathway via PKC and MAPK	2	18	2.3	0.0021	9606.ENSP00000215832,9606.ENSP00000263025	MAPK1,MAPK3
Reactome	HSA-9725371	Nuclear events stimulated by ALK signaling in cancer	2	19	2.28	0.0022	9606.ENSP00000215832,9606.ENSP00000263025	MAPK1,MAPK3
Reactome	HSA-445144	Signal transduction by L1	2	21	2.23	0.0027	9606.ENSP00000215832,9606.ENSP00000263025	MAPK1,MAPK3
Reactome	HSA-118	Signaling by NODAL	2	22	2.21	0.0029	9606.ENSP00000215832,9606.ENSP00000263025	MAPK1,MAPK3

	1150							
Reactome	HSA-9768919	NPAS4 regulates expression of target genes	2	22	2.21	0.0029	9606.ENSP00000215832,9606.ENSP00000263025	MAPK1,MAPK3
Reactome	HSA-112409	RAF-independent MAPK1/3 activation	2	23	2.19	0.0030	9606.ENSP00000215832,9606.ENSP00000263025	MAPK1,MAPK3
Reactome	HSA-982772	Growth hormone receptor signaling	2	23	2.19	0.0030	9606.ENSP00000215832,9606.ENSP00000263025	MAPK1,MAPK3
Reactome	HSA-166208	mTORC1-mediated signalling	2	24	2.17	0.0031	9606.ENSP00000416255,9606.ENSP00000425561	EIF4G1,EIF4E
Reactome	HSA-429947	Deadenylation of mRNA	2	25	2.16	0.0031	9606.ENSP00000416255,9606.ENSP00000425561	EIF4G1,EIF4E
Reactome	HSA-8940973	RUNX2 regulates osteoblast differentiation	2	24	2.17	0.0031	9606.ENSP00000215832,9606.ENSP00000263025	MAPK1,MAPK3
Reactome	HSA-9634638	Estrogen-dependent nuclear events downstream of ESR-membrane signaling	2	24	2.17	0.0031	9606.ENSP00000215832,9606.ENSP00000263025	MAPK1,MAPK3
Reactome	HSA-9658195	Leishmania infection	3	165	1.51	0.0031	9606.ENSP00000215832,9606.ENSP00000229795,9606.ENSP00000263025	MAPK1,MAPK14,MAPK3
Rea	HS	Downregulation of	2	29	2.	0.003	9606.ENSP00000215832,9606.ENSP00000263025	MAPK1,MAPK3

ctome	A-2173795	SMAD2/3:SMAD4 transcriptional activity			09	9		
Reactome	HSA-9006934	Signaling by Receptor Tyrosine Kinases	4	521	1.14	0.0042	9606.ENSP00000215832,9606.ENSP00000229795,9606.ENSP00000263025,9606.ENSP00000498083	MAPK1,MAPK14,MAPK3,MKNK1
Reactome	HSA-456926	Thrombin signalling through proteinase activated receptors (PARs)	2	32	2.05	0.0045	9606.ENSP00000215832,9606.ENSP00000263025	MAPK1,MAPK3
Reactome	HSA-2559585	Oncogene Induced Senescence	2	33	2.04	0.0048	9606.ENSP00000215832,9606.ENSP00000263025	MAPK1,MAPK3
Reactome	HSA-2871796	FCERI mediated MAPK activation	2	34	2.02	0.0049	9606.ENSP00000215832,9606.ENSP00000263025	MAPK1,MAPK3
Reactome	HSA-422475	Axon guidance	4	551	1.11	0.0049	9606.ENSP00000215832,9606.ENSP00000229795,9606.ENSP00000263025,9606.ENSP00000416255	MAPK1,MAPK14,MAPK3,EIF4G1
Reactome	HSA-2173796	SMAD2/SMAD3:SMAD4 heterotrimer regulates transcription	2	36	2.0	0.0054	9606.ENSP00000215832,9606.ENSP00000263025	MAPK1,MAPK3
Reactome	HSA-5663213	RHO GTPases Activate WASPs and WAVEs	2	36	2.0	0.0054	9606.ENSP00000215832,9606.ENSP00000263025	MAPK1,MAPK3
Reacto	HSA-	Signaling by high-kinase activity BRAF	2	36	2.0	0.0054	9606.ENSP00000215832,9606.ENSP00000263025	MAPK1,MAPK3

me	6802948	mutants						
Reactome	HSA-5674135	MAP2K and MAPK activation	2	40	1.95	0.0062	9606.ENSP00000215832,9606.ENSP00000263025	MAPK1,MAPK3
Reactome	HSA-9656223	Signaling by RAF1 mutants	2	41	1.94	0.0065	9606.ENSP00000215832,9606.ENSP00000263025	MAPK1,MAPK3
Reactome	HSA-6802946	Signaling by moderate kinase activity BRAF mutants	2	45	1.9	0.0075	9606.ENSP00000215832,9606.ENSP00000263025	MAPK1,MAPK3
Reactome	HSA-6802955	Paradoxical activation of RAF signaling by kinase inactive BRAF	2	45	1.9	0.0075	9606.ENSP00000215832,9606.ENSP00000263025	MAPK1,MAPK3
Reactome	HSA-9649948	Signaling downstream of RAS mutants	2	45	1.9	0.0075	9606.ENSP00000215832,9606.ENSP00000263025	MAPK1,MAPK3
Reactome	HSA-76002	Platelet activation, signaling and aggregation	3	260	1.32	0.0085	9606.ENSP00000215832,9606.ENSP00000229795,9606.ENSP00000263025	MAPK1,MAPK14,MAPK3
Reactome	HSA-72649	Translation initiation complex formation	2	58	1.79	0.0109	9606.ENSP00000416255,9606.ENSP00000425561	EIF4G1,EIF4E
Reactome	HSA-727	Ribosomal scanning and start codon recognition	2	58	1.79	0.0109	9606.ENSP00000416255,9606.ENSP00000425561	EIF4G1,EIF4E

	02							
Reactome	HSA-8943724	Regulation of PTEN gene transcription	2	60	1.78	0.0112	9606.ENSP00000215832,9606.ENSP00000263025	MAPK1,MAPK3
Reactome	HSA-9664422	FCGR3A-mediated phagocytosis	2	62	1.76	0.0118	9606.ENSP00000215832,9606.ENSP00000263025	MAPK1,MAPK3
Reactome	HSA-375165	NCAM signaling for neurite out-growth	2	63	1.75	0.0119	9606.ENSP00000215832,9606.ENSP00000263025	MAPK1,MAPK3
Reactome	HSA-2029482	Regulation of actin dynamics for phagocytic cup formation	2	64	1.75	0.0121	9606.ENSP00000215832,9606.ENSP00000263025	MAPK1,MAPK3
Reactome	HSA-6802952	Signaling by BRAF and RAF1 fusions	2	65	1.74	0.0124	9606.ENSP00000215832,9606.ENSP00000263025	MAPK1,MAPK3
Reactome	HSA-3371453	Regulation of HSF1-mediated heat shock response	2	69	1.72	0.0138	9606.ENSP00000215832,9606.ENSP00000263025	MAPK1,MAPK3
Reactome	HSA-2559582	Senescence-Associated Secretory Phenotype (SASP)	2	81	1.65	0.0183	9606.ENSP00000215832,9606.ENSP00000263025	MAPK1,MAPK3
Reactome	HSA-450531	Regulation of mRNA stability by proteins that bind AU-rich elements	2	86	1.62	0.0201	9606.ENSP00000229795,9606.ENSP00000416255	MAPK14,EIF4G1

Reactome	HSA-162582	Signal Transduction	6	2540	0.63	0.0221	9606.ENSP00000215832,9606.ENSP00000229795,9606.ENSP00000263025,9606.ENSP00000416255,9606.ENSP00000425561,9606.ENSP00000498083	MAPK1,MAPK14,MAPK3,EIF4G1,EIF4E,MKNK1
Reactome	HSA-6811558	PI5P, PP2A and IER3 Regulate PI3K/AKT Signaling	2	106	1.53	0.0287	9606.ENSP00000215832,9606.ENSP00000263025	MAPK1,MAPK3
Reactome	HSA-156827	L13a-mediated translational silencing of Ceruloplasmin expression	2	110	1.51	0.0306	9606.ENSP00000416255,9606.ENSP00000425561	EIF4G1,EIF4E
Reactome	HSA-72706	GTP hydrolysis and joining of the 60S ribosomal subunit	2	111	1.51	0.0309	9606.ENSP00000416255,9606.ENSP00000425561	EIF4G1,EIF4E
Reactome	HSA-168249	Innate Immune System	4	1041	0.84	0.0332	9606.ENSP00000215832,9606.ENSP00000229795,9606.ENSP00000263025,9606.ENSP00000345728	MAPK1,MAPK14,MAPK3,ATP7A

Table 8. Pathway Enrichment Analysis for GO, KEGG, STRING clusters and Reactome Terms within MYLK gene

#category	term ID	term description	observed gene count	background gene count	strength	false discovery rate	matching proteins in your network (IDs)	matching proteins in your network (labels)
GO Function	GO:0005509	Calcium ion binding	9	717	1.35	3.02e-08	9606.ENSP00000279022,9606.ENSP00000291295,9606.ENSP00000304643,9606.ENSP00000315299,9606.ENSP00000369689,9606.ENSP00000419081,9606.ENSP00000463559,9606.ENSP00000464359,9606.ENSP00000499717	MYL9,CALM3,CALML6,CALML3,CALML5,CALML4,MYL12B,MYL12A,CALM1
GO Function	GO:0032036	Myosin heavy chain binding	3	14	2.58	0.00022	9606.ENSP00000279022,9606.ENSP00000463559,9606.ENSP00000464359	MYL9,MYL12B,MYL12A
GO Function	GO:0008092	Cytoskeletal protein binding	7	1002	1.1	0.00041	9606.ENSP00000279022,9606.ENSP00000291295,9606.ENSP00000353452,9606.ENSP00000379616,9606.ENSP00000463559,9606.ENSP00000464359,9606.ENSP00000499717	MYL9,CALM3,MYLK,MYH11,MYL12B,MYL12A,CALM1
GO Function	GO:0010856	Adenylate cyclase activator activity	2	4	2.95	0.0024	9606.ENSP00000291295,9606.ENSP00000499717	CALM3,CALM1
GO Function	GO:0031997	N-terminal myristoylation domain binding	2	3	3.08	0.0024	9606.ENSP00000291295,9606.ENSP00000499717	CALM3,CALM1
GO Function	GO:0035254	Glutamate receptor binding	3	44	2.09	0.0024	9606.ENSP00000291295,9606.ENSP00000464359,9606.ENSP00000499717	CALM3,MYL12A,CALM1
GO Function	GO:0043167	Ion binding	11	6033	0.51	0.0024	9606.ENSP00000279022,9606.ENSP00000291295,9606.ENSP00000304643,9606.ENSP00000315299,9606.ENSP00000353452,9606.ENSP00000369689,9606.ENSP00000379616,9606.ENSP00000419081,9606.ENSP00000463559,9606.ENSP00000464359,9606.E	MYL9,CALM3,CALML6,CALML3,MYLK,CALML5,MYH11,CALML4,MYL12B,MYL12A,CALM1

							NSP00000499717	
GO Function	GO:0046872	Metal ion binding	10	4250	0.62	0.0024	9606.ENSP00000279022,9606.ENSP00000291295,9606.ENSP00000304643,9606.ENSP00000315299,9606.ENSP00000353452,9606.ENSP00000369689,9606.ENSP00000419081,9606.ENSP00000463559,9606.ENSP00000464359,9606.ENSP00000499717	MYL9,CALM3,CALML6,CALML3,MYLK,CALML5,CALML4,MYL12B,MYL12A,CALM1
GO Function	GO:0031800	Type 3 metabotropic glutamate receptor binding	2	5	2.86	0.0029	9606.ENSP00000291295,9606.ENSP00000499717	CALM3,CALM1
GO Function	GO:0030235	Nitric-oxide synthase regulator activity	2	8	2.65	0.0052	9606.ENSP00000291295,9606.ENSP00000499717	CALM3,CALM1
GO Function	GO:0030234	Enzyme regulator activity	6	1239	0.94	0.0083	9606.ENSP00000291295,9606.ENSP00000304643,9606.ENSP00000315299,9606.ENSP00000369689,9606.ENSP00000419081,9606.ENSP00000499717	CALM3,CALML6,CALML3,CALML5,CALML4,CALM1
GO Function	GO:0008179	Adenylate cyclase binding	2	12	2.47	0.0090	9606.ENSP00000291295,9606.ENSP00000499717	CALM3,CALM1
GO Function	GO:0019855	Calcium channel inhibitor activity	2	12	2.47	0.0090	9606.ENSP00000291295,9606.ENSP00000499717	CALM3,CALM1
GO Function	GO:0031432	Titin binding	2	13	2.44	0.0091	9606.ENSP00000291295,9606.ENSP00000499717	CALM3,CALM1
GO Function	GO:0050998	Nitric-oxide synthase binding	2	15	2.38	0.0105	9606.ENSP00000291295,9606.ENSP00000499717	CALM3,CALM1
GO Function	GO:0072542	Protein phosphatase activator activity	2	16	2.35	0.0112	9606.ENSP00000291295,9606.ENSP00000499717	CALM3,CALM1

GO Function	GO:0043548	Phosphatidylinositol 3-kinase binding	2	32	2.05	0.0353	9606.ENSP00000291295,9606.ENSP00000499717	CALM3,CALM1
GO Function	GO:0097718	Disordered domain specific binding	2	34	2.02	0.0379	9606.ENSP00000291295,9606.ENSP00000499717	CALM3,CALM1
GO Component	GO:0016460	Myosin II complex	4	26	2.44	2.92e-06	9606.ENSP00000279022,9606.ENSP00000379616,9606.ENSP00000463559,9606.ENSP00000464359	MYL9,MYH11,MYL12B,MYL12A
GO Component	GO:0043292	Contractile fiber	6	246	1.64	2.92e-06	9606.ENSP00000279022,9606.ENSP00000291295,9606.ENSP00000379616,9606.ENSP00000463559,9606.ENSP00000464359,9606.ENSP00000499717	MYL9,CALM3,MYH11,MYL12B,MYL12A,CALM1
GO Component	GO:0001725	Stress fiber	4	65	2.04	2.28e-05	9606.ENSP00000279022,9606.ENSP00000353452,9606.ENSP00000463559,9606.ENSP00000464359	MYL9,MYLK,MYL12B,MYL12A
GO Component	GO:0030017	Sarcomere	5	217	1.62	2.28e-05	9606.ENSP00000279022,9606.ENSP00000291295,9606.ENSP00000463559,9606.ENSP00000464359,9606.ENSP00000499717	MYL9,CALM3,MYL12B,MYL12A,CALM1
GO Component	GO:0015629	Actin cytoskeleton	5	482	1.27	0.00075	9606.ENSP00000279022,9606.ENSP00000353452,9606.ENSP00000379616,9606.ENSP00000463559,9606.ENSP00000464359	MYL9,MYLK,MYH11,MYL12B,MYL12A
GO Component	GO:0005859	Muscle myosin complex	2	15	2.38	0.0060	9606.ENSP00000279022,9606.ENSP00000379616	MYL9,MYH11
GO Component	GO:0030018	Z disc	3	131	1.61	0.0066	9606.ENSP00000279022,9606.ENSP00000463559,9606.ENSP00000464359	MYL9,MYL12B,MYL12A
GO Compo	GO:0005856	Cytoskeleton	7	2369	0.72	0.0094	9606.ENSP00000279022,9606.ENSP00000291295,9606.ENSP00000353452,9606.ENSP00000379616,9606.ENSP00000463559,9606.ENSP00000464359,960	MYL9,CALM3,MYLK,MYH11,MYL12B,MYL12A,CALM1

nent							6.ENSP00000499717	
GO Compo nent	GO:0043209	Myelin sheath	2	45	1.9	0.0343	9606.ENSP00000291295,9606.ENSP00000499717	CALM3,CALM1
GO Compo nent	GO:0097225	Sperm midpiece	2	49	1.86	0.0383	9606.ENSP00000291295,9606.ENSP00000499717	CALM3,CALM1
STRING clus ters	CL:22569	Muscle protein, and Sarcoplasmic reticulum membrane	7	104	2.08	2.20e-10	9606.ENSP00000279022,9606.ENSP00000315299,9606.ENSP00000353452,9606.ENSP00000379616,9606.ENSP00000419081,9606.ENSP00000463559,9606.ENSP00000464359	MYL9,CALML3,MYLK, MYH11,CALML4,MYL12B,MYL12A
STRING clus ters	CL:22574	Muscle protein, and Myosin light chain kinase activity	6	57	2.28	3.90e-10	9606.ENSP00000279022,9606.ENSP00000315299,9606.ENSP00000353452,9606.ENSP00000379616,9606.ENSP00000463559,9606.ENSP00000464359	MYL9,CALML3,MYLK, MYH11,MYL12B,MYL12A
STRING clus ters	CL:22377	Mixed, incl. CaMK IV-mediated phosphorylation of CREB, and MARCKS family	4	19	2.58	2.35e-07	9606.ENSP00000291295,9606.ENSP00000304643,9606.ENSP00000369689,9606.ENSP00000499717	CALM3,CALML6,CALML5,CALM1
STRING clus ters	CL:22378	CREB1 phosphorylation through the activation of CaMKII/CaMKK/ CaMKIV cascasde, and MARCKS family	3	10	2.73	1.30e-05	9606.ENSP00000291295,9606.ENSP00000304643,9606.ENSP00000369689	CALM3,CALML6,CALML5
STRING clus ters	CL:22576	Striated muscle contraction pathway, and Myosin II complex	3	44	2.09	0.00068	9606.ENSP00000279022,9606.ENSP00000463559,9606.ENSP00000464359	MYL9,MYL12B,MYL12A
STRIN	CL:22388	CREB1 phosphorylation	2	5	2.86	0.0017	9606.ENSP00000304643,9606.ENSP00000369689	CALML6,CALML5

G clusters		through the activation of CaMKII/CaMKK/ CaMKIV cascasde, and Sporotrichosis						
STRING clusters	CL:22647	Myosin II filament, and DJBP, EF-hand domain	2	5	2.86	0.0017	9606.ENSP00000279022,9606.ENSP00000463559	MYL9,MYL12B
KEGG	hsa04270	Vascular smooth muscle contraction	8	132	2.04	2.88e-13	9606.ENSP00000279022,9606.ENSP00000291295,9606.ENSP00000304643,9606.ENSP00000315299,9606.ENSP00000353452,9606.ENSP00000369689,9606.ENSP00000379616,9606.ENSP00000419081	MYL9,CALM3,CALML6,CALML3,MYLK,CALML5,MYH11,CALML4
KEGG	hsa04921	Oxytocin signaling pathway	7	147	1.93	8.37e-11	9606.ENSP00000279022,9606.ENSP00000291295,9606.ENSP00000304643,9606.ENSP00000315299,9606.ENSP00000353452,9606.ENSP00000369689,9606.ENSP00000419081	MYL9,CALM3,CALML6,CALML3,MYLK,CALML5,CALML4
KEGG	hsa04022	cGMP-PKG signaling pathway	7	163	1.89	1.13e-10	9606.ENSP00000279022,9606.ENSP00000291295,9606.ENSP00000304643,9606.ENSP00000315299,9606.ENSP00000353452,9606.ENSP00000369689,9606.ENSP00000419081	MYL9,CALM3,CALML6,CALML3,MYLK,CALML5,CALML4
KEGG	hsa04971	Gastric acid secretion	6	71	2.18	1.13e-10	9606.ENSP00000291295,9606.ENSP00000304643,9606.ENSP00000315299,9606.ENSP00000353452,9606.ENSP00000369689,9606.ENSP00000419081	CALM3,CALML6,CALML3,MYLK,CALML5,CALML4
KEGG	hsa04744	Phototransduction	5	26	2.54	2.12e-10	9606.ENSP00000291295,9606.ENSP00000304643,9606.ENSP00000315299,9606.ENSP00000369689,9606.ENSP00000419081	CALM3,CALML6,CALML3,CALML5,CALML4
KEGG	hsa04371	Apelin signaling pathway	6	133	1.91	2.77e-09	9606.ENSP00000291295,9606.ENSP00000304643,9606.ENSP00000315299,9606.ENSP00000353452,9606.ENSP00000369689,9606.ENSP00000419081	CALM3,CALML6,CALML3,MYLK,CALML5,CALML4
KEGG	hsa04720	Long-term potentiation	5	63	2.15	9.18e-09	9606.ENSP00000291295,9606.ENSP00000304643,9606.ENSP00000315299,9606.ENSP00000369689,9606.ENSP00000419081	CALM3,CALML6,CALML3,CALML5,CALML4
KEGG	hsa04924	Renin secretion	5	66	2.13	9.32e-09	9606.ENSP00000291295,9606.ENSP00000304643,9606.ENSP00000315299,9606.ENSP00000369689,9606.ENSP00000419081	CALM3,CALML6,CALML3,CALML5,CALML4
KEGG	hsa05031	Amphetamine addiction	5	65	2.14	9.32e-09	9606.ENSP00000291295,9606.ENSP00000304643,9606.ENSP00000315299,9606.ENSP00000369689,96	CALM3,CALML6,CALML3,CALML5,CALML4

							06.ENSP00000419081	
KEGG	hsa05214	Glioma	5	71	2.1	1.14e-08	9606.ENSP00000291295,9606.ENSP00000304643,9606.ENSP00000315299,9606.ENSP00000369689,9606.ENSP00000419081	CALM3,CALML6,CALML3,CALML5,CALML4
KEGG	hsa04020	Calcium signaling pathway	6	191	1.75	1.18e-08	9606.ENSP00000291295,9606.ENSP00000304643,9606.ENSP00000315299,9606.ENSP00000353452,9606.ENSP00000369689,9606.ENSP00000419081	CALM3,CALML6,CALML3,MYLK,CALML5,CALML4
KEGG	hsa05133	Pertussis	5	73	2.09	1.18e-08	9606.ENSP00000291295,9606.ENSP00000304643,9606.ENSP00000315299,9606.ENSP00000369689,9606.ENSP00000419081	CALM3,CALML6,CALML3,CALML5,CALML4
KEGG	hsa04024	cAMP signaling pathway	6	207	1.72	1.69e-08	9606.ENSP00000279022,9606.ENSP00000291295,9606.ENSP00000304643,9606.ENSP00000315299,9606.ENSP00000369689,9606.ENSP00000419081	MYL9,CALM3,CALML6,CALML3,CALML5,CALML4
KEGG	hsa04912	GnRH signaling pathway	5	87	2.01	2.15e-08	9606.ENSP00000291295,9606.ENSP00000304643,9606.ENSP00000315299,9606.ENSP00000369689,9606.ENSP00000419081	CALM3,CALML6,CALML3,CALML5,CALML4
KEGG	hsa04970	Salivary secretion	5	89	2.0	2.24e-08	9606.ENSP00000291295,9606.ENSP00000304643,9606.ENSP00000315299,9606.ENSP00000369689,9606.ENSP00000419081	CALM3,CALML6,CALML3,CALML5,CALML4
KEGG	hsa04713	Circadian entrainment	5	91	1.99	2.34e-08	9606.ENSP00000291295,9606.ENSP00000304643,9606.ENSP00000315299,9606.ENSP00000369689,9606.ENSP00000419081	CALM3,CALML6,CALML3,CALML5,CALML4
KEGG	hsa04750	Inflammatory mediator regulation of TRP channels	5	92	1.99	2.34e-08	9606.ENSP00000291295,9606.ENSP00000304643,9606.ENSP00000315299,9606.ENSP00000369689,9606.ENSP00000419081	CALM3,CALML6,CALML3,CALML5,CALML4
KEGG	hsa04070	Phosphatidylinositol signaling system	5	94	1.98	2.43e-08	9606.ENSP00000291295,9606.ENSP00000304643,9606.ENSP00000315299,9606.ENSP00000369689,9606.ENSP00000419081	CALM3,CALML6,CALML3,CALML5,CALML4
KEGG	hsa04916	Melanogenesis	5	95	1.97	2.43e-08	9606.ENSP00000291295,9606.ENSP00000304643,9606.ENSP00000315299,9606.ENSP00000369689,9606.ENSP00000419081	CALM3,CALML6,CALML3,CALML5,CALML4
KEGG	hsa04925	Aldosterone synthesis and secretion	5	94	1.98	2.43e-08	9606.ENSP00000291295,9606.ENSP00000304643,9606.ENSP00000315299,9606.ENSP00000369689,9606.ENSP00000419081	CALM3,CALML6,CALML3,CALML5,CALML4
KEGG	hsa04625	C-type lectin receptor signaling pathway	5	101	1.95	2.81e-08	9606.ENSP00000291295,9606.ENSP00000304643,9606.ENSP00000315299,9606.ENSP00000369689,9606.ENSP00000419081	CALM3,CALML6,CALML3,CALML5,CALML4
KEGG	hsa04922	Glucagon signaling pathway	5	100	1.95	2.81e-08	9606.ENSP00000291295,9606.ENSP00000304643,9606.ENSP00000315299,9606.ENSP00000369689,96	CALM3,CALML6,CALML3,CALML5,CALML4

							06.ENSP00000419081	
KEGG	hsa04722	Neurotrophin signaling pathway	5	112	1.9	4.44e-08	9606.ENSP00000291295,9606.ENSP00000304643,9606.ENSP00000315299,9606.ENSP00000369689,9606.ENSP00000419081	CALM3,CALML6,CALML3,CALML5,CALML4
KEGG	hsa04114	Oocyte meiosis	5	121	1.87	6.18e-08	9606.ENSP00000291295,9606.ENSP00000304643,9606.ENSP00000315299,9606.ENSP00000369689,9606.ENSP00000419081	CALM3,CALML6,CALML3,CALML5,CALML4
KEGG	hsa04728	Dopaminergic synapse	5	126	1.85	7.22e-08	9606.ENSP00000291295,9606.ENSP00000304643,9606.ENSP00000315299,9606.ENSP00000369689,9606.ENSP00000419081	CALM3,CALML6,CALML3,CALML5,CALML4
KEGG	hsa05418	Fluid shear stress and atherosclerosis	5	129	1.84	7.79e-08	9606.ENSP00000291295,9606.ENSP00000304643,9606.ENSP00000315299,9606.ENSP00000369689,9606.ENSP00000419081	CALM3,CALML6,CALML3,CALML5,CALML4
KEGG	hsa04910	Insulin signaling pathway	5	132	1.83	8.38e-08	9606.ENSP00000291295,9606.ENSP00000304643,9606.ENSP00000315299,9606.ENSP00000369689,9606.ENSP00000419081	CALM3,CALML6,CALML3,CALML5,CALML4
KEGG	hsa04915	Estrogen signaling pathway	5	133	1.83	8.38e-08	9606.ENSP00000291295,9606.ENSP00000304643,9606.ENSP00000315299,9606.ENSP00000369689,9606.ENSP00000419081	CALM3,CALML6,CALML3,CALML5,CALML4
KEGG	hsa04261	Adrenergic signaling in cardiomyocytes	5	146	1.79	1.27e-07	9606.ENSP00000291295,9606.ENSP00000304643,9606.ENSP00000315299,9606.ENSP00000369689,9606.ENSP00000419081	CALM3,CALML6,CALML3,CALML5,CALML4
KEGG	hsa05034	Alcoholism	5	146	1.79	1.27e-07	9606.ENSP00000291295,9606.ENSP00000304643,9606.ENSP00000315299,9606.ENSP00000369689,9606.ENSP00000419081	CALM3,CALML6,CALML3,CALML5,CALML4
KEGG	hsa04218	Cellular senescence	5	150	1.78	1.36e-07	9606.ENSP00000291295,9606.ENSP00000304643,9606.ENSP00000315299,9606.ENSP00000369689,9606.ENSP00000419081	CALM3,CALML6,CALML3,CALML5,CALML4
KEGG	hsa05152	Tuberculosis	5	165	1.73	2.09e-07	9606.ENSP00000291295,9606.ENSP00000304643,9606.ENSP00000315299,9606.ENSP00000369689,9606.ENSP00000419081	CALM3,CALML6,CALML3,CALML5,CALML4
KEGG	hsa05167	Kaposi sarcoma-associated herpesvirus infection	5	187	1.68	3.73e-07	9606.ENSP00000291295,9606.ENSP00000304643,9606.ENSP00000315299,9606.ENSP00000369689,9606.ENSP00000419081	CALM3,CALML6,CALML3,CALML5,CALML4
KEGG	hsa04015	Rap1 signaling pathway	5	201	1.65	5.15e-07	9606.ENSP00000291295,9606.ENSP00000304643,9606.ENSP00000315299,9606.ENSP00000369689,9606.ENSP00000419081	CALM3,CALML6,CALML3,CALML5,CALML4

KEGG	hsa05170	Human immunodeficiency virus 1 infection	5	203	1.64	5.25e-07	9606.ENSP00000291295,9606.ENSP00000304643,9606.ENSP00000315299,9606.ENSP00000369689,9606.ENSP00000419081	CALM3,CALML6,CALML3,CALML5,CALML4
KEGG	hsa04810	Regulation of actin cytoskeleton	5	209	1.63	5.89e-07	9606.ENSP00000279022,9606.ENSP00000353452,9606.ENSP00000379616,9606.ENSP00000463559,9606.ENSP00000464359	MYL9,MYLK,MYH11,MYL12B,MYL12A
KEGG	hsa05163	Human cytomegalovirus infection	5	217	1.62	6.88e-07	9606.ENSP00000291295,9606.ENSP00000304643,9606.ENSP00000315299,9606.ENSP00000369689,9606.ENSP00000419081	CALM3,CALML6,CALML3,CALML5,CALML4
KEGG	hsa04014	Ras signaling pathway	5	225	1.6	7.99e-07	9606.ENSP00000291295,9606.ENSP00000304643,9606.ENSP00000315299,9606.ENSP00000369689,9606.ENSP00000419081	CALM3,CALML6,CALML3,CALML5,CALML4
KEGG	hsa05012	Parkinson disease	5	236	1.58	9.83e-07	9606.ENSP00000291295,9606.ENSP00000304643,9606.ENSP00000315299,9606.ENSP00000369689,9606.ENSP00000419081	CALM3,CALML6,CALML3,CALML5,CALML4
KEGG	hsa05010	Alzheimer disease	5	354	1.4	6.91e-06	9606.ENSP00000291295,9606.ENSP00000304643,9606.ENSP00000315299,9606.ENSP00000369689,9606.ENSP00000419081	CALM3,CALML6,CALML3,CALML5,CALML4
KEGG	hsa04530	Tight junction	4	157	1.66	1.11e-05	9606.ENSP00000279022,9606.ENSP00000379616,9606.ENSP00000463559,9606.ENSP00000464359	MYL9,MYH11,MYL12B,MYL12A
KEGG	hsa04740	Olfactory transduction	5	417	1.33	1.46e-05	9606.ENSP00000291295,9606.ENSP00000304643,9606.ENSP00000315299,9606.ENSP00000369689,9606.ENSP00000419081	CALM3,CALML6,CALML3,CALML5,CALML4
KEGG	hsa04510	Focal adhesion	4	195	1.57	2.46e-05	9606.ENSP00000279022,9606.ENSP00000353452,9606.ENSP00000463559,9606.ENSP00000464359	MYL9,MYLK,MYL12B,MYL12A
KEGG	hsa05200	Pathways in cancer	5	515	1.24	3.88e-05	9606.ENSP00000291295,9606.ENSP00000304643,9606.ENSP00000315299,9606.ENSP00000369689,9606.ENSP00000419081	CALM3,CALML6,CALML3,CALML5,CALML4
KEGG	hsa04670	Leukocyte transendothelial migration	3	111	1.68	0.00022	9606.ENSP00000279022,9606.ENSP00000463559,9606.ENSP00000464359	MYL9,MYL12B,MYL12A
KEGG	hsa04611	Platelet activation	3	122	1.64	0.00029	9606.ENSP00000353452,9606.ENSP00000463559,9606.ENSP00000464359	MYLK,MYL12B,MYL12A
KEGG	hsa04360	Axon guidance	3	176	1.48	0.00082	9606.ENSP00000279022,9606.ENSP00000463559,9606.ENSP00000464359	MYL9,MYL12B,MYL12A
KEGG	hsa05132	Salmonella infection	3	209	1.41	0.0013	9606.ENSP00000279022,9606.ENSP00000463559,9606.ENSP00000464359	MYL9,MYL12B,MYL12A
KEGG	hsa05131	Shigellosis	3	218	1.39	0.0015	9606.ENSP00000279022,9606.ENSP00000463559,9606.ENSP00000464359	MYL9,MYL12B,MYL12A

Reactome	HSA-445355	Smooth Muscle Contraction	6	43	2.4	1.80e-10	9606.ENSP00000279022,9606.ENSP00000291295,9606.ENSP00000353452,9606.ENSP00000379616,9606.ENSP00000463559,9606.ENSP00000464359	MYL9,CALM3,MYLK,MYH11,MYL12B,MYL12A
Reactome	HSA-5627123	RHO GTPases activate PAKs	5	21	2.63	1.39e-09	9606.ENSP00000279022,9606.ENSP00000291295,9606.ENSP00000353452,9606.ENSP00000379616,9606.ENSP00000463559	MYL9,CALM3,MYLK,MYH11,MYL12B
Reactome	HSA-2682334	EPH-Ephrin signaling	4	92	1.89	9.70e-05	9606.ENSP00000279022,9606.ENSP00000379616,9606.ENSP00000463559,9606.ENSP00000464359	MYL9,MYH11,MYL12B,MYL12A
Reactome	HSA-416572	Sema4D induced cell migration and growth-cone collapse	3	20	2.43	9.70e-05	9606.ENSP00000279022,9606.ENSP00000379616,9606.ENSP00000463559	MYL9,MYH11,MYL12B
Reactome	HSA-5625900	RHO GTPases activate CIT	3	19	2.45	9.70e-05	9606.ENSP00000279022,9606.ENSP00000379616,9606.ENSP00000463559	MYL9,MYH11,MYL12B
Reactome	HSA-5627117	RHO GTPases Activate ROCKs	3	19	2.45	9.70e-05	9606.ENSP00000279022,9606.ENSP00000379616,9606.ENSP00000463559	MYL9,MYH11,MYL12B
Reactome	HSA-3928663	EPHA-mediated growth cone collapse	3	29	2.27	0.00015	9606.ENSP00000279022,9606.ENSP00000379616,9606.ENSP00000463559	MYL9,MYH11,MYL12B
Reactome	HSA-5625740	RHO GTPases activate PKNs	3	63	1.93	0.0012	9606.ENSP00000279022,9606.ENSP00000379616,9606.ENSP00000463559	MYL9,MYH11,MYL12B

Table 9. Pathway Enrichment Analysis for GO, KEGG, STRING clusters and Reactome Terms within MYLK4 gene

#category	term ID	term description	observed	background gene	strength	false discover	matching proteins in your network (IDs)	matching proteins in your network (labels)

y			gene count	count		y rate		
GO Process	GO:0006936	Muscle contraction	5	230	1.59	0.0016	9606.ENSP00000228841,9606.ENSP00000347055,9606.ENSP00000446955,9606.ENSP00000450385,9606.ENSP00000499406	MYL2,MYL4,MYL6,MYL6B,MYL3
GO Process	GO:0002026	Regulation of the force of heart contraction	3	26	2.32	0.0025	9606.ENSP00000228841,9606.ENSP00000347055,9606.ENSP00000499406	MYL2,MYL4,MYL3
GO Process	GO:0006937	Regulation of muscle contraction	4	167	1.63	0.0067	9606.ENSP00000228841,9606.ENSP00000279022,9606.ENSP00000309591,9606.ENSP00000499406	MYL2,MYL9,PRKACA,MYL3
GO Process	GO:0008016	Regulation of heart contraction	4	201	1.55	0.0111	9606.ENSP00000228841,9606.ENSP00000309591,9606.ENSP00000347055,9606.ENSP00000499406	MYL2,PRKACA,MYL4,MYL3
GO Process	GO:0061061	Muscle structure development	5	498	1.25	0.0113	9606.ENSP00000228841,9606.ENSP00000279022,9606.ENSP00000446955,9606.ENSP00000450385,9606.ENSP00000499406	MYL2,MYL9,MYL6,MYL6B,MYL3
GO Process	GO:0044057	Regulation of system process	5	567	1.2	0.0158	9606.ENSP00000228841,9606.ENSP00000279022,9606.ENSP00000309591,9606.ENSP00000347055,9606.ENSP00000499406	MYL2,MYL9,PRKACA,MYL4,MYL3
GO Process	GO:0060048	Cardiac muscle contraction	3	76	1.85	0.0158	9606.ENSP00000228841,9606.ENSP00000347055,9606.ENSP00000499406	MYL2,MYL4,MYL3
GO Process	GO:0006942	Regulation of striated muscle contraction	3	93	1.76	0.0248	9606.ENSP00000228841,9606.ENSP00000309591,9606.ENSP00000499406	MYL2,PRKACA,MYL3
GO Process	GO:0007517	Muscle organ development	4	303	1.37	0.0248	9606.ENSP00000228841,9606.ENSP00000446955,9606.ENSP00000450385,9606.ENSP00000499406	MYL2,MYL6,MYL6B,MYL3
GO Process	GO:0030049	Muscle filament sliding	2	11	2.51	0.0248	9606.ENSP00000446955,9606.ENSP00000450385	MYL6,MYL6B
GO Process	GO:0060537	Muscle tissue development	4	323	1.35	0.0248	9606.ENSP00000228841,9606.ENSP00000446955,9606.ENSP00000450385,9606.ENSP00000499406	MYL2,MYL6,MYL6B,MYL3
GO Process	GO:0034380	High-density lipoprotein particle assembly	2	12	2.47	0.0252	9606.ENSP00000309591,9606.ENSP00000366488	PRKACA,PRKACG
GO	GO:0003008	System process	7	2029	0.79	0.0256	9606.ENSP00000228841,9606.ENSP00000309591,9606.EN	MYL2,PRKACA,MYL4,P

Process							SP00000347055,9606.ENSP00000366488,9606.ENSP00000446955,9606.ENSP00000450385,9606.ENSP00000499406	RKACG,MYL6,MYL6B,MYL3
GO Process	GO:0050878	Regulation of body fluid levels	4	371	1.29	0.0286	9606.ENSP00000279022,9606.ENSP00000309591,9606.ENSP00000366488,9606.ENSP00000464359	MYL9,PRKACA,PRKACG,MYL12A
GO Process	GO:0010737	Protein kinase A signaling	2	17	2.32	0.0343	9606.ENSP00000309591,9606.ENSP00000366488	PRKACA,PRKACG
GO Function	GO:0032036	Myosin heavy chain binding	6	14	2.89	1.08e-12	9606.ENSP00000228841,9606.ENSP00000279022,9606.ENSP00000347055,9606.ENSP00000463559,9606.ENSP00000464359,9606.ENSP00000499406	MYL2,MYL9,MYL4,MYL12B,MYL12A,MYL3
GO Function	GO:0008307	Structural constituent of muscle	5	44	2.31	5.78e-08	9606.ENSP00000228841,9606.ENSP00000279022,9606.ENSP00000446955,9606.ENSP00000450385,9606.ENSP00000499406	MYL2,MYL9,MYL6,MYL6B,MYL3
GO Function	GO:0005509	Calcium ion binding	8	717	1.3	5.95e-07	9606.ENSP00000228841,9606.ENSP00000279022,9606.ENSP00000347055,9606.ENSP00000446955,9606.ENSP00000450385,9606.ENSP00000463559,9606.ENSP00000464359,9606.ENSP00000499406	MYL2,MYL9,MYL4,MYL6,MYL6B,MYL12B,MYL12A,MYL3
GO Function	GO:0003785	Actin monomer binding	3	30	2.25	0.00069	9606.ENSP00000228841,9606.ENSP00000347055,9606.ENSP00000499406	MYL2,MYL4,MYL3
GO Function	GO:0043167	Ion binding	11	6033	0.51	0.0018	9606.ENSP00000228841,9606.ENSP00000274643,9606.ENSP00000279022,9606.ENSP00000309591,9606.ENSP00000347055,9606.ENSP00000366488,9606.ENSP00000446955,9606.ENSP00000450385,9606.ENSP00000463559,9606.ENSP00000464359,9606.ENSP00000499406	MYL2,MYLK4,MYL9,PRKACA,MYL4,PRKACG,MYL6,MYL6B,MYL12B,MYL12A,MYL3
GO Function	GO:0032038	Myosin II heavy chain binding	2	3	3.08	0.0020	9606.ENSP00000347055,9606.ENSP00000499406	MYL4,MYL3
GO Function	GO:0004679	AMP-activated protein kinase activity	2	7	2.71	0.0056	9606.ENSP00000309591,9606.ENSP00000366488	PRKACA,PRKACG
GO Fun	GO:0004691	cAMP-dependent protein kinase	2	8	2.65	0.0063	9606.ENSP00000309591,9606.ENSP00000366488	PRKACA,PRKACG

ction		activity						
GO Function	GO:0046872	Metal ion binding	9	4250	0.58	0.0138	9606.ENSP00000228841,9606.ENSP00000279022,9606.ENSP00000309591,9606.ENSP00000347055,9606.ENSP00000446955,9606.ENSP00000450385,9606.ENSP00000463559,9606.ENSP00000464359,9606.ENSP00000499406	MYL2,MYL9,PRKACA,MYL4,MYL6,MYL6B,MYL12B,MYL12A,MYL3
GO Function	GO:0034237	Protein kinase A regulatory subunit binding	2	25	2.16	0.0304	9606.ENSP00000309591,9606.ENSP00000366488	PRKACA,PRKACG
GO Component	GO:0016459	Myosin complex	8	56	2.41	2.63e-15	9606.ENSP00000228841,9606.ENSP00000279022,9606.ENSP00000347055,9606.ENSP00000446955,9606.ENSP00000450385,9606.ENSP00000463559,9606.ENSP00000464359,9606.ENSP00000499406	MYL2,MYL9,MYL4,MYL6,MYL6B,MYL12B,MYL12A,MYL3
GO Component	GO:0016460	Myosin II complex	7	26	2.68	6.27e-15	9606.ENSP00000279022,9606.ENSP00000347055,9606.ENSP00000446955,9606.ENSP00000450385,9606.ENSP00000463559,9606.ENSP00000464359,9606.ENSP00000499406	MYL9,MYL4,MYL6,MYL6B,MYL12B,MYL12A,MYL3
GO Component	GO:0043292	Contractile fiber	7	246	1.71	1.14e-08	9606.ENSP00000228841,9606.ENSP00000279022,9606.ENSP00000347055,9606.ENSP00000450385,9606.ENSP00000463559,9606.ENSP00000464359,9606.ENSP00000499406	MYL2,MYL9,MYL4,MYL6B,MYL12B,MYL12A,MYL3
GO Component	GO:0030017	Sarcomere	6	217	1.69	3.54e-07	9606.ENSP00000228841,9606.ENSP00000279022,9606.ENSP00000347055,9606.ENSP00000463559,9606.ENSP00000464359,9606.ENSP00000499406	MYL2,MYL9,MYL4,MYL12B,MYL12A,MYL3
GO Component	GO:0005859	Muscle myosin complex	3	15	2.55	3.07e-05	9606.ENSP00000279022,9606.ENSP00000450385,9606.ENSP00000499406	MYL9,MYL6B,MYL3
GO Component	GO:0005856	Cytoskeleton	9	2369	0.83	5.98e-05	9606.ENSP00000228841,9606.ENSP00000279022,9606.ENSP00000309591,9606.ENSP00000347055,9606.ENSP00000446955,9606.ENSP00000450385,9606.ENSP00000463559,9606.ENSP00000464359,9606.ENSP00000499406	MYL2,MYL9,PRKACA,MYL4,MYL6,MYL6B,MYL12B,MYL12A,MYL3
GO Component	GO:0031674	I band	4	146	1.69	0.00019	9606.ENSP00000279022,9606.ENSP00000463559,9606.ENSP00000464359,9606.ENSP00000499406	MYL9,MYL12B,MYL12A,MYL3
GO	GO:0031672	A band	3	38	2.15	0.00023	9606.ENSP00000228841,9606.ENSP00000347055,9606.EN	MYL2,MYL4,MYL3

Component							SP00000499406	
GO Component	GO:0001725	Stress fiber	3	65	1.92	0.00093	9606.ENSP00000279022,9606.ENSP00000463559,9606.ENSP00000464359	MYL9,MYL12B,MYL12A
GO Component	GO:0016461	Unconventional myosin complex	2	8	2.65	0.0014	9606.ENSP00000446955,9606.ENSP00000450385	MYL6,MYL6B
GO Component	GO:0005829	Cytosol	10	5438	0.52	0.0023	9606.ENSP00000228841,9606.ENSP00000279022,9606.ENSP00000309591,9606.ENSP00000347055,9606.ENSP00000366488,9606.ENSP00000446955,9606.ENSP00000450385,9606.ENSP00000463559,9606.ENSP00000464359,9606.ENSP00000499406	MYL2,MYL9,PRKACA,MYL4,PRKACG,MYL6,MYL6B,MYL12B,MYL12A,MYL3
GO Component	GO:0030018	Z disc	3	131	1.61	0.0050	9606.ENSP00000279022,9606.ENSP00000463559,9606.ENSP00000464359	MYL9,MYL12B,MYL12A
GO Component	GO:0032991	Protein-containing complex	9	5506	0.47	0.0280	9606.ENSP00000228841,9606.ENSP00000279022,9606.ENSP00000309591,9606.ENSP00000347055,9606.ENSP00000446955,9606.ENSP00000450385,9606.ENSP00000463559,9606.ENSP00000464359,9606.ENSP00000499406	MYL2,MYL9,PRKACA,MYL4,MYL6,MYL6B,MYL12B,MYL12A,MYL3
GO Component	GO:0097546	Ciliary base	2	46	1.89	0.0280	9606.ENSP00000309591,9606.ENSP00000366488	PRKACA,PRKACG
STRING clusters	CL:22574	Muscle protein, and Myosin light chain kinase activity	9	57	2.45	7.50e-18	9606.ENSP00000228841,9606.ENSP00000274643,9606.ENSP00000279022,9606.ENSP00000347055,9606.ENSP00000446955,9606.ENSP00000450385,9606.ENSP00000463559,9606.ENSP00000464359,9606.ENSP00000499406	MYL2,MYLK4,MYL9,MYL4,MYL6,MYL6B,MYL12B,MYL12A,MYL3
STRING clusters	CL:22576	Striated muscle contraction pathway, and Myosin II complex	8	44	2.51	2.50e-16	9606.ENSP00000228841,9606.ENSP00000279022,9606.ENSP00000347055,9606.ENSP00000446955,9606.ENSP00000450385,9606.ENSP00000463559,9606.ENSP00000464359,9606.ENSP00000499406	MYL2,MYL9,MYL4,MYL6,MYL6B,MYL12B,MYL12A,MYL3
STRING	CL:22629	Myosin II complex	4	15	2.68	9.27e-08	9606.ENSP00000279022,9606.ENSP00000446955,9606.ENSP00000450385,9606.ENSP00000463559	MYL9,MYL6,MYL6B,MYL12B

clust ers								
STR ING clust ers	CL:22647	Myosin II filament, and DJBP, EF-hand domain	3	5	3.03	3.01e-06	9606.ENSP00000279022,9606.ENSP00000446955,9606.EN SP00000463559	MYL9,MYL6,MYL12B
STR ING clust ers	CL:22579	Familial hypertrophic cardiomyopathy	3	12	2.65	2.23e-05	9606.ENSP00000228841,9606.ENSP00000347055,9606.EN SP00000499406	MYL2,MYL4,MYL3
STR ING clust ers	CL:22581	Cardiac myofibril, and Actin-myosin filament sliding	2	6	2.78	0.0026	9606.ENSP00000228841,9606.ENSP00000499406	MYL2,MYL3
STR ING clust ers	CL:10811	CREB1 phosphorylation through the activation of Adenylate Cyclase, and WSK motif	2	11	2.51	0.0067	9606.ENSP00000309591,9606.ENSP00000366488	PRKACA,PRKACG
KE GG	hsa04530	Tight junction	8	157	1.96	1.10e-12	9606.ENSP00000228841,9606.ENSP00000279022,9606.EN SP00000309591,9606.ENSP00000366488,9606.ENSP00000 446955,9606.ENSP00000450385,9606.ENSP00000463559,9 606.ENSP00000464359	MYL2,MYL9,PRKACA,P RKACG,MYL6,MYL6B, MYL12B,MYL12A
KE GG	hsa04270	Vascular smooth muscle contraction	6	132	1.91	7.96e-09	9606.ENSP00000274643,9606.ENSP00000279022,9606.EN SP00000309591,9606.ENSP00000366488,9606.ENSP00000 446955,9606.ENSP00000450385	MYLK4,MYL9,PRKACA ,PRKACG,MYL6,MYL6 B
KE GG	hsa04371	Apelin signaling pathway	6	133	1.91	7.96e-09	9606.ENSP00000228841,9606.ENSP00000274643,9606.EN SP00000309591,9606.ENSP00000347055,9606.ENSP00000 366488,9606.ENSP00000499406	MYL2,MYLK4,PRKACA ,MYL4,PRKACG,MYL3
KE GG	hsa04921	Oxytocin signaling pathway	6	147	1.86	7.96e-09	9606.ENSP00000274643,9606.ENSP00000279022,9606.EN SP00000309591,9606.ENSP00000366488,9606.ENSP00000 446955,9606.ENSP00000450385	MYLK4,MYL9,PRKACA ,PRKACG,MYL6,MYL6 B
KE GG	hsa04611	Platelet activation	5	122	1.87	3.09e-07	9606.ENSP00000274643,9606.ENSP00000309591,9606.EN SP00000366488,9606.ENSP00000463559,9606.ENSP00000 464359	MYLK4,PRKACA,PRKA CG,MYL12B,MYL12A
KE GG	hsa04261	Adrenergic signaling in	5	146	1.79	6.16e-07	9606.ENSP00000228841,9606.ENSP00000309591,9606.EN SP00000347055,9606.ENSP00000366488,9606.ENSP00000	MYL2,PRKACA,MYL4,P RKACG,MYL3

		cardiomyocytes					499406	
KEGG	hsa04510	Focal adhesion	5	195	1.66	2.16e-06	9606.ENSP00000228841,9606.ENSP00000274643,9606.ENSP00000279022,9606.ENSP00000463559,9606.ENSP00000464359	MYL2,MYLK4,MYL9,MYL12B,MYL12A
KEGG	hsa04810	Regulation of actin cytoskeleton	5	209	1.63	2.65e-06	9606.ENSP00000228841,9606.ENSP00000274643,9606.ENSP00000279022,9606.ENSP00000463559,9606.ENSP00000464359	MYL2,MYLK4,MYL9,MYL12B,MYL12A
KEGG	hsa05414	Dilated cardiomyopathy	4	94	1.88	6.89e-06	9606.ENSP00000228841,9606.ENSP00000309591,9606.ENSP00000366488,9606.ENSP00000499406	MYL2,PRKACA,PRKACG,MYL3
KEGG	hsa04670	Leukocyte transendothelial migration	4	111	1.81	1.18e-05	9606.ENSP00000228841,9606.ENSP00000279022,9606.ENSP00000463559,9606.ENSP00000464359	MYL2,MYL9,MYL12B, MYL12A
KEGG	hsa05132	Salmonella infection	4	209	1.53	0.00013	9606.ENSP00000228841,9606.ENSP00000279022,9606.ENSP00000463559,9606.ENSP00000464359	MYL2,MYL9,MYL12B, MYL12A
KEGG	hsa05131	Shigellosis	4	218	1.52	0.00014	9606.ENSP00000228841,9606.ENSP00000279022,9606.ENSP00000463559,9606.ENSP00000464359	MYL2,MYL9,MYL12B, MYL12A
KEGG	hsa04971	Gastric acid secretion	3	71	1.88	0.00021	9606.ENSP00000274643,9606.ENSP00000309591,9606.ENSP00000366488	MYLK4,PRKACA,PRKACG
KEGG	hsa04260	Cardiac muscle contraction	3	87	1.79	0.00036	9606.ENSP00000228841,9606.ENSP00000347055,9606.ENSP00000499406	MYL2,MYL4,MYL3
KEGG	hsa04360	Axon guidance	3	176	1.48	0.0026	9606.ENSP00000279022,9606.ENSP00000463559,9606.ENSP00000464359	MYL9,MYL12B,MYL12A
KEGG	hsa04020	Calcium signaling pathway	3	191	1.45	0.0031	9606.ENSP00000274643,9606.ENSP00000309591,9606.ENSP00000366488	MYLK4,PRKACA,PRKACG
KEGG	hsa04024	cAMP signaling pathway	3	207	1.41	0.0037	9606.ENSP00000279022,9606.ENSP00000309591,9606.ENSP00000366488	MYL9,PRKACA,PRKACG
KEGG	hsa04962	Vasopressin-regulated water reabsorption	2	43	1.92	0.0052	9606.ENSP00000309591,9606.ENSP00000366488	PRKACA,PRKACG
KEGG	hsa04340	Hedgehog signaling pathway	2	46	1.89	0.0056	9606.ENSP00000309591,9606.ENSP00000366488	PRKACA,PRKACG
KEGG	hsa05110	Vibrio cholerae infection	2	47	1.88	0.0056	9606.ENSP00000309591,9606.ENSP00000366488	PRKACA,PRKACG
KEGG	hsa04913	Ovarian steroidogenesis	2	50	1.86	0.0057	9606.ENSP00000309591,9606.ENSP00000366488	PRKACA,PRKACG
KEGG	hsa04961	Endocrine and other factor-	2	51	1.85	0.0057	9606.ENSP00000309591,9606.ENSP00000366488	PRKACA,PRKACG

		regulated calcium reabsorption						
KEGG	hsa05030	Cocaine addiction	2	49	1.86	0.0057	9606.ENSP00000309591,9606.ENSP00000366488	PRKACA,PRKACG
KEGG	hsa04923	Regulation of lipolysis in adipocytes	2	54	1.82	0.0060	9606.ENSP00000309591,9606.ENSP00000366488	PRKACA,PRKACG
KEGG	hsa04213	Longevity regulating pathway - multiple species	2	61	1.77	0.0073	9606.ENSP00000309591,9606.ENSP00000366488	PRKACA,PRKACG
KEGG	hsa04720	Long-term potentiation	2	63	1.75	0.0075	9606.ENSP00000309591,9606.ENSP00000366488	PRKACA,PRKACG
KEGG	hsa04924	Renin secretion	2	66	1.73	0.0076	9606.ENSP00000309591,9606.ENSP00000366488	PRKACA,PRKACG
KEGG	hsa04927	Cortisol synthesis and secretion	2	65	1.74	0.0076	9606.ENSP00000309591,9606.ENSP00000366488	PRKACA,PRKACG
KEGG	hsa05031	Amphetamine addiction	2	65	1.74	0.0076	9606.ENSP00000309591,9606.ENSP00000366488	PRKACA,PRKACG
KEGG	hsa04918	Thyroid hormone synthesis	2	73	1.69	0.0086	9606.ENSP00000309591,9606.ENSP00000366488	PRKACA,PRKACG
KEGG	hsa04742	Taste transduction	2	80	1.65	0.0099	9606.ENSP00000309591,9606.ENSP00000366488	PRKACA,PRKACG
KEGG	hsa04911	Insulin secretion	2	82	1.64	0.0101	9606.ENSP00000309591,9606.ENSP00000366488	PRKACA,PRKACG
KEGG	hsa04727	GABAergic synapse	2	85	1.62	0.0105	9606.ENSP00000309591,9606.ENSP00000366488	PRKACA,PRKACG
KEGG	hsa01522	Endocrine resistance	2	94	1.58	0.0107	9606.ENSP00000309591,9606.ENSP00000366488	PRKACA,PRKACG
KEGG	hsa04211	Longevity regulating pathway	2	87	1.61	0.0107	9606.ENSP00000309591,9606.ENSP00000366488	PRKACA,PRKACG
KEGG	hsa04540	Gap junction	2	87	1.61	0.0107	9606.ENSP00000309591,9606.ENSP00000366488	PRKACA,PRKACG
KEGG	hsa04713	Circadian entrainment	2	91	1.6	0.0107	9606.ENSP00000309591,9606.ENSP00000366488	PRKACA,PRKACG
KEGG	hsa04750	Inflammatory mediator regulation of	2	92	1.59	0.0107	9606.ENSP00000309591,9606.ENSP00000366488	PRKACA,PRKACG

		TRP channels						
KEGG	hsa04912	GnRH signaling pathway	2	87	1.61	0.0107	9606.ENSP00000309591,9606.ENSP00000366488	PRKACA,PRKACG
KEGG	hsa04914	Progesterone-mediated oocyte maturation	2	95	1.58	0.0107	9606.ENSP00000309591,9606.ENSP00000366488	PRKACA,PRKACG
KEGG	hsa04916	Melanogenesis	2	95	1.58	0.0107	9606.ENSP00000309591,9606.ENSP00000366488	PRKACA,PRKACG
KEGG	hsa04922	Glucagon signaling pathway	2	100	1.55	0.0107	9606.ENSP00000309591,9606.ENSP00000366488	PRKACA,PRKACG
KEGG	hsa04925	Aldosterone synthesis and secretion	2	94	1.58	0.0107	9606.ENSP00000309591,9606.ENSP00000366488	PRKACA,PRKACG
KEGG	hsa04928	Parathyroid hormone synthesis, secretion and action	2	104	1.54	0.0107	9606.ENSP00000309591,9606.ENSP00000366488	PRKACA,PRKACG
KEGG	hsa04970	Salivary secretion	2	89	1.6	0.0107	9606.ENSP00000309591,9606.ENSP00000366488	PRKACA,PRKACG
KEGG	hsa04976	Bile secretion	2	88	1.61	0.0107	9606.ENSP00000309591,9606.ENSP00000366488	PRKACA,PRKACG
KEGG	hsa05032	Morphine addiction	2	88	1.61	0.0107	9606.ENSP00000309591,9606.ENSP00000366488	PRKACA,PRKACG
KEGG	hsa05146	Amoebiasis	2	101	1.55	0.0107	9606.ENSP00000309591,9606.ENSP00000366488	PRKACA,PRKACG
KEGG	hsa05410	Hypertrophic cardiomyopathy	2	88	1.61	0.0107	9606.ENSP00000228841,9606.ENSP00000499406	MYL2,MYL3
KEGG	hsa04725	Cholinergic synapse	2	109	1.52	0.0110	9606.ENSP00000309591,9606.ENSP00000366488	PRKACA,PRKACG
KEGG	hsa04726	Serotonergic synapse	2	108	1.52	0.0110	9606.ENSP00000309591,9606.ENSP00000366488	PRKACA,PRKACG
KEGG	hsa04724	Glutamatergic synapse	2	112	1.5	0.0114	9606.ENSP00000309591,9606.ENSP00000366488	PRKACA,PRKACG
KEGG	hsa04935	Growth hormone synthesis, secretion and action	2	117	1.49	0.0122	9606.ENSP00000309591,9606.ENSP00000366488	PRKACA,PRKACG

KEGG	hsa04114	Oocyte meiosis	2	121	1.47	0.0125	9606.ENSP00000309591,9606.ENSP00000366488	PRKACA,PRKACG
KEGG	hsa04919	Thyroid hormone signaling pathway	2	120	1.47	0.0125	9606.ENSP00000309591,9606.ENSP00000366488	PRKACA,PRKACG
KEGG	hsa04728	Dopaminergic synapse	2	126	1.45	0.0133	9606.ENSP00000309591,9606.ENSP00000366488	PRKACA,PRKACG
KEGG	hsa04926	Relaxin signaling pathway	2	126	1.45	0.0133	9606.ENSP00000309591,9606.ENSP00000366488	PRKACA,PRKACG
KEGG	hsa04140	Autophagy - animal	2	131	1.44	0.0138	9606.ENSP00000309591,9606.ENSP00000366488	PRKACA,PRKACG
KEGG	hsa04910	Insulin signaling pathway	2	132	1.43	0.0138	9606.ENSP00000309591,9606.ENSP00000366488	PRKACA,PRKACG
KEGG	hsa04915	Estrogen signaling pathway	2	133	1.43	0.0138	9606.ENSP00000309591,9606.ENSP00000366488	PRKACA,PRKACG
KEGG	hsa04723	Retrograde endocannabinoid signaling	2	142	1.4	0.0154	9606.ENSP00000309591,9606.ENSP00000366488	PRKACA,PRKACG
KEGG	hsa05034	Alcoholism	2	146	1.39	0.0160	9606.ENSP00000309591,9606.ENSP00000366488	PRKACA,PRKACG
KEGG	hsa04310	Wnt signaling pathway	2	154	1.37	0.0172	9606.ENSP00000309591,9606.ENSP00000366488	PRKACA,PRKACG
KEGG	hsa04934	Cushing syndrome	2	153	1.37	0.0172	9606.ENSP00000309591,9606.ENSP00000366488	PRKACA,PRKACG
KEGG	hsa04022	cGMP-PKG signaling pathway	2	163	1.34	0.0188	9606.ENSP00000274643,9606.ENSP00000279022	MYLK4,MYL9
KEGG	hsa05203	Viral carcinogenesis	2	183	1.29	0.0232	9606.ENSP00000309591,9606.ENSP00000366488	PRKACA,PRKACG
KEGG	hsa04062	Chemokine signaling pathway	2	186	1.28	0.0236	9606.ENSP00000309591,9606.ENSP00000366488	PRKACA,PRKACG
KEGG	hsa05205	Proteoglycans in cancer	2	194	1.27	0.0252	9606.ENSP00000309591,9606.ENSP00000366488	PRKACA,PRKACG
KEGG	hsa05166	Human T-cell leukemia virus 1 infection	2	210	1.23	0.0289	9606.ENSP00000309591,9606.ENSP00000366488	PRKACA,PRKACG
KE	hsa05163	Human	2	217	1.22	0.0304	9606.ENSP00000309591,9606.ENSP00000366488	PRKACA,PRKACG

GG		cytomegalovirus infection						
KEGG	hsa04014	Ras signaling pathway	2	225	1.2	0.0321	9606.ENSP00000309591,9606.ENSP00000366488	PRKACA,PRKACG
KEGG	hsa04714	Thermogenesis	2	226	1.2	0.0321	9606.ENSP00000309591,9606.ENSP00000366488	PRKACA,PRKACG
KEGG	hsa05012	Parkinson disease	2	236	1.18	0.0342	9606.ENSP00000309591,9606.ENSP00000366488	PRKACA,PRKACG
KEGG	hsa05020	Prion disease	2	263	1.13	0.0415	9606.ENSP00000309591,9606.ENSP00000366488	PRKACA,PRKACG
KEGG	hsa04010	MAPK signaling pathway	2	286	1.1	0.0481	9606.ENSP00000309591,9606.ENSP00000366488	PRKACA,PRKACG
Reactome	HSA-397014	Muscle contraction	9	203	1.9	2.00e-13	9606.ENSP00000228841,9606.ENSP00000279022,9606.ENSP00000309591,9606.ENSP00000347055,9606.ENSP00000446955,9606.ENSP00000450385,9606.ENSP00000463559,9606.ENSP00000464359,9606.ENSP00000499406	MYL2,MYL9,PRKACA,MYL4,MYL6,MYL6B,MYL12B,MYL12A,MYL3
Reactome	HSA-445355	Smooth Muscle Contraction	5	43	2.32	3.61e-08	9606.ENSP00000279022,9606.ENSP00000446955,9606.ENSP00000450385,9606.ENSP00000463559,9606.ENSP00000464359	MYL9,MYL6,MYL6B,MYL12B,MYL12A
Reactome	HSA-2682334	EPH-Ephrin signaling	4	92	1.89	0.00013	9606.ENSP00000279022,9606.ENSP00000446955,9606.ENSP00000463559,9606.ENSP00000464359	MYL9,MYL6,MYL12B,MYL12A
Reactome	HSA-3928663	EPHA-mediated growth cone collapse	3	29	2.27	0.00013	9606.ENSP00000279022,9606.ENSP00000446955,9606.ENSP00000463559	MYL9,MYL6,MYL12B
Reactome	HSA-416572	Sema4D induced cell migration and growth-cone collapse	3	20	2.43	0.00013	9606.ENSP00000279022,9606.ENSP00000446955,9606.ENSP00000463559	MYL9,MYL6,MYL12B
Reactome	HSA-422475	Axon guidance	6	551	1.29	0.00013	9606.ENSP00000279022,9606.ENSP00000309591,9606.ENSP00000366488,9606.ENSP00000446955,9606.ENSP00000463559,9606.ENSP00000464359	MYL9,PRKACA,PRKACG,MYL6,MYL12B,MYL12A
Reactome	HSA-5625900	RHO GTPases activate CIT	3	19	2.45	0.00013	9606.ENSP00000279022,9606.ENSP00000446955,9606.ENSP00000463559	MYL9,MYL6,MYL12B
Reactome	HSA-5627117	RHO GTPases Activate ROCKs	3	19	2.45	0.00013	9606.ENSP00000279022,9606.ENSP00000446955,9606.ENSP00000463559	MYL9,MYL6,MYL12B
Rea	HSA-5627123	RHO GTPases	3	21	2.41	0.00013	9606.ENSP00000279022,9606.ENSP00000446955,9606.EN	MYL9,MYL6,MYL12B

cto me		activate PAKs					SP00000463559	
Reactome	HSA-390522	Striated Muscle Contraction	3	36	2.17	0.00022	9606.ENSP00000228841,9606.ENSP00000347055,9606.ENSP00000499406	MYL2,MYL4,MYL3
Reactome	HSA-163358	PKA-mediated phosphorylation of key metabolic factors	2	5	2.86	0.0010	9606.ENSP00000309591,9606.ENSP00000366488	PRKACA,PRKACG
Reactome	HSA-5625740	RHO GTPases activate PKNs	3	63	1.93	0.0010	9606.ENSP00000279022,9606.ENSP00000446955,9606.ENSP00000463559	MYL9,MYL6,MYL12B
Reactome	HSA-8963896	HDL assembly	2	8	2.65	0.0017	9606.ENSP00000309591,9606.ENSP00000366488	PRKACA,PRKACG
Reactome	HSA-9010642	ROBO receptors bind AKAP5	2	9	2.6	0.0020	9606.ENSP00000309591,9606.ENSP00000366488	PRKACA,PRKACG
Reactome	HSA-442720	CREB1 phosphorylation through the activation of Adenylate Cyclase	2	12	2.47	0.0031	9606.ENSP00000309591,9606.ENSP00000366488	PRKACA,PRKACG
Reactome	HSA-9634600	Regulation of glycolysis by fructose 2,6-bisphosphate metabolism	2	12	2.47	0.0031	9606.ENSP00000309591,9606.ENSP00000366488	PRKACA,PRKACG
Reactome	HSA-392517	Rap1 signalling	2	16	2.35	0.0047	9606.ENSP00000309591,9606.ENSP00000366488	PRKACA,PRKACG
Reactome	HSA-164378	PKA activation in glucagon signalling	2	17	2.32	0.0050	9606.ENSP00000309591,9606.ENSP00000366488	PRKACA,PRKACG
Reactome	HSA-163615	PKA activation	2	18	2.3	0.0053	9606.ENSP00000309591,9606.ENSP00000366488	PRKACA,PRKACG
Rea	HSA-5621575	CD209 (DC-	2	21	2.23	0.0063	9606.ENSP00000309591,9606.ENSP00000366488	PRKACA,PRKACG

cto me		SIGN) signaling						
Reactome	HSA-163560	Triglyceride catabolism	2	24	2.17	0.0077	9606.ENSP00000309591,9606.ENSP00000366488	PRKACA,PRKACG
Reactome	HSA-180024	DARPP-32 events	2	24	2.17	0.0077	9606.ENSP00000309591,9606.ENSP00000366488	PRKACA,PRKACG
Reactome	HSA-381676	Glucagon-like Peptide-1 (GLP1) regulates insulin secretion	2	42	1.93	0.0170	9606.ENSP00000309591,9606.ENSP00000366488	PRKACA,PRKACG
Reactome	HSA-432040	Vasopressin regulates renal water homeostasis via Aquaporins	2	42	1.93	0.0170	9606.ENSP00000309591,9606.ENSP00000366488	PRKACA,PRKACG
Reactome	HSA-8853659	RET signaling	2	41	1.94	0.0170	9606.ENSP00000309591,9606.ENSP00000366488	PRKACA,PRKACG
Reactome	HSA-9660821	ADORA2B mediated anti-inflammatory cytokines production	2	43	1.92	0.0170	9606.ENSP00000309591,9606.ENSP00000366488	PRKACA,PRKACG
Reactome	HSA-9664323	FCGR3A-mediated IL10 synthesis	2	41	1.94	0.0170	9606.ENSP00000309591,9606.ENSP00000366488	PRKACA,PRKACG
Reactome	HSA-9634597	GPER1 signaling	2	45	1.9	0.0172	9606.ENSP00000309591,9606.ENSP00000366488	PRKACA,PRKACG
Reactome	HSA-5610780	Degradation of GLI1 by the proteasome	2	59	1.78	0.0260	9606.ENSP00000309591,9606.ENSP00000366488	PRKACA,PRKACG
Reactome	HSA-5610783	Degradation of GLI2 by the proteasome	2	59	1.78	0.0260	9606.ENSP00000309591,9606.ENSP00000366488	PRKACA,PRKACG
Rea cto	HSA-5610785	GLI3 is processed to	2	59	1.78	0.0260	9606.ENSP00000309591,9606.ENSP00000366488	PRKACA,PRKACG

me		GLI3R by the proteasome						
Reactome	HSA-5687128	MAPK6/MAPK4 signaling	2	88	1.61	0.0467	9606.ENSP00000309591,9606.ENSP00000366488	PRKACA,PRKACG

Table 10. Pathway Enrichment Analysis for GO, KEGG, STRING clusters and Reactome Terms within PLK4 gene

#category	term ID	term description	observed gene count	background gene count	strength	false discovery rate	matching proteins in your network (IDs)	matching proteins in your network (labels)
GO Process	GO:0007098	Centrosome cycle	10	88	2.31	9.89e-19	9606.ENSP00000257287,9606.ENSP00000270861,9606.ENSP00000287482,9606.ENSP00000300093,9606.ENSP00000360944,9606.ENSP00000370337,9606.ENSP00000370803,9606.ENSP00000371308,9606.ENSP00000378699,9606.ENSP00000427550	CEP135,PLK4,SASS6,PLK1,STIL,CEP152,CCP110,CENPJ,CDK1,CEP192
GO Process	GO:0051298	Centrosome duplication	8	35	2.61	2.21e-16	9606.ENSP00000257287,9606.ENSP00000270861,9606.ENSP00000287482,9606.ENSP00000360944,9606.ENSP00000370337,9606.ENSP00000370803,9606.ENSP00000371308,9606.ENSP00000427550	CEP135,PLK4,SASS6,STIL,CEP152,CCP110,CENPJ,CEP192
GO Proc	GO:0007099	Centriole replication	7	23	2.74	1.15e-14	9606.ENSP00000257287,9606.ENSP00000270861,9606.ENSP00000287482,9606.ENSP00000370337,9606.ENSP00000370803,	CEP135,PLK4,SASS6,CEP152,CCP110,CEN

ess							9606.ENSP00000371308,9606.ENSP00000427550	PJ,CEP192
GO Process	GO:0022402	Cell cycle process	11	835	1.37	2.24e-12	9606.ENSP00000257287,9606.ENSP00000270861,9606.ENSP00000287482,9606.ENSP00000287598,9606.ENSP00000300093,9606.ENSP00000360944,9606.ENSP00000370337,9606.ENSP00000370803,9606.ENSP00000371308,9606.ENSP00000378699,9606.ENSP00000427550	CEP135,PLK4,SASS6,BUB1B,PLK1,STIL,CEP152,CCP110,CENPJ,CDK1,CEP192
GO Process	GO:1902117	Positive regulation of organelle assembly	6	82	2.12	4.74e-09	9606.ENSP00000257287,9606.ENSP00000270861,9606.ENSP00000287482,9606.ENSP00000360944,9606.ENSP00000370803,9606.ENSP00000371308	CEP135,PLK4,SASS6,STIL,CCP110,CENPJ
GO Process	GO:1902115	Regulation of organelle assembly	7	210	1.78	8.07e-09	9606.ENSP00000257287,9606.ENSP00000270861,9606.ENSP00000287482,9606.ENSP00000300093,9606.ENSP00000360944,9606.ENSP00000370803,9606.ENSP00000371308	CEP135,PLK4,SASS6,PLK1,STIL,CCP110,CENPJ
GO Process	GO:0046601	Positive regulation of centriole replication	4	9	2.9	4.52e-08	9606.ENSP00000270861,9606.ENSP00000287482,9606.ENSP00000360944,9606.ENSP00000371308	PLK4,SASS6,STIL,CENPJ
GO Process	GO:0010564	Regulation of cell cycle process	8	716	1.3	4.99e-07	9606.ENSP00000270861,9606.ENSP00000287482,9606.ENSP00000287598,9606.ENSP00000300093,9606.ENSP00000360944,9606.ENSP00000370803,9606.ENSP00000371308,9606.ENSP00000378699	PLK4,SASS6,BUB1B,PLK1,STIL,CCP110,CENPJ,CDK1
GO Process	GO:0090169	Regulation of spindle assembly	4	35	2.31	3.94e-06	9606.ENSP00000287482,9606.ENSP00000300093,9606.ENSP00000360944,9606.ENSP00000371308	SASS6,PLK1,STIL,CENPJ
GO Process	GO:0006996	Organelle organization	11	3470	0.75	4.48e-06	9606.ENSP00000257287,9606.ENSP00000270861,9606.ENSP00000287482,9606.ENSP00000287598,9606.ENSP00000300093,9606.ENSP00000360944,9606.ENSP00000370337,9606.ENSP00000370803,9606.ENSP00000371308,9606.ENSP00000378699,9606.ENSP00000427550	CEP135,PLK4,SASS6,BUB1B,PLK1,STIL,CEP152,CCP110,CENPJ,CDK1,CEP192
GO Process	GO:0060236	Regulation of mitotic spindle organization	4	45	2.2	8.63e-06	9606.ENSP00000287482,9606.ENSP00000300093,9606.ENSP00000360944,9606.ENSP00000371308	SASS6,PLK1,STIL,CENPJ
GO Process	GO:1901990	Regulation of mitotic cell cycle phase transition	6	332	1.51	8.63e-06	9606.ENSP00000287482,9606.ENSP00000287598,9606.ENSP00000300093,9606.ENSP00000360944,9606.ENSP00000371308,9606.ENSP00000378699	SASS6,BUB1B,PLK1,STIL,CENPJ,CDK1
GO Process	GO:1905832	Positive regulation of spindle assembly	3	8	2.83	1.39e-05	9606.ENSP00000287482,9606.ENSP00000360944,9606.ENSP00000371308	SASS6,STIL,CENPJ
GO Process	GO:0033043	Regulation of organelle organization	8	1190	1.08	1.58e-05	9606.ENSP00000257287,9606.ENSP00000270861,9606.ENSP00000287482,9606.ENSP00000287598,9606.ENSP00000300093,9606.ENSP00000360944,9606.ENSP00000370803,9606.ENSP0	CEP135,PLK4,SASS6,BUB1B,PLK1,STIL,CCP110,CENPJ

							0000371308	
GO Process	GO:0090068	Positive regulation of cell cycle process	5	251	1.55	7.55e-05	9606.ENSP00000270861,9606.ENSP00000287482,9606.ENSP00000360944,9606.ENSP00000371308,9606.ENSP00000378699	PLK4,SASS6,STIL,CENPJ,CDK1
GO Process	GO:1901992	Positive regulation of mitotic cell cycle phase transition	4	92	1.89	8.07e-05	9606.ENSP00000287482,9606.ENSP00000360944,9606.ENSP00000371308,9606.ENSP00000378699	SASS6,STIL,CENPJ,CDK1
GO Process	GO:0032886	Regulation of microtubule-based process	5	261	1.54	8.60e-05	9606.ENSP00000270861,9606.ENSP00000287482,9606.ENSP00000300093,9606.ENSP00000360944,9606.ENSP00000371308	PLK4,SASS6,PLK1,STIL,CENPJ
GO Process	GO:0000278	Mitotic cell cycle	6	631	1.23	0.00019	9606.ENSP00000270861,9606.ENSP00000287598,9606.ENSP00000300093,9606.ENSP00000360944,9606.ENSP00000378699,9606.ENSP00000427550	PLK4,BUB1B,PLK1,STIL,CDK1,CEP192
GO Process	GO:0045724	Positive regulation of cilium assembly	3	28	2.28	0.00023	9606.ENSP00000257287,9606.ENSP00000370803,9606.ENSP00000371308	CEP135,CCP110,CENPJ
GO Process	GO:0007051	Spindle organization	4	160	1.65	0.00052	9606.ENSP00000300093,9606.ENSP00000360944,9606.ENSP00000371308,9606.ENSP00000427550	PLK1,STIL,CENPJ,CEP192
GO Process	GO:1900087	Positive regulation of G1/S transition of mitotic cell cycle	3	44	2.09	0.00072	9606.ENSP00000287482,9606.ENSP00000360944,9606.ENSP00000371308	SASS6,STIL,CENPJ
GO Process	GO:0051418	Microtubule nucleation by microtubule organizing center	2	5	2.86	0.0019	9606.ENSP00000371308,9606.ENSP00000427550	CENPJ,CEP192
GO Process	GO:0051493	Regulation of cytoskeleton organization	5	541	1.22	0.0019	9606.ENSP00000270861,9606.ENSP00000287482,9606.ENSP00000300093,9606.ENSP00000360944,9606.ENSP00000371308	PLK4,SASS6,PLK1,STIL,CENPJ
GO Process	GO:0098535	De novo centriole assembly involved in multi-ciliated epithelial cell differentiation	2	5	2.86	0.0019	9606.ENSP00000270861,9606.ENSP00000370337	PLK4,CEP152
GO Process	GO:1903047	Mitotic cell cycle process	5	537	1.22	0.0019	9606.ENSP00000287598,9606.ENSP00000300093,9606.ENSP00000360944,9606.ENSP00000378699,9606.ENSP00000427550	BUB1B,PLK1,STIL,CDK1,CEP192
GO Proc	GO:0022607	Cellular component assembly	8	2467	0.76	0.0020	9606.ENSP00000257287,9606.ENSP00000270861,9606.ENSP00000287482,9606.ENSP00000370337,9606.ENSP00000370803,	CEP135,PLK4,SASS6,CEP152,CCP110,CEN

ess							9606.ENSP00000371308,9606.ENSP00000378699,9606.ENSP00000427550	PJ,CDK1,CEP192
GO Process	GO:1903722	Regulation of centriole elongation	2	7	2.71	0.0028	9606.ENSP00000370803,9606.ENSP00000371308	CCP110,CENPJ
GO Process	GO:0010948	Negative regulation of cell cycle process	4	272	1.42	0.0031	9606.ENSP00000287598,9606.ENSP00000300093,9606.ENSP00000370803,9606.ENSP00000378699	BUB1B,PLK1,CCP110,CDK1
GO Process	GO:1902857	Positive regulation of non-motile cilium assembly	2	8	2.65	0.0034	9606.ENSP00000257287,9606.ENSP00000371308	CEP135,CENPJ
GO Process	GO:0034502	Protein localization to chromosome	3	92	1.77	0.0044	9606.ENSP00000287598,9606.ENSP00000300093,9606.ENSP00000378699	BUB1B,PLK1,CDK1
GO Process	GO:0032465	Regulation of cytokinesis	3	94	1.76	0.0046	9606.ENSP00000270861,9606.ENSP00000300093,9606.ENSP00000370803	PLK4,PLK1,CCP110
GO Process	GO:0007052	Mitotic spindle organization	3	97	1.74	0.0050	9606.ENSP00000300093,9606.ENSP00000360944,9606.ENSP00000427550	PLK1,STIL,CEP192
GO Process	GO:0033365	Protein localization to organelle	5	713	1.1	0.0059	9606.ENSP00000287598,9606.ENSP00000300093,9606.ENSP00000360944,9606.ENSP00000378699,9606.ENSP00000427550	BUB1B,PLK1,STIL,CDK1,CEP192
GO Process	GO:0007093	Mitotic cell cycle checkpoint signaling	3	127	1.63	0.0101	9606.ENSP00000287598,9606.ENSP00000300093,9606.ENSP00000378699	BUB1B,PLK1,CDK1
GO Process	GO:0071539	Protein localization to centrosome	2	22	2.21	0.0171	9606.ENSP00000360944,9606.ENSP00000427550	STIL,CEP192
GO Process	GO:1903829	Positive regulation of protein localization	4	461	1.19	0.0187	9606.ENSP00000257287,9606.ENSP00000300093,9606.ENSP00000371308,9606.ENSP00000378699	CEP135,PLK1,CENPJ,CDK1
GO Process	GO:1901991	Negative regulation of mitotic cell cycle phase transition	3	167	1.51	0.0204	9606.ENSP00000287598,9606.ENSP00000300093,9606.ENSP00000378699	BUB1B,PLK1,CDK1
GO Process	GO:0044772	Mitotic cell cycle phase transition	3	173	1.49	0.0223	9606.ENSP00000287598,9606.ENSP00000300093,9606.ENSP00000378699	BUB1B,PLK1,CDK1
GO	GO:0007	Mitotic spindle	2	31	2.06	0.0286	9606.ENSP00000287598,9606.ENSP00000300093	BUB1B,PLK1

Process	094	assembly checkpoint signaling						
GO Process	GO:0051301	Cell division	4	527	1.13	0.0286	9606.ENSP00000287598,9606.ENSP00000300093,9606.ENSP00000371308,9606.ENSP00000378699	BUB1B,PLK1,CENPJ,CDK1
GO Process	GO:0007095	Mitotic G2 DNA damage checkpoint signaling	2	34	2.02	0.0308	9606.ENSP00000300093,9606.ENSP00000378699	PLK1,CDK1
GO Process	GO:0071459	Protein localization to chromosome, centromeric region	2	41	1.94	0.0392	9606.ENSP00000287598,9606.ENSP00000378699	BUB1B,CDK1
GO Process	GO:0007062	Sister chromatid cohesion	2	42	1.93	0.0403	9606.ENSP00000287598,9606.ENSP00000300093	BUB1B,PLK1
GO Process	GO:0030261	Chromosome condensation	2	42	1.93	0.0403	9606.ENSP00000300093,9606.ENSP00000378699	PLK1,CDK1
GO Component	GO:0005814	Centriole	9	152	2.03	1.07e-14	9606.ENSP00000257287,9606.ENSP00000270861,9606.ENSP00000287482,9606.ENSP00000300093,9606.ENSP00000360944,9606.ENSP00000370337,9606.ENSP00000370803,9606.ENSP00000371308,9606.ENSP00000427550	CEP135,PLK4,SASS6,PLK1,STIL,CEP152,CCP110,CENPJ,CEP192
GO Component	GO:0120099	Procentriole replication complex	6	6	3.25	1.07e-14	9606.ENSP00000270861,9606.ENSP00000287482,9606.ENSP00000360944,9606.ENSP00000370337,9606.ENSP00000371308,9606.ENSP00000427550	PLK4,SASS6,STIL,CEP152,CENPJ,CEP192
GO Component	GO:0005815	Microtubule organizing center	11	825	1.38	5.12e-13	9606.ENSP00000257287,9606.ENSP00000270861,9606.ENSP00000287482,9606.ENSP00000287598,9606.ENSP00000300093,9606.ENSP00000360944,9606.ENSP00000370337,9606.ENSP00000370803,9606.ENSP00000371308,9606.ENSP00000378699,9606.ENSP00000427550	CEP135,PLK4,SASS6,BUB1B,PLK1,STIL,CEP152,CCP110,CENPJ,CDK1,CEP192
GO Component	GO:0005813	Centrosome	10	609	1.47	4.76e-12	9606.ENSP00000257287,9606.ENSP00000270861,9606.ENSP00000287482,9606.ENSP00000300093,9606.ENSP00000360944,9606.ENSP00000370337,9606.ENSP00000370803,9606.ENSP00000371308,9606.ENSP00000378699,9606.ENSP00000427550	CEP135,PLK4,SASS6,PLK1,STIL,CEP152,CCP110,CENPJ,CDK1,CEP192
GO Component	GO:0120098	Procentriole	3	3	3.25	7.56e-07	9606.ENSP00000270861,9606.ENSP00000370337,9606.ENSP00000427550	PLK4,CEP152,CEP192
GO	GO:0098	Deuterosome	3	5	3.03	1.85e-06	9606.ENSP00000270861,9606.ENSP00000287482,9606.ENSP0	PLK4,SASS6,CEP152

Component	536						0000370337	
GO Component	GO:0005829	Cytosol	10	5438	0.52	0.0040	9606.ENSP00000257287,9606.ENSP00000270861,9606.ENSP00000287598,9606.ENSP00000300093,9606.ENSP00000360944,9606.ENSP00000370337,9606.ENSP00000370803,9606.ENSP00000371308,9606.ENSP00000378699,9606.ENSP00000427550	CEP135,PLK4,BUB1B,PLK1,STIL,CEP152,CCP110,CENPJ,CDK1,CEP192
GO Component	GO:0000940	Outer kinetochore	2	12	2.47	0.0041	9606.ENSP00000287598,9606.ENSP00000300093	BUB1B,PLK1
GO Component	GO:0032991	Protein-containing complex	10	5506	0.51	0.0041	9606.ENSP00000270861,9606.ENSP00000287482,9606.ENSP00000287598,9606.ENSP00000300093,9606.ENSP00000360944,9606.ENSP00000370337,9606.ENSP00000370803,9606.ENSP00000371308,9606.ENSP00000378699,9606.ENSP00000427550	PLK4,SASS6,BUB1B,PLK1,STIL,CEP152,CCP110,CENPJ,CDK1,CEP192
GO Component	GO:0005819	Spindle	4	425	1.23	0.0095	9606.ENSP00000270861,9606.ENSP00000287598,9606.ENSP00000300093,9606.ENSP00000378699	PLK4,BUB1B,PLK1,CDK1
GO Component	GO:0000242	Pericentriolar material	2	22	2.21	0.0106	9606.ENSP00000370337,9606.ENSP00000427550	CEP152,CEP192
STRING clusters	CL:25996	Mixed, incl. Centriole replication, and Ciliary rootlet component, centrosome cohesion	8	25	2.76	1.85e-17	9606.ENSP00000257287,9606.ENSP00000270861,9606.ENSP00000287482,9606.ENSP00000360944,9606.ENSP00000370337,9606.ENSP00000370803,9606.ENSP00000371308,9606.ENSP00000427550	CEP135,PLK4,SASS6,STIL,CEP152,CCP110,CENPJ,CEP192
STRING clusters	CL:26000	Procentriole replication complex, and Microcephalic osteodysplastic primordial dwarfism type II	7	11	3.06	1.05e-16	9606.ENSP00000257287,9606.ENSP00000270861,9606.ENSP00000287482,9606.ENSP00000360944,9606.ENSP00000370337,9606.ENSP00000371308,9606.ENSP00000427550	CEP135,PLK4,SASS6,STIL,CEP152,CENPJ,CEP192
STRING clusters	CL:26003	Procentriole replication complex	6	6	3.25	3.42e-15	9606.ENSP00000257287,9606.ENSP00000270861,9606.ENSP00000287482,9606.ENSP00000360944,9606.ENSP00000370337,9606.ENSP00000371308	CEP135,PLK4,SASS6,STIL,CEP152,CENPJ
STR	CL:6644	Mixed, incl.	2	7	2.71	0.0042	9606.ENSP00000287598,9606.ENSP00000300093	BUB1B,PLK1

INGclusters		Activation of NIMA Kinases NEK9, NEK6, NEK7, and Mad3/Bub1 homology region 1						
KEGG	hsa04110	Cell cycle	3	120	1.65	0.0127	9606.ENSP00000287598,9606.ENSP00000300093,9606.ENSP00000378699	BUB1B,PLK1,CDK1
Reactome	HSA-2565942	Regulation of PLK1 Activity at G2/M Transition	8	87	2.22	1.39e-14	9606.ENSP00000257287,9606.ENSP00000270861,9606.ENSP00000300093,9606.ENSP00000370337,9606.ENSP00000370803,9606.ENSP00000371308,9606.ENSP00000378699,9606.ENSP00000427550	CEP135,PLK4,PLK1,CEP152,CCP110,CENPJ,CDK1,CEP192
Reactome	HSA-380259	Loss of Nlp from mitotic centrosomes	8	69	2.32	1.39e-14	9606.ENSP00000257287,9606.ENSP00000270861,9606.ENSP00000300093,9606.ENSP00000370337,9606.ENSP00000370803,9606.ENSP00000371308,9606.ENSP00000378699,9606.ENSP00000427550	CEP135,PLK4,PLK1,CEP152,CCP110,CENPJ,CDK1,CEP192
Reactome	HSA-380270	Recruitment of mitotic centrosome proteins and complexes	8	80	2.25	1.39e-14	9606.ENSP00000257287,9606.ENSP00000270861,9606.ENSP00000300093,9606.ENSP00000370337,9606.ENSP00000370803,9606.ENSP00000371308,9606.ENSP00000378699,9606.ENSP00000427550	CEP135,PLK4,PLK1,CEP152,CCP110,CENPJ,CDK1,CEP192
Reactome	HSA-8854518	AURKA Activation by TPX2	8	72	2.3	1.39e-14	9606.ENSP00000257287,9606.ENSP00000270861,9606.ENSP00000300093,9606.ENSP00000370337,9606.ENSP00000370803,9606.ENSP00000371308,9606.ENSP00000378699,9606.ENSP00000427550	CEP135,PLK4,PLK1,CEP152,CCP110,CENPJ,CDK1,CEP192
Reactome	HSA-380320	Recruitment of NuMA to mitotic centrosomes	8	93	2.19	1.90e-14	9606.ENSP00000257287,9606.ENSP00000270861,9606.ENSP00000300093,9606.ENSP00000370337,9606.ENSP00000370803,9606.ENSP00000371308,9606.ENSP00000378699,9606.ENSP00000427550	CEP135,PLK4,PLK1,CEP152,CCP110,CENPJ,CDK1,CEP192
Reactome	HSA-5620912	Anchoring of the basal body to the plasma membrane	8	97	2.17	2.30e-14	9606.ENSP00000257287,9606.ENSP00000270861,9606.ENSP00000300093,9606.ENSP00000370337,9606.ENSP00000370803,9606.ENSP00000371308,9606.ENSP00000378699,9606.ENSP00000427550	CEP135,PLK4,PLK1,CEP152,CCP110,CENPJ,CDK1,CEP192
Reactome	HSA-68877	Mitotic Prometaphase	9	201	1.9	2.30e-14	9606.ENSP00000257287,9606.ENSP00000270861,9606.ENSP00000287598,9606.ENSP00000300093,9606.ENSP00000370337,9606.ENSP00000370803,9606.ENSP00000371308,9606.ENSP00000378699,9606.ENSP00000427550	CEP135,PLK4,BUB1B,PLK1,CEP152,CCP110,CENPJ,CDK1,CEP192
Reactome	HSA-176417	Phosphorylation of Emi1	2	6	2.78	0.0011	9606.ENSP00000300093,9606.ENSP00000378699	PLK1,CDK1
Rea	HSA-	Regulation of APC/C	3	80	1.83	0.0013	9606.ENSP00000287598,9606.ENSP00000300093,9606.ENSP0	BUB1B,PLK1,CDK1

cto me	176408	activators between G1/S and early anaphase					0000378699	
Reactome	HSA-176814	Activation of APC/C and APC/C:Cdc20 mediated degradation of mitotic proteins	3	76	1.85	0.0013	9606.ENSP00000287598,9606.ENSP00000300093,9606.ENSP00000378699	BUB1B,PLK1,CDK1
Reactome	HSA-2980767	Activation of NIMA Kinases NEK9, NEK6, NEK7	2	7	2.71	0.0013	9606.ENSP00000300093,9606.ENSP00000378699	PLK1,CDK1
Reactome	HSA-162658	Golgi Cisternae Pericentriolar Stack Reorganization	2	14	2.41	0.0034	9606.ENSP00000300093,9606.ENSP00000378699	PLK1,CDK1
Reactome	HSA-2500257	Resolution of Sister Chromatid Cohesion	3	125	1.63	0.0040	9606.ENSP00000287598,9606.ENSP00000300093,9606.ENSP00000378699	BUB1B,PLK1,CDK1
Reactome	HSA-176412	Phosphorylation of the APC/C	2	20	2.25	0.0059	9606.ENSP00000300093,9606.ENSP00000378699	PLK1,CDK1
Reactome	HSA-69273	Cyclin A/B1/B2 associated events during G2/M transition	2	25	2.16	0.0087	9606.ENSP00000300093,9606.ENSP00000378699	PLK1,CDK1
Reactome	HSA-2299718	Condensation of Prophase Chromosomes	2	43	1.92	0.0218	9606.ENSP00000300093,9606.ENSP00000378699	PLK1,CDK1
Reactome	HSA-68882	Mitotic Anaphase	3	232	1.36	0.0218	9606.ENSP00000287598,9606.ENSP00000300093,9606.ENSP00000378699	BUB1B,PLK1,CDK1
Reactome	HSA-6791312	TP53 Regulates Transcription of Cell Cycle Genes	2	49	1.86	0.0271	9606.ENSP00000371308,9606.ENSP00000378699	CENPJ,CDK1
Reactome	HSA-69620	Cell Cycle Checkpoints	3	272	1.3	0.0294	9606.ENSP00000287598,9606.ENSP00000300093,9606.ENSP00000378699	BUB1B,PLK1,CDK1

Table 11. Pathway Enrichment Analysis for GO, KEGG, STRING clusters and Reactome Terms within RPS6KA4 gene

#category	term ID	term description	observed gene count	background gene count	strength	false discovery rate	matching proteins in your network (IDs)	matching proteins in your network (labels)
GO Process	GO:0006468	Protein phosphorylation	8	736	1.29	9.29e-06	9606.ENSP00000211287,9606.ENSP00000215659,9606.ENSP00000229795,9606.ENSP00000264110,9606.ENSP00000333685,9606.ENSP00000333896,9606.ENSP00000387699,9606.ENSP00000479667	MAPK13,MAPK12,MAPK14,ATF2,MAPK11,RPS6KA4,CREB1,RPS6KA5
GO Process	GO:0010604	Positive regulation of macromolecule metabolic process	11	3533	0.75	3.68e-05	9606.ENSP00000211287,9606.ENSP00000215659,9606.ENSP00000229795,9606.ENSP00000264110,9606.ENSP00000333685,9606.ENSP00000333896,9606.ENSP00000336790,9606.ENSP00000350359,9606.ENSP00000364349,9606.ENSP00000387699,9606.ENSP00000479667	MAPK13,MAPK12,MAPK14,ATF2,MAPK11,RPS6KA4,ATF4,CREB5,ATF6B,CREB1,RPS6KA5
GO Process	GO:0035556	Intracellular signal transduction	9	1518	1.03	3.68e-05	9606.ENSP00000211287,9606.ENSP00000215659,9606.ENSP00000229795,9606.ENSP00000264110,9606.ENSP00000333685,9606.ENSP00000333896,9606.ENSP00000336790,9606.ENSP00000387699,9606.ENSP00000479667	MAPK13,MAPK12,MAPK14,ATF2,MAPK11,RPS6KA4,ATF4,CREB1,RPS6KA5
GO Process	GO:0001817	Regulation of cytokine production	7	739	1.23	8.17e-05	9606.ENSP00000211287,9606.ENSP00000229795,9606.ENSP00000264110,9606.ENSP00000333685,9606.ENSP00000333896,9606.ENSP00000336790,9606.ENSP00000479667	MAPK13,MAPK14,ATF2,MAPK11,RPS6KA4,ATF4,RPS6KA5
GO Process	GO:0018105	Peptidyl-serine phosphorylation	5	193	1.67	9.59e-05	9606.ENSP00000211287,9606.ENSP00000215659,9606.ENSP00000229795,9606.ENSP00000333896,9606.ENSP00000479667	MAPK13,MAPK12,MAPK14,RPS6KA4,RPS6KA5
GO Process	GO:0071310	Cellular response to organic substance	9	2019	0.9	0.00011	9606.ENSP00000211287,9606.ENSP00000229795,9606.ENSP00000264110,9606.ENSP00000333685,9606.ENSP00000333896,9606.ENSP00000336790,9606.ENSP00000364349,9606.EN	MAPK13,MAPK14,ATF2,MAPK11,RPS6KA4,ATF4,ATF6B,CREB1,RPS

							SP00000387699,9606.ENSP00000479667	6KA5
GO Process	GO:0000165	MAPK cascade	5	219	1.61	0.00012	9606.ENSP00000211287,9606.ENSP00000215659,9606.ENSP00000229795,9606.ENSP00000264110,9606.ENSP00000333685	MAPK13,MAPK12,MAPK14,ATF2,MAPK11
GO Process	GO:0038066	p38MAPK cascade	3	13	2.62	0.00012	9606.ENSP00000229795,9606.ENSP00000264110,9606.ENSP00000333685	MAPK14,ATF2,MAPK11
GO Process	GO:0051403	Stress-activated MAPK cascade	4	75	1.98	0.00012	9606.ENSP00000211287,9606.ENSP00000229795,9606.ENSP00000264110,9606.ENSP00000333685	MAPK13,MAPK14,ATF2,MAPK11
GO Process	GO:0071347	Cellular response to interleukin-1	4	98	1.86	0.00024	9606.ENSP00000211287,9606.ENSP00000333685,9606.ENSP00000333896,9606.ENSP00000479667	MAPK13,MAPK11,RPS6KA4,RPS6KA5
GO Process	GO:0045893	Positive regulation of transcription, DNA-templated	8	1710	0.92	0.00045	9606.ENSP00000229795,9606.ENSP00000264110,9606.ENSP00000333896,9606.ENSP00000336790,9606.ENSP00000350359,9606.ENSP00000364349,9606.ENSP00000387699,9606.ENSP00000479667	MAPK14,ATF2,RPS6KA4,ATF4,CREB5,ATF6B,CREB1,RPS6KA5
GO Process	GO:0071345	Cellular response to cytokine stimulus	6	711	1.18	0.00062	9606.ENSP00000211287,9606.ENSP00000229795,9606.ENSP00000333685,9606.ENSP00000333896,9606.ENSP00000387699,9606.ENSP00000479667	MAPK13,MAPK14,MAPK11,RPS6KA4,CREB1,RPS6KA5
GO Process	GO:0045944	Positive regulation of transcription by RNA polymerase II	7	1250	1.0	0.00069	9606.ENSP00000229795,9606.ENSP00000264110,9606.ENSP00000333896,9606.ENSP00000336790,9606.ENSP00000364349,9606.ENSP00000387699,9606.ENSP00000479667	MAPK14,ATF2,RPS6KA4,ATF4,ATF6B,CREB1,RPS6KA5
GO Process	GO:0042789	mRNA transcription by RNA polymerase II	3	44	2.09	0.0010	9606.ENSP00000264110,9606.ENSP00000336790,9606.ENSP00000387699	ATF2,ATF4,CREB1
GO Process	GO:0051173	Positive regulation of nitrogen compound metabolic process	9	3166	0.71	0.0014	9606.ENSP00000215659,9606.ENSP00000229795,9606.ENSP00000264110,9606.ENSP00000333896,9606.ENSP00000336790,9606.ENSP00000350359,9606.ENSP00000364349,9606.ENSP00000387699,9606.ENSP00000479667	MAPK12,MAPK14,ATF2,RPS6KA4,ATF4,CREB5,ATF6B,CREB1,RPS6KA5
GO Process	GO:0072740	Cellular response to anisomycin	2	3	3.08	0.0014	9606.ENSP00000211287,9606.ENSP00000264110	MAPK13,ATF2
GO Process	GO:0001819	Positive regulation of cytokine production	5	482	1.27	0.0015	9606.ENSP00000211287,9606.ENSP00000229795,9606.ENSP00000264110,9606.ENSP00000333685,9606.ENSP00000336790	MAPK13,MAPK14,ATF2,MAPK11,ATF4
GO Process	GO:0018193	Peptidyl-amino acid modification	6	900	1.08	0.0015	9606.ENSP00000211287,9606.ENSP00000215659,9606.ENSP00000229795,9606.ENSP00000264110,9606.ENSP00000333896,9606.ENSP00000479667	MAPK13,MAPK12,MAPK14,ATF2,RPS6KA4,RPS6KA5

GO Process	GO:0006950	Response to stress	9	3358	0.68	0.0019	9606.ENSP00000211287,9606.ENSP00000215659,9606.ENSP00000229795,9606.ENSP00000264110,9606.ENSP00000333685,9606.ENSP00000333896,9606.ENSP00000336790,9606.ENSP00000364349,9606.ENSP00000479667	MAPK13,MAPK12,MAPK14,ATF2,MAPK11,RPS6KA4,ATF4,ATF6B,RPS6KA5
GO Process	GO:0007165	Signal transduction	10	4714	0.58	0.0019	9606.ENSP00000211287,9606.ENSP00000215659,9606.ENSP00000229795,9606.ENSP00000264110,9606.ENSP00000333685,9606.ENSP00000333896,9606.ENSP00000336790,9606.ENSP00000364349,9606.ENSP00000387699,9606.ENSP00000479667	MAPK13,MAPK12,MAPK14,ATF2,MAPK11,RPS6KA4,ATF4,ATF6B,CREB1,RPS6KA5
GO Process	GO:0033554	Cellular response to stress	7	1572	0.9	0.0019	9606.ENSP00000211287,9606.ENSP00000215659,9606.ENSP00000229795,9606.ENSP00000264110,9606.ENSP00000333685,9606.ENSP00000336790,9606.ENSP00000364349	MAPK13,MAPK12,MAPK14,ATF2,MAPK11,ATF4,ATF6B
GO Process	GO:0033129	Positive regulation of histone phosphorylation	2	5	2.86	0.0021	9606.ENSP00000333896,9606.ENSP00000479667	RPS6KA4,RPS6KA5
GO Process	GO:0051149	Positive regulation of muscle cell differentiation	3	64	1.92	0.0021	9606.ENSP00000215659,9606.ENSP00000229795,9606.ENSP00000333685	MAPK12,MAPK14,MAPK11
GO Process	GO:0010468	Regulation of gene expression	10	4899	0.56	0.0026	9606.ENSP00000211287,9606.ENSP00000229795,9606.ENSP00000264110,9606.ENSP00000333685,9606.ENSP00000333896,9606.ENSP00000336790,9606.ENSP00000350359,9606.ENSP00000364349,9606.ENSP00000387699,9606.ENSP00000479667	MAPK13,MAPK14,ATF2,MAPK11,RPS6KA4,ATF4,CREB5,ATF6B,CREB1,RPS6KA5
GO Process	GO:0006357	Regulation of transcription by RNA polymerase II	8	2586	0.74	0.0032	9606.ENSP00000229795,9606.ENSP00000264110,9606.ENSP00000333896,9606.ENSP00000336790,9606.ENSP00000350359,9606.ENSP00000364349,9606.ENSP00000387699,9606.ENSP00000479667	MAPK14,ATF2,RPS6KA4,ATF4,CREB5,ATF6B,CREB1,RPS6KA5
GO Process	GO:0098586	Cellular response to virus	3	87	1.79	0.0044	9606.ENSP00000229795,9606.ENSP00000264110,9606.ENSP00000333685	MAPK14,ATF2,MAPK11
GO Process	GO:0051239	Regulation of multicellular organismal process	8	2749	0.72	0.0047	9606.ENSP00000211287,9606.ENSP00000229795,9606.ENSP00000264110,9606.ENSP00000333685,9606.ENSP00000333896,9606.ENSP00000336790,9606.ENSP00000387699,9606.ENSP00000479667	MAPK13,MAPK14,ATF2,MAPK11,RPS6KA4,ATF4,CREB1,RPS6KA5
GO Process	GO:1990440	Positive regulation of transcription from RNA polymerase II promoter in response to endoplasmic reticulum	2	12	2.47	0.0069	9606.ENSP00000336790,9606.ENSP00000364349	ATF4,ATF6B

		stress						
GO Process	GO:0048522	Positive regulation of cellular process	10	5584	0.51	0.0072	9606.ENSP00000215659,9606.ENSP00000229795,9606.ENSP00000264110,9606.ENSP00000333685,9606.ENSP00000333896,9606.ENSP00000336790,9606.ENSP00000350359,9606.ENSP00000364349,9606.ENSP00000387699,9606.ENSP00000479667	MAPK12,MAPK14,ATF2,MAPK11,RPS6KA4,ATF4,CREB5,ATF6B,CREB1,RPS6KA5
GO Process	GO:1990253	Cellular response to leucine starvation	2	13	2.44	0.0076	9606.ENSP00000264110,9606.ENSP00000336790	ATF2,ATF4
GO Process	GO:0006366	Transcription by RNA polymerase II	4	357	1.3	0.0083	9606.ENSP00000229795,9606.ENSP00000264110,9606.ENSP00000336790,9606.ENSP00000387699	MAPK14,ATF2,ATF4,CREB1
GO Process	GO:0032793	Positive regulation of CREB transcription factor activity	2	18	2.3	0.0131	9606.ENSP00000333896,9606.ENSP00000479667	RPS6KA4,RPS6KA5
GO Process	GO:0051240	Positive regulation of multicellular organismal process	6	1505	0.85	0.0154	9606.ENSP00000211287,9606.ENSP00000229795,9606.ENSP00000264110,9606.ENSP00000333685,9606.ENSP00000336790,9606.ENSP00000387699	MAPK13,MAPK14,ATF2,MAPK11,ATF4,CREB1
GO Process	GO:0070498	interleukin-1-mediated signaling pathway	2	20	2.25	0.0154	9606.ENSP00000333896,9606.ENSP00000479667	RPS6KA4,RPS6KA5
GO Process	GO:0071243	Cellular response to arsenic-containing substance	2	20	2.25	0.0154	9606.ENSP00000211287,9606.ENSP00000336790	MAPK13,ATF4
GO Process	GO:1901522	Positive regulation of transcription from RNA polymerase II promoter involved in cellular response to chemical stimulus	2	23	2.19	0.0192	9606.ENSP00000264110,9606.ENSP00000336790	ATF2,ATF4
GO Process	GO:0010243	Response to organonitrogen compound	5	963	0.97	0.0213	9606.ENSP00000211287,9606.ENSP00000229795,9606.ENSP00000264110,9606.ENSP00000336790,9606.ENSP00000387699	MAPK13,MAPK14,ATF2,ATF4,CREB1
GO Process	GO:0071478	Cellular response to radiation	3	183	1.47	0.0266	9606.ENSP00000211287,9606.ENSP00000229795,9606.ENSP00000336790	MAPK13,MAPK14,ATF4
GO Process	GO:0051241	Negative regulation of multicellular organismal process	5	1035	0.94	0.0282	9606.ENSP00000264110,9606.ENSP00000333685,9606.ENSP00000333896,9606.ENSP00000336790,9606.ENSP00000479667	ATF2,MAPK11,RPS6KA4,ATF4,RPS6KA5

GO Process	GO:1901564	Organonitrogen compound metabolic process	9	4981	0.51	0.0282	9606.ENSP00000211287,9606.ENSP00000215659,9606.ENSP00000229795,9606.ENSP00000264110,9606.ENSP00000333685,9606.ENSP00000333896,9606.ENSP00000336790,9606.ENSP00000387699,9606.ENSP00000479667	MAPK13,MAPK12,MAPK14,ATF2,MAPK11,RPS6KA4,ATF4,CREB1,RPS6KA5
GO Process	GO:0010035	Response to inorganic substance	4	532	1.13	0.0295	9606.ENSP00000211287,9606.ENSP00000264110,9606.ENSP00000336790,9606.ENSP00000387699	MAPK13,ATF2,ATF4,CREB1
GO Process	GO:0006984	ER-nucleus signaling pathway	2	33	2.04	0.0308	9606.ENSP00000336790,9606.ENSP00000364349	ATF4,ATF6B
GO Process	GO:0060348	Bone development	3	216	1.4	0.0373	9606.ENSP00000229795,9606.ENSP00000264110,9606.ENSP00000333685	MAPK14,ATF2,MAPK11
GO Process	GO:0034599	Cellular response to oxidative stress	3	224	1.38	0.0400	9606.ENSP00000211287,9606.ENSP00000264110,9606.ENSP00000336790	MAPK13,ATF2,ATF4
GO Process	GO:0060043	Regulation of cardiac muscle cell proliferation	2	39	1.96	0.0400	9606.ENSP00000229795,9606.ENSP00000333685	MAPK14,MAPK11
GO Process	GO:0048519	Negative regulation of biological process	9	5313	0.48	0.0404	9606.ENSP00000215659,9606.ENSP00000229795,9606.ENSP00000264110,9606.ENSP00000333685,9606.ENSP00000333896,9606.ENSP00000336790,9606.ENSP00000364349,9606.ENSP00000387699,9606.ENSP00000479667	MAPK12,MAPK14,ATF2,MAPK11,RPS6KA4,ATF4,ATF6B,CREB1,RPS6KA5
GO Process	GO:0035066	Positive regulation of histone acetylation	2	41	1.94	0.0421	9606.ENSP00000333896,9606.ENSP00000479667	RPS6KA4,RPS6KA5
GO Process	GO:0032735	Positive regulation of interleukin-12 production	2	43	1.92	0.0452	9606.ENSP00000229795,9606.ENSP00000333685	MAPK14,MAPK11
GO Function	GO:0004707	MAP kinase activity	4	15	2.68	9.99e-07	9606.ENSP00000211287,9606.ENSP00000215659,9606.ENSP00000229795,9606.ENSP00000333685	MAPK13,MAPK12,MAPK14,MAPK11
GO Function	GO:0035497	cAMP response element binding	4	17	2.62	9.99e-07	9606.ENSP00000264110,9606.ENSP00000350359,9606.ENSP00000364349,9606.ENSP00000387699	ATF2,CREB5,ATF6B,CREB1
GO Fun	GO:0106310	Protein serine kinase activity	6	361	1.47	2.81e-05	9606.ENSP00000211287,9606.ENSP00000215659,9606.ENSP00000229795,9606.ENSP00000333685,9606.ENSP000003338	MAPK13,MAPK12,MAPK14,MAPK11,RPS6KA4

ctio n							96,9606.ENSP00000479667	,RPS6KA5
GO Function	GO:0004674	Protein serine/threonine kinase activity	6	434	1.39	6.20e-05	9606.ENSP00000211287,9606.ENSP00000215659,9606.ENSP00000229795,9606.ENSP00000333685,9606.ENSP00000333896,9606.ENSP00000479667	MAPK13,MAPK12,MAPK14,MAPK11,RPS6KA4,RPS6KA5
GO Function	GO:0044022	Histone kinase activity (H3-S28 specific)	2	2	3.25	0.0011	9606.ENSP00000333896,9606.ENSP00000479667	RPS6KA4,RPS6KA5
GO Function	GO:0097159	Organic cyclic compound binding	11	6050	0.51	0.0011	9606.ENSP00000211287,9606.ENSP00000215659,9606.ENSP00000229795,9606.ENSP00000264110,9606.ENSP00000333685,9606.ENSP00000333896,9606.ENSP00000336790,9606.ENSP00000350359,9606.ENSP00000364349,9606.ENSP00000387699,9606.ENSP00000479667	MAPK13,MAPK12,MAPK14,ATF2,MAPK11,RPS6KA4,ATF4,CREB5,ATF6B,CREB1,RPS6KA5
GO Function	GO:1901363	Heterocyclic compound binding	11	5977	0.52	0.0011	9606.ENSP00000211287,9606.ENSP00000215659,9606.ENSP00000229795,9606.ENSP00000264110,9606.ENSP00000333685,9606.ENSP00000333896,9606.ENSP00000336790,9606.ENSP00000350359,9606.ENSP00000364349,9606.ENSP00000387699,9606.ENSP00000479667	MAPK13,MAPK12,MAPK14,ATF2,MAPK11,RPS6KA4,ATF4,CREB5,ATF6B,CREB1,RPS6KA5
GO Function	GO:0035175	Histone kinase activity (H3-S10 specific)	2	3	3.08	0.0013	9606.ENSP00000333896,9606.ENSP00000479667	RPS6KA4,RPS6KA5
GO Function	GO:0008140	cAMP response element binding protein binding	2	10	2.55	0.0066	9606.ENSP00000264110,9606.ENSP00000336790	ATF2,ATF4
GO Function	GO:0043522	Leucine zipper domain binding	2	11	2.51	0.0072	9606.ENSP00000264110,9606.ENSP00000336790	ATF2,ATF4
GO Function	GO:0061629	RNA polymerase II-specific DNA-binding transcription factor binding	4	353	1.31	0.0097	9606.ENSP00000229795,9606.ENSP00000264110,9606.ENSP00000336790,9606.ENSP00000387699	MAPK14,ATF2,ATF4,CREB1
GO Function ctio	GO:0005524	ATP binding	6	1491	0.86	0.0152	9606.ENSP00000211287,9606.ENSP00000215659,9606.ENSP00000229795,9606.ENSP00000333685,9606.ENSP00000333896,9606.ENSP00000479667	MAPK13,MAPK12,MAPK14,MAPK11,RPS6KA4,RPS6KA5

n								
GO Function	GO:0016740	Transferase activity	7	2268	0.74	0.0152	9606.ENSP00000211287,9606.ENSP00000215659,9606.ENSP00000229795,9606.ENSP00000264110,9606.ENSP00000333685,9606.ENSP00000333896,9606.ENSP00000479667	MAPK13,MAPK12,MAPK14,ATF2,MAPK11,RPS6KA4,RPS6KA5
GO Function	GO:0140096	Catalytic activity, acting on a protein	7	2279	0.74	0.0152	9606.ENSP00000211287,9606.ENSP00000215659,9606.ENSP00000229795,9606.ENSP00000264110,9606.ENSP00000333685,9606.ENSP00000333896,9606.ENSP00000479667	MAPK13,MAPK12,MAPK14,ATF2,MAPK11,RPS6KA4,RPS6KA5
GO Function	GO:0000287	Magnesium ion binding	3	223	1.38	0.0423	9606.ENSP00000215659,9606.ENSP00000333896,9606.ENSP00000479667	MAPK12,RPS6KA4,RPS6KA5
GO Function	GO:0000978	RNA polymerase II cis-regulatory region sequence-specific DNA binding	5	1196	0.87	0.0449	9606.ENSP00000264110,9606.ENSP00000336790,9606.ENSP00000350359,9606.ENSP00000364349,9606.ENSP00000387699	ATF2,ATF4,CREB5,ATF6B,CREB1
GO Component	GO:1990589	ATF4-CREB1 transcription factor complex	2	4	2.95	0.0087	9606.ENSP00000336790,9606.ENSP00000387699	ATF4,CREB1
GO Component	GO:0090575	RNA polymerase II transcription regulator complex	4	256	1.45	0.0093	9606.ENSP00000264110,9606.ENSP00000336790,9606.ENSP00000364349,9606.ENSP00000387699	ATF2,ATF4,ATF6B,CREB1
GO Component	GO:0005634	Nucleus	11	7672	0.41	0.0214	9606.ENSP00000211287,9606.ENSP00000215659,9606.ENSP00000229795,9606.ENSP00000264110,9606.ENSP00000333685,9606.ENSP00000333896,9606.ENSP00000336790,9606.ENSP00000350359,9606.ENSP00000364349,9606.ENSP00000387699,9606.ENSP00000479667	MAPK13,MAPK12,MAPK14,ATF2,MAPK11,RPS6KA4,ATF4,CREB5,ATF6B,CREB1,RPS6KA5
STRING clusters	CL:18395	Mixed, incl. Basic-leucine zipper domain, and MAPK signaling pathway	11	163	2.08	8.43e-20	9606.ENSP00000211287,9606.ENSP00000215659,9606.ENSP00000229795,9606.ENSP00000264110,9606.ENSP00000333685,9606.ENSP00000333896,9606.ENSP00000336790,9606.ENSP00000350359,9606.ENSP00000364349,9606.ENSP00000387699,9606.ENSP00000479667	MAPK13,MAPK12,MAPK14,ATF2,MAPK11,RPS6KA4,ATF4,CREB5,ATF6B,CREB1,RPS6KA5
STRING clus	CL:18396	Mixed, incl. Basic-leucine zipper domain, and MAPK signaling pathway	8	140	2.01	3.09e-12	9606.ENSP00000211287,9606.ENSP00000215659,9606.ENSP00000229795,9606.ENSP00000264110,9606.ENSP00000333685,9606.ENSP00000336790,9606.ENSP00000350359,9606.ENSP00000387699	MAPK13,MAPK12,MAPK14,ATF2,MAPK11,ATF4,CREB5,CREB1

ters								
STRING clusters	CL:18397	Mixed, incl. Basic-leucine zipper domain, and Host-pathogen interaction of human coronaviruses - MAPK signaling	7	98	2.11	4.91e-11	9606.ENSP00000211287,9606.ENSP00000215659,9606.ENSP00000229795,9606.ENSP00000264110,9606.ENSP00000333685,9606.ENSP00000336790,9606.ENSP00000350359	MAPK13,MAPK12,MAPK14,ATF2,MAPK11,ATF4,CREB5
STRING clusters	CL:18402	Mixed, incl. MAP kinase activity, and Protein tyrosine/threonine phosphatase activity	4	31	2.36	3.12e-06	9606.ENSP00000211287,9606.ENSP00000215659,9606.ENSP00000229795,9606.ENSP00000333685	MAPK13,MAPK12,MAPK14,MAPK11
STRING clusters	CL:18677	CREB3 factors activate genes, and Histone kinase activity (H3-S28 specific)	3	12	2.65	3.35e-05	9606.ENSP00000333896,9606.ENSP00000364349,9606.ENSP00000479667	RPS6KA4,ATF6B,RPS6KA5
STRING clusters	CL:18406	Mixed, incl. Cellular response to sorbitol, and Activation of the AP-1 family of transcription factors	3	15	2.55	5.34e-05	9606.ENSP00000211287,9606.ENSP00000229795,9606.ENSP00000333685	MAPK13,MAPK14,MAPK11
STRING clusters	CL:18506	Response of EIF2AK1 (HRI) to heme deficiency, and Transcription factor AP-1 complex	3	18	2.47	7.83e-05	9606.ENSP00000264110,9606.ENSP00000336790,9606.ENSP00000350359	ATF2,ATF4,CREB5
STRING clusters	CL:18424	MAP kinase activated protein kinase, C-terminal, and Mitogen-activated protein (MAP) kinase p38-like	2	6	2.78	0.0023	9606.ENSP00000211287,9606.ENSP00000333685	MAPK13,MAPK11
KEGG	hsa04668	TNF signaling pathway	11	111	2.25	1.08e-22	9606.ENSP00000211287,9606.ENSP00000215659,9606.ENSP00000229795,9606.ENSP00000264110,9606.ENSP00000333685,9606.ENSP00000333896,9606.ENSP00000336790,9606.ENSP00000350359,9606.ENSP00000364349,9606.ENSP00000387699,9606.ENSP00000479667	MAPK13,MAPK12,MAPK14,ATF2,MAPK11,RPS6KA4,ATF4,CREB5,ATF6B,CREB1,RPS6KA5
KEGG	hsa04261	Adrenergic signaling in cardiomyocytes	10	146	2.09	1.32e-18	9606.ENSP00000211287,9606.ENSP00000215659,9606.ENSP00000229795,9606.ENSP00000264110,9606.ENSP000003336	MAPK13,MAPK12,MAPK14,ATF2,MAPK11,AT

							85,9606.ENSP00000336790,9606.ENSP00000350359,9606.ENSP00000364349,9606.ENSP00000387699,9606.ENSP00000479667	F4,CREB5,ATF6B,CREB1,RPS6KA5
KEGG	hsa04935	Growth hormone synthesis, secretion and action	9	117	2.14	8.13e-17	9606.ENSP00000211287,9606.ENSP00000215659,9606.ENSP00000229795,9606.ENSP00000264110,9606.ENSP00000333685,9606.ENSP00000336790,9606.ENSP00000350359,9606.ENSP00000364349,9606.ENSP00000387699	MAPK13,MAPK12,MAPK14,ATF2,MAPK11,ATF4,CREB5,ATF6B,CREB1
KEGG	hsa04728	Dopaminergic synapse	9	126	2.11	1.16e-16	9606.ENSP00000211287,9606.ENSP00000215659,9606.ENSP00000229795,9606.ENSP00000264110,9606.ENSP00000333685,9606.ENSP00000336790,9606.ENSP00000350359,9606.ENSP00000364349,9606.ENSP00000387699	MAPK13,MAPK12,MAPK14,ATF2,MAPK11,ATF4,CREB5,ATF6B,CREB1
KEGG	hsa04926	Relaxin signaling pathway	9	126	2.11	1.16e-16	9606.ENSP00000211287,9606.ENSP00000215659,9606.ENSP00000229795,9606.ENSP00000264110,9606.ENSP00000333685,9606.ENSP00000336790,9606.ENSP00000350359,9606.ENSP00000364349,9606.ENSP00000387699	MAPK13,MAPK12,MAPK14,ATF2,MAPK11,ATF4,CREB5,ATF6B,CREB1
KEGG	hsa05161	Hepatitis B	9	158	2.01	5.50e-16	9606.ENSP00000211287,9606.ENSP00000215659,9606.ENSP00000229795,9606.ENSP00000264110,9606.ENSP00000333685,9606.ENSP00000336790,9606.ENSP00000350359,9606.ENSP00000364349,9606.ENSP00000387699	MAPK13,MAPK12,MAPK14,ATF2,MAPK11,ATF4,CREB5,ATF6B,CREB1
KEGG	hsa05163	Human cytomegalovirus infection	9	217	1.87	7.56e-15	9606.ENSP00000211287,9606.ENSP00000215659,9606.ENSP00000229795,9606.ENSP00000264110,9606.ENSP00000333685,9606.ENSP00000336790,9606.ENSP00000350359,9606.ENSP00000364349,9606.ENSP00000387699	MAPK13,MAPK12,MAPK14,ATF2,MAPK11,ATF4,CREB5,ATF6B,CREB1
KEGG	hsa05020	Prion disease	9	263	1.79	3.59e-14	9606.ENSP00000211287,9606.ENSP00000215659,9606.ENSP00000229795,9606.ENSP00000264110,9606.ENSP00000333685,9606.ENSP00000336790,9606.ENSP00000350359,9606.ENSP00000364349,9606.ENSP00000387699	MAPK13,MAPK12,MAPK14,ATF2,MAPK11,ATF4,CREB5,ATF6B,CREB1
KEGG	hsa04010	MAPK signaling pathway	8	286	1.7	1.32e-11	9606.ENSP00000211287,9606.ENSP00000215659,9606.ENSP00000229795,9606.ENSP00000264110,9606.ENSP00000333685,9606.ENSP00000333896,9606.ENSP00000336790,9606.ENSP00000479667	MAPK13,MAPK12,MAPK14,ATF2,MAPK11,RPS6KA4,ATF4,RPS6KA5
KEGG	hsa04714	Thermogenesis	7	226	1.74	3.14e-10	9606.ENSP00000211287,9606.ENSP00000215659,9606.ENSP00000229795,9606.ENSP00000264110,9606.ENSP00000333685,9606.ENSP00000350359,9606.ENSP00000387699	MAPK13,MAPK12,MAPK14,ATF2,MAPK11,CREB5,CREB1
KEGG	hsa04722	Neurotrophin signaling pathway	6	112	1.98	5.58e-10	9606.ENSP00000211287,9606.ENSP00000215659,9606.ENSP00000229795,9606.ENSP00000333685,9606.ENSP00000336790,9606.ENSP00000479667	MAPK13,MAPK12,MAPK14,MAPK11,ATF4,RPS6KA5
KEGG	hsa05030	Cocaine addiction	5	49	2.26	1.63e-09	9606.ENSP00000264110,9606.ENSP00000336790,9606.ENSP00000350359,9606.ENSP00000364349,9606.ENSP000003876	ATF2,ATF4,CREB5,ATF6B,CREB1

KEGG	hsa04927	Cortisol synthesis and secretion	5	65	2.14	5.74e-09	9606.ENSP00000264110,9606.ENSP00000336790,9606.ENSP00000350359,9606.ENSP00000364349,9606.ENSP00000387699	ATF2,ATF4,CREB5,ATF6B,CREB1
KEGG	hsa05031	Amphetamine addiction	5	65	2.14	5.74e-09	9606.ENSP00000264110,9606.ENSP00000336790,9606.ENSP00000350359,9606.ENSP00000364349,9606.ENSP00000387699	ATF2,ATF4,CREB5,ATF6B,CREB1
KEGG	hsa04918	Thyroid hormone synthesis	5	73	2.09	8.66e-09	9606.ENSP00000264110,9606.ENSP00000336790,9606.ENSP00000350359,9606.ENSP00000364349,9606.ENSP00000387699	ATF2,ATF4,CREB5,ATF6B,CREB1
KEGG	hsa04911	Insulin secretion	5	82	2.04	1.42e-08	9606.ENSP00000264110,9606.ENSP00000336790,9606.ENSP00000350359,9606.ENSP00000364349,9606.ENSP00000387699	ATF2,ATF4,CREB5,ATF6B,CREB1
KEGG	hsa04211	Longevity regulating pathway	5	87	2.01	1.77e-08	9606.ENSP00000264110,9606.ENSP00000336790,9606.ENSP00000350359,9606.ENSP00000364349,9606.ENSP00000387699	ATF2,ATF4,CREB5,ATF6B,CREB1
KEGG	hsa04912	GnRH signaling pathway	5	87	2.01	1.77e-08	9606.ENSP00000211287,9606.ENSP00000215659,9606.ENSP00000229795,9606.ENSP00000333685,9606.ENSP00000336790	MAPK13,MAPK12,MAPK14,MAPK11,ATF4
KEGG	hsa04925	Aldosterone synthesis and secretion	5	94	1.98	2.30e-08	9606.ENSP00000264110,9606.ENSP00000336790,9606.ENSP00000350359,9606.ENSP00000364349,9606.ENSP00000387699	ATF2,ATF4,CREB5,ATF6B,CREB1
KEGG	hsa04928	Parathyroid hormone synthesis, secretion and action	5	104	1.93	3.56e-08	9606.ENSP00000264110,9606.ENSP00000336790,9606.ENSP00000350359,9606.ENSP00000364349,9606.ENSP00000387699	ATF2,ATF4,CREB5,ATF6B,CREB1
KEGG	hsa04380	Osteoclast differentiation	5	120	1.87	6.79e-08	9606.ENSP00000211287,9606.ENSP00000215659,9606.ENSP00000229795,9606.ENSP00000333685,9606.ENSP00000387699	MAPK13,MAPK12,MAPK14,MAPK11,CREB1
KEGG	hsa04915	Estrogen signaling pathway	5	133	1.83	1.07e-07	9606.ENSP00000264110,9606.ENSP00000336790,9606.ENSP00000350359,9606.ENSP00000364349,9606.ENSP00000387699	ATF2,ATF4,CREB5,ATF6B,CREB1
KEGG	hsa05034	Alcoholism	5	146	1.79	1.61e-07	9606.ENSP00000264110,9606.ENSP00000336790,9606.ENSP00000350359,9606.ENSP00000364349,9606.ENSP00000387699	ATF2,ATF4,CREB5,ATF6B,CREB1
KEGG	hsa04934	Cushing syndrome	5	153	1.77	1.93e-07	9606.ENSP00000264110,9606.ENSP00000336790,9606.ENSP00000350359,9606.ENSP00000364349,9606.ENSP00000387699	ATF2,ATF4,CREB5,ATF6B,CREB1
KEGG	hsa04022	cGMP-PKG signaling pathway	5	163	1.74	2.53e-07	9606.ENSP00000264110,9606.ENSP00000336790,9606.ENSP00000350359,9606.ENSP00000364349,9606.ENSP000003876	ATF2,ATF4,CREB5,ATF6B,CREB1

KEGG	hsa05152	Tuberculosis	5	165	1.73	2.58e-07	9606.ENSP00000211287,9606.ENSP00000215659,9606.ENSP00000229795,9606.ENSP00000333685,9606.ENSP00000387699	MAPK13,MAPK12,MAPK14,MAPK11,CREB1
KEGG	hsa04370	VEGF signaling pathway	4	56	2.11	3.14e-07	9606.ENSP00000211287,9606.ENSP00000215659,9606.ENSP00000229795,9606.ENSP00000333685	MAPK13,MAPK12,MAPK14,MAPK11
KEGG	hsa05203	Viral carcinogenesis	5	183	1.69	3.96e-07	9606.ENSP00000264110,9606.ENSP00000336790,9606.ENSP00000350359,9606.ENSP00000364349,9606.ENSP00000387699	ATF2,ATF4,CREB5,ATF6B,CREB1
KEGG	hsa05167	Kaposi sarcoma-associated herpesvirus infection	5	187	1.68	4.25e-07	9606.ENSP00000211287,9606.ENSP00000215659,9606.ENSP00000229795,9606.ENSP00000333685,9606.ENSP00000387699	MAPK13,MAPK12,MAPK14,MAPK11,CREB1
KEGG	hsa04664	Fc epsilon RI signaling pathway	4	65	2.04	4.99e-07	9606.ENSP00000211287,9606.ENSP00000215659,9606.ENSP00000229795,9606.ENSP00000333685	MAPK13,MAPK12,MAPK14,MAPK11
KEGG	hsa05120	Epithelial cell signaling in Helicobacter pylori infection	4	65	2.04	4.99e-07	9606.ENSP00000211287,9606.ENSP00000215659,9606.ENSP00000229795,9606.ENSP00000333685	MAPK13,MAPK12,MAPK14,MAPK11
KEGG	hsa04917	Prolactin signaling pathway	4	68	2.02	5.56e-07	9606.ENSP00000211287,9606.ENSP00000215659,9606.ENSP00000229795,9606.ENSP00000333685	MAPK13,MAPK12,MAPK14,MAPK11
KEGG	hsa04622	RIG-I-like receptor signaling pathway	4	69	2.02	5.70e-07	9606.ENSP00000211287,9606.ENSP00000215659,9606.ENSP00000229795,9606.ENSP00000333685	MAPK13,MAPK12,MAPK14,MAPK11
KEGG	hsa05140	Leishmaniasis	4	69	2.02	5.70e-07	9606.ENSP00000211287,9606.ENSP00000215659,9606.ENSP00000229795,9606.ENSP00000333685	MAPK13,MAPK12,MAPK14,MAPK11
KEGG	hsa05166	Human T-cell leukemia virus 1 infection	5	210	1.63	6.20e-07	9606.ENSP00000264110,9606.ENSP00000336790,9606.ENSP00000350359,9606.ENSP00000364349,9606.ENSP00000387699	ATF2,ATF4,CREB5,ATF6B,CREB1
KEGG	hsa05133	Pertussis	4	73	1.99	6.49e-07	9606.ENSP00000211287,9606.ENSP00000215659,9606.ENSP00000229795,9606.ENSP00000333685	MAPK13,MAPK12,MAPK14,MAPK11
KEGG	hsa05131	Shigellosis	5	218	1.61	7.04e-07	9606.ENSP00000211287,9606.ENSP00000215659,9606.ENSP00000229795,9606.ENSP00000333685,9606.ENSP00000479667	MAPK13,MAPK12,MAPK14,MAPK11,RPS6KA5
KEGG	hsa04658	Th1 and Th2 cell differentiation	4	85	1.93	1.11e-06	9606.ENSP00000211287,9606.ENSP00000215659,9606.ENSP00000229795,9606.ENSP00000333685	MAPK13,MAPK12,MAPK14,MAPK11
KEGG	hsa05235	PD-L1 expression and PD-1 checkpoint pathway in cancer	4	87	1.92	1.18e-06	9606.ENSP00000211287,9606.ENSP00000215659,9606.ENSP00000229795,9606.ENSP00000333685	MAPK13,MAPK12,MAPK14,MAPK11
KEGG	hsa04657	IL-17 signaling pathway	4	91	1.9	1.37e-06	9606.ENSP00000211287,9606.ENSP00000215659,9606.ENSP00000229795,9606.ENSP00000333685	MAPK13,MAPK12,MAPK14,MAPK11

KEGG	hsa04750	Inflammatory mediator regulation of TRP channels	4	92	1.89	1.39e-06	9606.ENSP00000211287,9606.ENSP00000215659,9606.ENSP00000229795,9606.ENSP00000333685	MAPK13,MAPK12,MAPK14,MAPK11
KEGG	hsa01522	Endocrine resistance	4	94	1.88	1.48e-06	9606.ENSP00000211287,9606.ENSP00000215659,9606.ENSP00000229795,9606.ENSP00000333685	MAPK13,MAPK12,MAPK14,MAPK11
KEGG	hsa04914	Progesterone-mediated oocyte maturation	4	95	1.88	1.50e-06	9606.ENSP00000211287,9606.ENSP00000215659,9606.ENSP00000229795,9606.ENSP00000333685	MAPK13,MAPK12,MAPK14,MAPK11
KEGG	hsa04933	AGE-RAGE signaling pathway in diabetic complications	4	96	1.87	1.53e-06	9606.ENSP00000211287,9606.ENSP00000215659,9606.ENSP00000229795,9606.ENSP00000333685	MAPK13,MAPK12,MAPK14,MAPK11
KEGG	hsa05142	Chagas disease	4	97	1.87	1.56e-06	9606.ENSP00000211287,9606.ENSP00000215659,9606.ENSP00000229795,9606.ENSP00000333685	MAPK13,MAPK12,MAPK14,MAPK11
KEGG	hsa04659	Th17 cell differentiation	4	99	1.86	1.65e-06	9606.ENSP00000211287,9606.ENSP00000215659,9606.ENSP00000229795,9606.ENSP00000333685	MAPK13,MAPK12,MAPK14,MAPK11
KEGG	hsa04620	Toll-like receptor signaling pathway	4	100	1.86	1.68e-06	9606.ENSP00000211287,9606.ENSP00000215659,9606.ENSP00000229795,9606.ENSP00000333685	MAPK13,MAPK12,MAPK14,MAPK11
KEGG	hsa04625	C-type lectin receptor signaling pathway	4	101	1.85	1.68e-06	9606.ENSP00000211287,9606.ENSP00000215659,9606.ENSP00000229795,9606.ENSP00000333685	MAPK13,MAPK12,MAPK14,MAPK11
KEGG	hsa04660	T cell receptor signaling pathway	4	100	1.86	1.68e-06	9606.ENSP00000211287,9606.ENSP00000215659,9606.ENSP00000229795,9606.ENSP00000333685	MAPK13,MAPK12,MAPK14,MAPK11
KEGG	hsa04922	Glucagon signaling pathway	4	100	1.86	1.68e-06	9606.ENSP00000264110,9606.ENSP00000336790,9606.ENSP00000350359,9606.ENSP00000387699	ATF2,ATF4,CREB5,CREB1
KEGG	hsa05145	Toxoplasmosis	4	103	1.84	1.73e-06	9606.ENSP00000211287,9606.ENSP00000215659,9606.ENSP00000229795,9606.ENSP00000333685	MAPK13,MAPK12,MAPK14,MAPK11
KEGG	hsa04670	Leukocyte transendothelial migration	4	111	1.81	2.27e-06	9606.ENSP00000211287,9606.ENSP00000215659,9606.ENSP00000229795,9606.ENSP00000333685	MAPK13,MAPK12,MAPK14,MAPK11
KEGG	hsa04071	Sphingolipid signaling pathway	4	116	1.79	2.64e-06	9606.ENSP00000211287,9606.ENSP00000215659,9606.ENSP00000229795,9606.ENSP00000333685	MAPK13,MAPK12,MAPK14,MAPK11
KEGG	hsa04114	Oocyte meiosis	4	121	1.77	3.06e-06	9606.ENSP00000211287,9606.ENSP00000215659,9606.ENSP00000229795,9606.ENSP00000333685	MAPK13,MAPK12,MAPK14,MAPK11
KEGG	hsa04611	Platelet activation	4	122	1.77	3.10e-06	9606.ENSP00000211287,9606.ENSP00000215659,9606.ENSP00000229795,9606.ENSP00000333685	MAPK13,MAPK12,MAPK14,MAPK11
KEGG	hsa05135	Yersinia infection	4	124	1.76	3.24e-06	9606.ENSP00000211287,9606.ENSP00000215659,9606.ENSP00000229795,9606.ENSP00000333685	MAPK13,MAPK12,MAPK14,MAPK11
KEGG	hsa04068	FoxO signaling pathway	4	126	1.75	3.39e-06	9606.ENSP00000211287,9606.ENSP00000215659,9606.ENSP00000229795,9606.ENSP00000333685	MAPK13,MAPK12,MAPK14,MAPK11
KEGG	hsa05418	Fluid shear stress and atherosclerosis	4	129	1.74	3.65e-06	9606.ENSP00000211287,9606.ENSP00000215659,9606.ENSP00000229795,9606.ENSP00000333685	MAPK13,MAPK12,MAPK14,MAPK11

KEGG	hsa04151	PI3K-Akt signaling pathway	5	349	1.41	4.37e-06	9606.ENSP00000264110,9606.ENSP00000336790,9606.ENSP00000350359,9606.ENSP00000364349,9606.ENSP00000387699	ATF2,ATF4,CREB5,ATF6B,CREB1
KEGG	hsa05014	Amyotrophic lateral sclerosis	5	350	1.41	4.37e-06	9606.ENSP00000211287,9606.ENSP00000215659,9606.ENSP00000229795,9606.ENSP00000333685,9606.ENSP00000336790	MAPK13,MAPK12,MAPK14,MAPK11,ATF4
KEGG	hsa04550	Signaling pathways regulating pluripotency of stem cells	4	141	1.71	4.91e-06	9606.ENSP00000211287,9606.ENSP00000215659,9606.ENSP00000229795,9606.ENSP00000333685	MAPK13,MAPK12,MAPK14,MAPK11
KEGG	hsa04723	Retrograde endocannabinoid signaling	4	142	1.7	4.96e-06	9606.ENSP00000211287,9606.ENSP00000215659,9606.ENSP00000229795,9606.ENSP00000333685	MAPK13,MAPK12,MAPK14,MAPK11
KEGG	hsa04218	Cellular senescence	4	150	1.68	6.04e-06	9606.ENSP00000211287,9606.ENSP00000215659,9606.ENSP00000229795,9606.ENSP00000333685	MAPK13,MAPK12,MAPK14,MAPK11
KEGG	hsa04621	NOD-like receptor signaling pathway	4	173	1.62	1.04e-05	9606.ENSP00000211287,9606.ENSP00000215659,9606.ENSP00000229795,9606.ENSP00000333685	MAPK13,MAPK12,MAPK14,MAPK11
KEGG	hsa05130	Pathogenic Escherichia coli infection	4	187	1.58	1.38e-05	9606.ENSP00000211287,9606.ENSP00000215659,9606.ENSP00000229795,9606.ENSP00000333685	MAPK13,MAPK12,MAPK14,MAPK11
KEGG	hsa05169	Epstein-Barr virus infection	4	192	1.57	1.51e-05	9606.ENSP00000211287,9606.ENSP00000215659,9606.ENSP00000229795,9606.ENSP00000333685	MAPK13,MAPK12,MAPK14,MAPK11
KEGG	hsa05205	Proteoglycans in cancer	4	194	1.57	1.55e-05	9606.ENSP00000211287,9606.ENSP00000215659,9606.ENSP00000229795,9606.ENSP00000333685	MAPK13,MAPK12,MAPK14,MAPK11
KEGG	hsa04015	Rap1 signaling pathway	4	201	1.55	1.75e-05	9606.ENSP00000211287,9606.ENSP00000215659,9606.ENSP00000229795,9606.ENSP00000333685	MAPK13,MAPK12,MAPK14,MAPK11
KEGG	hsa05170	Human immunodeficiency virus 1 infection	4	203	1.55	1.79e-05	9606.ENSP00000211287,9606.ENSP00000215659,9606.ENSP00000229795,9606.ENSP00000333685	MAPK13,MAPK12,MAPK14,MAPK11
KEGG	hsa05132	Salmonella infection	4	209	1.53	1.98e-05	9606.ENSP00000211287,9606.ENSP00000215659,9606.ENSP00000229795,9606.ENSP00000333685	MAPK13,MAPK12,MAPK14,MAPK11
KEGG	hsa05215	Prostate cancer	3	97	1.74	9.61e-05	9606.ENSP00000336790,9606.ENSP00000350359,9606.ENSP00000387699	ATF4,CREB5,CREB1
KEGG	hsa04725	Cholinergic synapse	3	109	1.69	0.00013	9606.ENSP00000336790,9606.ENSP00000350359,9606.ENSP00000387699	ATF4,CREB5,CREB1
KEGG	hsa04962	Vasopressin-regulated water reabsorption	2	43	1.92	0.0013	9606.ENSP00000350359,9606.ENSP00000387699	CREB5,CREB1
KEGG	hsa04713	Circadian entrainment	2	91	1.6	0.0054	9606.ENSP00000387699,9606.ENSP00000479667	CREB1,RPS6KA5
KEGG	hsa04931	Insulin resistance	2	106	1.53	0.0071	9606.ENSP00000350359,9606.ENSP00000387699	CREB5,CREB1

KEGG	hsa04152	AMPK signaling pathway	2	120	1.47	0.0089	9606.ENSP00000350359,9606.ENSP00000387699	CREB5,CREB1
KEGG	hsa04141	Protein processing in endoplasmic reticulum	2	163	1.34	0.0159	9606.ENSP00000336790,9606.ENSP00000364349	ATF4,ATF6B
KEGG	hsa04024	cAMP signaling pathway	2	207	1.24	0.0249	9606.ENSP00000350359,9606.ENSP00000387699	CREB5,CREB1
KEGG	hsa05016	Huntington disease	2	295	1.08	0.0484	9606.ENSP00000350359,9606.ENSP00000387699	CREB5,CREB1
Reactome	HSA-187037	Signaling by NTRK1 (TRKA)	7	115	2.04	2.16e-10	9606.ENSP00000211287,9606.ENSP00000215659,9606.ENSP00000229795,9606.ENSP00000264110,9606.ENSP00000333685,9606.ENSP00000387699,9606.ENSP00000479667	MAPK13,MAPK12,MAPK14,ATF2,MAPK11,CREB1,RPS6KA5
Reactome	HSA-450282	MAPK targets/ Nuclear events mediated by MAP kinases	5	30	2.47	4.57e-09	9606.ENSP00000229795,9606.ENSP00000264110,9606.ENSP00000333685,9606.ENSP00000387699,9606.ENSP00000479667	MAPK14,ATF2,MAPK11,CREB1,RPS6KA5
Reactome	HSA-376172	DSCAM interactions	4	11	2.81	4.08e-08	9606.ENSP00000211287,9606.ENSP00000215659,9606.ENSP00000229795,9606.ENSP00000333685	MAPK13,MAPK12,MAPK14,MAPK11
Reactome	HSA-171007	p38MAPK events	4	13	2.74	5.68e-08	9606.ENSP00000211287,9606.ENSP00000215659,9606.ENSP00000229795,9606.ENSP00000333685	MAPK13,MAPK12,MAPK14,MAPK11
Reactome	HSA-198725	Nuclear Events (kinase and transcription factor activation)	5	61	2.17	6.24e-08	9606.ENSP00000229795,9606.ENSP00000264110,9606.ENSP00000333685,9606.ENSP00000387699,9606.ENSP00000479667	MAPK14,ATF2,MAPK11,CREB1,RPS6KA5
Reactome	HSA-1592230	Mitochondrial biogenesis	5	93	1.98	2.82e-07	9606.ENSP00000215659,9606.ENSP00000229795,9606.ENSP00000264110,9606.ENSP00000333685,9606.ENSP00000387699	MAPK12,MAPK14,ATF2,MAPK11,CREB1
Reactome	HSA-168638	NOD1/2 Signaling Pathway	4	35	2.31	3.55e-07	9606.ENSP00000211287,9606.ENSP00000215659,9606.ENSP00000229795,9606.ENSP00000333685	MAPK13,MAPK12,MAPK14,MAPK11
Reactome	HSA-422475	Axon guidance	7	551	1.36	3.55e-07	9606.ENSP00000211287,9606.ENSP00000215659,9606.ENSP00000229795,9606.ENSP00000333685,9606.ENSP00000333896,9606.ENSP00000387699,9606.ENSP00000479667	MAPK13,MAPK12,MAPK14,MAPK11,RPS6KA4,CREB1,RPS6KA5
Reactome	HSA-450341	Activation of the AP-1 family of transcription factors	3	9	2.78	1.91e-06	9606.ENSP00000229795,9606.ENSP00000264110,9606.ENSP00000333685	MAPK14,ATF2,MAPK11
Reactome	HSA-2151209	Activation of PPARGC1A (PGC-1alpha) by phosphorylation	3	10	2.73	2.41e-06	9606.ENSP00000215659,9606.ENSP00000229795,9606.ENSP00000333685	MAPK12,MAPK14,MAPK11

Reactome	HSA-4420097	VEGFA-VEGFR2 Pathway	4	96	1.87	1.27e-05	9606.ENSP00000211287,9606.ENSP00000215659,9606.ENSP00000229795,9606.ENSP00000333685	MAPK13,MAPK12,MAPK14,MAPK11
Reactome	HSA-198753	ERK/MAPK targets	3	22	2.39	1.77e-05	9606.ENSP00000229795,9606.ENSP00000333685,9606.ENSP00000479667	MAPK14,MAPK11,RPS6KA5
Reactome	HSA-168249	Innate Immune System	7	1041	1.08	1.88e-05	9606.ENSP00000211287,9606.ENSP00000215659,9606.ENSP00000229795,9606.ENSP00000264110,9606.ENSP00000333685,9606.ENSP00000387699,9606.ENSP00000479667	MAPK13,MAPK12,MAPK14,ATF2,MAPK11,CREB1,RPS6KA5
Reactome	HSA-525793	Myogenesis	3	29	2.27	3.46e-05	9606.ENSP00000215659,9606.ENSP00000229795,9606.ENSP00000333685	MAPK12,MAPK14,MAPK11
Reactome	HSA-199920	CREB phosphorylation	2	7	2.71	0.00053	9606.ENSP00000387699,9606.ENSP00000479667	CREB1,RPS6KA5
Reactome	HSA-2262752	Cellular responses to stress	5	747	1.08	0.0015	9606.ENSP00000229795,9606.ENSP00000264110,9606.ENSP00000333685,9606.ENSP00000336790,9606.ENSP00000387699	MAPK14,ATF2,MAPK11,ATF4,CREB1
Reactome	HSA-450604	KSRP (KHSRP) binds and destabilizes mRNA	2	17	2.32	0.0022	9606.ENSP00000229795,9606.ENSP00000333685	MAPK14,MAPK11
Reactome	HSA-450302	Activated TAK1 mediates p38 MAPK activation	2	22	2.21	0.0035	9606.ENSP00000229795,9606.ENSP00000333685	MAPK14,MAPK11
Reactome	HSA-5668599	RHO GTPases Activate NADPH Oxidases	2	24	2.17	0.0041	9606.ENSP00000229795,9606.ENSP00000333685	MAPK14,MAPK11
Reactome	HSA-9031628	NGF-stimulated transcription	2	39	1.96	0.0099	9606.ENSP00000264110,9606.ENSP00000387699	ATF2,CREB1
Reactome	HSA-9707616	Heme signaling	2	46	1.89	0.0133	9606.ENSP00000264110,9606.ENSP00000387699	ATF2,CREB1
Reactome	HSA-437239	Recycling pathway of L1	2	48	1.87	0.0142	9606.ENSP00000333896,9606.ENSP00000479667	RPS6KA4,RPS6KA5
Reactome	HSA-2151201	Transcriptional activation of mitochondrial biogenesis	2	54	1.82	0.0175	9606.ENSP00000264110,9606.ENSP00000387699	ATF2,CREB1

Reactome	HSA-375165	NCAM signaling for neurite out-growth	2	63	1.75	0.0231	9606.ENSP00000387699,9606.ENSP00000479667	CREB1,RPS6KA5
Reactome	HSA-400253	Circadian Clock	2	68	1.72	0.0264	9606.ENSP00000264110,9606.ENSP00000387699	ATF2,CREB1
Reactome	HSA-3700989	Transcriptional Regulation by TP53	3	361	1.17	0.0355	9606.ENSP00000229795,9606.ENSP00000264110,9606.ENSP00000333685	MAPK14,ATF2,MAPK11
Reactome	HSA-9662851	Anti-inflammatory response favouring Leishmania parasite infection	2	80	1.65	0.0355	9606.ENSP00000229795,9606.ENSP00000387699	MAPK14,CREB1
Reactome	HSA-2559580	Oxidative Stress Induced Senescence	2	92	1.59	0.0436	9606.ENSP00000229795,9606.ENSP00000333685	MAPK14,MAPK11
Reactome	HSA-6804756	Regulation of TP53 Activity through Phosphorylation	2	92	1.59	0.0436	9606.ENSP00000229795,9606.ENSP00000333685	MAPK14,MAPK11
Reactome	HSA-9633012	Response of EIF2AK4 (GCN2) to amino acid deficiency	2	100	1.55	0.0497	9606.ENSP00000264110,9606.ENSP00000336790	ATF2,ATF4

Table 12. Pathway Enrichment Analysis for GO, KEGG, STRING clusters and Reactome Terms within TTK gene

#category	term ID	term description	observed gene count	background gene count	strength	false discovery rate	matching proteins in your network (IDs)	matching proteins in your network (labels)
GO Process	GO:0098813	Nuclear chromosome segregation	11	229	1.93	7.51e-18	9606.ENSP00000247191,9606.ENSP00000251496,9606.ENSP00000260731,9606.ENSP00000261597,9606.ENSP00000271452,9606.ENSP00000287598,9606.ENSP00000296509,9606.ENSP00000302530,9606.ENSP00000358813,9606.ENSP00000361540,9606.ENSP00000385334	DLGAP5,NCAPG,KIF11,NDC80,NUF2,BUB1B,MAD2L1,BUB1,TTK,CDC20,MAD1L1
GO Process	GO:1905818	Regulation of chromosome separation	10	109	2.22	7.51e-18	9606.ENSP00000247191,9606.ENSP00000251496,9606.ENSP00000261597,9606.ENSP00000271452,9606.ENSP00000287598,9606.ENSP00000296509,9606.ENSP00000302530,9606.ENSP00000358813,9606.ENSP00000361540,9606.ENSP00000385334	DLGAP5,NCAPG,NDC80,NUF2,BUB1B,MAD2L1,BUB1,TTK,CDC20,MAD1L1
GO Process	GO:0000819	Sister chromatid segregation	10	144	2.09	2.70e-17	9606.ENSP00000247191,9606.ENSP00000251496,9606.ENSP00000260731,9606.ENSP00000261597,9606.ENSP00000271452,9606.ENSP00000287598,9606.ENSP00000296509,9606.ENSP00000302530,9606.ENSP00000361540,9606.ENSP00000385334	DLGAP5,NCAPG,KIF11,NDC80,NUF2,BUB1B,MAD2L1,BUB1,CDC20,MAD1L1
GO Process	GO:0000280	Nuclear division	11	323	1.79	4.68e-17	9606.ENSP00000247191,9606.ENSP00000251496,9606.ENSP00000260731,9606.ENSP00000261597,9606.ENSP00000271452,9606.ENSP00000287598,9606.ENSP00000296509,9606.ENSP00000302530,9606.ENSP00000358813,9606.ENSP00000361540,9606.ENSP00000385334	DLGAP5,NCAPG,KIF11,NDC80,NUF2,BUB1B,MAD2L1,BUB1,TTK,CDC20,MAD1L1
GO Process	GO:0007094	Mitotic spindle assembly checkpoint signaling	8	31	2.66	4.68e-17	9606.ENSP00000261597,9606.ENSP00000271452,9606.ENSP00000287598,9606.ENSP00000296509,9606.ENSP00000302530,9606.ENSP00000358813,9606.ENSP00000361540,9606.ENSP00000385334	NDC80,NUF2,BUB1B,MAD2L1,BUB1,TTK,CDC20,MAD1L1
GO Process	GO:0030071	Regulation of mitotic metaphase/anaphase transition	9	91	2.25	6.94e-17	9606.ENSP00000247191,9606.ENSP00000261597,9606.ENSP00000271452,9606.ENSP00000287598,9606.ENSP00000296509,9606.ENSP00000302530,9606.ENSP00000358813,9606.ENSP00000361540,9606.ENSP00000385334	DLGAP5,NDC80,NUF2,BUB1B,MAD2L1,BUB1,TTK,CDC20,MAD1L1
GO Process	GO:0007088	Regulation of mitotic nuclear division	9	118	2.14	4.90e-16	9606.ENSP00000247191,9606.ENSP00000261597,9606.ENSP00000271452,9606.ENSP00000287598,9606.ENSP00000296509,9606.ENSP00000302530,9606.ENSP00000358813,9606.ENSP00000361540,9606.ENSP00000385334	DLGAP5,NDC80,NUF2,BUB1B,MAD2L1,BUB1,TTK,CDC20,MAD1L1
GO	GO:00	Regulation of	10	253	1.85	9.52e-16	9606.ENSP00000247191,9606.ENSP00000251496,9606.ENS	DLGAP5,NCAPG,N

Process	33044	chromosome organization					P00000261597,9606.ENSP00000271452,9606.ENSP00000287598,9606.ENSP00000296509,9606.ENSP00000302530,9606.ENSP00000358813,9606.ENSP00000361540,9606.ENSP00000385334	DC80,NUF2,BUB1B,MAD2L1,BUB1,TTK,CDC20,MAD1L1
GO Process	GO:1903047	Mitotic cell cycle process	11	537	1.56	3.76e-15	9606.ENSP00000247191,9606.ENSP00000251496,9606.ENSP00000260731,9606.ENSP00000261597,9606.ENSP00000271452,9606.ENSP00000287598,9606.ENSP00000296509,9606.ENSP00000302530,9606.ENSP00000358813,9606.ENSP00000361540,9606.ENSP00000385334	DLGAP5,NCAPG,KIF11,NDC80,NUF2,BUB1B,MAD2L1,BUB1,TTK,CDC20,MAD1L1
GO Process	GO:0010564	Regulation of cell cycle process	11	716	1.44	7.83e-14	9606.ENSP00000247191,9606.ENSP00000251496,9606.ENSP00000260731,9606.ENSP00000261597,9606.ENSP00000271452,9606.ENSP00000287598,9606.ENSP00000296509,9606.ENSP00000302530,9606.ENSP00000358813,9606.ENSP00000361540,9606.ENSP00000385334	DLGAP5,NCAPG,KIF11,NDC80,NUF2,BUB1B,MAD2L1,BUB1,TTK,CDC20,MAD1L1
GO Process	GO:0000070	Mitotic sister chromatid segregation	8	122	2.07	2.22e-13	9606.ENSP00000247191,9606.ENSP00000251496,9606.ENSP00000260731,9606.ENSP00000261597,9606.ENSP00000271452,9606.ENSP00000296509,9606.ENSP00000361540,9606.ENSP00000385334	DLGAP5,NCAPG,KIF11,NDC80,NUF2,MAD2L1,CDC20,MAD1L1
GO Process	GO:1902850	Microtubule cytoskeleton organization involved in mitosis	7	129	1.99	7.16e-11	9606.ENSP00000247191,9606.ENSP00000260731,9606.ENSP00000261597,9606.ENSP00000271452,9606.ENSP00000296509,9606.ENSP00000358813,9606.ENSP00000361540	DLGAP5,KIF11,NDC80,NUF2,MAD2L1,TTK,CDC20
GO Process	GO:0051301	Cell division	9	527	1.49	1.30e-10	9606.ENSP00000251496,9606.ENSP00000260731,9606.ENSP00000261597,9606.ENSP00000271452,9606.ENSP00000287598,9606.ENSP00000296509,9606.ENSP00000302530,9606.ENSP00000361540,9606.ENSP00000385334	NCAPG,KIF11,NDC80,NUF2,BUB1B,MAD2L1,BUB1,CDC20,MAD1L1
GO Process	GO:0007052	Mitotic spindle organization	6	97	2.04	2.35e-09	9606.ENSP00000247191,9606.ENSP00000260731,9606.ENSP00000261597,9606.ENSP00000271452,9606.ENSP00000358813,9606.ENSP00000361540	DLGAP5,KIF11,NDC80,NUF2,TTK,CDC20
GO Process	GO:0140013	Meiotic nuclear division	5	171	1.72	6.31e-06	9606.ENSP00000261597,9606.ENSP00000271452,9606.ENSP00000287598,9606.ENSP00000302530,9606.ENSP00000358813	NDC80,NUF2,BUB1B,BUB1,TTK
GO Process	GO:0090267	Positive regulation of mitotic cell cycle spindle assembly checkpoint	3	12	2.65	1.48e-05	9606.ENSP00000261597,9606.ENSP00000296509,9606.ENSP00000385334	NDC80,MAD2L1,MAD1L1
GO Process	GO:0051315	Attachment of mitotic spindle microtubules to	3	13	2.62	1.77e-05	9606.ENSP00000261597,9606.ENSP00000271452,9606.ENSP00000385334	NDC80,NUF2,MAD1L1

		kinetochore						
GO Process	GO:0050000	Chromosome localization	4	84	1.93	2.88e-05	9606.ENSP00000247191,9606.ENSP00000261597,9606.ENSP00000271452,9606.ENSP00000385334	DLGAP5,NDC80,NUF2,MAD1L1
GO Process	GO:0090068	Positive regulation of cell cycle process	5	251	1.55	3.61e-05	9606.ENSP00000247191,9606.ENSP00000251496,9606.ENSP00000261597,9606.ENSP00000296509,9606.ENSP00000385334	DLGAP5,NCAPG,NDC80,MAD2L1,MAD1L1
GO Process	GO:0045132	Meiotic chromosome segregation	4	98	1.86	4.86e-05	9606.ENSP00000271452,9606.ENSP00000287598,9606.ENSP00000302530,9606.ENSP00000358813	NUF2,BUB1B,BUB1,TTK
GO Process	GO:0051383	Kinetochore organization	3	21	2.41	5.74e-05	9606.ENSP00000247191,9606.ENSP00000261597,9606.ENSP00000271452	DLGAP5,NDC80,NUF2
GO Process	GO:0051444	Negative regulation of ubiquitin-protein transferase activity	3	23	2.37	6.98e-05	9606.ENSP00000287598,9606.ENSP00000296509,9606.ENSP00000361540	BUB1B,MAD2L1,CDC20
GO Process	GO:1905820	Positive regulation of chromosome separation	3	28	2.28	0.00012	9606.ENSP00000247191,9606.ENSP00000251496,9606.ENSP00000385334	DLGAP5,NCAPG,MAD1L1
GO Process	GO:0007062	Sister chromatid cohesion	3	42	2.11	0.00035	9606.ENSP00000287598,9606.ENSP00000302530,9606.ENSP00000361540	BUB1B,BUB1,CDC20
GO Process	GO:0051640	Organelle localization	5	514	1.24	0.00092	9606.ENSP00000247191,9606.ENSP00000261597,9606.ENSP00000271452,9606.ENSP00000296509,9606.ENSP00000385334	DLGAP5,NDC80,NUF2,MAD2L1,MAD1L1
GO Process	GO:0051754	Meiotic sister chromatid cohesion, centromeric	2	5	2.86	0.0011	9606.ENSP00000287598,9606.ENSP00000302530	BUB1B,BUB1
GO Process	GO:0051225	Spindle assembly	3	96	1.75	0.0034	9606.ENSP00000260731,9606.ENSP00000261597,9606.ENSP00000361540	KIF11,NDC80,CDC20
GO Process	GO:0140694	Non-membrane-bounded organelle assembly	4	314	1.36	0.0034	9606.ENSP00000247191,9606.ENSP00000260731,9606.ENSP00000261597,9606.ENSP00000361540	DLGAP5,KIF11,NDC80,CDC20
GO Process	GO:0045842	Positive regulation of mitotic metaphase/anaphase transition	2	15	2.38	0.0063	9606.ENSP00000247191,9606.ENSP00000385334	DLGAP5,MAD1L1
GO Process	GO:0051656	Establishment of organelle localization	4	380	1.28	0.0069	9606.ENSP00000261597,9606.ENSP00000271452,9606.ENSP00000296509,9606.ENSP00000385334	NDC80,NUF2,MAD2L1,MAD1L1
GO Process	GO:0051984	Positive regulation of chromosome segregation	2	26	2.14	0.0162	9606.ENSP00000251496,9606.ENSP00000385334	NCAPG,MAD1L1

GO Process	GO:1904666	Regulation of ubiquitin protein ligase activity	2	26	2.14	0.0162	9606.ENSP00000296509,9606.ENSP00000361540	MAD2L1,CDC20
GO Process	GO:0023052	Signaling	9	5057	0.5	0.0237	9606.ENSP00000247191,9606.ENSP00000261597,9606.ENSP00000271452,9606.ENSP00000287598,9606.ENSP00000296509,9606.ENSP00000302530,9606.ENSP00000358813,9606.ENSP00000361540,9606.ENSP00000385334	DLGAP5,NDC80,NUF2,BUB1B,MAD2L1,BUB1,TTK,CDC20,MAD1L1
GO Process	GO:0051642	Centrosome localization	2	33	2.04	0.0247	9606.ENSP00000247191,9606.ENSP00000296509	DLGAP5,MAD2L1
GO Process	GO:0000132	Establishment of mitotic spindle orientation	2	34	2.02	0.0259	9606.ENSP00000261597,9606.ENSP00000296509	NDC80,MAD2L1
GO Process	GO:0007143	Female meiotic nuclear division	2	34	2.02	0.0259	9606.ENSP00000261597,9606.ENSP00000358813	NDC80,TTK
GO Process	GO:0071459	Protein localization to chromosome, centromeric region	2	41	1.94	0.0354	9606.ENSP00000287598,9606.ENSP00000358813	BUB1B,TTK
GO Function	GO:0043515	Kinetochore binding	2	6	2.78	0.0390	9606.ENSP00000358813,9606.ENSP00000385334	TTK,MAD1L1
GO Component	GO:0000779	Condensed chromosome, centromeric region	8	175	1.91	1.56e-11	9606.ENSP00000251496,9606.ENSP00000261597,9606.ENSP00000271452,9606.ENSP00000287598,9606.ENSP00000296509,9606.ENSP00000302530,9606.ENSP00000358813,9606.ENSP00000385334	NCAPG,NDC80,NUF2,BUB1B,MAD2L1,BUB1,TTK,MAD1L1
GO Component	GO:0000776	Kinetochore	7	165	1.88	5.59e-10	9606.ENSP00000261597,9606.ENSP00000271452,9606.ENSP00000287598,9606.ENSP00000296509,9606.ENSP00000302530,9606.ENSP00000358813,9606.ENSP00000385334	NDC80,NUF2,BUB1B,MAD2L1,BUB1,TTK,MAD1L1
GO Component	GO:0005819	Spindle	7	425	1.47	2.42e-07	9606.ENSP00000247191,9606.ENSP00000260731,9606.ENSP00000287598,9606.ENSP00000296509,9606.ENSP00000358813,9606.ENSP00000361540,9606.ENSP00000385334	DLGAP5,KIF11,BUB1B,MAD2L1,TTK,CDC20,MAD1L1
GO Component	GO:0033597	Mitotic checkpoint complex	3	4	3.13	1.32e-06	9606.ENSP00000287598,9606.ENSP00000296509,9606.ENSP00000361540	BUB1B,MAD2L1,CDC20
GO Component	GO:0000922	Spindle pole	5	169	1.72	5.73e-06	9606.ENSP00000247191,9606.ENSP00000260731,9606.ENSP00000296509,9606.ENSP00000361540,9606.ENSP00000385334	DLGAP5,KIF11,MAD2L1,CDC20,MAD1L1
GO Component	GO:0015630	Microtubule cytoskeleton	8	1355	1.02	1.59e-05	9606.ENSP00000247191,9606.ENSP00000260731,9606.ENSP00000261597,9606.ENSP00000287598,9606.ENSP00000296509,9606.ENSP00000358813,9606.ENSP00000361540,9606.ENSP00000385334	DLGAP5,KIF11,NDC80,BUB1B,MAD2L1,TTK,CDC20,MAD1L1

GO Component	GO:0099080	Supramolecular complex	8	1366	1.02	1.59e-05	9606.ENSP00000260731,9606.ENSP00000261597,9606.ENSP00000271452,9606.ENSP00000287598,9606.ENSP00000296509,9606.ENSP00000302530,9606.ENSP00000358813,9606.ENSP00000385334	KIF11,NDC80,NUF2,BUB1B,MAD2L1,BUB1,TTK,MAD1L1
GO Component	GO:0043232	Intracellular non-membrane-bounded organelle	11	5191	0.58	7.99e-05	9606.ENSP00000247191,9606.ENSP00000251496,9606.ENSP00000260731,9606.ENSP00000261597,9606.ENSP00000271452,9606.ENSP00000287598,9606.ENSP00000296509,9606.ENSP00000302530,9606.ENSP00000358813,9606.ENSP00000361540,9606.ENSP00000385334	DLGAP5,NCAPG,KIF11,NDC80,NUF2,BUB1B,MAD2L1,BUB1,TTK,CDC20,MAD1L1
GO Component	GO:1990728	Mitotic spindle assembly checkpoint MAD1-MAD2 complex	2	2	3.25	0.00025	9606.ENSP00000296509,9606.ENSP00000385334	MAD2L1,MAD1L1
GO Component	GO:0031262	Ndc80 complex	2	4	2.95	0.00058	9606.ENSP00000261597,9606.ENSP00000271452	NDC80,NUF2
GO Component	GO:0005829	Cytosol	10	5438	0.52	0.0024	9606.ENSP00000247191,9606.ENSP00000251496,9606.ENSP00000260731,9606.ENSP00000261597,9606.ENSP00000271452,9606.ENSP00000287598,9606.ENSP00000296509,9606.ENSP00000302530,9606.ENSP00000361540,9606.ENSP00000385334	DLGAP5,NCAPG,KIF11,NDC80,NUF2,BUB1B,MAD2L1,BUB1,CDC20,MAD1L1
GO Component	GO:0000940	Outer kinetochore	2	12	2.47	0.0028	9606.ENSP00000261597,9606.ENSP00000287598	NDC80,BUB1B
GO Component	GO:0044615	Nuclear pore nuclear basket	2	12	2.47	0.0028	9606.ENSP00000296509,9606.ENSP00000385334	MAD2L1,MAD1L1
GO Component	GO:0005634	Nucleus	11	7672	0.41	0.0031	9606.ENSP00000247191,9606.ENSP00000251496,9606.ENSP00000260731,9606.ENSP00000261597,9606.ENSP00000271452,9606.ENSP00000287598,9606.ENSP00000296509,9606.ENSP00000302530,9606.ENSP00000358813,9606.ENSP00000361540,9606.ENSP00000385334	DLGAP5,NCAPG,KIF11,NDC80,NUF2,BUB1B,MAD2L1,BUB1,TTK,CDC20,MAD1L1
GO Component	GO:0005815	Microtubule organizing center	5	825	1.04	0.0045	9606.ENSP00000247191,9606.ENSP00000261597,9606.ENSP00000287598,9606.ENSP00000361540,9606.ENSP00000385334	DLGAP5,NDC80,BUB1B,CDC20,MAD1L1
GO Component	GO:0005680	Anaphase-promoting complex	2	22	2.21	0.0069	9606.ENSP00000287598,9606.ENSP00000361540	BUB1B,CDC20
GO	GO:00	Mitotic spindle	3	180	1.47	0.0105	9606.ENSP00000260731,9606.ENSP00000296509,9606.ENS	KIF11,MAD2L1,MA

Component	72686						P00000385334	D1L1
GO Component	GO:0044815	DNA packaging complex	3	208	1.41	0.0153	9606.ENSP00000251496,9606.ENSP00000261597,9606.ENSP00000271452	NCAPG,NDC80,NUF2
GO Component	GO:0005813	Centrosome	4	609	1.07	0.0202	9606.ENSP00000247191,9606.ENSP00000261597,9606.ENSP00000361540,9606.ENSP00000385334	DLGAP5,NDC80,CDC20,MAD1L1
STRING clusters	CL:6596	Mixed, incl. Mitotic Spindle Checkpoint, and Mitotic sister chromatid segregation	11	151	2.12	3.75e-20	9606.ENSP00000247191,9606.ENSP00000251496,9606.ENSP00000260731,9606.ENSP00000261597,9606.ENSP00000271452,9606.ENSP00000287598,9606.ENSP00000296509,9606.ENSP00000302530,9606.ENSP00000358813,9606.ENSP00000361540,9606.ENSP00000385334	DLGAP5,NCAPG,KIF11,NDC80,NUF2,BUB1B,MAD2L1,BUB1,TTK,CDC20,MAD1L1
STRING clusters	CL:6604	Mixed, incl. Amplification of signal from the kinetochores, and Condensin complex	10	93	2.28	2.43e-19	9606.ENSP00000247191,9606.ENSP00000251496,9606.ENSP00000260731,9606.ENSP00000261597,9606.ENSP00000271452,9606.ENSP00000287598,9606.ENSP00000302530,9606.ENSP00000358813,9606.ENSP00000361540,9606.ENSP00000385334	DLGAP5,NCAPG,KIF11,NDC80,NUF2,BUB1B,BUB1,TTK,CDC20,MAD1L1
STRING clusters	CL:6606	Mitotic sister chromatid segregation, and Mitotic spindle checkpoint signaling	8	61	2.37	1.85e-15	9606.ENSP00000247191,9606.ENSP00000251496,9606.ENSP00000260731,9606.ENSP00000287598,9606.ENSP00000302530,9606.ENSP00000358813,9606.ENSP00000361540,9606.ENSP00000385334	DLGAP5,NCAPG,KIF11,BUB1B,BUB1,TTK,CDC20,MAD1L1
STRING clusters	CL:6608	Mixed, incl. Regulation of mitotic sister chromatid segregation, and Kinesin motor domain, conserved site	7	51	2.39	2.81e-13	9606.ENSP00000247191,9606.ENSP00000260731,9606.ENSP00000287598,9606.ENSP00000302530,9606.ENSP00000358813,9606.ENSP00000361540,9606.ENSP00000385334	DLGAP5,KIF11,BUB1B,BUB1,TTK,CDC20,MAD1L1
STRING clusters	CL:6695	RZZ complex, and Kinetochore binding	3	5	3.03	4.13e-06	9606.ENSP00000302530,9606.ENSP00000358813,9606.ENSP00000385334	BUB1,TTK,MAD1L1
STRING clusters	CL:6619	Mixed, incl. Spindle elongation, and Outer kinetochore	3	24	2.35	0.00019	9606.ENSP00000247191,9606.ENSP00000260731,9606.ENSP00000287598	DLGAP5,KIF11,BUB1B
STRING clusters	CL:6622	Mixed, incl. Mitotic centrosome separation, and	2	7	2.71	0.0036	9606.ENSP00000247191,9606.ENSP00000260731	DLGAP5,KIF11

		Nuclear microtubule						
STRING clusters	CL:6728	Centromere complex assembly, and NMS complex	2	31	2.06	0.0452	9606.ENSP00000261597,9606.ENSP00000271452	NDC80,NUF2
KEGG	hsa04110	Cell cycle	6	120	1.95	9.15e-09	9606.ENSP00000287598,9606.ENSP00000296509,9606.ENSP00000302530,9606.ENSP00000358813,9606.ENSP00000361540,9606.ENSP00000385334	BUB1B,MAD2L1,BUB1,TTK,CDC20,MAD1L1
KEGG	hsa04114	Oocyte meiosis	4	121	1.77	8.26e-05	9606.ENSP00000296509,9606.ENSP00000302530,9606.ENSP00000361540,9606.ENSP00000385334	MAD2L1,BUB1,CDC20,MAD1L1
KEGG	hsa05166	Human T-cell leukemia virus 1 infection	4	210	1.53	0.00047	9606.ENSP00000287598,9606.ENSP00000296509,9606.ENSP00000361540,9606.ENSP00000385334	BUB1B,MAD2L1,CDC20,MAD1L1
KEGG	hsa04914	Progesterone-mediated oocyte maturation	3	95	1.75	0.0016	9606.ENSP00000296509,9606.ENSP00000302530,9606.ENSP00000385334	MAD2L1,BUB1,MAD1L1
Reactome	HSA-141444	Amplification of signal from unattached kinetochores via a MAD2 inhibitory signal	7	94	2.13	5.12e-11	9606.ENSP00000261597,9606.ENSP00000271452,9606.ENSP00000287598,9606.ENSP00000296509,9606.ENSP00000302530,9606.ENSP00000361540,9606.ENSP00000385334	NDC80,NUF2,BUB1B,MAD2L1,BUB1,CDC20,MAD1L1
Reactome	HSA-68877	Mitotic Prometaphase	8	201	1.85	5.12e-11	9606.ENSP00000251496,9606.ENSP00000261597,9606.ENSP00000271452,9606.ENSP00000287598,9606.ENSP00000296509,9606.ENSP00000302530,9606.ENSP00000361540,9606.ENSP00000385334	NCAPG,NDC80,NUF2,BUB1B,MAD2L1,BUB1,CDC20,MAD1L1
Reactome	HSA-9648025	EML4 and NUDC in mitotic spindle formation	7	116	2.03	5.12e-11	9606.ENSP00000261597,9606.ENSP00000271452,9606.ENSP00000287598,9606.ENSP00000296509,9606.ENSP00000302530,9606.ENSP00000361540,9606.ENSP00000385334	NDC80,NUF2,BUB1B,MAD2L1,BUB1,CDC20,MAD1L1
Reactome	HSA-2500257	Resolution of Sister Chromatid Cohesion	7	125	2.0	6.32e-11	9606.ENSP00000261597,9606.ENSP00000271452,9606.ENSP00000287598,9606.ENSP00000296509,9606.ENSP00000302530,9606.ENSP00000361540,9606.ENSP00000385334	NDC80,NUF2,BUB1B,MAD2L1,BUB1,CDC20,MAD1L1
Reactome	HSA-5663220	RHO GTPases Activate Formins	7	139	1.96	1.11e-10	9606.ENSP00000261597,9606.ENSP00000271452,9606.ENSP00000287598,9606.ENSP00000296509,9606.ENSP00000302530,9606.ENSP00000361540,9606.ENSP00000385334	NDC80,NUF2,BUB1B,MAD2L1,BUB1,CDC20,MAD1L1
Reactome	HSA-2467813	Separation of Sister Chromatids	7	189	1.82	7.86e-10	9606.ENSP00000261597,9606.ENSP00000271452,9606.ENSP00000287598,9606.ENSP00000296509,9606.ENSP00000302530,9606.ENSP00000361540,9606.ENSP00000385334	NDC80,NUF2,BUB1B,MAD2L1,BUB1,CDC20,MAD1L1
Reactome	HSA-14143	Inactivation of APC/C via direct	3	21	2.41	3.30e-05	9606.ENSP00000287598,9606.ENSP00000296509,9606.ENSP00000361540	BUB1B,MAD2L1,CDC20

	0	inhibition of the APC/C complex						
Reactome	HSA-179409	APC-Cdc20 mediated degradation of Nek2A	3	26	2.32	5.35e-05	9606.ENSP00000287598,9606.ENSP00000296509,9606.ENSP00000361540	BUB1B,MAD2L1,CDC20
Reactome	HSA-162582	Signal Transduction	8	2540	0.75	0.00093	9606.ENSP00000247191,9606.ENSP00000261597,9606.ENSP00000271452,9606.ENSP00000287598,9606.ENSP00000296509,9606.ENSP00000302530,9606.ENSP00000361540,9606.ENSP00000385334	DLGAP5,NDC80,NUF2,BUB1B,MAD2L1,BUB1,CDC20,MAD1L1
Reactome	HSA-174184	Cdc20:Phospho-APC/C mediated degradation of Cyclin A	3	72	1.87	0.00093	9606.ENSP00000287598,9606.ENSP00000296509,9606.ENSP00000361540	BUB1B,MAD2L1,CDC20
